# The impact of COVID-19 social isolation and reduced microbial exposure on the immune system in children: a retrospective study

**DOI:** 10.7717/peerj.21469

**Published:** 2026-07-07

**Authors:** Xuan Wu, Shiwei Li, Yahong Li, Dongxia Shi, Ping Chen

**Affiliations:** 1 Precision Medicine Laboratory, The Tianshui First People’s Hospital, Tianshui, Gansu, China; 2 Geriatrics Department, Tianshui First People’s Hospital, Tianshui, Gansu, China

**Keywords:** COVID-19, Children, Immune development, Immune cells, Immunoglobulin, Epidemiological measures

## Abstract

**Objective:**

To assess the impact of the COVID-19 pandemic and associated containment measures on the development of the immune system in children.

**Methods:**

This retrospective study analyzed clinical data from children aged 0–13 years diagnosed with respiratory tract infections at Tianshui First People’s Hospital between 2019 and 2024. The dataset included 79,703 complete blood count samples and 7,931 immunoglobulin (IgG, IgA, IgM) analysis samples. Non-parametric tests (rank-sum test) and generalized linear models (Gamma distribution with Log link function) were employed for statistical analysis to evaluate the effects of different years, age groups, genders, and diagnostic types (upper/lower respiratory tract infections) on immunological parameters.

**Results:**

The study found that the COVID-19 pandemic had heterogeneous effects on various immune cell types. Neutrophil and monocyte counts showed an increasing trend during the pandemic, whereas lymphocyte, eosinophil, and basophil counts were suppressed—with lymphocyte reduction being particularly pronounced. The suppressive effect on basophils may be persistent. Compared with 2019, no significant changes were observed in immunoglobulin levels among children from 2020 to 2022. However, beginning in 2023, serum concentrations of immunoglobulin G and immunoglobulin M showed a marked increase. This upward trend persisted through 2024, accompanied by a concurrent rebound in lymphocyte counts. Further analysis of interaction effects among age, gender, and diagnosis revealed that the impact of the pandemic on immune development was also significantly influenced by these factors.

**Conclusion:**

The COVID-19 pandemic and associated public health interventions have altered children’s patterns of pathogen exposure, delayed the development of adaptive immunity, and disrupted the balance between innate and adaptive immunity, thereby exerting complex and long-lasting effects on immune system maturation. These findings provide valuable data for understanding the response patterns of the children’s immune system under exceptional public health events and offer guidance for formulating targeted child health policies, vaccination strategies, and clinical practices in the future.

## Introduction

The COVID-19 pandemic has profoundly impacted global public health defense systems and resulted in significant mortality worldwide. However, studies indicate that the disease severity is relatively lower in children compared to other age groups (Howard-Jones et al., 2022). However, the underlying mechanisms remain unclear. Current research predominantly focuses on the clinical manifestations and disease characteristics of pediatric COVID-19 infection, as well as vaccination (Tang et al., 2022). However, the impact of the COVID-19 pandemic on child health extends far beyond this aspect. China implemented stringent containment measures during the outbreak, including widespread home confinement, to reduce viral transmission. These measures not only effectively controlled the spread of the virus but also considerably curtailed children’s exposure to external environments, thereby limiting their opportunities for pathogen encounter. It is well established that exposure to pathogens plays a crucial role in the development of the immune system during childhood (Pieren, Boer & De Wit, 2022). However, studies investigating the long-term impact of the COVID-19 pandemic on the children’s immune system are still limited, particularly regarding its specific effect on immune responses to respiratory pathogens—a research direction of significant importance. The present study systematically collected clinical data on immune cells and immunological molecules from children with respiratory tract infections between 2019 and 2024, aiming to objectively characterize the impact and temporal trends of the COVID-19 pandemic on these pediatric immune parameters. Furthermore, the results can offer a theoretical basis for public health authorities in formulating future strategies, such as considering expanded vaccine coverage and increased vaccination frequency for children, along with other comprehensive measures to enhance immune function, thereby mitigating the long-term effects of similar public health emergencies on immune development in children.

## Materials & Methods

### Study population

All data in this study were obtained from pediatric patients aged 0–13 years (including both inpatients and outpatients) who visited Tianshui First People’s Hospital between 2019 and 2024, comprising 79,703 complete blood count (CBC) specimens and 7,931 immunoglobulin (Ig) analysis specimens. The numbers of immune cell specimens collected from 2019 to 2024 were 5,557, 1,120, 3,401, 7,927, 29,384, and 32,314, respectively. Similarly, the numbers of immune molecule specimens for the same period were 1,012, 240, 634, 1,820, 1,994, and 2,231. Due to the retrospective nature of the study, there were a small number of missing data points, primarily due to sample hemolysis, failed assays, or incomplete medical records. Cases with missing values for the primary outcome measures (*e.g.*, specific immune cell counts or immunoglobulin levels) were excluded from the final analysis, including 112 immune cell specimens and five immunoglobulin specimens. The inclusion criteria for this study were patients clinically diagnosed with respiratory tract infections, including those with concurrent non-infectious conditions. Since individuals with asymptomatic respiratory pathogen infections have not visited the hospital for medical care, they were therefore excluded from this study. Informed consent was obtained from all participants or their legal guardians prior to enrollment. All data on immune cells, immune molecules, and clinical parameters were extracted from the hospital’s medical information system.

Given the stage-specific development of the immune system in children and the distinct maturation timelines of different immune cells and molecules, different age-grouping criteria were applied for their respective analyses in this study. The grouping was conducted with reference to the National Health Industry Standard of the People’s Republic of China (WS/T 779-2021).

For CBC analysis, patients were categorized into the following age groups:

Group 1: 0–3 months,

Group 2: 3 months–4 years,

Group 3: 4–6 years,

Group 4: 6–13 years.

For immunoglobulin analysis, patients were grouped as follows:

Group 1: 0–1 year,

Group 2: 1–4 years,

Group 3: 4–6 years,

Group 4: 6–13 years.

### Definitions

1. Patients with upper respiratory tract infections (URTIs) must meet inclusion criteria as follows: cough, runny nose, nasal congestion, sore throat, red and swollen tonsils, no symptoms of bronchial or pulmonary infection;

2. Patients with lower respiratory tract infections (LRTIs) include pneumonia (including lobar pneumonia), pulmonary infection, respiratory distress syndrome and respiratory failure.

### Statistics and ethics

The statistical analyses in this study were performed using SPSS Statistics (IBM Corp., Armonk, NY, USA) 23 and GraphPad Prism 10.6. Non-parametric tests and generalized linear models were employed for data analysis. As all datasets exhibited a positively skewed distribution, the rank-sum test (under non-parametric methods) was applied to assess differences among groups. Generalized linear modeling with Gamma distribution and Log link function was used to identify major influencing factors and interaction effects. A significance threshold of *p* < 0.05 was applied for inclusion (*). The COVID-19 outbreak in Tianshui City was first documented in February 2020; therefore, immunological data from 2019 were used as the baseline reference for assessing children’s immune status.

This study was approved by the Ethics Review Committee of the First People’s Hospital of Tianshui. The IRB approval number was 2023-07.

### Experimental method

**1. CBC analysis**

• **Instrument:** The BC-6800Plus automated hematology analyzer (Mindray Medical International Limited) was used for CBC analysis.
• **Methodology:** The instrument employs a combination of methodologies, including electrical impedance, radiofrequency analysis, flow cytometry, and fluorescent staining for comprehensive cell counting and differentiation.
• **Medical Device Registration No.:** Guangdong Medical Equipment registration 20172401464
• **Reagents:** All reagents were supplied by Mindray. Specific reagents, their batch numbers, and medical device filing certificate numbers are detailed below:
  ∘ Hemolytic Agent (M-68P LD): Batch No. 2019021312 (Filing Certificate: Suning Medical Equipment 20170025, Hangzhou, China).
  ∘ Hemolytic Agent (M-68P LN): Batch No. 2019021023 (Filing Certificate: Suning Medical Equipment 20170018, Hangzhou, China).
  ∘ Diluent (M-68P DR): Batch No. 2019012318 (Filing Certificate: Suning Medical Equipment 20170019, Hangzhou, China).
  ∘ Stain (M-68P FN): Batch No. 2019021129 (Filing Certificate: Suning Medical Equipment 20170014, Hangzhou, China).
  ∘ Stain (M-68P FR): Batch No. 2019021324 (Filing Certificate: Suning Medical Equipment 20170025, Hangzhou, China).
  ∘ Stain (M-68P FD): Batch No. 2019012359 (Filing Certificate: Suning Medical Equipment 20170025, Hangzhou, China).
• **Sample Type:** EDTA-K_2_ anticoagulated whole blood.
• **Parameters Analyzed:** White blood cell count (WBC), neutrophil count, lymphocyte count, monocyte count, eosinophil count, and basophil count.
• **Units:** ×10^9^/L.

**2. Immunoglobulin analysis**

• **Instrument:** The IMMAGE 800 immunochemistry system (Beckman Coulter, Brea, CA, USA) was used for quantitative immunoglobulin analysis.
• **Methodology:** Analyses were performed using rate nephelometry (rate scatter immunoassay) and rate inhibition nephelometry.
• **Medical Device Registration No.:** Su Ji registered 20192220361
• **Reagents:** Immunoglobulin-specific reagent kits (Beckman Coulter, Brea, CA, USA) were used:
  ∘ Immunoglobulin G (IgG) Test Kit: Batch No. M405443 (Registration No.: National medical equipment injection 20152400549)
  ∘ Immunoglobulin M (IgM) Test Kit: Batch No. M412235 (Registration No.: National medical equipment injection 20152400749)
  ∘ Immunoglobulin A (IgA) Test Kit: Batch No. M410286 (Registration No.: National medical equipment injection 20152400299)
• **Sample Type:** Serum.
• **Parameters Analyzed:** IgG, IgM, IgA concentrations.
• **Units:** g/L

All detection in this study was performed in the laboratory of Tianshui First People’s Hospital. All instruments were calibrated and underwent performance validation, experimental methods were verified, all reagents underwent lot-specific validation. Ambient laboratory conditions were maintained within a temperature range of 19–26 °C and a relative humidity range of 35–48% RH.

## Results

### Immune cells

**Figure 1:**
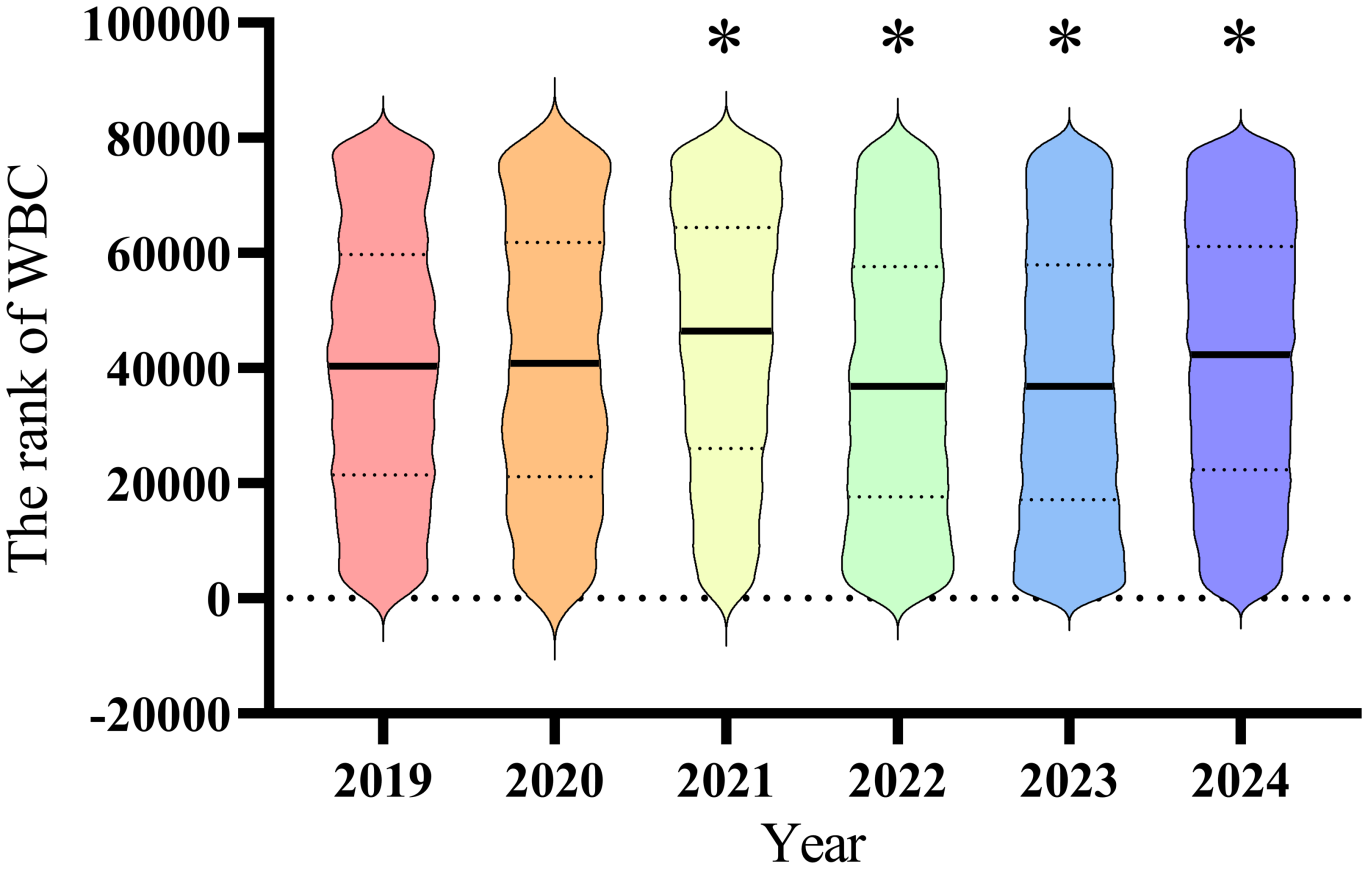
Violin plots with embedded boxplots illustrating the distribution of WBC counts rank from 2019 to 2024, analyzed using the Kruskal–Wallis rank-sum test. An asterisk (*) denotes a statistically significant difference (*p* < 0.05) compared to the 2019 reference group.

**Figure 2:**
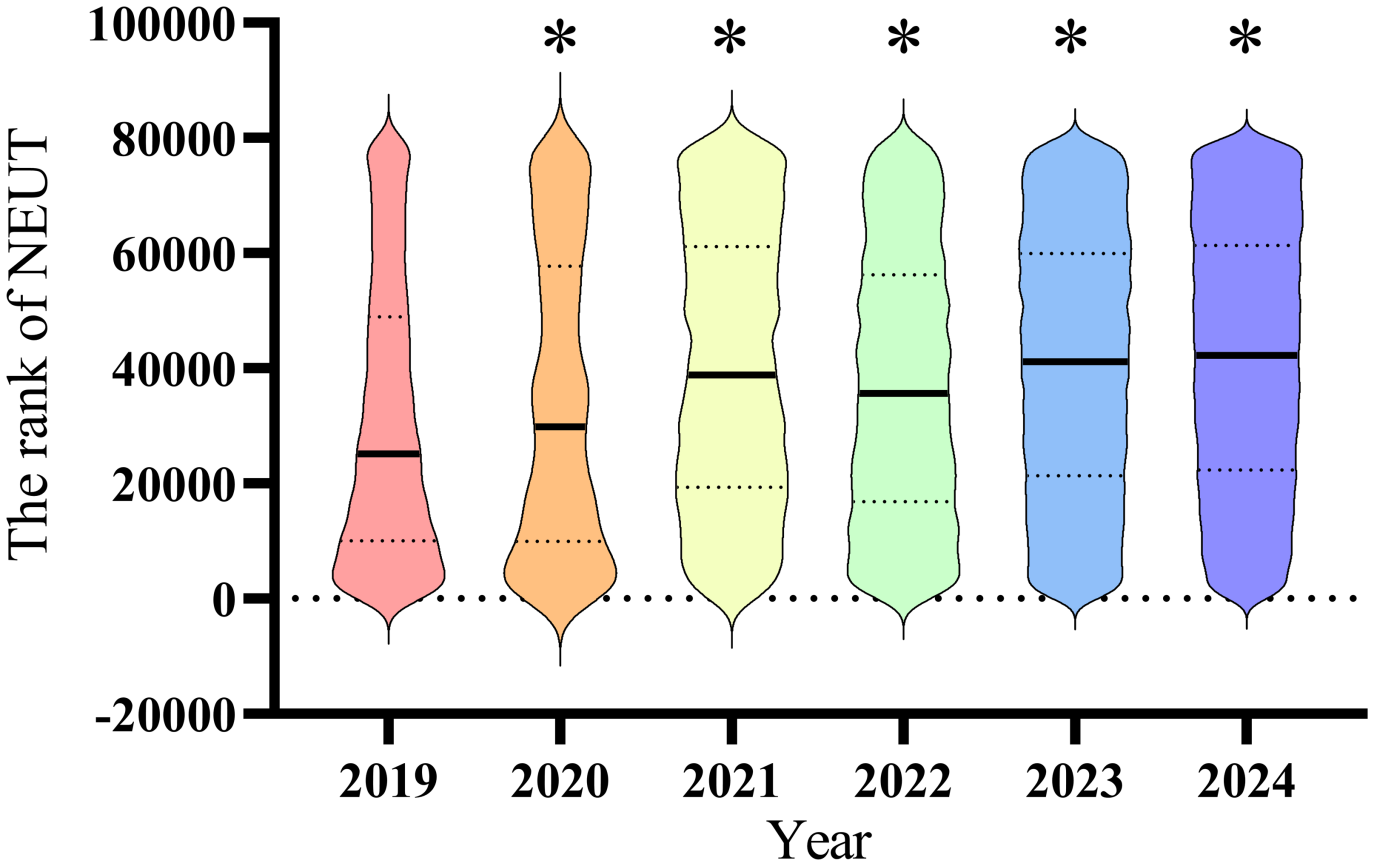
Violin plots with embedded boxplots illustrating the distribution of neutrophil counts rank from 2019 to 2024, analyzed using the Kruskal–Wallis rank-sum test. An asterisk (*) denotes a statistically significant difference (*p* < 0.05) compared to the 2019 reference group.

**Figure 3:**
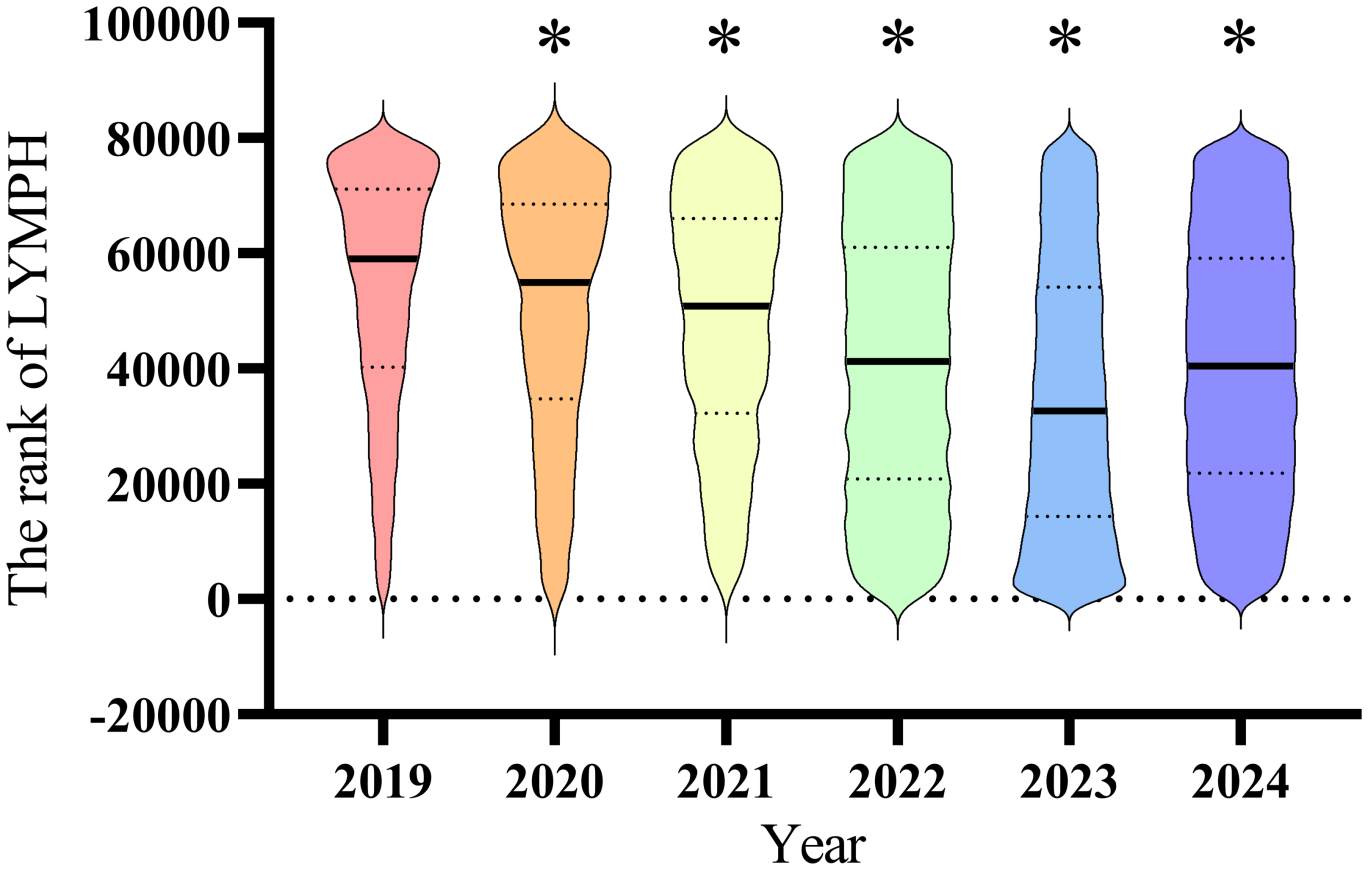
Violin plots with embedded boxplots illustrating the distribution of lymphocyte counts rank from 2019 to 2024, analyzed using the Kruskal–Wallis rank-sum test. An asterisk (*) denotes a statistically significant difference (*p* < 0.05) compared to the 2019 reference group.

**Figure 4:**
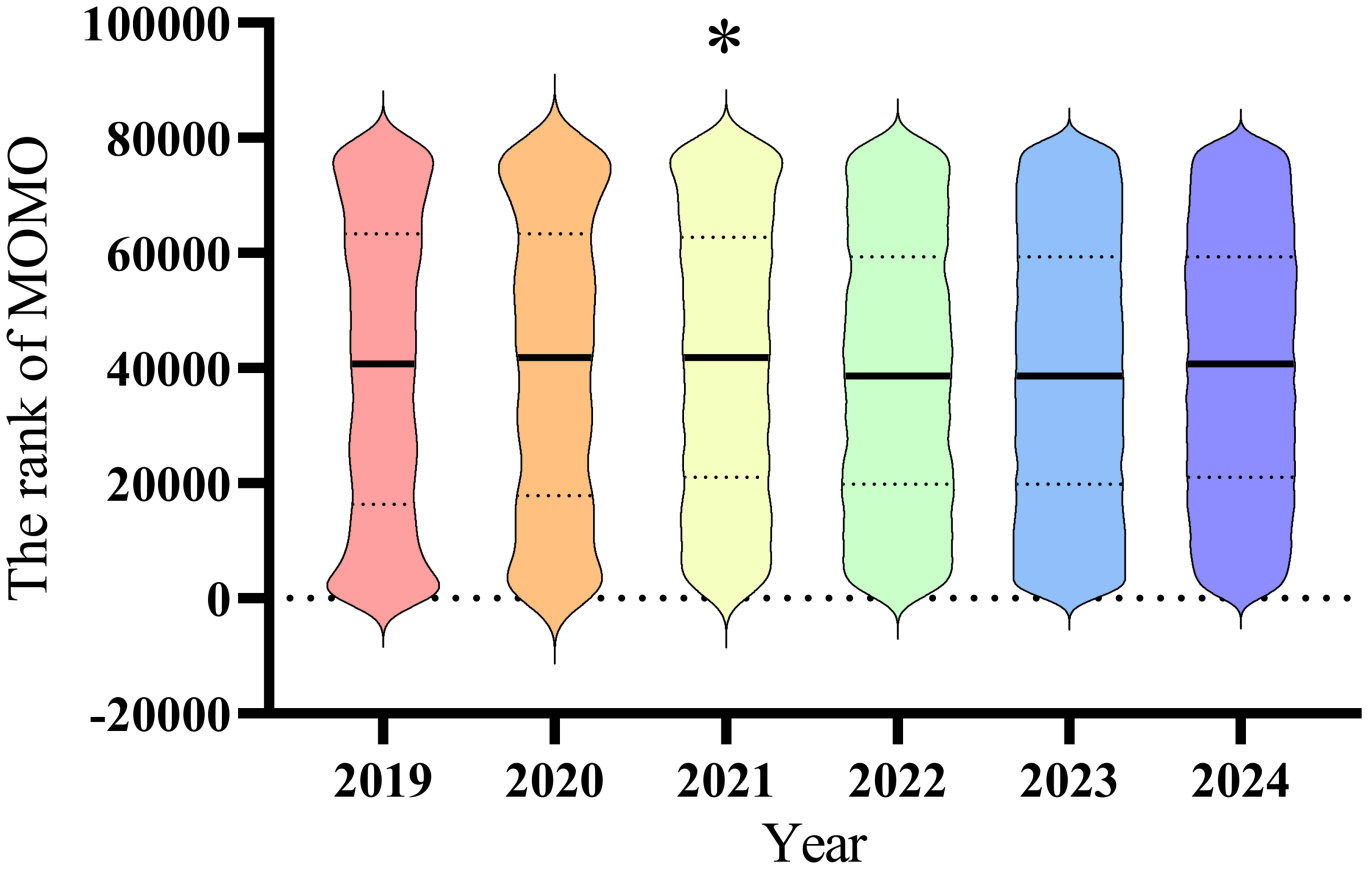
Violin plots with embedded boxplots illustrating the distribution of monocyte counts rank from 2019 to 2024, analyzed using the Kruskal–Wallis rank-sum test. An asterisk (*) denotes a statistically significant difference (*p* < 0.05) compared to the 2019 reference group.

**Figure 5:**
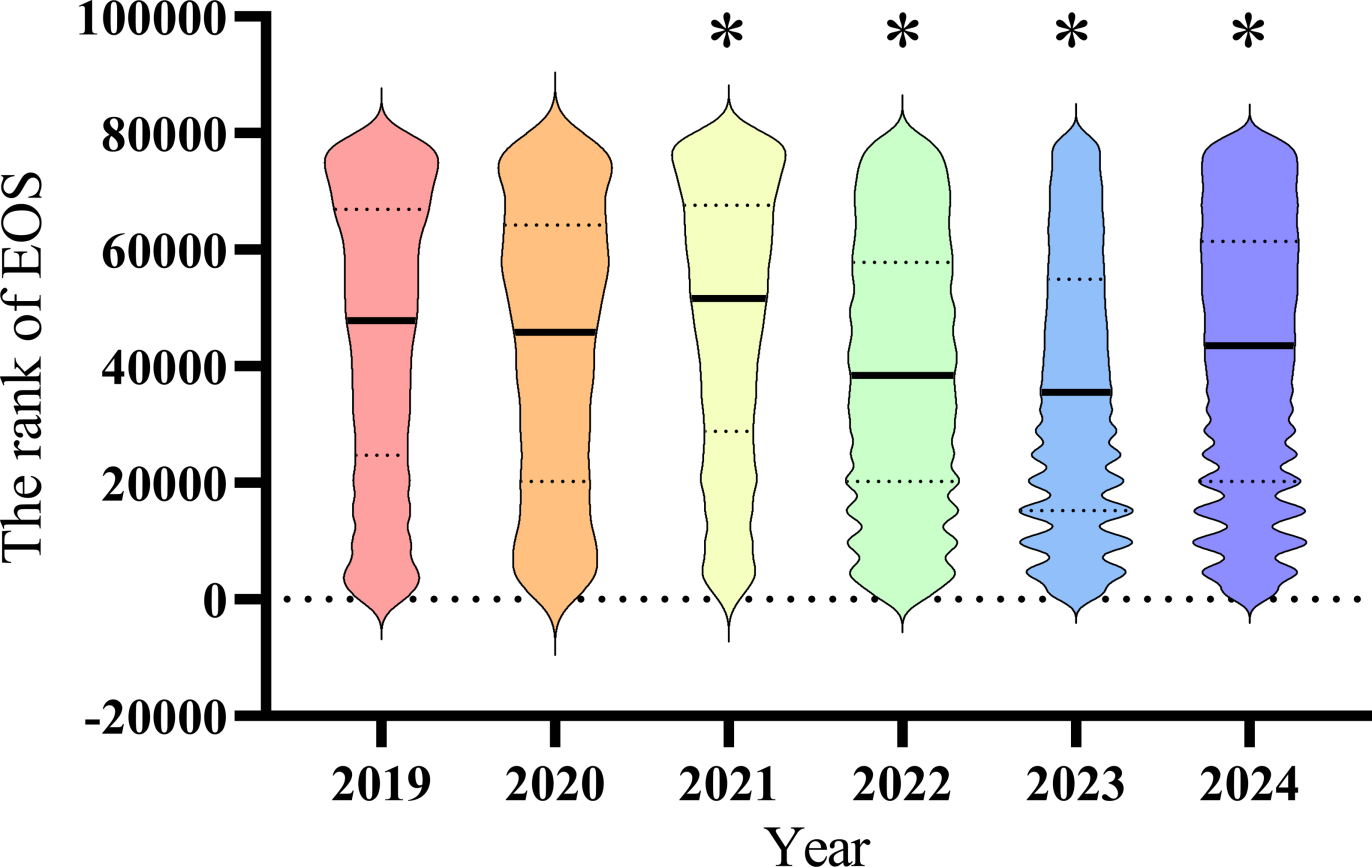
Violin plots with embedded boxplots illustrating the distribution of eosinophils counts rank from 2019 to 2024, analyzed using the Kruskal–Wallis rank-sum test. An asterisk (*) denotes a statistically significant difference (*p* < 0.05) compared to the 2019 reference group.

**Figure 6:**
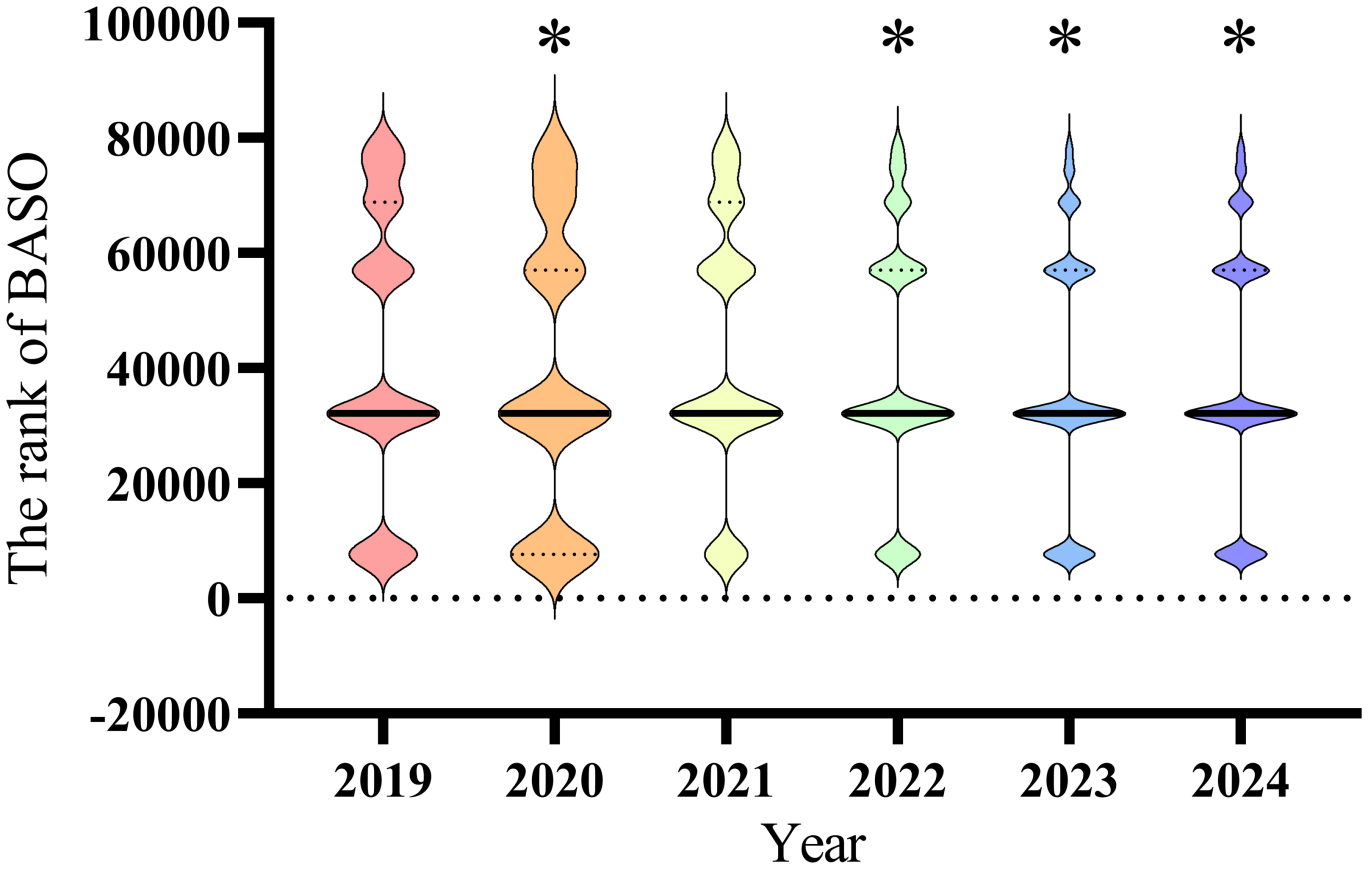
Violin plots with embedded boxplots illustrating the distribution of basophils counts rank from 2019 to 2024, analyzed using the Kruskal–Wallis rank-sum test. An asterisk (*) denotes a statistically significant difference (*p* < 0.05) compared to the 2019 reference group.

Among the patients participating in the analysis of immune cell data, the number of male patients was significantly higher than that of female patients, while the distribution of other data showed little difference. The proportion of age group 1 patients was relatively high in 2019–2020, while the proportion of children in the older age group was relatively high in 2023 and 2024 (Table 1). The rank-order plots of immune cell counts reveal pronounced alterations in the distributional trends across different cell types. The median leukocyte count demonstrated dynamic interannual variation without a clear monotonic trend (Fig. 1, Table 2). A pronounced increasing trend was observed for neutrophil counts over successive years (Fig. 2). In contrast, lymphocyte counts displayed a marked decline from 2019 onward, culminating in a notable rebound in 2024 (Fig. 3). Monocyte counts remained the most stable, showing minimal fluctuation throughout the observation period (Fig. 4). However, analysis using a generalized linear model that included age, sex, diagnosis, and other independent variables as covariates revealed that monocyte counts have been consistently higher since 2021 compared to 2019. Eosinophil counts exhibited a general pattern of decline followed by a partial recovery in 2024 (Fig. 5). A significant change was observed in the central tendency of basophil counts, with the mean rank of the data in 2019 being significantly higher than that of most subsequent years, with the exception of 2021 (Fig. 6).

**Table 1:** Table of patient characteristics involved in immune cell data analysis.

Year	Total	Male	Female	Group=1	Group=2	Group=3	Group=4	URTIs	LRTIs
2019	5,557	3,333 (59.98%)	2,224 (40.02%)	1,483 (26.69%)	3,217 (57.89%)	559 (10.06%)	298 (5.36%)	1,331 (23.95%)	4,226 (76.05%)
2020	1,120	677 (60.45%)	443 (39.55%)	404 (36.07%)	571 (50.98%)	83 (7.41%)	62 (5.54%)	229 (20.45%)	891 (79.55%)
2021	3,402	1,982 (58.26%)	1,420 (41.74%)	318 (9.35%)	1,945 (57.17%)	746 (21.93%)	393 (11.55%)	1,347 (39.59%)	2,055 (60.41%)
2022	7,928	4,492 (56.66%)	3,436 (43.34%)	494 (6.23%)	4,546 (57.34%)	2,157 (27.21%)	731 (9.22%)	3,084 (38.90%)	4,844 (61.10%)
2023	29,390	16,130 (54.88%)	13,259 (45.11%)	945 (3.22%)	12,043 (40.98%)	9,149 (31.13%)	7,253 (24.68%)	12,304 (41.86%)	17,086 (58.14%)
2024	32,316	17,963 (55.59%)	14,353 (44.41%)	851 (2.63%)	15,088 (46.69%)	10,120 (31.32%)	6,257 (19.36%)	12,541 (38.81%)	19,775 (61.19%)

**Table 2:** Table of median immune cell counts and 95% confidence intervals from 2019 to 2024.

Year	White blood cells	Neutrophil	Lymphocyte	Monocyte	Eosinophils	Basophils
2019	8.54 (8.43–8.67)	3.10 (3.03–3.18)	3.93 (3.86–3.98)	0.62 (0.60–0.63)	0.13 (0.12–0.13)	0.01 (0.01–0.01)
2020	8.58 (8.34–8.92)	3.49 (3.26–3.76)	3.63 (3.49–3.80)	0.63 (0.60–0.65)	0.12 (0.11–0.14)	0.01 (0.01–0.01)
2021	9.25 (9.08–9.40)	4.26 (4.11–4.40)	3.35 (3.29–3.44)	0.63 (0.62–0.65)	0.15 (0.14–0.16)	0.01 (0.01–0.01)
2022	8.15 (8.06–8.25)	3.97 (3.89–4.06)	2.83 (2.80–2.88)	0.60 (0.60–0.61)	0.09 (0.09–0.10)	0.01 (0.01–0.01)
2023	8.15 (8.10–8.20)	4.47 (4.43–4.52)	2.42 (2.40–2.44)	0.60 (0.60–0.61)	0.08 (0.07–0.08)	0.01 (0.01–0.01)
2024	8.77 (8.72–8.81)	4.58 (4.54–4.62)	2.79 (2.77–2.81)	0.62 (0.61–0.62)	0.11 (0.11–0.11)	0.01 (0.01–0.01)

All immune cell parameters exhibited significant age-dependent variations. Total white blood cell counts demonstrated an overall decline with advancing age. Lymphocyte and monocyte counts were highest in the youngest age group and progressively decreased with age. Conversely, neutrophil counts increased with age, a pattern consistent with established physiological immune development in children (Supplemental Information S3–S8).

Stratified analysis by age revealed distinct temporal patterns (Table 3). In the youngest age group, neutrophil counts in 2019 were comparable to those in 2020, lower than in 2021, but higher than in the period from 2022 to 2024. This trend underwent a striking reversal in the older age groups (2–4), where 2019 neutrophil counts were significantly lower than in all subsequent years. Moreover, a consistent pattern emerged for lymphocytes, eosinophils, and basophils across all age groups: counts in 2019 were generally higher than in most later years, with the exception of some non-significant differences observed in certain groups during 2020–2021. Analysis of sex-related differences indicated no significant disparity in monocyte or basophil counts between boys and girls in 2019. However, beginning in 2020, statistically significant sex differences emerged for both cell types, with boys exhibiting higher counts than girls. Immune cell counts also displayed differential responses according to clinical diagnosis. For instance, neutrophil counts in children with LRTIs were lower in 2019 compared to other years. Furthermore, a statistically significant divergence in lymphocyte counts emerged between children with upper and lower respiratory tract infections starting in 2020.

**Table 3:** Parameter estimates of significant predictors in the generalized linear model for immune cells concentrations.

Immune cells	Independent variable *a*	Independent variable *b*(I)	Independent variable *b*(J)	Difference in means (I–J)	*p*	95% Wald Confidence Interval for the difference	
						Lower bound	Upper bound
Neutrophil	[Group=1]	2019	2020	0.090	0.667	−0.320	0.500
						2021	−1.887	0.000	−2.404	−1.369
						2022	0.836	0.000	0.542	1.129
						2023	1.139	0.000	0.895	1.383
				2024	1.476	0.000	1.232	1.719
		[Group=2]	2019	2020	0.158	0.173	−0.070	0.386
						2021	−1.018	0.000	−1.183	−0.853
						2022	−0.697	0.000	−0.821	−0.573
						2023	−1.003	0.000	−1.109	−0.897
				2024	−0.891	0.000	−0.994	−0.788
		[Group=3]	2019	2020	−1.703	0.000	−2.632	−0.774
						2021	−1.269	0.000	−1.646	−0.892
						2022	−0.948	0.000	−1.246	−0.651
						2023	−1.114	0.000	−1.380	−0.849
				2024	−1.799	0.000	−2.066	−1.532
		[Group=4]	2019	2020	−1.416	0.012	−2.515	−0.317
						2021	−1.199	0.000	−1.738	−0.660
						2022	−0.510	0.026	−0.958	−0.061
						2023	−0.730	0.000	−1.107	−0.354
				2024	−1.679	0.000	−2.061	−1.297
		LRTIs	2019	2020	−1.045	0.000	−1.409	−0.680
						2021	−1.268	0.000	−1.479	−1.058
						2022	−0.553	0.000	−0.715	−0.391
						2023	−0.604	0.000	−0.739	−0.469
				2024	−0.494	0.000	−0.629	−0.359
		URTIs	2019	2020	−0.144	0.562	−0.631	0.343
						2021	−1.389	0.000	−1.703	−1.076
						2022	−0.032	0.786	−0.261	0.197
						2023	−0.111	0.279	−0.313	0.090
	2024			−0.591	0.000	−0.796	−0.387
Lymphocyte	[Group=1]	2019	2020	0.661	0.000	0.377	0.945
						2021	0.686	0.000	0.423	0.950
						2022	0.242	0.050	0.000	0.484
						2023	0.111	0.286	−0.093	0.314
				2024	−0.268	0.016	−0.487	−0.050
		[Group=2]	2019	2020	0.649	0.000	0.454	0.843
						2021	0.631	0.000	0.509	0.753
						2022	1.096	0.000	0.997	1.196
						2023	1.180	0.000	1.089	1.270
				2024	1.001	0.000	0.911	1.091
		[Group=3]	2019	2020	0.545	0.001	0.214	0.876
						2021	0.051	0.582	−0.131	0.233
						2022	0.797	0.000	0.649	0.946
						2023	1.095	0.000	0.955	1.236
				2024	0.730	0.000	0.590	0.871
		[Group=4]	2019	2020	0.269	0.132	−0.081	0.619
						2021	−0.120	0.269	−0.332	0.093
						2022	0.497	0.000	0.320	0.674
						2023	0.712	0.000	0.554	0.871
	2024			0.403	0.000	0.243	0.563
Monocyte	2019	Male	Female	0.012	0.178	−0.005	0.029
		2020	Male	Female	0.021	0.282	−0.017	0.058
		2021	Male	Female	0.036	0.004	0.011	0.060
		2022	Male	Female	0.045	0.000	0.029	0.060
		2023	Male	Female	0.038	0.000	0.029	0.047
	2024	Male	Female	0.036	0.000	0.026	0.045
Eosinophils	[Group=1]	2019	2020	0.121	0.000	0.087	0.155
						2021	0.127	0.000	0.095	0.159
						2022	0.079	0.000	0.048	0.110
						2023	0.071	0.000	0.043	0.098
				2024	0.010	0.531	−0.021	0.040
		[Group=2]	2019	2020	0.026	0.000	0.014	0.038
						2021	−0.032	0.000	−0.042	−0.022
						2022	0.026	0.000	0.020	0.033
						2023	0.021	0.000	0.015	0.027
				2024	0.010	0.002	0.004	0.016
		[Group=3]	2019	2020	0.004	0.815	−0.032	0.040
						2021	−0.051	0.000	−0.071	−0.030
						2022	0.037	0.000	0.022	0.052
						2023	0.056	0.000	0.042	0.070
				2024	0.015	0.034	0.001	0.030
		[Group=4]	2019	2020	−0.041	0.123	−0.094	0.011
						2021	−0.090	0.000	−0.119	−0.060
						2022	0.014	0.195	−0.007	0.035
						2023	0.043	0.000	0.024	0.062
	2024			−0.006	0.559	−0.025	0.013
Basophils	2019	Male	Female	0.000	0.444	−0.001	0.001
		2020	Male	Female	0.003	0.004	0.001	0.005
		2021	Male	Female	0.003	0.000	0.002	0.004
		2022	Male	Female	0.002	0.000	0.001	0.002
		2023	Male	Female	0.002	0.000	0.002	0.003
		2024	Male	Female	0.002	0.000	0.001	0.002
		[Group=1]	2019	2020	0.004	0.007	0.001	0.006
						2021	0.009	0.000	0.007	0.011
						2022	0.009	0.000	0.007	0.010
						2023	0.010	0.000	0.009	0.012
				2024	0.011	0.000	0.009	0.012
		[Group=2]	2019	2020	0.007	0.000	0.005	0.008
						2021	0.004	0.000	0.003	0.005
						2022	0.006	0.000	0.005	0.007
						2023	0.008	0.000	0.007	0.008
				2024	0.008	0.000	0.007	0.008
		[Group=3]	2019	2020	0.002	0.306	−0.002	0.005
						2021	0.001	0.326	−0.001	0.003
						2022	0.003	0.000	0.001	0.004
						2023	0.005	0.000	0.004	0.007
				2024	0.004	0.000	0.003	0.006
		[Group=4]	2019	2020	0.004	0.050	0.000	0.008
						2021	−0.001	0.720	−0.003	0.002
						2022	0.003	0.005	0.001	0.005
						2023	0.006	0.000	0.004	0.008
	2024			0.006	0.000	0.004	0.008

### Immunoglobulins

**Figure 7:**
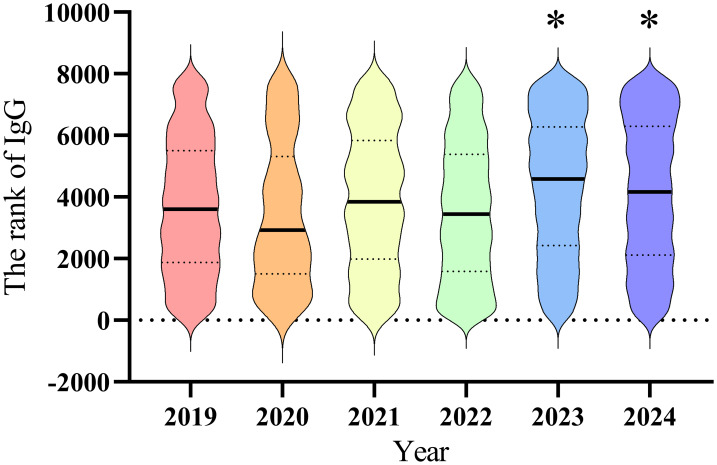
Violin plots with embedded boxplots showing the distribution of IgG concentrations rank from 2019 to 2024, analyzed using the Kruskal–Wallis rank-sum test. An asterisk (*) denotes a statistically significant difference (*p* < 0.05) compared to the 2019 reference group.

**Figure 8:**
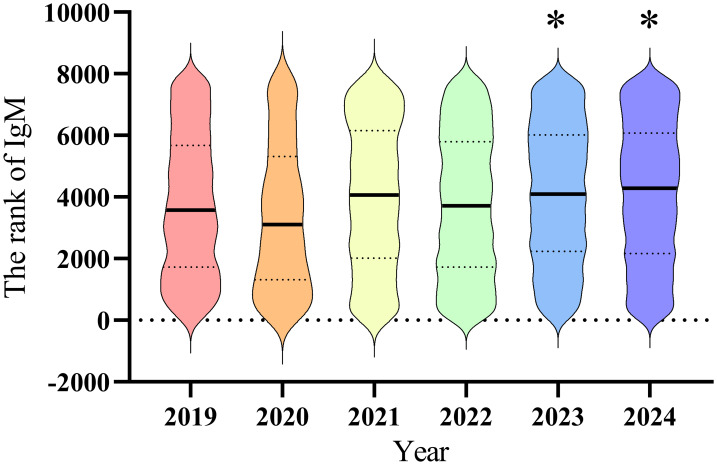
Violin plots with embedded boxplots showing the distribution of IgM concentrations rank from 2019 to 2024, analyzed using the Kruskal–Wallis rank-sum test. An asterisk (*) denotes a statistically significant difference (*p* < 0.05) compared to the 2019 reference group.

**Figure 9:**
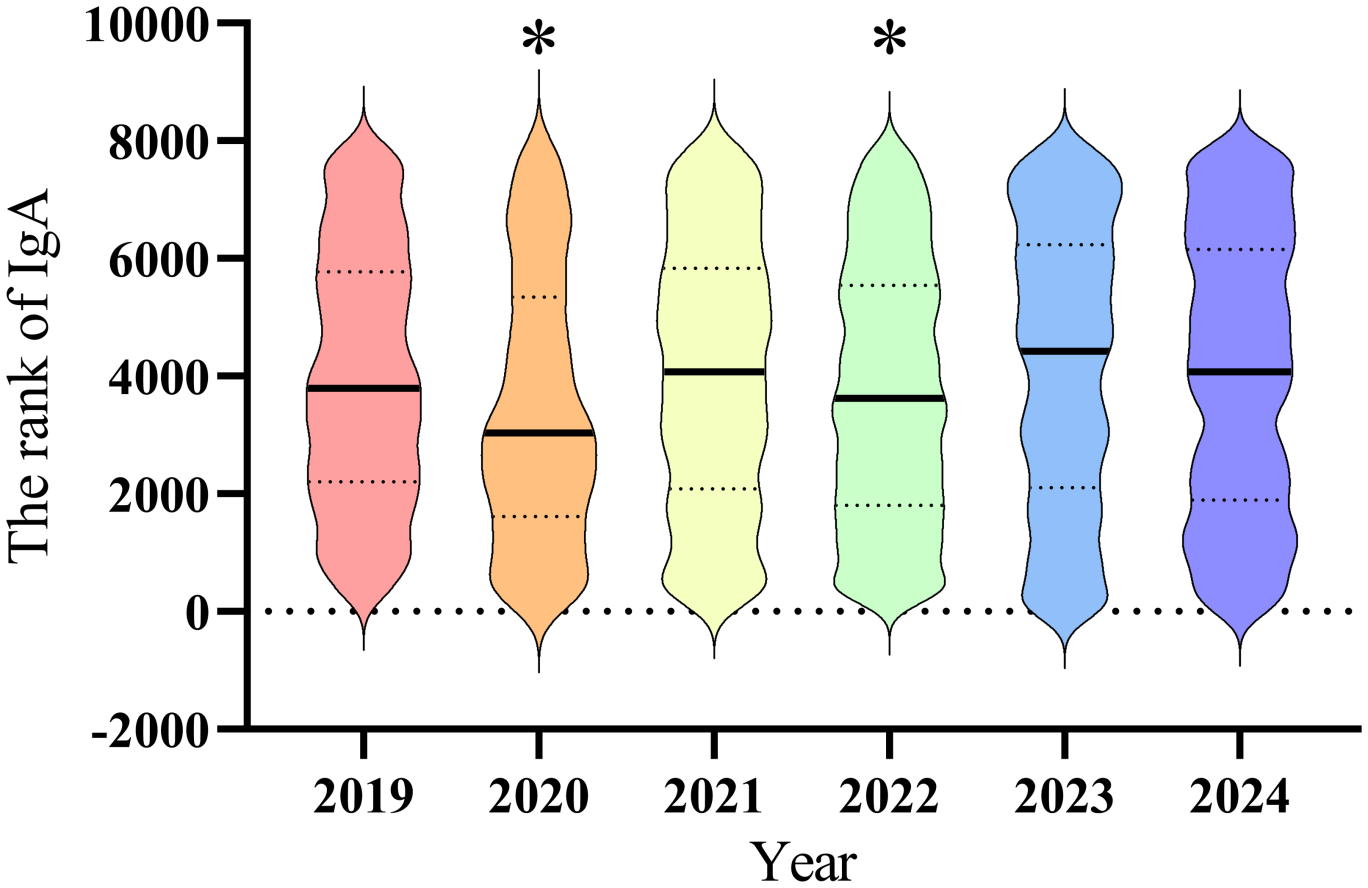
Violin plots with embedded boxplots showing the distribution of IgA concentrations rank from 2019 to 2024, analyzed using the Kruskal–Wallis rank-sum test. An asterisk (*) denotes a statistically significant difference (*p* < 0.05) compared to the 2019 reference group.

Among the patients participating in the immunoglobulin data analysis, the number of male patients was significantly higher than that of female patients, while the distribution of the remaining data showed little difference (Table 4). Rank-order plots and median immunoglobulin levels revealed discernible temporal trends over the study period (Figs. 7–9, Table 5). The distribution trends of both IgM and IgG changed significantly in 2023 and 2024 (Figs. 7 & 8). As shown in Fig. 9, the distribution trend of IgA in 2019 was significantly different from those in 2020 and 2022.

**Table 4:** Table of patient characteristics involved in immunoglobulins data analysis.

Year	Total	Male	Female	Group=1	Group=2	Group=3	Group=4	URTIs	LRTIs
2019	1,012	598 (59.09%)	414 (40.91%)	330 (32.61%)	434 (42.89%)	158 (15.61%)	90 (8.89%)	341 (33.70%)	671 (66.30%)
2020	240	143 (59.58%)	97 (40.42%)	99 (41.25%)	96 (40.00%)	18 (7.50%)	27 (11.25%)	90 (37.50%)	150 (62.50%)
2021	634	401 (63.25%)	233 (36.75%)	203 (32.02%)	255 (40.22%)	120 (18.93%)	56 (8.83%)	189 (29.81%)	445 (70.19%)
2022	1,820	1,066 (58.57%)	754 (41.43%)	641 (35.22%)	780 (42.86%)	318 (17.47%)	81 (4.45%)	415 (22.80%)	1,405 (77.20%)
2023	1,994	1,194 (59.88%)	800 (40.12%)	487 (24.42%)	764 (38.31%)	425 (21.31%)	318 (15.95%)	564 (28.28%)	1,430 (71.72%)
2024	2,231	1,311 (58.76%)	920 (41.24%)	714 (32.00%)	787 (35.28%)	436 (19.54%)	294 (13.18%)	464 (20.80%)	1,767 (79.20%)

**Table 5:** Table of median immune molecule levels and 95% confidence intervals from 2019 to 2024.

Year	IgG	IgA	IgM
2019	6.32 (6.09–6.51)	0.45 (0.43–0.48)	0.88 (0.84–0.91)
2020	5.71 (5.31–6.26)	0.34 (0.29–0.40)	0.82 (0.74–0.89)
2021	6.53 (6.29–6.91)	0.49 (0.45–0.54)	0.96 (0.90–1.00)
2022	6.18 (5.98–6.34)	0.43 (0.41–0.44)	0.90 (0.87–0.93)
2023	7.19 (7.01–7.32)	0.56 (0.51–0.59)	0.96 (0.94–1.00)
2024	6.79 (6.68–6.99)	0.49 (0.48–0.52)	0.99 (0.96–1.01)

All immunoglobulin parameters demonstrated a significant age-dependent increase, with concentrations generally rising in older children (Supplemental Information S9–S11). Within younger age groups, IgA levels in 2019 were slightly higher than those observed from 2020 to 2024. An inverse pattern was noted for IgM, where concentrations in 2019 were comparatively higher than in subsequent years among older children (Table 6). Sex-related differences were also evident. Male children with lower respiratory tract infections exhibited significantly lower IgG levels than females. Similarly, IgM concentrations were consistently lower in males across age groups, whereas IgA levels were higher in males compared to females (Tables S9–S11).

**Table 6:** Parameter estimates of significant predictors in the generalized linear model for immunoglobulins concentrations.

Immunoglobulin	Independent variable *a*	Independent variable *b*(I)	Independent variable *b*(J)	Difference in means (I–J)	*p*	95% Wald Confidence Interval for the difference	
						Lower bound	Upper bound
IgA	[Group=1]	2019	2020	0.046	0.003	0.015	0.077
						2021	0.034	0.010	0.008	0.060
						2022	0.028	0.009	0.007	0.048
						2023	0.058	0.000	0.038	0.077
				2024	0.041	0.000	0.021	0.060
		[Group=2]	2019	2020	0.065	0.098	−0.012	0.141
						2021	−0.036	0.257	−0.099	0.026
						2022	0.006	0.795	−0.040	0.052
						2023	0.070	0.002	0.027	0.114
				2024	0.061	0.007	0.017	0.105
		[Group=3]	2019	2020	−0.014	0.941	−0.376	0.348
						2021	−0.046	0.613	−0.225	0.133
						2022	0.049	0.487	−0.089	0.188
						2023	0.030	0.660	−0.103	0.163
				2024	0.015	0.822	−0.119	0.150
		[Group=4]	2019	2020	0.130	0.565	−0.313	0.574
						2021	0.168	0.335	−0.174	0.509
						2022	0.064	0.697	−0.256	0.383
						2023	0.141	0.274	−0.112	0.393
	2024			−0.042	0.754	−0.305	0.221
IgM	[Group=1]	2019	2020	0.035	0.295	−0.030	0.099
						2021	0.003	0.923	−0.050	0.055
						2022	0.018	0.375	−0.022	0.058
						2023	0.002	0.934	−0.039	0.043
				2024	−0.020	0.317	−0.060	0.019
		[Group=2]	2019	2020	−0.005	0.928	−0.109	0.099
						2021	−0.077	0.053	−0.154	0.001
						2022	−0.023	0.428	−0.080	0.034
						2023	0.010	0.728	−0.046	0.066
				2024	−0.027	0.362	−0.084	0.031
		[Group=3]	2019	2020	−0.119	0.400	−0.396	0.158
						2021	−0.112	0.097	−0.243	0.020
						2022	−0.039	0.457	−0.140	0.063
						2023	−0.017	0.726	−0.114	0.079
				2024	−0.076	0.129	−0.175	0.022
		[Group=4]	2019	2020	0.051	0.725	−0.232	0.333
						2021	0.218	0.038	0.013	0.422
						2022	0.089	0.369	−0.106	0.284
						2023	0.234	0.003	0.081	0.387
	2024			0.218	0.006	0.063	0.374

## Discussion

The aforementioned data indicate that the COVID-19 pandemic exerted a profound yet heterogeneous impact on the development of the pediatric immune system. Due to differences in the developmental processes and regulatory factors of various immune cells and immunoglobulins, the effects observed were highly variable. The pandemic appeared to inversely affect lymphocytes, eosinophils, and basophils, suppressing their numerical increase, with a particularly pronounced inhibitory effect on lymphocytes. Studies have shown that lymphocytopenia accompanied by neutrophilia is a hallmark of severe COVID-19. Pediatric cases generally present with milder clinical manifestations. Lymphocyte reduction is less commonly observed in children infected with SARS-CoV-2, with some even exhibiting increased lymphocyte counts (Ma et al., 2021; McKenna et al., 2022). Early in life, children primarily rely on innate lymphoid cells (ILCs) for immune defense. The number of ILCs gradually declines with age. These cells play a crucial role in protecting children against SARS-CoV-2 and other pathogens but may undergo significant depletion during infection (Silverstein et al., 2022). In contrast, neutrophil and counts were promoted. In this study, the contrasting changes in neutrophil counts among children of different age groups before and after the COVID-19 pandemic may reflect a compensatory replenishment mechanism of the immune system. These observations suggest that the relatively delayed development and maturation of the adaptive immune system in children might lead to compensatory proliferation of innate immunity upon respiratory pathogen infection, thereby counterbalancing the temporary insufficiency of adaptive immune responses. The differential impact was also reflected in the distinct responses of various immune cells to infections in different parts of the respiratory tract. Neutrophils exhibited increased variability in response to lower respiratory tract infections, whereas lymphocytes demonstrated a weakened response to upper respiratory tract infections. Significant sex-based differences were observed in monocyte and basophil counts, indicating that the pandemic may have differentially affected immune development in boys and girls. Lymphocyte and eosinophil counts showed a rebound trend by 2024, indicating that the impact of the COVID-19 pandemic is weakening or disappearing.

Compared to 2019, no significant differences were observed in children’s immunoglobulin levels between 2020 and 2022. Studies have indicated no statistically significant difference in antibody levels against common coronaviruses and SARS-CoV-2 between children and adults (Pierce et al., 2020). However, a marked increase in serum concentrations of IgG and IgM was noted from 2023 to 2024, accompanied by a rebound in lymphocyte counts by 2024. These shifts suggest that the relaxation of COVID-19 containment measures may have led to a rapid rise in children’s exposure to pathogens in their environment, potentially stimulating lymphocyte development and enhancing immunoglobulin production.

The development of the immune system in children is a protracted and intricate dynamic process. During the neonatal period, the immune system is not yet fully mature, and immune defense primarily relies on maternally derived antibodies, which help protect infants from pathogen infections in early life (Pieren, Boer & De Wit, 2022). As children grow, innate immunity initially assumes the major role in immune defense: although not fully mature, neutrophils and monocytes already possess certain pathogen-clearing capabilities (Kloc et al., 2020). Subsequently, the adaptive immune system gradually develops and matures, shifting the protective mechanisms from immediate defense toward long-term immunological memory. There exists a highly co-evolutionary relationship between pathogens and the human immune system, a process that profoundly shapes both the composition and functionality of immune defenses. The immune system gradually builds a memory repertoire and produces specific antibodies through recognition of pathogen-derived antigens (Hooper, Littman & Macpherson, 2012; Cristiani et al., 2020). Concurrently, continuous interaction between the immune system and pathogens leads to a gradual down-regulation of immune response intensity, which helps reduce excessive immune activation such as hypersensitivity reactions. While adaptive immunity plays a dominant role, innate immunity not only assists in defense but also fulfills functions such as antigen presentation and immune regulation. The two systems operate synergistically; when adaptive immunity is compromised, innate immunity may exhibit reactive enhancement (Pieren, Boer & De Wit, 2022). COVID-19 infection can lead to multi-organ damage, including in the immune, respiratory, and digestive systems. The primary mechanism involves SARS-CoV-2 binding to the ACE2 receptor and entering host cells *via* transmembrane serine protease 2 (TMPRSS2)-mediated facilitation for replication. Intracellular virus can be detected by pattern recognition receptors (PRRs), including Toll-like receptors 3 and 7 (TLR3, TLR7). TLR3 signaling can induce NLRP3 inflammasome activation, promote the release of pro-inflammatory cytokines such as IL-1*β* and IL-18, and trigger pyroptosis (Li et al., 2021). Studies have shown that COVID-19 patients exhibit a significant reduction in T cells, which correlates negatively with serum concentrations of IL-6, IL-10, and TNF-*α*. Concurrent upregulation of PD-1 expression suggests T cell exhaustion (Diao et al., 2020). Furthermore, the presence of abundant autoantibodies and autoimmune activation post-infection may cause immune cell damage and suppress normal immune protein development, substantially impacting immune maturation (Vlachoyiannopoulos et al., 2020). Due to lower expression of cell surface receptors such as ACE2 and the still-maturing immune system in children, the aforementioned autoimmune injury mechanisms are less likely to occur in this population. This may partly explain the lower incidence and clinical severity of COVID-19 infections among children (Howard-Jones et al., 2022). Our data indicate that during acute infection, the adaptive immune response is relatively insufficient, accompanied by rapid depletion of lymphocytes, while neutrophils may increase compensatorily to offset the functional deficit of lymphocytes. An elevation in neutrophils—representing innate immunity—alongside a reduction in lymphocytes—representing adaptive immunity—often reflects a shift in immune balance toward acute inflammation or a stress state. This phenomenon differs fundamentally from the typical homeostatic “compensation” observed in a mature immune system. For instance, the innate immune system in children often responds rapidly and robustly upon SARS-CoV-2 infection, which helps compensate to some extent for the functional limitations of their adaptive immunity (Vono et al., 2021). However, excessive activation of innate immunity may trigger a severe systemic inflammatory response, thereby disrupting normal immune developmental rhythms and leading to immune cell dysfunction and impaired formation of immunological memory (Isaza-Correa et al., 2023). Although previous studies have demonstrated sex differences in the development of the immune system in children, the data from this study further reveal that in 2019 (prior to the COVID-19 pandemic), there were no significant differences in the levels of immune cells and immune molecules between male and female children (Kobor et al., 2024). However, since the outbreak of the pandemic in 2020, these parameters have begun to exhibit pronounced sexual dimorphism. This phenomenon suggests that SARS-CoV-2 infection may have acted as a “magnifying glass”, exposing or exacerbating underlying sex differences in the pediatric immune system, thereby rendering previously indistinct immune characteristics clearly discernible in the post-pandemic era.

The COVID-19 pandemic and China’s corresponding containment policies created a unique natural experiment characterized by prolonged home confinement and markedly reduced pathogen exposure among children. As the impact of COVID-19 itself on the developing immune system remained poorly understood, this study seized a critical opportunity to systematically evaluate the effects of these combined factors using a large clinical dataset from the pediatric population. Our findings provide objective and reliable data that significantly advance the field. They offer a solid empirical foundation for basic research on immune development in children, shedding light on intrinsic regulatory patterns and underlying mechanisms. Within the realm of viral pathogenesis, this work contributes novel insights into the interaction between COVID-19 and the pediatric immune system. From a clinical perspective, the results support the development of targeted therapeutic strategies for respiratory infections in children. At the public health level, this study offers an evidence-based framework to inform future responses to similar large-scale health crises, thereby enhancing the scientific rigor and preparedness of public health decision-making. Furthermore, these findings have meaningful implications for vaccination policy. They provide a theoretical basis for refining current immunization schedules and exploring more effective timing and sequences of vaccine administration, ultimately supporting child health and strengthening population-wide immunity.

The large sample size in this study increases the robustness of the statistical findings. Nevertheless, it is important to critically consider the following limitations. Firstly, the analysis was restricted to quantitative measurements of immune cells and immunoglobulins, without further investigation into functional subtypes of immune cells or molecular profiles. As a result, the deeper mechanistic effects of the COVID-19 pandemic on the developing immune system in children remain unexplored. The generalizability of our conclusions is primarily limited by the single-center design and the exclusive focus on a pediatric population with respiratory infections. Furthermore, the lack of a matched healthy control group prevents direct comparison, which may affect the theoretical completeness of the immunological framework proposed. This study only reported statistical changes in immune parameters, including neutrophils, lymphocytes, and basophils, but lacked relevant data on patient clinical manifestations. Previous studies have indicated that children with reduced lymphocyte counts are more likely to develop severe illness, characterized by increased disease severity and elevated mortality (Airlangga et al., 2024). The observed reduction in specimen numbers during 2020 is likely attributable to the implementation of strict pandemic control policies, which consequently reduced the volume of non-urgent patient visits to healthcare facilities for examinations. This may cause some deviation in the statistical results. Critical aspects such as autoimmunity and intestinal immunity were not assessed, limiting the comprehensiveness of our immunological interpretation. Finally, potential confounding factors—including general growth indicators, vaccination status, school attendance, the severity of the infections, chronic conditions, immunosuppression—were not incorporated into the analysis. Their absence may influence the interpretation of the observed data and merits consideration in future studies.

## Conclusion

During the COVID-19 pandemic, an increase in neutrophils and monocytes alongside a decrease in lymphocytes was observed in children, suggesting that an enhanced innate immune response may partially compensate for the relative insufficiency of adaptive immune function. Following the relaxation of containment measures in late 2022, although children were widely exposed to SARS-CoV-2 infection, no significant decline in lymphocytes was noted. In contrast, lymphocyte counts rebounded by 2024, accompanied by a marked elevation in immunoglobulin levels. These changes are likely attributable to the substantial increase in pathogen exposure and sustained immune stimulation in children after the lifting of restrictions. Collectively, these findings support the view that prolonged reduction in pathogen contact may delay the normal development of the immune system in children. This study provides important time-series data on immune development in children and offers new evidence for understanding the interaction between COVID-19 and the pediatric immune system. The results not only contribute to elucidating the response patterns of children’s immune systems during major public health events but may also inform the development of targeted child health policies, optimization of vaccination strategies, and improvements in clinical management. To further validate these findings and elucidate the underlying mechanisms, future research should prioritize longitudinal cohort studies tracking immune development from early life through adolescence, alongside functional immunological analyses—including assessments of T-cell responses, cytokine production profiles, and B-cell differentiation—to better characterize the long-term impacts of pandemic-related pathogen containment on immune system maturation.

##  Supplemental Information

10.7717/peerj.21469/supp-1 Supplemental Information 1 2019–2025 Blood cell analysis data.

10.7717/peerj.21469/supp-2 Supplemental Information 2 2019–2025 Immune molecular data.

10.7717/peerj.21469/supp-3 Supplemental Information 3 White blood cell Generalized Linear Model.

10.7717/peerj.21469/supp-4 Supplemental Information 4 Neutrophils Generalized Linear Model.

10.7717/peerj.21469/supp-5 Supplemental Information 5 Lymphocyte Generalized Linear Model.

10.7717/peerj.21469/supp-6 Supplemental Information 6 Monocyte cell Generalized Linear Model.

10.7717/peerj.21469/supp-7 Supplemental Information 7 Eosinophils Generalized Linear Model.

10.7717/peerj.21469/supp-8 Supplemental Information 8 Basophils Generalized Linear Model.

10.7717/peerj.21469/supp-9 Supplemental Information 9 Immunoglobulin A Generalized Linear Model.

10.7717/peerj.21469/supp-10 Supplemental Information 10 Immunoglobulin M Generalized Linear Model.

10.7717/peerj.21469/supp-11 Supplemental Information 11 Immunoglobulin G Generalized Linear Model.

10.7717/peerj.21469/supp-12 Supplemental Information 12 STROBE Checklist.

## Additional Information and Declarations

### Data Availability

The following information was supplied regarding data availability: The data is available in the Supplemental Files.
