# Supplementary material for: The impact of COVID-19 social isolation and reduced microbial exposure on the immune system in children: a retrospective study
Source: PeerJ. 2026 Jul 7;14:e21469. doi: 10.7717/peerj.21469 (PMC13353229; doi:10.7717/peerj.21469)
Supplement: Supplemental Information 3 [file peerj-14-21469-s003.docx]

**White blood cell Generalized Linear Model**

For CBC analysis, patients were categorized into the following age groups:
Group 1: 0–3 months,
Group 2: 3 months–4 years,
Group 3: 4–6 years,
Group 4: 6–13 years.

Diagnostic 1 is LRTIs . Diagnostic 2 URTIs .

| **Parameter Estimate** | | | | | | | |
| --- | --- | --- | --- | --- | --- | --- | --- |
| Parameter | B | Standard Error | 95% Wald Confidence Interval | | Hypothesis Testing | | |
|  |  |  | Lower Bound | Upper Bound | Wald χ² | Degrees of Freedom | P |
| （Intercept） | 2.213 | .0274 | 2.160 | 2.267 | 6546.235 | 1 | .000 |
| [Year=2020] | .051 | .0648 | -.076 | .178 | .627 | 1 | .429 |
| [Year=2021] | .152 | .0362 | .081 | .222 | 17.540 | 1 | .000 |
| [Year=2022] | -.041 | .0318 | -.103 | .022 | 1.627 | 1 | .202 |
| [Year=2023] | -.045 | .0274 | -.098 | .009 | 2.645 | 1 | .104 |
| [Year=2024] | .147 | .0274 | .093 | .200 | 28.564 | 1 | .000 |
| [Year=2025] | 0 | . | . | . | . | . | . |
| [Male ] | .005 | .0149 | -.024 | .034 | .118 | 1 | .731 |
| [Female ] | 0 | . | . | . | . | . | . |
| [Age=1] | .113 | .0351 | .044 | .182 | 10.381 | 1 | .001 |
| [Age=2] | .026 | .0267 | -.027 | .078 | .933 | 1 | .334 |
| [Age=3] | .041 | .0314 | -.021 | .102 | 1.704 | 1 | .192 |
| [Age=4] | 0 | . | . | . | . | . | . |
| [LRTIs ] | -.136 | .0164 | -.168 | -.103 | 68.303 | 1 | .000 |
| [URTIs ] | 0 | . | . | . | . | . | . |
| [Year=2020] * [Male ] | .002 | .0285 | -.054 | .058 | .004 | 1 | .949 |
| [Year=2020] * [Female ] | 0 | . | . | . | . | . | . |
| [Year=2021] * [Male ] | .033 | .0190 | -.005 | .070 | 2.965 | 1 | .085 |
| [Year=2021] * [Female ] | 0 | . | . | . | . | . | . |
| [Year=2022] * [Male ] | .036 | .0154 | .006 | .066 | 5.393 | 1 | .020 |
| [Year=2022] * [Female ] | 0 | . | . | . | . | . | . |
| [Year=2023] * [Male ] | .031 | .0133 | .005 | .057 | 5.335 | 1 | .021 |
| [Year=2023] * [Female ] | 0 | . | . | . | . | . | . |
| [Year=2024] * [Male ] | .025 | .0132 | -.001 | .051 | 3.601 | 1 | .058 |
| [Year=2024] * [Female ] | 0 | . | . | . | . | . | . |
| [Year=2025] * [Male ] | 0 | . | . | . | . | . | . |
| [Year=2025] * [Female ] | 0 | . | . | . | . | . | . |
| [Year=2020] * [Age=1] | -.220 | .0672 | -.351 | -.088 | 10.697 | 1 | .001 |
| [Year=2020] * [Age=2] | -.231 | .0634 | -.355 | -.106 | 13.241 | 1 | .000 |
| [Year=2020] * [Age=3] | -.013 | .0782 | -.166 | .140 | .027 | 1 | .869 |
| [Year=2020] * [Age=4] | 0 | . | . | . | . | . | . |
| [Year=2021] * [Age=1] | -.050 | .0427 | -.134 | .033 | 1.389 | 1 | .239 |
| [Year=2021] * [Age=2] | -.124 | .0349 | -.192 | -.055 | 12.535 | 1 | .000 |
| [Year=2021] * [Age=3] | -.031 | .0404 | -.110 | .048 | .585 | 1 | .444 |
| [Year=2021] * [Age=4] | 0 | . | . | . | . | . | . |
| [Year=2022] * [Age=1] | -.119 | .0374 | -.193 | -.046 | 10.215 | 1 | .001 |
| [Year=2022] * [Age=2] | -.055 | .0309 | -.116 | .005 | 3.202 | 1 | .074 |
| [Year=2022] * [Age=3] | .010 | .0355 | -.060 | .079 | .075 | 1 | .784 |
| [Year=2022] * [Age=4] | 0 | . | . | . | . | . | . |
| [Year=2023] * [Age=1] | -.143 | .0316 | -.205 | -.081 | 20.462 | 1 | .000 |
| [Year=2023] * [Age=2] | -.026 | .0266 | -.078 | .026 | .945 | 1 | .331 |
| [Year=2023] * [Age=3] | -.008 | .0312 | -.069 | .053 | .064 | 1 | .800 |
| [Year=2023] * [Age=4] | 0 | . | . | . | . | . | . |
| [Year=2024] * [Age=1] | -.273 | .0320 | -.336 | -.210 | 72.820 | 1 | .000 |
| [Year=2024] * [Age=2] | -.161 | .0266 | -.213 | -.109 | 36.665 | 1 | .000 |
| [Year=2024] * [Age=3] | -.032 | .0313 | -.093 | .030 | 1.017 | 1 | .313 |
| [Year=2024] * [Age=4] | 0 | . | . | . | . | . | . |
| [Year=2025] * [Age=1] | 0 | . | . | . | . | . | . |
| [Year=2025] * [Age=2] | 0 | . | . | . | . | . | . |
| [Year=2025] * [Age=3] | 0 | . | . | . | . | . | . |
| [Year=2025] * [Age=4] | 0 | . | . | . | . | . | . |
| [Year=2020] * [LRTIs ] | .168 | .0369 | .096 | .241 | 20.730 | 1 | .000 |
| [Year=2020] * [URTIs ] | 0 | . | . | . | . | . | . |
| [Year=2021] * [LRTIs ] | .033 | .0208 | -.008 | .074 | 2.547 | 1 | .111 |
| [Year=2021] * [URTIs ] | 0 | . | . | . | . | . | . |
| [Year=2022] * [LRTIs ] | .079 | .0172 | .046 | .113 | 21.208 | 1 | .000 |
| [Year=2022] * [URTIs ] | 0 | . | . | . | . | . | . |
| [Year=2023] * [LRTIs ] | .092 | .0151 | .063 | .122 | 37.337 | 1 | .000 |
| [Year=2023] * [URTIs ] | 0 | . | . | . | . | . | . |
| [Year=2024] * [LRTIs ] | .006 | .0150 | -.023 | .036 | .173 | 1 | .678 |
| [Year=2024] * [URTIs ] | 0 | . | . | . | . | . | . |
| [Year=2025] * [LRTIs ] | 0 | . | . | . | . | . | . |
| [Year=2025] * [URTIs ] | 0 | . | . | . | . | . | . |
| [Male ] * [Age=1] | -.096 | .0156 | -.126 | -.065 | 37.910 | 1 | .000 |
| [Male ] * [Age=2] | -.029 | .0084 | -.046 | -.013 | 12.068 | 1 | .001 |
| [Male ] * [Age=3] | -.008 | .0090 | -.025 | .010 | .761 | 1 | .383 |
| [Male ] * [Age=4] | 0 | . | . | . | . | . | . |
| [Female ] * [Age=1] | 0 | . | . | . | . | . | . |
| [Female ] * [Age=2] | 0 | . | . | . | . | . | . |
| [Female ] * [Age=3] | 0 | . | . | . | . | . | . |
| [Female ] * [Age=4] | 0 | . | . | . | . | . | . |
| [Male ] * [LRTIs ] | -.017 | .0063 | -.029 | -.004 | 7.015 | 1 | .008 |
| [Male ] * [URTIs ] | 0 | . | . | . | . | . | . |
| [Female ] * [LRTIs ] | 0 | . | . | . | . | . | . |
| [Female ] * [URTIs ] | 0 | . | . | . | . | . | . |
| [Age=1] * [LRTIs ] | .295 | .0216 | .252 | .337 | 186.098 | 1 | .000 |
| [Age=1] * [URTIs ] | 0 | . | . | . | . | . | . |
| [Age=2] * [LRTIs ] | .120 | .0085 | .104 | .137 | 200.968 | 1 | .000 |
| [Age=2] * [URTIs ] | 0 | . | . | . | . | . | . |
| [Age=3] * [LRTIs ] | .006 | .0092 | -.012 | .024 | .448 | 1 | .503 |
| [Age=3] * [URTIs ] | 0 | . | . | . | . | . | . |
| [Age=4] * [LRTIs ] | 0 | . | . | . | . | . | . |
| [Age=4] * [URTIs ] | 0 | . | . | . | . | . | . |
| （标度） | .180 | .0009 | .179 | .182 |  |  |  |

**Estimated Marginal Means 1：Year**

| **Estimate** | | | | |
| --- | --- | --- | --- | --- |
| Year | Mean | Standard Error | 95% Wald Confidence Interval | |
|  |  |  | Lower Bound | Upper Bound |
| 2020 | 9.4455 | .19551 | 9.0700 | 9.8365 |
| 2021 | 10.5732 | .10418 | 10.3710 | 10.7794 |
| 2022 | 9.0332 | .06444 | 8.9078 | 9.1604 |
| 2023 | 9.0061 | .03877 | 8.9305 | 9.0824 |
| 2024 | 9.6879 | .04272 | 9.6046 | 9.7720 |
| 2025 | 9.2541 | .08496 | 9.0891 | 9.4222 |

| **Pairwise Comparisons** | | | | | | | |
| --- | --- | --- | --- | --- | --- | --- | --- |
| (I) Year | (J) Year | Mean Difference (I-J) | Standard Error | Degrees of Freedom | P | 95% Wald Confidence Interval | |
|  |  |  |  |  |  | Lower Bound | Upper Bound |
| 2020 | 2021 | -1.1278 | .21934 | 1 | .000 | -1.5577 | -.6979 |
|  | 2022 | .4123 | .20438 | 1 | .044 | .0117 | .8129 |
|  | 2023 | .4394 | .19788 | 1 | .026 | .0515 | .8272 |
|  | 2024 | -.2425 | .19876 | 1 | .223 | -.6320 | .1471 |
|  | 2025 | .1913 | .21090 | 1 | .364 | -.2220 | .6047 |
| 2021 | 2020 | 1.1278 | .21934 | 1 | .000 | .6979 | 1.5577 |
|  | 2022 | 1.5401 | .11995 | 1 | .000 | 1.3050 | 1.7752 |
|  | 2023 | 1.5671 | .10847 | 1 | .000 | 1.3545 | 1.7797 |
|  | 2024 | .8853 | .11005 | 1 | .000 | .6696 | 1.1010 |
|  | 2025 | 1.3191 | .13088 | 1 | .000 | 1.0626 | 1.5756 |
| 2022 | 2020 | -.4123 | .20438 | 1 | .044 | -.8129 | -.0117 |
|  | 2021 | -1.5401 | .11995 | 1 | .000 | -1.7752 | -1.3050 |
|  | 2023 | .0271 | .07255 | 1 | .709 | -.1152 | .1693 |
|  | 2024 | -.6548 | .07481 | 1 | .000 | -.8014 | -.5081 |
|  | 2025 | -.2210 | .10380 | 1 | .033 | -.4244 | -.0175 |
| 2023 | 2020 | -.4394 | .19788 | 1 | .026 | -.8272 | -.0515 |
|  | 2021 | -1.5671 | .10847 | 1 | .000 | -1.7797 | -1.3545 |
|  | 2022 | -.0271 | .07255 | 1 | .709 | -.1693 | .1152 |
|  | 2024 | -.6818 | .05444 | 1 | .000 | -.7885 | -.5751 |
|  | 2025 | -.2480 | .09029 | 1 | .006 | -.4250 | -.0711 |
| 2024 | 2020 | .2425 | .19876 | 1 | .223 | -.1471 | .6320 |
|  | 2021 | -.8853 | .11005 | 1 | .000 | -1.1010 | -.6696 |
|  | 2022 | .6548 | .07481 | 1 | .000 | .5081 | .8014 |
|  | 2023 | .6818 | .05444 | 1 | .000 | .5751 | .7885 |
|  | 2025 | .4338 | .09218 | 1 | .000 | .2531 | .6145 |
| 2025 | 2020 | -.1913 | .21090 | 1 | .364 | -.6047 | .2220 |
|  | 2021 | -1.3191 | .13088 | 1 | .000 | -1.5756 | -1.0626 |
|  | 2022 | .2210 | .10380 | 1 | .033 | .0175 | .4244 |
|  | 2023 | .2480 | .09029 | 1 | .006 | .0711 | .4250 |
|  | 2024 | -.4338 | .09218 | 1 | .000 | -.6145 | -.2531 |

| **Overall Test** | | |
| --- | --- | --- |
| Wald χ² | Degrees of Freedom | P |
| 329.418 | 5 | .000 |

**Estimated Marginal Means 2：Gender**

| **Estimate** | | | | |
| --- | --- | --- | --- | --- |
| Gender | Mean | Standard Error | 95% Wald Confidence Interval | |
|  |  |  | Lower Bound | Upper Bound |
| 1 | 9.4127 | .04969 | 9.3158 | 9.5106 |
| 2 | 9.5590 | .05715 | 9.4477 | 9.6717 |

| **Pairwise Comparisons** | | | | | | | |
| --- | --- | --- | --- | --- | --- | --- | --- |
| (I) Gender | (J) Gender | Mean Difference (I-J) | Standard Error | Degrees of Freedom | P | 95% Wald Confidence Interval | |
|  |  |  |  |  |  | Lower Bound | Upper Bound |
| 1 | 2 | -.1464 | .05881 | 1 | .013 | -.2616 | -.0311 |
| 2 | 1 | .1464 | .05881 | 1 | .013 | .0311 | .2616 |

| **Overall Test** | | |
| --- | --- | --- |
| Wald χ² | Degrees of Freedom | P |
| 6.194 | 1 | .013 |

**Estimated Marginal Means 3：Age**

| **Estimate** | | | | |
| --- | --- | --- | --- | --- |
| Age | Mean | Standard Error | 95% Wald Confidence Interval | |
|  |  |  | Lower Bound | Upper Bound |
| 1 | 10.0593 | .11062 | 9.8448 | 10.2785 |
| 2 | 9.0451 | .03746 | 8.9720 | 9.1188 |
| 3 | 9.5650 | .08637 | 9.3972 | 9.7357 |
| 4 | 9.3023 | .10219 | 9.1041 | 9.5047 |

| **Pairwise Comparisons** | | | | | | | |
| --- | --- | --- | --- | --- | --- | --- | --- |
| (I) Age | (J) Age | Mean Difference (I-J) | Standard Error | Degrees of Freedom | P | 95% Wald Confidence Interval | |
|  |  |  |  |  |  | Lower Bound | Upper Bound |
| 1 | 2 | 1.0142 | .11388 | 1 | .000 | .7910 | 1.2374 |
|  | 3 | .4944 | .13813 | 1 | .000 | .2236 | .7651 |
|  | 4 | .7571 | .15104 | 1 | .000 | .4610 | 1.0531 |
| 2 | 1 | -1.0142 | .11388 | 1 | .000 | -1.2374 | -.7910 |
|  | 3 | -.5199 | .09266 | 1 | .000 | -.7015 | -.3383 |
|  | 4 | -.2572 | .10887 | 1 | .018 | -.4706 | -.0438 |
| 3 | 1 | -.4944 | .13813 | 1 | .000 | -.7651 | -.2236 |
|  | 2 | .5199 | .09266 | 1 | .000 | .3383 | .7015 |
|  | 4 | .2627 | .13382 | 1 | .050 | .0004 | .5250 |
| 4 | 1 | -.7571 | .15104 | 1 | .000 | -1.0531 | -.4610 |
|  | 2 | .2572 | .10887 | 1 | .018 | .0438 | .4706 |
|  | 3 | -.2627 | .13382 | 1 | .050 | -.5250 | -.0004 |

| **Overall Test** | | |
| --- | --- | --- |
| Wald χ² | Degrees of Freedom | P |
| 101.712 | 3 | .000 |

**Estimated Marginal Means 4：Diagnostic**

| **Estimate** | | | | |
| --- | --- | --- | --- | --- |
| Diagnostic | Mean | Standard Error | 95% Wald Confidence Interval | |
|  |  |  | Lower Bound | Upper Bound |
| 1 | 9.6033 | .04527 | 9.5149 | 9.6924 |
| 2 | 9.3693 | .07129 | 9.2306 | 9.5101 |

| **Pairwise Comparisons** | | | | | | | |
| --- | --- | --- | --- | --- | --- | --- | --- |
| (I) Diagnostic | (J) Diagnostic | Mean Difference (I-J) | Standard Error | Degrees of Freedom | P | 95% Wald Confidence Interval | |
|  |  |  |  |  |  | Lower Bound | Upper Bound |
| 1 | 2 | .2339 | .07961 | 1 | .003 | .0779 | .3900 |
| 2 | 1 | -.2339 | .07961 | 1 | .003 | -.3900 | -.0779 |

| **Overall Test** | | |
| --- | --- | --- |
| Wald χ² | Degrees of Freedom | P |
| 8.635 | 1 | .003 |

**Estimated Marginal Means 5：Year* Gender**

| **Estimate** | | | | | |
| --- | --- | --- | --- | --- | --- |
| Year | Gender | Mean | Standard Error | 95% Wald Confidence Interval | |
|  |  |  |  | Lower Bound | Upper Bound |
| 2020 | 1 | 9.2834 | .20902 | 8.8827 | 9.7023 |
|  | 2 | 9.6103 | .25320 | 9.1267 | 10.1196 |
| 2021 | 1 | 10.5536 | .12406 | 10.3132 | 10.7996 |
|  | 2 | 10.5929 | .13776 | 10.3263 | 10.8664 |
| 2022 | 1 | 9.0305 | .07492 | 8.8849 | 9.1786 |
|  | 2 | 9.0358 | .08315 | 8.8743 | 9.2003 |
| 2023 | 1 | 8.9802 | .04496 | 8.8925 | 9.0688 |
|  | 2 | 9.0321 | .04904 | 8.9365 | 9.1287 |
| 2024 | 1 | 9.6326 | .04854 | 9.5379 | 9.7282 |
|  | 2 | 9.7436 | .05388 | 9.6386 | 9.8498 |
| 2025 | 1 | 9.0870 | .09484 | 8.9030 | 9.2748 |
|  | 2 | 9.4244 | .10801 | 9.2150 | 9.6384 |

| **Pairwise Comparisons** | | | | | | | | |
| --- | --- | --- | --- | --- | --- | --- | --- | --- |
| Gender | (I) Year | (J) Year | Mean Difference (I-J) | Standard Error | Degrees of Freedom | P | 95% Wald Confidence Interval | |
|  |  |  |  |  |  |  | Lower Bound | Upper Bound |
| 1 | 2020 | 2021 | -1.2702 | .24111 | 1 | .000 | -1.7427 | -.7976 |
|  |  | 2022 | .2529 | .22058 | 1 | .252 | -.1794 | .6852 |
|  |  | 2023 | .3032 | .21255 | 1 | .154 | -.1134 | .7198 |
|  |  | 2024 | -.3491 | .21333 | 1 | .102 | -.7672 | .0690 |
|  |  | 2025 | .1964 | .22721 | 1 | .387 | -.2489 | .6418 |
|  | 2021 | 2020 | 1.2702 | .24111 | 1 | .000 | .7976 | 1.7427 |
|  |  | 2022 | 1.5231 | .14158 | 1 | .000 | 1.2456 | 1.8005 |
|  |  | 2023 | 1.5734 | .12851 | 1 | .000 | 1.3215 | 1.8252 |
|  |  | 2024 | .9210 | .12974 | 1 | .000 | .6667 | 1.1753 |
|  |  | 2025 | 1.4666 | .15269 | 1 | .000 | 1.1673 | 1.7659 |
|  | 2022 | 2020 | -.2529 | .22058 | 1 | .252 | -.6852 | .1794 |
|  |  | 2021 | -1.5231 | .14158 | 1 | .000 | -1.8005 | -1.2456 |
|  |  | 2023 | .0503 | .08354 | 1 | .547 | -.1134 | .2140 |
|  |  | 2024 | -.6020 | .08538 | 1 | .000 | -.7694 | -.4347 |
|  |  | 2025 | -.0565 | .11771 | 1 | .631 | -.2872 | .1742 |
|  | 2023 | 2020 | -.3032 | .21255 | 1 | .154 | -.7198 | .1134 |
|  |  | 2021 | -1.5734 | .12851 | 1 | .000 | -1.8252 | -1.3215 |
|  |  | 2022 | -.0503 | .08354 | 1 | .547 | -.2140 | .1134 |
|  |  | 2024 | -.6523 | .06088 | 1 | .000 | -.7716 | -.5330 |
|  |  | 2025 | -.1068 | .10192 | 1 | .295 | -.3065 | .0930 |
|  | 2024 | 2020 | .3491 | .21333 | 1 | .102 | -.0690 | .7672 |
|  |  | 2021 | -.9210 | .12974 | 1 | .000 | -1.1753 | -.6667 |
|  |  | 2022 | .6020 | .08538 | 1 | .000 | .4347 | .7694 |
|  |  | 2023 | .6523 | .06088 | 1 | .000 | .5330 | .7716 |
|  |  | 2025 | .5456 | .10347 | 1 | .000 | .3428 | .7484 |
|  | 2025 | 2020 | -.1964 | .22721 | 1 | .387 | -.6418 | .2489 |
|  |  | 2021 | -1.4666 | .15269 | 1 | .000 | -1.7659 | -1.1673 |
|  |  | 2022 | .0565 | .11771 | 1 | .631 | -.1742 | .2872 |
|  |  | 2023 | .1068 | .10192 | 1 | .295 | -.0930 | .3065 |
|  |  | 2024 | -.5456 | .10347 | 1 | .000 | -.7484 | -.3428 |
| 2 | 2020 | 2021 | -.9826 | .28625 | 1 | .001 | -1.5436 | -.4216 |
|  |  | 2022 | .5745 | .26530 | 1 | .030 | .0545 | 1.0945 |
|  |  | 2023 | .5783 | .25722 | 1 | .025 | .0741 | 1.0824 |
|  |  | 2024 | -.1333 | .25813 | 1 | .606 | -.6392 | .3726 |
|  |  | 2025 | .1860 | .27232 | 1 | .495 | -.3477 | .7197 |
|  | 2021 | 2020 | .9826 | .28625 | 1 | .001 | .4216 | 1.5436 |
|  |  | 2022 | 1.5571 | .15728 | 1 | .000 | 1.2489 | 1.8654 |
|  |  | 2023 | 1.5609 | .14267 | 1 | .000 | 1.2812 | 1.8405 |
|  |  | 2024 | .8493 | .14405 | 1 | .000 | .5670 | 1.1316 |
|  |  | 2025 | 1.1686 | .17150 | 1 | .000 | .8324 | 1.5047 |
|  | 2022 | 2020 | -.5745 | .26530 | 1 | .030 | -1.0945 | -.0545 |
|  |  | 2021 | -1.5571 | .15728 | 1 | .000 | -1.8654 | -1.2489 |
|  |  | 2023 | .0037 | .09177 | 1 | .968 | -.1761 | .1836 |
|  |  | 2024 | -.7078 | .09383 | 1 | .000 | -.8917 | -.5239 |
|  |  | 2025 | -.3885 | .13339 | 1 | .004 | -.6500 | -.1271 |
|  | 2023 | 2020 | -.5783 | .25722 | 1 | .025 | -1.0824 | -.0741 |
|  |  | 2021 | -1.5609 | .14267 | 1 | .000 | -1.8405 | -1.2812 |
|  |  | 2022 | -.0037 | .09177 | 1 | .968 | -.1836 | .1761 |
|  |  | 2024 | -.7116 | .06566 | 1 | .000 | -.8402 | -.5829 |
|  |  | 2025 | -.3923 | .11614 | 1 | .001 | -.6199 | -.1646 |
|  | 2024 | 2020 | .1333 | .25813 | 1 | .606 | -.3726 | .6392 |
|  |  | 2021 | -.8493 | .14405 | 1 | .000 | -1.1316 | -.5670 |
|  |  | 2022 | .7078 | .09383 | 1 | .000 | .5239 | .8917 |
|  |  | 2023 | .7116 | .06566 | 1 | .000 | .5829 | .8402 |
|  |  | 2025 | .3193 | .11805 | 1 | .007 | .0879 | .5507 |
|  | 2025 | 2020 | -.1860 | .27232 | 1 | .495 | -.7197 | .3477 |
|  |  | 2021 | -1.1686 | .17150 | 1 | .000 | -1.5047 | -.8324 |
|  |  | 2022 | .3885 | .13339 | 1 | .004 | .1271 | .6500 |
|  |  | 2023 | .3923 | .11614 | 1 | .001 | .1646 | .6199 |
|  |  | 2024 | -.3193 | .11805 | 1 | .007 | -.5507 | -.0879 |

| **Overall Test** | | | |
| --- | --- | --- | --- |
| Gender | Wald χ² | Degrees of Freedom | P |
| 1 | 240.742 | 5 | .000 |
| 2 | 217.720 | 5 | .000 |

**Estimated Marginal Means 6：Year* Gender**

| **Estimate** | | | | | |
| --- | --- | --- | --- | --- | --- |
| Year | Gender | Mean | Standard Error | 95% Wald Confidence Interval | |
|  |  |  |  | Lower Bound | Upper Bound |
| 2020 | 1 | 9.2834 | .20902 | 8.8827 | 9.7023 |
|  | 2 | 9.6103 | .25320 | 9.1267 | 10.1196 |
| 2021 | 1 | 10.5536 | .12406 | 10.3132 | 10.7996 |
|  | 2 | 10.5929 | .13776 | 10.3263 | 10.8664 |
| 2022 | 1 | 9.0305 | .07492 | 8.8849 | 9.1786 |
|  | 2 | 9.0358 | .08315 | 8.8743 | 9.2003 |
| 2023 | 1 | 8.9802 | .04496 | 8.8925 | 9.0688 |
|  | 2 | 9.0321 | .04904 | 8.9365 | 9.1287 |
| 2024 | 1 | 9.6326 | .04854 | 9.5379 | 9.7282 |
|  | 2 | 9.7436 | .05388 | 9.6386 | 9.8498 |
| 2025 | 1 | 9.0870 | .09484 | 8.9030 | 9.2748 |
|  | 2 | 9.4244 | .10801 | 9.2150 | 9.6384 |

| **Pairwise Comparisons** | | | | | | | | |
| --- | --- | --- | --- | --- | --- | --- | --- | --- |
| Year | (I) Gender | (J) Gender | Mean Difference (I-J) | Standard Error | Degrees of Freedom | P | 95% Wald Confidence Interval | |
|  |  |  |  |  |  |  | Lower Bound | Upper Bound |
| 2020 | 1 | 2 | -.3269 | .24912 | 1 | .189 | -.8152 | .1614 |
|  | 2 | 1 | .3269 | .24912 | 1 | .189 | -.1614 | .8152 |
| 2021 | 1 | 2 | -.0393 | .15908 | 1 | .805 | -.3511 | .2725 |
|  | 2 | 1 | .0393 | .15908 | 1 | .805 | -.2725 | .3511 |
| 2022 | 1 | 2 | -.0053 | .09189 | 1 | .954 | -.1854 | .1748 |
|  | 2 | 1 | .0053 | .09189 | 1 | .954 | -.1748 | .1854 |
| 2023 | 1 | 2 | -.0518 | .05328 | 1 | .331 | -.1563 | .0526 |
|  | 2 | 1 | .0518 | .05328 | 1 | .331 | -.0526 | .1563 |
| 2024 | 1 | 2 | -.1111 | .05668 | 1 | .050 | -.2221 | .0000 |
|  | 2 | 1 | .1111 | .05668 | 1 | .050 | .0000 | .2221 |
| 2025 | 1 | 2 | -.3373 | .11121 | 1 | .002 | -.5553 | -.1194 |
|  | 2 | 1 | .3373 | .11121 | 1 | .002 | .1194 | .5553 |

| **Overall Test** | | | |
| --- | --- | --- | --- |
| Year | Wald χ² | Degrees of Freedom | P |
| 2020 | 1.722 | 1 | .189 |
| 2021 | .061 | 1 | .805 |
| 2022 | .003 | 1 | .954 |
| 2023 | .947 | 1 | .331 |
| 2024 | 3.840 | 1 | .050 |
| 2025 | 9.201 | 1 | .002 |

**Estimated Marginal Means 7：Year* Age**

| **Estimate** | | | | | |
| --- | --- | --- | --- | --- | --- |
| Year | Age | Mean | Standard Error | 95% Wald Confidence Interval | |
|  |  |  |  | Lower Bound | Upper Bound |
| 2020 | 1 | 9.7097 | .27708 | 9.1815 | 10.2682 |
|  | 2 | 8.3400 | .16331 | 8.0260 | 8.6664 |
|  | 3 | 10.0499 | .47272 | 9.1648 | 11.0205 |
|  | 4 | 9.7805 | .53221 | 8.7911 | 10.8813 |
| 2021 | 1 | 12.0700 | .31443 | 11.4692 | 12.7023 |
|  | 2 | 9.7407 | .09498 | 9.5563 | 9.9286 |
|  | 3 | 10.3577 | .16337 | 10.0424 | 10.6829 |
|  | 4 | 10.2629 | .22046 | 9.8398 | 10.7042 |
| 2022 | 1 | 9.5273 | .19500 | 9.1526 | 9.9172 |
|  | 2 | 8.8224 | .05648 | 8.7124 | 8.9338 |
|  | 3 | 9.1249 | .08473 | 8.9603 | 9.2925 |
|  | 4 | 8.6812 | .13653 | 8.4177 | 8.9530 |
| 2023 | 1 | 9.3073 | .14181 | 9.0335 | 9.5894 |
|  | 2 | 9.0846 | .03540 | 9.0155 | 9.1543 |
|  | 3 | 8.9639 | .04018 | 8.8855 | 9.0430 |
|  | 4 | 8.6800 | .04403 | 8.5942 | 8.7668 |
| 2024 | 1 | 9.4464 | .14803 | 9.1607 | 9.7410 |
|  | 2 | 9.1760 | .03215 | 9.1132 | 9.2392 |
|  | 3 | 10.1244 | .04411 | 10.0383 | 10.2112 |
|  | 4 | 10.0379 | .05475 | 9.9311 | 10.1457 |
| 2025 | 1 | 10.5546 | .16911 | 10.2283 | 10.8913 |
|  | 2 | 9.1659 | .07488 | 9.0203 | 9.3138 |
|  | 3 | 8.8833 | .16090 | 8.5735 | 9.2043 |
|  | 4 | 8.5341 | .21011 | 8.1320 | 8.9560 |

| **Pairwise Comparisons** | | | | | | | | |
| --- | --- | --- | --- | --- | --- | --- | --- | --- |
| Age | (I) Year | (J) Year | Mean Difference (I-J) | Standard Error | Degrees of Freedom | P | 95% Wald Confidence Interval | |
|  |  |  |  |  |  |  | Lower Bound | Upper Bound |
| 1 | 2020 | 2021 | -2.3604 | .39630 | 1 | .000 | -3.1371 | -1.5836 |
|  |  | 2022 | .1824 | .32176 | 1 | .571 | -.4482 | .8130 |
|  |  | 2023 | .4023 | .29393 | 1 | .171 | -.1737 | .9784 |
|  |  | 2024 | .2633 | .29858 | 1 | .378 | -.3219 | .8485 |
|  |  | 2025 | -.8449 | .29497 | 1 | .004 | -1.4230 | -.2668 |
|  | 2021 | 2020 | 2.3604 | .39630 | 1 | .000 | 1.5836 | 3.1371 |
|  |  | 2022 | 2.5428 | .35283 | 1 | .000 | 1.8513 | 3.2343 |
|  |  | 2023 | 2.7627 | .32772 | 1 | .000 | 2.1204 | 3.4051 |
|  |  | 2024 | 2.6236 | .33207 | 1 | .000 | 1.9728 | 3.2745 |
|  |  | 2025 | 1.5155 | .32750 | 1 | .000 | .8736 | 2.1574 |
|  | 2022 | 2020 | -.1824 | .32176 | 1 | .571 | -.8130 | .4482 |
|  |  | 2021 | -2.5428 | .35283 | 1 | .000 | -3.2343 | -1.8513 |
|  |  | 2023 | .2200 | .22573 | 1 | .330 | -.2225 | .6624 |
|  |  | 2024 | .0809 | .23097 | 1 | .726 | -.3718 | .5336 |
|  |  | 2025 | -1.0273 | .23322 | 1 | .000 | -1.4844 | -.5702 |
|  | 2023 | 2020 | -.4023 | .29393 | 1 | .171 | -.9784 | .1737 |
|  |  | 2021 | -2.7627 | .32772 | 1 | .000 | -3.4051 | -2.1204 |
|  |  | 2022 | -.2200 | .22573 | 1 | .330 | -.6624 | .2225 |
|  |  | 2024 | -.1391 | .18942 | 1 | .463 | -.5103 | .2322 |
|  |  | 2025 | -1.2473 | .19318 | 1 | .000 | -1.6259 | -.8686 |
|  | 2024 | 2020 | -.2633 | .29858 | 1 | .378 | -.8485 | .3219 |
|  |  | 2021 | -2.6236 | .33207 | 1 | .000 | -3.2745 | -1.9728 |
|  |  | 2022 | -.0809 | .23097 | 1 | .726 | -.5336 | .3718 |
|  |  | 2023 | .1391 | .18942 | 1 | .463 | -.2322 | .5103 |
|  |  | 2025 | -1.1082 | .20042 | 1 | .000 | -1.5010 | -.7154 |
|  | 2025 | 2020 | .8449 | .29497 | 1 | .004 | .2668 | 1.4230 |
|  |  | 2021 | -1.5155 | .32750 | 1 | .000 | -2.1574 | -.8736 |
|  |  | 2022 | 1.0273 | .23322 | 1 | .000 | .5702 | 1.4844 |
|  |  | 2023 | 1.2473 | .19318 | 1 | .000 | .8686 | 1.6259 |
|  |  | 2024 | 1.1082 | .20042 | 1 | .000 | .7154 | 1.5010 |
| 2 | 2020 | 2021 | -1.4006 | .18887 | 1 | .000 | -1.7708 | -1.0304 |
|  |  | 2022 | -.4823 | .17274 | 1 | .005 | -.8209 | -.1438 |
|  |  | 2023 | -.7446 | .16706 | 1 | .000 | -1.0720 | -.4171 |
|  |  | 2024 | -.8359 | .16639 | 1 | .000 | -1.1620 | -.5098 |
|  |  | 2025 | -.8258 | .17947 | 1 | .000 | -1.1776 | -.4741 |
|  | 2021 | 2020 | 1.4006 | .18887 | 1 | .000 | 1.0304 | 1.7708 |
|  |  | 2022 | .9183 | .11045 | 1 | .000 | .7018 | 1.1348 |
|  |  | 2023 | .6560 | .10131 | 1 | .000 | .4575 | .8546 |
|  |  | 2024 | .5647 | .10022 | 1 | .000 | .3683 | .7611 |
|  |  | 2025 | .5748 | .12085 | 1 | .000 | .3379 | .8117 |
|  | 2022 | 2020 | .4823 | .17274 | 1 | .005 | .1438 | .8209 |
|  |  | 2021 | -.9183 | .11045 | 1 | .000 | -1.1348 | -.7018 |
|  |  | 2023 | -.2623 | .06657 | 1 | .000 | -.3927 | -.1318 |
|  |  | 2024 | -.3536 | .06487 | 1 | .000 | -.4808 | -.2265 |
|  |  | 2025 | -.3435 | .09366 | 1 | .000 | -.5271 | -.1599 |
|  | 2023 | 2020 | .7446 | .16706 | 1 | .000 | .4171 | 1.0720 |
|  |  | 2021 | -.6560 | .10131 | 1 | .000 | -.8546 | -.4575 |
|  |  | 2022 | .2623 | .06657 | 1 | .000 | .1318 | .3927 |
|  |  | 2024 | -.0913 | .04768 | 1 | .055 | -.1848 | .0021 |
|  |  | 2025 | -.0813 | .08272 | 1 | .326 | -.2434 | .0809 |
|  | 2024 | 2020 | .8359 | .16639 | 1 | .000 | .5098 | 1.1620 |
|  |  | 2021 | -.5647 | .10022 | 1 | .000 | -.7611 | -.3683 |
|  |  | 2022 | .3536 | .06487 | 1 | .000 | .2265 | .4808 |
|  |  | 2023 | .0913 | .04768 | 1 | .055 | -.0021 | .1848 |
|  |  | 2025 | .0101 | .08136 | 1 | .901 | -.1494 | .1695 |
|  | 2025 | 2020 | .8258 | .17947 | 1 | .000 | .4741 | 1.1776 |
|  |  | 2021 | -.5748 | .12085 | 1 | .000 | -.8117 | -.3379 |
|  |  | 2022 | .3435 | .09366 | 1 | .000 | .1599 | .5271 |
|  |  | 2023 | .0813 | .08272 | 1 | .326 | -.0809 | .2434 |
|  |  | 2024 | -.0101 | .08136 | 1 | .901 | -.1695 | .1494 |
| 3 | 2020 | 2021 | -.3078 | .49994 | 1 | .538 | -1.2877 | .6720 |
|  |  | 2022 | .9250 | .48008 | 1 | .054 | -.0160 | 1.8659 |
|  |  | 2023 | 1.0860 | .47433 | 1 | .022 | .1563 | 2.0157 |
|  |  | 2024 | -.0744 | .47459 | 1 | .875 | -1.0046 | .8557 |
|  |  | 2025 | 1.1666 | .49914 | 1 | .019 | .1883 | 2.1449 |
|  | 2021 | 2020 | .3078 | .49994 | 1 | .538 | -.6720 | 1.2877 |
|  |  | 2022 | 1.2328 | .18369 | 1 | .000 | .8728 | 1.5928 |
|  |  | 2023 | 1.3938 | .16804 | 1 | .000 | 1.0645 | 1.7232 |
|  |  | 2024 | .2334 | .16881 | 1 | .167 | -.0975 | .5643 |
|  |  | 2025 | 1.4744 | .22895 | 1 | .000 | 1.0257 | 1.9232 |
|  | 2022 | 2020 | -.9250 | .48008 | 1 | .054 | -1.8659 | .0160 |
|  |  | 2021 | -1.2328 | .18369 | 1 | .000 | -1.5928 | -.8728 |
|  |  | 2023 | .1610 | .09350 | 1 | .085 | -.0223 | .3442 |
|  |  | 2024 | -.9994 | .09497 | 1 | .000 | -1.1856 | -.8133 |
|  |  | 2025 | .2416 | .18152 | 1 | .183 | -.1142 | .5974 |
|  | 2023 | 2020 | -1.0860 | .47433 | 1 | .022 | -2.0157 | -.1563 |
|  |  | 2021 | -1.3938 | .16804 | 1 | .000 | -1.7232 | -1.0645 |
|  |  | 2022 | -.1610 | .09350 | 1 | .085 | -.3442 | .0223 |
|  |  | 2024 | -1.1604 | .05917 | 1 | .000 | -1.2764 | -1.0445 |
|  |  | 2025 | .0806 | .16565 | 1 | .626 | -.2440 | .4053 |
|  | 2024 | 2020 | .0744 | .47459 | 1 | .875 | -.8557 | 1.0046 |
|  |  | 2021 | -.2334 | .16881 | 1 | .167 | -.5643 | .0975 |
|  |  | 2022 | .9994 | .09497 | 1 | .000 | .8133 | 1.1856 |
|  |  | 2023 | 1.1604 | .05917 | 1 | .000 | 1.0445 | 1.2764 |
|  |  | 2025 | 1.2410 | .16645 | 1 | .000 | .9148 | 1.5673 |
|  | 2025 | 2020 | -1.1666 | .49914 | 1 | .019 | -2.1449 | -.1883 |
|  |  | 2021 | -1.4744 | .22895 | 1 | .000 | -1.9232 | -1.0257 |
|  |  | 2022 | -.2416 | .18152 | 1 | .183 | -.5974 | .1142 |
|  |  | 2023 | -.0806 | .16565 | 1 | .626 | -.4053 | .2440 |
|  |  | 2024 | -1.2410 | .16645 | 1 | .000 | -1.5673 | -.9148 |
| 4 | 2020 | 2021 | -.4824 | .57596 | 1 | .402 | -1.6113 | .6465 |
|  |  | 2022 | 1.0993 | .54931 | 1 | .045 | .0227 | 2.1759 |
|  |  | 2023 | 1.1005 | .53401 | 1 | .039 | .0538 | 2.1471 |
|  |  | 2024 | -.2574 | .53498 | 1 | .630 | -1.3059 | .7912 |
|  |  | 2025 | 1.2464 | .57203 | 1 | .029 | .1253 | 2.3676 |
|  | 2021 | 2020 | .4824 | .57596 | 1 | .402 | -.6465 | 1.6113 |
|  |  | 2022 | 1.5817 | .25923 | 1 | .000 | 1.0736 | 2.0898 |
|  |  | 2023 | 1.5829 | .22462 | 1 | .000 | 1.1426 | 2.0231 |
|  |  | 2024 | .2250 | .22692 | 1 | .321 | -.2197 | .6698 |
|  |  | 2025 | 1.7288 | .30444 | 1 | .000 | 1.1322 | 2.3255 |
|  | 2022 | 2020 | -1.0993 | .54931 | 1 | .045 | -2.1759 | -.0227 |
|  |  | 2021 | -1.5817 | .25923 | 1 | .000 | -2.0898 | -1.0736 |
|  |  | 2023 | .0012 | .14335 | 1 | .994 | -.2798 | .2821 |
|  |  | 2024 | -1.3567 | .14696 | 1 | .000 | -1.6447 | -1.0686 |
|  |  | 2025 | .1471 | .25049 | 1 | .557 | -.3438 | .6381 |
|  | 2023 | 2020 | -1.1005 | .53401 | 1 | .039 | -2.1471 | -.0538 |
|  |  | 2021 | -1.5829 | .22462 | 1 | .000 | -2.0231 | -1.1426 |
|  |  | 2022 | -.0012 | .14335 | 1 | .994 | -.2821 | .2798 |
|  |  | 2024 | -1.3578 | .06957 | 1 | .000 | -1.4942 | -1.2215 |
|  |  | 2025 | .1460 | .21457 | 1 | .496 | -.2746 | .5665 |
|  | 2024 | 2020 | .2574 | .53498 | 1 | .630 | -.7912 | 1.3059 |
|  |  | 2021 | -.2250 | .22692 | 1 | .321 | -.6698 | .2197 |
|  |  | 2022 | 1.3567 | .14696 | 1 | .000 | 1.0686 | 1.6447 |
|  |  | 2023 | 1.3578 | .06957 | 1 | .000 | 1.2215 | 1.4942 |
|  |  | 2025 | 1.5038 | .21699 | 1 | .000 | 1.0785 | 1.9291 |
|  | 2025 | 2020 | -1.2464 | .57203 | 1 | .029 | -2.3676 | -.1253 |
|  |  | 2021 | -1.7288 | .30444 | 1 | .000 | -2.3255 | -1.1322 |
|  |  | 2022 | -.1471 | .25049 | 1 | .557 | -.6381 | .3438 |
|  |  | 2023 | -.1460 | .21457 | 1 | .496 | -.5665 | .2746 |
|  |  | 2024 | -1.5038 | .21699 | 1 | .000 | -1.9291 | -1.0785 |

| **Overall Test** | | | |
| --- | --- | --- | --- |
| Age | Wald χ² | Degrees of Freedom | P |
| 1 | 104.431 | 5 | .000 |
| 2 | 97.662 | 5 | .000 |
| 3 | 446.608 | 5 | .000 |
| 4 | 429.855 | 5 | .000 |

**Estimated Marginal Means 8：Year* Age**

| **Estimate** | | | | | |
| --- | --- | --- | --- | --- | --- |
| Year | Age | Mean | Standard Error | 95% Wald Confidence Interval | |
|  |  |  |  | Lower Bound | Upper Bound |
| 2020 | 1 | 9.7097 | .27708 | 9.1815 | 10.2682 |
|  | 2 | 8.3400 | .16331 | 8.0260 | 8.6664 |
|  | 3 | 10.0499 | .47272 | 9.1648 | 11.0205 |
|  | 4 | 9.7805 | .53221 | 8.7911 | 10.8813 |
| 2021 | 1 | 12.0700 | .31443 | 11.4692 | 12.7023 |
|  | 2 | 9.7407 | .09498 | 9.5563 | 9.9286 |
|  | 3 | 10.3577 | .16337 | 10.0424 | 10.6829 |
|  | 4 | 10.2629 | .22046 | 9.8398 | 10.7042 |
| 2022 | 1 | 9.5273 | .19500 | 9.1526 | 9.9172 |
|  | 2 | 8.8224 | .05648 | 8.7124 | 8.9338 |
|  | 3 | 9.1249 | .08473 | 8.9603 | 9.2925 |
|  | 4 | 8.6812 | .13653 | 8.4177 | 8.9530 |
| 2023 | 1 | 9.3073 | .14181 | 9.0335 | 9.5894 |
|  | 2 | 9.0846 | .03540 | 9.0155 | 9.1543 |
|  | 3 | 8.9639 | .04018 | 8.8855 | 9.0430 |
|  | 4 | 8.6800 | .04403 | 8.5942 | 8.7668 |
| 2024 | 1 | 9.4464 | .14803 | 9.1607 | 9.7410 |
|  | 2 | 9.1760 | .03215 | 9.1132 | 9.2392 |
|  | 3 | 10.1244 | .04411 | 10.0383 | 10.2112 |
|  | 4 | 10.0379 | .05475 | 9.9311 | 10.1457 |
| 2025 | 1 | 10.5546 | .16911 | 10.2283 | 10.8913 |
|  | 2 | 9.1659 | .07488 | 9.0203 | 9.3138 |
|  | 3 | 8.8833 | .16090 | 8.5735 | 9.2043 |
|  | 4 | 8.5341 | .21011 | 8.1320 | 8.9560 |

| **Pairwise Comparisons** | | | | | | | | |
| --- | --- | --- | --- | --- | --- | --- | --- | --- |
| Year | (I) Age | (J) Age | Mean Difference (I-J) | Standard Error | Degrees of Freedom | P | 95% Wald Confidence Interval | |
|  |  |  |  |  |  |  | Lower Bound | Upper Bound |
| 2020 | 1 | 2 | 1.3696 | .28646 | 1 | .000 | .8082 | 1.9311 |
|  |  | 3 | -.3403 | .52944 | 1 | .520 | -1.3779 | .6974 |
|  |  | 4 | -.0708 | .60499 | 1 | .907 | -1.2566 | 1.1149 |
|  | 2 | 1 | -1.3696 | .28646 | 1 | .000 | -1.9311 | -.8082 |
|  |  | 3 | -1.7099 | .49155 | 1 | .001 | -2.6733 | -.7464 |
|  |  | 4 | -1.4405 | .55743 | 1 | .010 | -2.5330 | -.3479 |
|  | 3 | 1 | .3403 | .52944 | 1 | .520 | -.6974 | 1.3779 |
|  |  | 2 | 1.7099 | .49155 | 1 | .001 | .7464 | 2.6733 |
|  |  | 4 | .2694 | .71277 | 1 | .705 | -1.1276 | 1.6664 |
|  | 4 | 1 | .0708 | .60499 | 1 | .907 | -1.1149 | 1.2566 |
|  |  | 2 | 1.4405 | .55743 | 1 | .010 | .3479 | 2.5330 |
|  |  | 3 | -.2694 | .71277 | 1 | .705 | -1.6664 | 1.1276 |
| 2021 | 1 | 2 | 2.3294 | .32667 | 1 | .000 | 1.6891 | 2.9696 |
|  |  | 3 | 1.7123 | .34948 | 1 | .000 | 1.0273 | 2.3973 |
|  |  | 4 | 1.8071 | .38155 | 1 | .000 | 1.0593 | 2.5550 |
|  | 2 | 1 | -2.3294 | .32667 | 1 | .000 | -2.9696 | -1.6891 |
|  |  | 3 | -.6171 | .18737 | 1 | .001 | -.9843 | -.2498 |
|  |  | 4 | -.5222 | .23923 | 1 | .029 | -.9911 | -.0534 |
|  | 3 | 1 | -1.7123 | .34948 | 1 | .000 | -2.3973 | -1.0273 |
|  |  | 2 | .6171 | .18737 | 1 | .001 | .2498 | .9843 |
|  |  | 4 | .0948 | .27303 | 1 | .728 | -.4403 | .6300 |
|  | 4 | 1 | -1.8071 | .38155 | 1 | .000 | -2.5550 | -1.0593 |
|  |  | 2 | .5222 | .23923 | 1 | .029 | .0534 | .9911 |
|  |  | 3 | -.0948 | .27303 | 1 | .728 | -.6300 | .4403 |
| 2022 | 1 | 2 | .7049 | .20176 | 1 | .000 | .3095 | 1.1003 |
|  |  | 3 | .4023 | .21093 | 1 | .056 | -.0111 | .8158 |
|  |  | 4 | .8461 | .23773 | 1 | .000 | .3801 | 1.3120 |
|  | 2 | 1 | -.7049 | .20176 | 1 | .000 | -1.1003 | -.3095 |
|  |  | 3 | -.3026 | .10072 | 1 | .003 | -.5000 | -.1051 |
|  |  | 4 | .1412 | .14751 | 1 | .339 | -.1480 | .4303 |
|  | 3 | 1 | -.4023 | .21093 | 1 | .056 | -.8158 | .0111 |
|  |  | 2 | .3026 | .10072 | 1 | .003 | .1051 | .5000 |
|  |  | 4 | .4437 | .16045 | 1 | .006 | .1292 | .7582 |
|  | 4 | 1 | -.8461 | .23773 | 1 | .000 | -1.3120 | -.3801 |
|  |  | 2 | -.1412 | .14751 | 1 | .339 | -.4303 | .1480 |
|  |  | 3 | -.4437 | .16045 | 1 | .006 | -.7582 | -.1292 |
| 2023 | 1 | 2 | .2227 | .14593 | 1 | .127 | -.0633 | .5087 |
|  |  | 3 | .3434 | .14714 | 1 | .020 | .0550 | .6318 |
|  |  | 4 | .6273 | .14820 | 1 | .000 | .3368 | .9177 |
|  | 2 | 1 | -.2227 | .14593 | 1 | .127 | -.5087 | .0633 |
|  |  | 3 | .1207 | .05341 | 1 | .024 | .0160 | .2254 |
|  |  | 4 | .4046 | .05634 | 1 | .000 | .2942 | .5150 |
|  | 3 | 1 | -.3434 | .14714 | 1 | .020 | -.6318 | -.0550 |
|  |  | 2 | -.1207 | .05341 | 1 | .024 | -.2254 | -.0160 |
|  |  | 4 | .2839 | .05943 | 1 | .000 | .1674 | .4004 |
|  | 4 | 1 | -.6273 | .14820 | 1 | .000 | -.9177 | -.3368 |
|  |  | 2 | -.4046 | .05634 | 1 | .000 | -.5150 | -.2942 |
|  |  | 3 | -.2839 | .05943 | 1 | .000 | -.4004 | -.1674 |
| 2024 | 1 | 2 | .2704 | .15121 | 1 | .074 | -.0260 | .5668 |
|  |  | 3 | -.6780 | .15404 | 1 | .000 | -.9799 | -.3760 |
|  |  | 4 | -.5915 | .15752 | 1 | .000 | -.9002 | -.2827 |
|  | 2 | 1 | -.2704 | .15121 | 1 | .074 | -.5668 | .0260 |
|  |  | 3 | -.9484 | .05426 | 1 | .000 | -1.0547 | -.8420 |
|  |  | 4 | -.8619 | .06327 | 1 | .000 | -.9859 | -.7379 |
|  | 3 | 1 | .6780 | .15404 | 1 | .000 | .3760 | .9799 |
|  |  | 2 | .9484 | .05426 | 1 | .000 | .8420 | 1.0547 |
|  |  | 4 | .0865 | .06994 | 1 | .216 | -.0506 | .2236 |
|  | 4 | 1 | .5915 | .15752 | 1 | .000 | .2827 | .9002 |
|  |  | 2 | .8619 | .06327 | 1 | .000 | .7379 | .9859 |
|  |  | 3 | -.0865 | .06994 | 1 | .216 | -.2236 | .0506 |
| 2025 | 1 | 2 | 1.3887 | .17506 | 1 | .000 | 1.0456 | 1.7318 |
|  |  | 3 | 1.6713 | .22795 | 1 | .000 | 1.2245 | 2.1181 |
|  |  | 4 | 2.0205 | .26865 | 1 | .000 | 1.4940 | 2.5470 |
|  | 2 | 1 | -1.3887 | .17506 | 1 | .000 | -1.7318 | -1.0456 |
|  |  | 3 | .2826 | .17441 | 1 | .105 | -.0593 | .6244 |
|  |  | 4 | .6318 | .22225 | 1 | .004 | .1962 | 1.0674 |
|  | 3 | 1 | -1.6713 | .22795 | 1 | .000 | -2.1181 | -1.2245 |
|  |  | 2 | -.2826 | .17441 | 1 | .105 | -.6244 | .0593 |
|  |  | 4 | .3492 | .26422 | 1 | .186 | -.1686 | .8671 |
|  | 4 | 1 | -2.0205 | .26865 | 1 | .000 | -2.5470 | -1.4940 |
|  |  | 2 | -.6318 | .22225 | 1 | .004 | -1.0674 | -.1962 |
|  |  | 3 | -.3492 | .26422 | 1 | .186 | -.8671 | .1686 |

| **Overall Test** | | | |
| --- | --- | --- | --- |
| Year | Wald χ² | Degrees of Freedom | P |
| 2020 | 34.578 | 3 | .000 |
| 2021 | 56.521 | 3 | .000 |
| 2022 | 21.756 | 3 | .000 |
| 2023 | 58.850 | 3 | .000 |
| 2024 | 383.980 | 3 | .000 |
| 2025 | 82.239 | 3 | .000 |

**Estimated Marginal Means 9：Year* Diagnostic**

| **Estimate** | | | | | |
| --- | --- | --- | --- | --- | --- |
| Year | Diagnostic | Mean | Standard Error | 95% Wald Confidence Interval | |
|  |  |  |  | Lower Bound | Upper Bound |
| 2020 | 1 | 10.0778 | .22125 | 9.6534 | 10.5209 |
|  | 2 | 8.8528 | .27567 | 8.3287 | 9.4099 |
| 2021 | 1 | 10.5448 | .11170 | 10.3282 | 10.7661 |
|  | 2 | 10.6017 | .15379 | 10.3046 | 10.9075 |
| 2022 | 1 | 9.2193 | .07161 | 9.0800 | 9.3607 |
|  | 2 | 8.8508 | .08904 | 8.6780 | 9.0270 |
| 2023 | 1 | 9.2518 | .04139 | 9.1711 | 9.3333 |
|  | 2 | 8.7669 | .05617 | 8.6575 | 8.8777 |
| 2024 | 1 | 9.5327 | .04398 | 9.4469 | 9.6193 |
|  | 2 | 9.8457 | .06314 | 9.7227 | 9.9703 |
| 2025 | 1 | 9.0775 | .08481 | 8.9127 | 9.2452 |
|  | 2 | 9.4343 | .13117 | 9.1806 | 9.6949 |

| **Pairwise Comparisons** | | | | | | | | |
| --- | --- | --- | --- | --- | --- | --- | --- | --- |
| Diagnostic | (I) Year | (J) Year | Mean Difference (I-J) | Standard Error | Degrees of Freedom | P | 95% Wald Confidence Interval | |
|  |  |  |  |  |  |  | Lower Bound | Upper Bound |
| 1 | 2020 | 2021 | -.4670 | .24756 | 1 | .059 | -.9522 | .0182 |
|  |  | 2022 | .8585 | .23227 | 1 | .000 | .4033 | 1.3137 |
|  |  | 2023 | .8260 | .22514 | 1 | .000 | .3847 | 1.2673 |
|  |  | 2024 | .5451 | .22557 | 1 | .016 | .1030 | .9872 |
|  |  | 2025 | 1.0003 | .23649 | 1 | .000 | .5368 | 1.4639 |
|  | 2021 | 2020 | .4670 | .24756 | 1 | .059 | -.0182 | .9522 |
|  |  | 2022 | 1.3255 | .13219 | 1 | .000 | 1.0664 | 1.5846 |
|  |  | 2023 | 1.2930 | .11887 | 1 | .000 | 1.0600 | 1.5260 |
|  |  | 2024 | 1.0122 | .11967 | 1 | .000 | .7776 | 1.2467 |
|  |  | 2025 | 1.4674 | .13984 | 1 | .000 | 1.1933 | 1.7415 |
|  | 2022 | 2020 | -.8585 | .23227 | 1 | .000 | -1.3137 | -.4033 |
|  |  | 2021 | -1.3255 | .13219 | 1 | .000 | -1.5846 | -1.0664 |
|  |  | 2023 | -.0325 | .08188 | 1 | .691 | -.1930 | .1280 |
|  |  | 2024 | -.3134 | .08289 | 1 | .000 | -.4758 | -.1509 |
|  |  | 2025 | .1418 | .11050 | 1 | .199 | -.0747 | .3584 |
|  | 2023 | 2020 | -.8260 | .22514 | 1 | .000 | -1.2673 | -.3847 |
|  |  | 2021 | -1.2930 | .11887 | 1 | .000 | -1.5260 | -1.0600 |
|  |  | 2022 | .0325 | .08188 | 1 | .691 | -.1280 | .1930 |
|  |  | 2024 | -.2809 | .05876 | 1 | .000 | -.3960 | -.1657 |
|  |  | 2025 | .1744 | .09434 | 1 | .065 | -.0106 | .3593 |
|  | 2024 | 2020 | -.5451 | .22557 | 1 | .016 | -.9872 | -.1030 |
|  |  | 2021 | -1.0122 | .11967 | 1 | .000 | -1.2467 | -.7776 |
|  |  | 2022 | .3134 | .08289 | 1 | .000 | .1509 | .4758 |
|  |  | 2023 | .2809 | .05876 | 1 | .000 | .1657 | .3960 |
|  |  | 2025 | .4552 | .09539 | 1 | .000 | .2683 | .6422 |
|  | 2025 | 2020 | -1.0003 | .23649 | 1 | .000 | -1.4639 | -.5368 |
|  |  | 2021 | -1.4674 | .13984 | 1 | .000 | -1.7415 | -1.1933 |
|  |  | 2022 | -.1418 | .11050 | 1 | .199 | -.3584 | .0747 |
|  |  | 2023 | -.1744 | .09434 | 1 | .065 | -.3593 | .0106 |
|  |  | 2024 | -.4552 | .09539 | 1 | .000 | -.6422 | -.2683 |
| 2 | 2020 | 2021 | -1.7489 | .30943 | 1 | .000 | -2.3554 | -1.1424 |
|  |  | 2022 | .0020 | .28490 | 1 | .994 | -.5564 | .5604 |
|  |  | 2023 | .0859 | .27655 | 1 | .756 | -.4562 | .6279 |
|  |  | 2024 | -.9929 | .27756 | 1 | .000 | -1.5369 | -.4489 |
|  |  | 2025 | -.5814 | .29914 | 1 | .052 | -1.1678 | .0049 |
|  | 2021 | 2020 | 1.7489 | .30943 | 1 | .000 | 1.1424 | 2.3554 |
|  |  | 2022 | 1.7509 | .16835 | 1 | .000 | 1.4210 | 2.0809 |
|  |  | 2023 | 1.8348 | .15376 | 1 | .000 | 1.5334 | 2.1361 |
|  |  | 2024 | .7560 | .15544 | 1 | .000 | .4513 | 1.0606 |
|  |  | 2025 | 1.1675 | .19131 | 1 | .000 | .7925 | 1.5424 |
|  | 2022 | 2020 | -.0020 | .28490 | 1 | .994 | -.5604 | .5564 |
|  |  | 2021 | -1.7509 | .16835 | 1 | .000 | -2.0809 | -1.4210 |
|  |  | 2023 | .0839 | .09381 | 1 | .371 | -.1000 | .2677 |
|  |  | 2024 | -.9949 | .09698 | 1 | .000 | -1.1850 | -.8049 |
|  |  | 2025 | -.5835 | .14867 | 1 | .000 | -.8749 | -.2921 |
|  | 2023 | 2020 | -.0859 | .27655 | 1 | .756 | -.6279 | .4562 |
|  |  | 2021 | -1.8348 | .15376 | 1 | .000 | -2.1361 | -1.5334 |
|  |  | 2022 | -.0839 | .09381 | 1 | .371 | -.2677 | .1000 |
|  |  | 2024 | -1.0788 | .06809 | 1 | .000 | -1.2122 | -.9453 |
|  |  | 2025 | -.6673 | .13205 | 1 | .000 | -.9261 | -.4085 |
|  | 2024 | 2020 | .9929 | .27756 | 1 | .000 | .4489 | 1.5369 |
|  |  | 2021 | -.7560 | .15544 | 1 | .000 | -1.0606 | -.4513 |
|  |  | 2022 | .9949 | .09698 | 1 | .000 | .8049 | 1.1850 |
|  |  | 2023 | 1.0788 | .06809 | 1 | .000 | .9453 | 1.2122 |
|  |  | 2025 | .4115 | .13403 | 1 | .002 | .1488 | .6742 |
|  | 2025 | 2020 | .5814 | .29914 | 1 | .052 | -.0049 | 1.1678 |
|  |  | 2021 | -1.1675 | .19131 | 1 | .000 | -1.5424 | -.7925 |
|  |  | 2022 | .5835 | .14867 | 1 | .000 | .2921 | .8749 |
|  |  | 2023 | .6673 | .13205 | 1 | .000 | .4085 | .9261 |
|  |  | 2024 | -.4115 | .13403 | 1 | .002 | -.6742 | -.1488 |

| **Overall Test** | | | |
| --- | --- | --- | --- |
| Diagnostic | Wald χ² | Degrees of Freedom | P |
| 1 | 159.084 | 5 | .000 |
| 2 | 362.209 | 5 | .000 |

**Estimated Marginal Means 10：Year* Diagnostic**

| **Estimate** | | | | | |
| --- | --- | --- | --- | --- | --- |
| Year | Diagnostic | Mean | Standard Error | 95% Wald Confidence Interval | |
|  |  |  |  | Lower Bound | Upper Bound |
| 2020 | 1 | 10.0778 | .22125 | 9.6534 | 10.5209 |
|  | 2 | 8.8528 | .27567 | 8.3287 | 9.4099 |
| 2021 | 1 | 10.5448 | .11170 | 10.3282 | 10.7661 |
|  | 2 | 10.6017 | .15379 | 10.3046 | 10.9075 |
| 2022 | 1 | 9.2193 | .07161 | 9.0800 | 9.3607 |
|  | 2 | 8.8508 | .08904 | 8.6780 | 9.0270 |
| 2023 | 1 | 9.2518 | .04139 | 9.1711 | 9.3333 |
|  | 2 | 8.7669 | .05617 | 8.6575 | 8.8777 |
| 2024 | 1 | 9.5327 | .04398 | 9.4469 | 9.6193 |
|  | 2 | 9.8457 | .06314 | 9.7227 | 9.9703 |
| 2025 | 1 | 9.0775 | .08481 | 8.9127 | 9.2452 |
|  | 2 | 9.4343 | .13117 | 9.1806 | 9.6949 |

| **Pairwise Comparisons** | | | | | | | | |
| --- | --- | --- | --- | --- | --- | --- | --- | --- |
| Year | (I) Diagnostic | (J) Diagnostic | Mean Difference (I-J) | Standard Error | Degrees of Freedom | P | 95% Wald Confidence Interval | |
|  |  |  |  |  |  |  | Lower Bound | Upper Bound |
| 2020 | 1 | 2 | 1.2250 | .31868 | 1 | .000 | .6004 | 1.8496 |
|  | 2 | 1 | -1.2250 | .31868 | 1 | .000 | -1.8496 | -.6004 |
| 2021 | 1 | 2 | -.0569 | .16964 | 1 | .737 | -.3894 | .2756 |
|  | 2 | 1 | .0569 | .16964 | 1 | .737 | -.2756 | .3894 |
| 2022 | 1 | 2 | .3685 | .09813 | 1 | .000 | .1762 | .5608 |
|  | 2 | 1 | -.3685 | .09813 | 1 | .000 | -.5608 | -.1762 |
| 2023 | 1 | 2 | .4849 | .06172 | 1 | .000 | .3639 | .6058 |
|  | 2 | 1 | -.4849 | .06172 | 1 | .000 | -.6058 | -.3639 |
| 2024 | 1 | 2 | -.3131 | .06693 | 1 | .000 | -.4442 | -.1819 |
|  | 2 | 1 | .3131 | .06693 | 1 | .000 | .1819 | .4442 |
| 2025 | 1 | 2 | -.3568 | .13987 | 1 | .011 | -.6309 | -.0827 |
|  | 2 | 1 | .3568 | .13987 | 1 | .011 | .0827 | .6309 |

| **Overall Test** | | | |
| --- | --- | --- | --- |
| Year | Wald χ² | Degrees of Freedom | P |
| 2020 | 14.775 | 1 | .000 |
| 2021 | .112 | 1 | .737 |
| 2022 | 14.103 | 1 | .000 |
| 2023 | 61.727 | 1 | .000 |
| 2024 | 21.878 | 1 | .000 |
| 2025 | 6.508 | 1 | .011 |

**Estimated Marginal Means 11：Gender* Age**

| **Estimate** | | | | | |
| --- | --- | --- | --- | --- | --- |
| Gender | Age | Mean | Standard Error | 95% Wald Confidence Interval | |
|  |  |  |  | Lower Bound | Upper Bound |
| 1 | 1 | 9.6740 | .11992 | 9.4418 | 9.9119 |
|  | 2 | 8.9938 | .04380 | 8.9084 | 9.0801 |
|  | 3 | 9.6127 | .09279 | 9.4325 | 9.7963 |
|  | 4 | 9.3855 | .10748 | 9.1772 | 9.5985 |
| 2 | 1 | 10.4600 | .14000 | 10.1892 | 10.7380 |
|  | 2 | 9.0966 | .05167 | 8.9959 | 9.1985 |
|  | 3 | 9.5174 | .09604 | 9.3311 | 9.7076 |
|  | 4 | 9.2198 | .11355 | 8.9999 | 9.4451 |

| **Pairwise Comparisons** | | | | | | | | |
| --- | --- | --- | --- | --- | --- | --- | --- | --- |
| Age | (I) Gender | (J) Gender | Mean Difference (I-J) | Standard Error | Degrees of Freedom | P | 95% Wald Confidence Interval | |
|  |  |  |  |  |  |  | Lower Bound | Upper Bound |
| 1 | 1 | 2 | -.7860 | .13680 | 1 | .000 | -1.0542 | -.5179 |
|  | 2 | 1 | .7860 | .13680 | 1 | .000 | .5179 | 1.0542 |
| 2 | 1 | 2 | -.1028 | .05963 | 1 | .085 | -.2197 | .0141 |
|  | 2 | 1 | .1028 | .05963 | 1 | .085 | -.0141 | .2197 |
| 3 | 1 | 2 | .0953 | .07640 | 1 | .213 | -.0545 | .2450 |
|  | 2 | 1 | -.0953 | .07640 | 1 | .213 | -.2450 | .0545 |
| 4 | 1 | 2 | .1657 | .08456 | 1 | .050 | -.0001 | .3314 |
|  | 2 | 1 | -.1657 | .08456 | 1 | .050 | -.3314 | .0001 |

| **Overall Test** | | | |
| --- | --- | --- | --- |
| Age | Wald χ² | Degrees of Freedom | P |
| 1 | 33.018 | 1 | .000 |
| 2 | 2.973 | 1 | .085 |
| 3 | 1.554 | 1 | .213 |
| 4 | 3.839 | 1 | .050 |

**Estimated Marginal Means 12：Gender* Age**

| **Estimate** | | | | | |
| --- | --- | --- | --- | --- | --- |
| Gender | Age | Mean | Standard Error | 95% Wald Confidence Interval | |
|  |  |  |  | Lower Bound | Upper Bound |
| 1 | 1 | 9.6740 | .11992 | 9.4418 | 9.9119 |
|  | 2 | 8.9938 | .04380 | 8.9084 | 9.0801 |
|  | 3 | 9.6127 | .09279 | 9.4325 | 9.7963 |
|  | 4 | 9.3855 | .10748 | 9.1772 | 9.5985 |
| 2 | 1 | 10.4600 | .14000 | 10.1892 | 10.7380 |
|  | 2 | 9.0966 | .05167 | 8.9959 | 9.1985 |
|  | 3 | 9.5174 | .09604 | 9.3311 | 9.7076 |
|  | 4 | 9.2198 | .11355 | 8.9999 | 9.4451 |

| **Pairwise Comparisons** | | | | | | | | |
| --- | --- | --- | --- | --- | --- | --- | --- | --- |
| Gender | (I) Age | (J) Age | Mean Difference (I-J) | Standard Error | Degrees of Freedom | P | 95% Wald Confidence Interval | |
|  |  |  |  |  |  |  | Lower Bound | Upper Bound |
| 1 | 1 | 2 | .6801 | .12430 | 1 | .000 | .4365 | .9238 |
|  |  | 3 | .0613 | .14886 | 1 | .681 | -.2305 | .3530 |
|  |  | 4 | .2885 | .16108 | 1 | .073 | -.0272 | .6042 |
|  | 2 | 1 | -.6801 | .12430 | 1 | .000 | -.9238 | -.4365 |
|  |  | 3 | -.6189 | .09754 | 1 | .000 | -.8101 | -.4277 |
|  |  | 4 | -.3917 | .11398 | 1 | .001 | -.6151 | -.1683 |
|  | 3 | 1 | -.0613 | .14886 | 1 | .681 | -.3530 | .2305 |
|  |  | 2 | .6189 | .09754 | 1 | .000 | .4277 | .8101 |
|  |  | 4 | .2272 | .13935 | 1 | .103 | -.0459 | .5003 |
|  | 4 | 1 | -.2885 | .16108 | 1 | .073 | -.6042 | .0272 |
|  |  | 2 | .3917 | .11398 | 1 | .001 | .1683 | .6151 |
|  |  | 3 | -.2272 | .13935 | 1 | .103 | -.5003 | .0459 |
| 2 | 1 | 2 | 1.3634 | .14427 | 1 | .000 | 1.0806 | 1.6461 |
|  |  | 3 | .9426 | .16537 | 1 | .000 | .6185 | 1.2667 |
|  |  | 4 | 1.2402 | .17801 | 1 | .000 | .8913 | 1.5891 |
|  | 2 | 1 | -1.3634 | .14427 | 1 | .000 | -1.6461 | -1.0806 |
|  |  | 3 | -.4208 | .09996 | 1 | .000 | -.6167 | -.2249 |
|  |  | 4 | -.1232 | .11711 | 1 | .293 | -.3527 | .1064 |
|  | 3 | 1 | -.9426 | .16537 | 1 | .000 | -1.2667 | -.6185 |
|  |  | 2 | .4208 | .09996 | 1 | .000 | .2249 | .6167 |
|  |  | 4 | .2977 | .14130 | 1 | .035 | .0207 | .5746 |
|  | 4 | 1 | -1.2402 | .17801 | 1 | .000 | -1.5891 | -.8913 |
|  |  | 2 | .1232 | .11711 | 1 | .293 | -.1064 | .3527 |
|  |  | 3 | -.2977 | .14130 | 1 | .035 | -.5746 | -.0207 |

| **Overall Test** | | | |
| --- | --- | --- | --- |
| Gender | Wald χ² | Degrees of Freedom | P |
| 1 | 68.008 | 3 | .000 |
| 2 | 98.955 | 3 | .000 |

**Estimated Marginal Means 13：Gender* Diagnostic**

| **Estimate** | | | | | |
| --- | --- | --- | --- | --- | --- |
| Gender | Diagnostic | Mean | Standard Error | 95% Wald Confidence Interval | |
|  |  |  |  | Lower Bound | Upper Bound |
| 1 | 1 | 9.4898 | .05076 | 9.3908 | 9.5898 |
|  | 2 | 9.3362 | .07590 | 9.1886 | 9.4861 |
| 2 | 1 | 9.7181 | .05836 | 9.6044 | 9.8331 |
|  | 2 | 9.4026 | .08288 | 9.2415 | 9.5664 |

| **Pairwise Comparisons** | | | | | | | | |
| --- | --- | --- | --- | --- | --- | --- | --- | --- |
| Diagnostic | (I) Gender | (J) Gender | Mean Difference (I-J) | Standard Error | Degrees of Freedom | P | 95% Wald Confidence Interval | |
|  |  |  |  |  |  |  | Lower Bound | Upper Bound |
| 1 | 1 | 2 | -.2283 | .06123 | 1 | .000 | -.3483 | -.1083 |
|  | 2 | 1 | .2283 | .06123 | 1 | .000 | .1083 | .3483 |
| 2 | 1 | 2 | -.0664 | .07018 | 1 | .344 | -.2039 | .0711 |
|  | 2 | 1 | .0664 | .07018 | 1 | .344 | -.0711 | .2039 |

| **Overall Test** | | | |
| --- | --- | --- | --- |
| Diagnostic | Wald χ² | Degrees of Freedom | P |
| 1 | 13.905 | 1 | .000 |
| 2 | .895 | 1 | .344 |

**Estimated Marginal Means 14：Gender* Diagnostic**

| **Estimate** | | | | | |
| --- | --- | --- | --- | --- | --- |
| Gender | Diagnostic | Mean | Standard Error | 95% Wald Confidence Interval | |
|  |  |  |  | Lower Bound | Upper Bound |
| 1 | 1 | 9.4898 | .05076 | 9.3908 | 9.5898 |
|  | 2 | 9.3362 | .07590 | 9.1886 | 9.4861 |
| 2 | 1 | 9.7181 | .05836 | 9.6044 | 9.8331 |
|  | 2 | 9.4026 | .08288 | 9.2415 | 9.5664 |

| **Pairwise Comparisons** | | | | | | | | |
| --- | --- | --- | --- | --- | --- | --- | --- | --- |
| Gender | (I) Diagnostic | (J) Diagnostic | Mean Difference (I-J) | Standard Error | Degrees of Freedom | P | 95% Wald Confidence Interval | |
|  |  |  |  |  |  |  | Lower Bound | Upper Bound |
| 1 | 1 | 2 | .1536 | .08277 | 1 | .063 | -.0086 | .3158 |
|  | 2 | 1 | -.1536 | .08277 | 1 | .063 | -.3158 | .0086 |
| 2 | 1 | 2 | .3155 | .08719 | 1 | .000 | .1446 | .4864 |
|  | 2 | 1 | -.3155 | .08719 | 1 | .000 | -.4864 | -.1446 |

| **Overall Test** | | | |
| --- | --- | --- | --- |
| Gender | Wald χ² | Degrees of Freedom | P |
| 1 | 3.444 | 1 | .063 |
| 2 | 13.094 | 1 | .000 |

**Estimated Marginal Means 15：Age* Diagnostic**

| **Estimate** | | | | | |
| --- | --- | --- | --- | --- | --- |
| Age | Diagnostic | Mean | Standard Error | 95% Wald Confidence Interval | |
|  |  |  |  | Lower Bound | Upper Bound |
| 1 | 1 | 11.1959 | .08714 | 11.0264 | 11.3680 |
|  | 2 | 9.0381 | .18176 | 8.6888 | 9.4014 |
| 2 | 1 | 9.2267 | .04253 | 9.1437 | 9.3104 |
|  | 2 | 8.8670 | .05657 | 8.7568 | 8.9786 |
| 3 | 1 | 9.2150 | .08690 | 9.0463 | 9.3870 |
|  | 2 | 9.9281 | .10554 | 9.7234 | 10.1372 |
| 4 | 1 | 8.9345 | .10753 | 8.7262 | 9.1477 |
|  | 2 | 9.6852 | .11646 | 9.4596 | 9.9162 |

| **Pairwise Comparisons** | | | | | | | | |
| --- | --- | --- | --- | --- | --- | --- | --- | --- |
| Diagnostic | (I) Age | (J) Age | Mean Difference (I-J) | Standard Error | Degrees of Freedom | P | 95% Wald Confidence Interval | |
|  |  |  |  |  |  |  | Lower Bound | Upper Bound |
| 1 | 1 | 2 | 1.9692 | .09677 | 1 | .000 | 1.7796 | 2.1589 |
|  |  | 3 | 1.9809 | .12294 | 1 | .000 | 1.7399 | 2.2219 |
|  |  | 4 | 2.2615 | .13815 | 1 | .000 | 1.9907 | 2.5322 |
|  | 2 | 1 | -1.9692 | .09677 | 1 | .000 | -2.1589 | -1.7796 |
|  |  | 3 | .0117 | .09319 | 1 | .900 | -.1710 | .1943 |
|  |  | 4 | .2922 | .11069 | 1 | .008 | .0753 | .5092 |
|  | 3 | 1 | -1.9809 | .12294 | 1 | .000 | -2.2219 | -1.7399 |
|  |  | 2 | -.0117 | .09319 | 1 | .900 | -.1943 | .1710 |
|  |  | 4 | .2806 | .13309 | 1 | .035 | .0197 | .5414 |
|  | 4 | 1 | -2.2615 | .13815 | 1 | .000 | -2.5322 | -1.9907 |
|  |  | 2 | -.2922 | .11069 | 1 | .008 | -.5092 | -.0753 |
|  |  | 3 | -.2806 | .13309 | 1 | .035 | -.5414 | -.0197 |
| 2 | 1 | 2 | .1710 | .17993 | 1 | .342 | -.1816 | .5237 |
|  |  | 3 | -.8901 | .19925 | 1 | .000 | -1.2806 | -.4995 |
|  |  | 4 | -.6471 | .20882 | 1 | .002 | -1.0564 | -.2378 |
|  | 2 | 1 | -.1710 | .17993 | 1 | .342 | -.5237 | .1816 |
|  |  | 3 | -1.0611 | .10502 | 1 | .000 | -1.2669 | -.8553 |
|  |  | 4 | -.8181 | .12057 | 1 | .000 | -1.0545 | -.5818 |
|  | 3 | 1 | .8901 | .19925 | 1 | .000 | .4995 | 1.2806 |
|  |  | 2 | 1.0611 | .10502 | 1 | .000 | .8553 | 1.2669 |
|  |  | 4 | .2430 | .14846 | 1 | .102 | -.0480 | .5339 |
|  | 4 | 1 | .6471 | .20882 | 1 | .002 | .2378 | 1.0564 |
|  |  | 2 | .8181 | .12057 | 1 | .000 | .5818 | 1.0545 |
|  |  | 3 | -.2430 | .14846 | 1 | .102 | -.5339 | .0480 |

| **Overall Test** | | | |
| --- | --- | --- | --- |
| Diagnostic | Wald χ² | Degrees of Freedom | P |
| 1 | 447.741 | 3 | .000 |
| 2 | 131.742 | 3 | .000 |

**Estimated Marginal Means 16：Age* Diagnostic**

| **Estimate** | | | | | |
| --- | --- | --- | --- | --- | --- |
| Age | Diagnostic | Mean | Standard Error | 95% Wald Confidence Interval | |
|  |  |  |  | Lower Bound | Upper Bound |
| 1 | 1 | 11.1959 | .08714 | 11.0264 | 11.3680 |
|  | 2 | 9.0381 | .18176 | 8.6888 | 9.4014 |
| 2 | 1 | 9.2267 | .04253 | 9.1437 | 9.3104 |
|  | 2 | 8.8670 | .05657 | 8.7568 | 8.9786 |
| 3 | 1 | 9.2150 | .08690 | 9.0463 | 9.3870 |
|  | 2 | 9.9281 | .10554 | 9.7234 | 10.1372 |
| 4 | 1 | 8.9345 | .10753 | 8.7262 | 9.1477 |
|  | 2 | 9.6852 | .11646 | 9.4596 | 9.9162 |

| **Pairwise Comparisons** | | | | | | | | |
| --- | --- | --- | --- | --- | --- | --- | --- | --- |
| Age | (I) Diagnostic | (J) Diagnostic | Mean Difference (I-J) | Standard Error | Degrees of Freedom | P | 95% Wald Confidence Interval | |
|  |  |  |  |  |  |  | Lower Bound | Upper Bound |
| 1 | 1 | 2 | 2.1579 | .19682 | 1 | .000 | 1.7721 | 2.5436 |
|  | 2 | 1 | -2.1579 | .19682 | 1 | .000 | -2.5436 | -1.7721 |
| 2 | 1 | 2 | .3597 | .06681 | 1 | .000 | .2287 | .4906 |
|  | 2 | 1 | -.3597 | .06681 | 1 | .000 | -.4906 | -.2287 |
| 3 | 1 | 2 | -.7131 | .08561 | 1 | .000 | -.8809 | -.5453 |
|  | 2 | 1 | .7131 | .08561 | 1 | .000 | .5453 | .8809 |
| 4 | 1 | 2 | -.7507 | .09167 | 1 | .000 | -.9304 | -.5710 |
|  | 2 | 1 | .7507 | .09167 | 1 | .000 | .5710 | .9304 |

| **Overall Test** | | | |
| --- | --- | --- | --- |
| Age | Wald χ² | Degrees of Freedom | P |
| 1 | 120.201 | 1 | .000 |
| 2 | 28.979 | 1 | .000 |
| 3 | 69.381 | 1 | .000 |
| 4 | 67.058 | 1 | .000 |
