# Supplementary material for: The impact of COVID-19 social isolation and reduced microbial exposure on the immune system in children: a retrospective study
Source: PeerJ. 2026 Jul 7;14:e21469. doi: 10.7717/peerj.21469 (PMC13353229; doi:10.7717/peerj.21469)
Supplement: Supplemental Information 4 [file peerj-14-21469-s004.docx]

**Neutrophils Generalized Linear Model**

For CBC analysis, patients were categorized into the following age groups:
Group 1: 0–3 months,
Group 2: 3 months–4 years,
Group 3: 4–6 years,
Group 4: 6–13 years.

Diagnostic 1 is LRTIs . Diagnostic 2 URTIs .

| **Parameter Estimate** | | | | | | | |
| --- | --- | --- | --- | --- | --- | --- | --- |
| Parameter | B | Standard Error | 95% Wald Confidence Interval | | Hypothesis Testing | | |
|  |  |  | Lower Bound | Upper Bound | Wald χ² | Degrees of Freedom | P |
| （Intercept） | 1.774 | .0411 | 1.693 | 1.854 | 1862.646 | 1 | .000 |
| [Year=2020] | .162 | .0972 | -.029 | .352 | 2.759 | 1 | .097 |
| [Year=2021] | .175 | .0540 | .069 | .281 | 10.482 | 1 | .001 |
| [Year=2022] | .002 | .0475 | -.091 | .096 | .003 | 1 | .958 |
| [Year=2023] | .049 | .0411 | -.032 | .129 | 1.406 | 1 | .236 |
| [Year=2024] | .264 | .0412 | .183 | .345 | 41.092 | 1 | .000 |
| [Year=2025] | 0 | . | . | . | . | . | . |
| [Male ] | -.024 | .0223 | -.068 | .019 | 1.183 | 1 | .277 |
| [Female ] | 0 | . | . | . | . | . | . |
| [Age=1] | -.305 | .0522 | -.407 | -.202 | 34.027 | 1 | .000 |
| [Age=2] | -.300 | .0398 | -.378 | -.222 | 56.667 | 1 | .000 |
| [Age=3] | -.056 | .0468 | -.148 | .035 | 1.453 | 1 | .228 |
| [Age=4] | 0 | . | . | . | . | . | . |
| [LRTIs ] | -.251 | .0244 | -.299 | -.203 | 105.514 | 1 | .000 |
| [URTIs ] | 0 | . | . | . | . | . | . |
| [Year=2020] * [Male ] | -.018 | .0426 | -.101 | .066 | .177 | 1 | .674 |
| [Year=2020] * [Female ] | 0 | . | . | . | . | . | . |
| [Year=2021] * [Male ] | .070 | .0283 | .014 | .125 | 6.078 | 1 | .014 |
| [Year=2021] * [Female ] | 0 | . | . | . | . | . | . |
| [Year=2022] * [Male ] | .073 | .0230 | .028 | .118 | 10.016 | 1 | .002 |
| [Year=2022] * [Female ] | 0 | . | . | . | . | . | . |
| [Year=2023] * [Male ] | .064 | .0198 | .025 | .102 | 10.284 | 1 | .001 |
| [Year=2023] * [Female ] | 0 | . | . | . | . | . | . |
| [Year=2024] * [Male ] | .050 | .0196 | .011 | .088 | 6.383 | 1 | .012 |
| [Year=2024] * [Female ] | 0 | . | . | . | . | . | . |
| [Year=2025] * [Male ] | 0 | . | . | . | . | . | . |
| [Year=2025] * [Female ] | 0 | . | . | . | . | . | . |
| [Year=2020] * [Age=1] | -.264 | .0997 | -.459 | -.069 | 7.015 | 1 | .008 |
| [Year=2020] * [Age=2] | -.287 | .0943 | -.472 | -.103 | 9.289 | 1 | .002 |
| [Year=2020] * [Age=3] | .059 | .1164 | -.170 | .287 | .253 | 1 | .615 |
| [Year=2020] * [Age=4] | 0 | . | . | . | . | . | . |
| [Year=2021] * [Age=1] | .121 | .0636 | -.004 | .246 | 3.610 | 1 | .057 |
| [Year=2021] * [Age=2] | .025 | .0520 | -.077 | .127 | .231 | 1 | .631 |
| [Year=2021] * [Age=3] | .023 | .0602 | -.095 | .141 | .151 | 1 | .698 |
| [Year=2021] * [Age=4] | 0 | . | . | . | . | . | . |
| [Year=2022] * [Age=1] | -.287 | .0556 | -.396 | -.178 | 26.608 | 1 | .000 |
| [Year=2022] * [Age=2] | .072 | .0460 | -.018 | .162 | 2.466 | 1 | .116 |
| [Year=2022] * [Age=3] | .085 | .0529 | -.019 | .189 | 2.582 | 1 | .108 |
| [Year=2022] * [Age=4] | 0 | . | . | . | . | . | . |
| [Year=2023] * [Age=1] | -.405 | .0469 | -.497 | -.313 | 74.700 | 1 | .000 |
| [Year=2023] * [Age=2] | .099 | .0396 | .022 | .177 | 6.273 | 1 | .012 |
| [Year=2023] * [Age=3] | .075 | .0465 | -.016 | .166 | 2.588 | 1 | .108 |
| [Year=2023] * [Age=4] | 0 | . | . | . | . | . | . |
| [Year=2024] * [Age=1] | -.653 | .0476 | -.746 | -.560 | 188.124 | 1 | .000 |
| [Year=2024] * [Age=2] | -.075 | .0396 | -.153 | .002 | 3.601 | 1 | .058 |
| [Year=2024] * [Age=3] | .034 | .0466 | -.058 | .125 | .523 | 1 | .470 |
| [Year=2024] * [Age=4] | 0 | . | . | . | . | . | . |
| [Year=2025] * [Age=1] | 0 | . | . | . | . | . | . |
| [Year=2025] * [Age=2] | 0 | . | . | . | . | . | . |
| [Year=2025] * [Age=3] | 0 | . | . | . | . | . | . |
| [Year=2025] * [Age=4] | 0 | . | . | . | . | . | . |
| [Year=2020] * [LRTIs ] | .185 | .0548 | .078 | .292 | 11.412 | 1 | .001 |
| [Year=2020] * [URTIs ] | 0 | . | . | . | . | . | . |
| [Year=2021] * [LRTIs ] | .003 | .0310 | -.058 | .063 | .007 | 1 | .932 |
| [Year=2021] * [URTIs ] | 0 | . | . | . | . | . | . |
| [Year=2022] * [LRTIs ] | .113 | .0257 | .062 | .163 | 19.277 | 1 | .000 |
| [Year=2022] * [URTIs ] | 0 | . | . | . | . | . | . |
| [Year=2023] * [LRTIs ] | .107 | .0225 | .063 | .151 | 22.494 | 1 | .000 |
| [Year=2023] * [URTIs ] | 0 | . | . | . | . | . | . |
| [Year=2024] * [LRTIs ] | -.008 | .0223 | -.052 | .036 | .130 | 1 | .719 |
| [Year=2024] * [URTIs ] | 0 | . | . | . | . | . | . |
| [Year=2025] * [LRTIs ] | 0 | . | . | . | . | . | . |
| [Year=2025] * [URTIs ] | 0 | . | . | . | . | . | . |
| [Male ] * [Age=1] | -.112 | .0233 | -.157 | -.066 | 23.024 | 1 | .000 |
| [Male ] * [Age=2] | -.044 | .0125 | -.069 | -.020 | 12.410 | 1 | .000 |
| [Male ] * [Age=3] | .007 | .0134 | -.019 | .033 | .276 | 1 | .600 |
| [Male ] * [Age=4] | 0 | . | . | . | . | . | . |
| [Female ] * [Age=1] | 0 | . | . | . | . | . | . |
| [Female ] * [Age=2] | 0 | . | . | . | . | . | . |
| [Female ] * [Age=3] | 0 | . | . | . | . | . | . |
| [Female ] * [Age=4] | 0 | . | . | . | . | . | . |
| [Male ] * [LRTIs ] | -.029 | .0094 | -.048 | -.011 | 9.844 | 1 | .002 |
| [Male ] * [URTIs ] | 0 | . | . | . | . | . | . |
| [Female ] * [LRTIs ] | 0 | . | . | . | . | . | . |
| [Female ] * [URTIs ] | 0 | . | . | . | . | . | . |
| [Age=1] * [LRTIs ] | .597 | .0323 | .533 | .660 | 342.007 | 1 | .000 |
| [Age=1] * [URTIs ] | 0 | . | . | . | . | . | . |
| [Age=2] * [LRTIs ] | .067 | .0127 | .043 | .092 | 28.283 | 1 | .000 |
| [Age=2] * [URTIs ] | 0 | . | . | . | . | . | . |
| [Age=3] * [LRTIs ] | -.015 | .0137 | -.042 | .012 | 1.215 | 1 | .270 |
| [Age=3] * [URTIs ] | 0 | . | . | . | . | . | . |
| [Age=4] * [LRTIs ] | 0 | . | . | . | . | . | . |
| [Age=4] * [URTIs ] | 0 | . | . | . | . | . | . |
| （标度） | .401 | .0019 | .397 | .404 |  |  |  |

**Estimated Marginal Means 1：Year**

| **Estimate** | | | | |
| --- | --- | --- | --- | --- |
| Year | Mean | Standard Error | 95% Wald Confidence Interval | |
|  |  |  | Lower Bound | Upper Bound |
| 2020 | 5.1975 | .16100 | 4.8913 | 5.5228 |
| 2021 | 5.9285 | .08732 | 5.7598 | 6.1021 |
| 2022 | 4.8997 | .05223 | 4.7984 | 5.0031 |
| 2023 | 4.9647 | .03172 | 4.9029 | 5.0273 |
| 2024 | 5.1418 | .03378 | 5.0760 | 5.2085 |
| 2025 | 4.6012 | .06331 | 4.4788 | 4.7270 |

| **Pairwise Comparisons** | | | | | | | |
| --- | --- | --- | --- | --- | --- | --- | --- |
| (I) Year | (J) Year | Mean Difference (I-J) | Standard Error | Degrees of Freedom | P | 95% Wald Confidence Interval | |
|  |  |  |  |  |  | Lower Bound | Upper Bound |
| 2020 | 2021 | -.7310 | .18125 | 1 | .000 | -1.0863 | -.3758 |
|  | 2022 | .2978 | .16806 | 1 | .076 | -.0316 | .6272 |
|  | 2023 | .2328 | .16299 | 1 | .153 | -.0867 | .5522 |
|  | 2024 | .0557 | .16350 | 1 | .734 | -.2648 | .3761 |
|  | 2025 | .5963 | .17134 | 1 | .001 | .2605 | .9321 |
| 2021 | 2020 | .7310 | .18125 | 1 | .000 | .3758 | 1.0863 |
|  | 2022 | 1.0288 | .09943 | 1 | .000 | .8339 | 1.2237 |
|  | 2023 | .9638 | .09060 | 1 | .000 | .7862 | 1.1414 |
|  | 2024 | .7867 | .09150 | 1 | .000 | .6073 | .9660 |
|  | 2025 | 1.3273 | .10493 | 1 | .000 | 1.1216 | 1.5330 |
| 2022 | 2020 | -.2978 | .16806 | 1 | .076 | -.6272 | .0316 |
|  | 2021 | -1.0288 | .09943 | 1 | .000 | -1.2237 | -.8339 |
|  | 2023 | -.0650 | .05891 | 1 | .270 | -.1805 | .0504 |
|  | 2024 | -.2421 | .06013 | 1 | .000 | -.3600 | -.1243 |
|  | 2025 | .2985 | .07979 | 1 | .000 | .1421 | .4549 |
| 2023 | 2020 | -.2328 | .16299 | 1 | .153 | -.5522 | .0867 |
|  | 2021 | -.9638 | .09060 | 1 | .000 | -1.1414 | -.7862 |
|  | 2022 | .0650 | .05891 | 1 | .270 | -.0504 | .1805 |
|  | 2024 | -.1771 | .04381 | 1 | .000 | -.2630 | -.0912 |
|  | 2025 | .3635 | .06841 | 1 | .000 | .2294 | .4976 |
| 2024 | 2020 | -.0557 | .16350 | 1 | .734 | -.3761 | .2648 |
|  | 2021 | -.7867 | .09150 | 1 | .000 | -.9660 | -.6073 |
|  | 2022 | .2421 | .06013 | 1 | .000 | .1243 | .3600 |
|  | 2023 | .1771 | .04381 | 1 | .000 | .0912 | .2630 |
|  | 2025 | .5406 | .06956 | 1 | .000 | .4043 | .6770 |
| 2025 | 2020 | -.5963 | .17134 | 1 | .001 | -.9321 | -.2605 |
|  | 2021 | -1.3273 | .10493 | 1 | .000 | -1.5330 | -1.1216 |
|  | 2022 | -.2985 | .07979 | 1 | .000 | -.4549 | -.1421 |
|  | 2023 | -.3635 | .06841 | 1 | .000 | -.4976 | -.2294 |
|  | 2024 | -.5406 | .06956 | 1 | .000 | -.6770 | -.4043 |

| **Overall Test** | | |
| --- | --- | --- |
| Wald χ² | Degrees of Freedom | P |
| 184.883 | 5 | .000 |

**Estimated Marginal Means 2：Gender**

| **Estimate** | | | | |
| --- | --- | --- | --- | --- |
| Gender | Mean | Standard Error | 95% Wald Confidence Interval | |
|  |  |  | Lower Bound | Upper Bound |
| 1 | 5.0143 | .03951 | 4.9375 | 5.0924 |
| 2 | 5.2005 | .04654 | 5.1101 | 5.2926 |

| **Pairwise Comparisons** | | | | | | | |
| --- | --- | --- | --- | --- | --- | --- | --- |
| (I) Gender | (J) Gender | Mean Difference (I-J) | Standard Error | Degrees of Freedom | P | 95% Wald Confidence Interval | |
|  |  |  |  |  |  | Lower Bound | Upper Bound |
| 1 | 2 | -.1862 | .04733 | 1 | .000 | -.2790 | -.0934 |
| 2 | 1 | .1862 | .04733 | 1 | .000 | .0934 | .2790 |

| **Overall Test** | | |
| --- | --- | --- |
| Wald χ² | Degrees of Freedom | P |
| 15.479 | 1 | .000 |

**Estimated Marginal Means 3：Age**

| **Estimate** | | | | |
| --- | --- | --- | --- | --- |
| Age | Mean | Standard Error | 95% Wald Confidence Interval | |
|  |  |  | Lower Bound | Upper Bound |
| 1 | 4.3949 | .07232 | 4.2554 | 4.5389 |
| 2 | 4.3706 | .02695 | 4.3181 | 4.4238 |
| 3 | 5.9069 | .07956 | 5.7530 | 6.0649 |
| 4 | 5.9934 | .09818 | 5.8040 | 6.1890 |

| **Pairwise Comparisons** | | | | | | | |
| --- | --- | --- | --- | --- | --- | --- | --- |
| (I) Age | (J) Age | Mean Difference (I-J) | Standard Error | Degrees of Freedom | P | 95% Wald Confidence Interval | |
|  |  |  |  |  |  | Lower Bound | Upper Bound |
| 1 | 2 | .0242 | .07519 | 1 | .747 | -.1231 | .1716 |
|  | 3 | -1.5120 | .10576 | 1 | .000 | -1.7193 | -1.3047 |
|  | 4 | -1.5985 | .12209 | 1 | .000 | -1.8378 | -1.3592 |
| 2 | 1 | -.0242 | .07519 | 1 | .747 | -.1716 | .1231 |
|  | 3 | -1.5362 | .08285 | 1 | .000 | -1.6986 | -1.3738 |
|  | 4 | -1.6228 | .10169 | 1 | .000 | -1.8221 | -1.4235 |
| 3 | 1 | 1.5120 | .10576 | 1 | .000 | 1.3047 | 1.7193 |
|  | 2 | 1.5362 | .08285 | 1 | .000 | 1.3738 | 1.6986 |
|  | 4 | -.0866 | .12625 | 1 | .493 | -.3340 | .1609 |
| 4 | 1 | 1.5985 | .12209 | 1 | .000 | 1.3592 | 1.8378 |
|  | 2 | 1.6228 | .10169 | 1 | .000 | 1.4235 | 1.8221 |
|  | 3 | .0866 | .12625 | 1 | .493 | -.1609 | .3340 |

| **Overall Test** | | |
| --- | --- | --- |
| Wald χ² | Degrees of Freedom | P |
| 563.860 | 3 | .000 |

**Estimated Marginal Means 4：Diagnostic**

| **Estimate** | | | | |
| --- | --- | --- | --- | --- |
| Diagnostic | Mean | Standard Error | 95% Wald Confidence Interval | |
|  |  |  | Lower Bound | Upper Bound |
| 1 | 5.0136 | .03509 | 4.9453 | 5.0828 |
| 2 | 5.2013 | .05919 | 5.0866 | 5.3186 |

| **Pairwise Comparisons** | | | | | | | |
| --- | --- | --- | --- | --- | --- | --- | --- |
| (I) Diagnostic | (J) Diagnostic | Mean Difference (I-J) | Standard Error | Degrees of Freedom | P | 95% Wald Confidence Interval | |
|  |  |  |  |  |  | Lower Bound | Upper Bound |
| 1 | 2 | -.1877 | .06478 | 1 | .004 | -.3147 | -.0608 |
| 2 | 1 | .1877 | .06478 | 1 | .004 | .0608 | .3147 |

| **Overall Test** | | |
| --- | --- | --- |
| Wald χ² | Degrees of Freedom | P |
| 8.399 | 1 | .004 |

**Estimated Marginal Means 5：Year* Gender**

| **Estimate** | | | | | |
| --- | --- | --- | --- | --- | --- |
| Year | Gender | Mean | Standard Error | 95% Wald Confidence Interval | |
|  |  |  |  | Lower Bound | Upper Bound |
| 2020 | 1 | 4.9587 | .16620 | 4.6434 | 5.2954 |
|  | 2 | 5.4477 | .21528 | 5.0417 | 5.8864 |
| 2021 | 1 | 5.9099 | .10367 | 5.7102 | 6.1166 |
|  | 2 | 5.9471 | .11554 | 5.7249 | 6.1779 |
| 2022 | 1 | 4.8917 | .06056 | 4.7745 | 5.0119 |
|  | 2 | 4.9076 | .06748 | 4.7771 | 5.0417 |
| 2023 | 1 | 4.9336 | .03681 | 4.8620 | 5.0063 |
|  | 2 | 4.9960 | .04023 | 4.9178 | 5.0755 |
| 2024 | 1 | 5.0741 | .03793 | 5.0003 | 5.1490 |
|  | 2 | 5.2104 | .04312 | 5.1266 | 5.2956 |
| 2025 | 1 | 4.4293 | .06914 | 4.2959 | 4.5670 |
|  | 2 | 4.7797 | .08201 | 4.6216 | 4.9432 |

| **Pairwise Comparisons** | | | | | | | | |
| --- | --- | --- | --- | --- | --- | --- | --- | --- |
| Gender | (I) Year | (J) Year | Mean Difference (I-J) | Standard Error | Degrees of Freedom | P | 95% Wald Confidence Interval | |
|  |  |  |  |  |  |  | Lower Bound | Upper Bound |
| 1 | 2020 | 2021 | -.9512 | .19418 | 1 | .000 | -1.3318 | -.5706 |
|  |  | 2022 | .0669 | .17566 | 1 | .703 | -.2773 | .4112 |
|  |  | 2023 | .0251 | .16923 | 1 | .882 | -.3066 | .3568 |
|  |  | 2024 | -.1154 | .16949 | 1 | .496 | -.4476 | .2168 |
|  |  | 2025 | .5294 | .17834 | 1 | .003 | .1798 | .8789 |
|  | 2021 | 2020 | .9512 | .19418 | 1 | .000 | .5706 | 1.3318 |
|  |  | 2022 | 1.0182 | .11712 | 1 | .000 | .7886 | 1.2477 |
|  |  | 2023 | .9763 | .10708 | 1 | .000 | .7664 | 1.1862 |
|  |  | 2024 | .8358 | .10757 | 1 | .000 | .6250 | 1.0466 |
|  |  | 2025 | 1.4806 | .12181 | 1 | .000 | 1.2418 | 1.7193 |
|  | 2022 | 2020 | -.0669 | .17566 | 1 | .703 | -.4112 | .2773 |
|  |  | 2021 | -1.0182 | .11712 | 1 | .000 | -1.2477 | -.7886 |
|  |  | 2023 | -.0418 | .06768 | 1 | .536 | -.1745 | .0908 |
|  |  | 2024 | -.1824 | .06835 | 1 | .008 | -.3163 | -.0484 |
|  |  | 2025 | .4624 | .08935 | 1 | .000 | .2873 | .6375 |
|  | 2023 | 2020 | -.0251 | .16923 | 1 | .882 | -.3568 | .3066 |
|  |  | 2021 | -.9763 | .10708 | 1 | .000 | -1.1862 | -.7664 |
|  |  | 2022 | .0418 | .06768 | 1 | .536 | -.0908 | .1745 |
|  |  | 2024 | -.1405 | .04871 | 1 | .004 | -.2360 | -.0450 |
|  |  | 2025 | .5043 | .07593 | 1 | .000 | .3554 | .6531 |
|  | 2024 | 2020 | .1154 | .16949 | 1 | .496 | -.2168 | .4476 |
|  |  | 2021 | -.8358 | .10757 | 1 | .000 | -1.0466 | -.6250 |
|  |  | 2022 | .1824 | .06835 | 1 | .008 | .0484 | .3163 |
|  |  | 2023 | .1405 | .04871 | 1 | .004 | .0450 | .2360 |
|  |  | 2025 | .6448 | .07648 | 1 | .000 | .4949 | .7947 |
|  | 2025 | 2020 | -.5294 | .17834 | 1 | .003 | -.8789 | -.1798 |
|  |  | 2021 | -1.4806 | .12181 | 1 | .000 | -1.7193 | -1.2418 |
|  |  | 2022 | -.4624 | .08935 | 1 | .000 | -.6375 | -.2873 |
|  |  | 2023 | -.5043 | .07593 | 1 | .000 | -.6531 | -.3554 |
|  |  | 2024 | -.6448 | .07648 | 1 | .000 | -.7947 | -.4949 |
| 2 | 2020 | 2021 | -.4994 | .24261 | 1 | .040 | -.9749 | -.0239 |
|  |  | 2022 | .5401 | .22468 | 1 | .016 | .0998 | .9805 |
|  |  | 2023 | .4517 | .21854 | 1 | .039 | .0234 | .8800 |
|  |  | 2024 | .2373 | .21908 | 1 | .279 | -.1921 | .6667 |
|  |  | 2025 | .6680 | .22812 | 1 | .003 | .2209 | 1.1151 |
|  | 2021 | 2020 | .4994 | .24261 | 1 | .040 | .0239 | .9749 |
|  |  | 2022 | 1.0395 | .13066 | 1 | .000 | .7834 | 1.2956 |
|  |  | 2023 | .9511 | .11937 | 1 | .000 | .7171 | 1.1851 |
|  |  | 2024 | .7367 | .12013 | 1 | .000 | .5013 | .9721 |
|  |  | 2025 | 1.1674 | .13879 | 1 | .000 | .8954 | 1.4395 |
|  | 2022 | 2020 | -.5401 | .22468 | 1 | .016 | -.9805 | -.0998 |
|  |  | 2021 | -1.0395 | .13066 | 1 | .000 | -1.2956 | -.7834 |
|  |  | 2023 | -.0884 | .07463 | 1 | .236 | -.2347 | .0578 |
|  |  | 2024 | -.3028 | .07575 | 1 | .000 | -.4513 | -.1544 |
|  |  | 2025 | .1279 | .10391 | 1 | .218 | -.0758 | .3316 |
|  | 2023 | 2020 | -.4517 | .21854 | 1 | .039 | -.8800 | -.0234 |
|  |  | 2021 | -.9511 | .11937 | 1 | .000 | -1.1851 | -.7171 |
|  |  | 2022 | .0884 | .07463 | 1 | .236 | -.0578 | .2347 |
|  |  | 2024 | -.2144 | .05317 | 1 | .000 | -.3186 | -.1102 |
|  |  | 2025 | .2163 | .08946 | 1 | .016 | .0410 | .3917 |
|  | 2024 | 2020 | -.2373 | .21908 | 1 | .279 | -.6667 | .1921 |
|  |  | 2021 | -.7367 | .12013 | 1 | .000 | -.9721 | -.5013 |
|  |  | 2022 | .3028 | .07575 | 1 | .000 | .1544 | .4513 |
|  |  | 2023 | .2144 | .05317 | 1 | .000 | .1102 | .3186 |
|  |  | 2025 | .4307 | .09069 | 1 | .000 | .2530 | .6085 |
|  | 2025 | 2020 | -.6680 | .22812 | 1 | .003 | -1.1151 | -.2209 |
|  |  | 2021 | -1.1674 | .13879 | 1 | .000 | -1.4395 | -.8954 |
|  |  | 2022 | -.1279 | .10391 | 1 | .218 | -.3316 | .0758 |
|  |  | 2023 | -.2163 | .08946 | 1 | .016 | -.3917 | -.0410 |
|  |  | 2024 | -.4307 | .09069 | 1 | .000 | -.6085 | -.2530 |

| **Overall Test** | | | |
| --- | --- | --- | --- |
| Gender | Wald χ² | Degrees of Freedom | P |
| 1 | 161.756 | 5 | .000 |
| 2 | 99.362 | 5 | .000 |

**Estimated Marginal Means 6：Year* Gender**

| **Estimate** | | | | | |
| --- | --- | --- | --- | --- | --- |
| Year | Gender | Mean | Standard Error | 95% Wald Confidence Interval | |
|  |  |  |  | Lower Bound | Upper Bound |
| 2020 | 1 | 4.9587 | .16620 | 4.6434 | 5.2954 |
|  | 2 | 5.4477 | .21528 | 5.0417 | 5.8864 |
| 2021 | 1 | 5.9099 | .10367 | 5.7102 | 6.1166 |
|  | 2 | 5.9471 | .11554 | 5.7249 | 6.1779 |
| 2022 | 1 | 4.8917 | .06056 | 4.7745 | 5.0119 |
|  | 2 | 4.9076 | .06748 | 4.7771 | 5.0417 |
| 2023 | 1 | 4.9336 | .03681 | 4.8620 | 5.0063 |
|  | 2 | 4.9960 | .04023 | 4.9178 | 5.0755 |
| 2024 | 1 | 5.0741 | .03793 | 5.0003 | 5.1490 |
|  | 2 | 5.2104 | .04312 | 5.1266 | 5.2956 |
| 2025 | 1 | 4.4293 | .06914 | 4.2959 | 4.5670 |
|  | 2 | 4.7797 | .08201 | 4.6216 | 4.9432 |

| **Pairwise Comparisons** | | | | | | | | |
| --- | --- | --- | --- | --- | --- | --- | --- | --- |
| Year | (I) Gender | (J) Gender | Mean Difference (I-J) | Standard Error | Degrees of Freedom | P | 95% Wald Confidence Interval | |
|  |  |  |  |  |  |  | Lower Bound | Upper Bound |
| 2020 | 1 | 2 | -.4890 | .20691 | 1 | .018 | -.8946 | -.0835 |
|  | 2 | 1 | .4890 | .20691 | 1 | .018 | .0835 | .8946 |
| 2021 | 1 | 2 | -.0372 | .13296 | 1 | .780 | -.2978 | .2234 |
|  | 2 | 1 | .0372 | .13296 | 1 | .780 | -.2234 | .2978 |
| 2022 | 1 | 2 | -.0158 | .07434 | 1 | .831 | -.1615 | .1299 |
|  | 2 | 1 | .0158 | .07434 | 1 | .831 | -.1299 | .1615 |
| 2023 | 1 | 2 | -.0624 | .04380 | 1 | .154 | -.1483 | .0234 |
|  | 2 | 1 | .0624 | .04380 | 1 | .154 | -.0234 | .1483 |
| 2024 | 1 | 2 | -.1363 | .04495 | 1 | .002 | -.2244 | -.0482 |
|  | 2 | 1 | .1363 | .04495 | 1 | .002 | .0482 | .2244 |
| 2025 | 1 | 2 | -.3504 | .08284 | 1 | .000 | -.5127 | -.1880 |
|  | 2 | 1 | .3504 | .08284 | 1 | .000 | .1880 | .5127 |

| **Overall Test** | | | |
| --- | --- | --- | --- |
| Year | Wald χ² | Degrees of Freedom | P |
| 2020 | 5.586 | 1 | .018 |
| 2021 | .078 | 1 | .780 |
| 2022 | .045 | 1 | .831 |
| 2023 | 2.032 | 1 | .154 |
| 2024 | 9.198 | 1 | .002 |
| 2025 | 17.886 | 1 | .000 |

**Estimated Marginal Means 7：Year* Age**

| **Estimate** | | | | | |
| --- | --- | --- | --- | --- | --- |
| Year | Age | Mean | Standard Error | 95% Wald Confidence Interval | |
|  |  |  |  | Lower Bound | Upper Bound |
| 2020 | 1 | 4.7014 | .19866 | 4.3277 | 5.1073 |
|  | 2 | 3.6642 | .10683 | 3.4607 | 3.8797 |
|  | 3 | 6.5022 | .45619 | 5.6668 | 7.4607 |
|  | 4 | 6.5148 | .52860 | 5.5569 | 7.6377 |
| 2021 | 1 | 6.6780 | .26185 | 6.1840 | 7.2114 |
|  | 2 | 4.8407 | .07037 | 4.7047 | 4.9806 |
|  | 3 | 6.0682 | .14252 | 5.7952 | 6.3541 |
|  | 4 | 6.2974 | .20147 | 5.9146 | 6.7049 |
| 2022 | 1 | 3.9557 | .12140 | 3.7248 | 4.2010 |
|  | 2 | 4.5196 | .04314 | 4.4358 | 4.6049 |
|  | 3 | 5.7480 | .07953 | 5.5942 | 5.9060 |
|  | 4 | 5.6083 | .13142 | 5.3565 | 5.8718 |
| 2023 | 1 | 3.6524 | .08251 | 3.4942 | 3.8177 |
|  | 2 | 4.8256 | .02802 | 4.7710 | 4.8808 |
|  | 3 | 5.9135 | .03952 | 5.8366 | 5.9915 |
|  | 4 | 5.8291 | .04411 | 5.7433 | 5.9162 |
| 2024 | 1 | 3.3155 | .07741 | 3.1672 | 3.4708 |
|  | 2 | 4.7137 | .02463 | 4.6657 | 4.7623 |
|  | 3 | 6.5986 | .04281 | 6.5153 | 6.6831 |
|  | 4 | 6.7779 | .05505 | 6.6708 | 6.8866 |
| 2025 | 1 | 4.7913 | .11432 | 4.5724 | 5.0207 |
|  | 2 | 3.8226 | .04636 | 3.7329 | 3.9146 |
|  | 3 | 4.7996 | .12978 | 4.5518 | 5.0608 |
|  | 4 | 5.0987 | .18729 | 4.7446 | 5.4793 |

| **Pairwise Comparisons** | | | | | | | | |
| --- | --- | --- | --- | --- | --- | --- | --- | --- |
| Age | (I) Year | (J) Year | Mean Difference (I-J) | Standard Error | Degrees of Freedom | P | 95% Wald Confidence Interval | |
|  |  |  |  |  |  |  | Lower Bound | Upper Bound |
| 1 | 2020 | 2021 | -1.9766 | .31060 | 1 | .000 | -2.5854 | -1.3679 |
|  |  | 2022 | .7456 | .22145 | 1 | .001 | .3116 | 1.1797 |
|  |  | 2023 | 1.0490 | .20503 | 1 | .000 | .6471 | 1.4508 |
|  |  | 2024 | 1.3858 | .20485 | 1 | .000 | .9843 | 1.7873 |
|  |  | 2025 | -.0899 | .20905 | 1 | .667 | -.4997 | .3198 |
|  | 2021 | 2020 | 1.9766 | .31060 | 1 | .000 | 1.3679 | 2.5854 |
|  |  | 2022 | 2.7223 | .27622 | 1 | .000 | 2.1809 | 3.2636 |
|  |  | 2023 | 3.0256 | .26384 | 1 | .000 | 2.5085 | 3.5427 |
|  |  | 2024 | 3.3624 | .26423 | 1 | .000 | 2.8446 | 3.8803 |
|  |  | 2025 | 1.8867 | .26394 | 1 | .000 | 1.3694 | 2.4040 |
|  | 2022 | 2020 | -.7456 | .22145 | 1 | .001 | -1.1797 | -.3116 |
|  |  | 2021 | -2.7223 | .27622 | 1 | .000 | -3.2636 | -2.1809 |
|  |  | 2023 | .3033 | .13751 | 1 | .027 | .0338 | .5729 |
|  |  | 2024 | .6402 | .13606 | 1 | .000 | .3735 | .9069 |
|  |  | 2025 | -.8356 | .14990 | 1 | .000 | -1.1293 | -.5418 |
|  | 2023 | 2020 | -1.0490 | .20503 | 1 | .000 | -1.4508 | -.6471 |
|  |  | 2021 | -3.0256 | .26384 | 1 | .000 | -3.5427 | -2.5085 |
|  |  | 2022 | -.3033 | .13751 | 1 | .027 | -.5729 | -.0338 |
|  |  | 2024 | .3368 | .10484 | 1 | .001 | .1314 | .5423 |
|  |  | 2025 | -1.1389 | .12449 | 1 | .000 | -1.3829 | -.8949 |
|  | 2024 | 2020 | -1.3858 | .20485 | 1 | .000 | -1.7873 | -.9843 |
|  |  | 2021 | -3.3624 | .26423 | 1 | .000 | -3.8803 | -2.8446 |
|  |  | 2022 | -.6402 | .13606 | 1 | .000 | -.9069 | -.3735 |
|  |  | 2023 | -.3368 | .10484 | 1 | .001 | -.5423 | -.1314 |
|  |  | 2025 | -1.4757 | .12432 | 1 | .000 | -1.7194 | -1.2321 |
|  | 2025 | 2020 | .0899 | .20905 | 1 | .667 | -.3198 | .4997 |
|  |  | 2021 | -1.8867 | .26394 | 1 | .000 | -2.4040 | -1.3694 |
|  |  | 2022 | .8356 | .14990 | 1 | .000 | .5418 | 1.1293 |
|  |  | 2023 | 1.1389 | .12449 | 1 | .000 | .8949 | 1.3829 |
|  |  | 2024 | 1.4757 | .12432 | 1 | .000 | 1.2321 | 1.7194 |
| 2 | 2020 | 2021 | -1.1765 | .12789 | 1 | .000 | -1.4271 | -.9258 |
|  |  | 2022 | -.8554 | .11516 | 1 | .000 | -1.0811 | -.6296 |
|  |  | 2023 | -1.1614 | .11041 | 1 | .000 | -1.3778 | -.9450 |
|  |  | 2024 | -1.0495 | .10959 | 1 | .000 | -1.2643 | -.8347 |
|  |  | 2025 | -.1584 | .11634 | 1 | .173 | -.3864 | .0696 |
|  | 2021 | 2020 | 1.1765 | .12789 | 1 | .000 | .9258 | 1.4271 |
|  |  | 2022 | .3211 | .08250 | 1 | .000 | .1594 | .4828 |
|  |  | 2023 | .0151 | .07570 | 1 | .842 | -.1333 | .1635 |
|  |  | 2024 | .1270 | .07451 | 1 | .088 | -.0191 | .2730 |
|  |  | 2025 | 1.0180 | .08421 | 1 | .000 | .8530 | 1.1831 |
|  | 2022 | 2020 | .8554 | .11516 | 1 | .000 | .6296 | 1.0811 |
|  |  | 2021 | -.3211 | .08250 | 1 | .000 | -.4828 | -.1594 |
|  |  | 2023 | -.3060 | .05137 | 1 | .000 | -.4067 | -.2053 |
|  |  | 2024 | -.1941 | .04958 | 1 | .000 | -.2913 | -.0970 |
|  |  | 2025 | .6969 | .06324 | 1 | .000 | .5730 | .8209 |
|  | 2023 | 2020 | 1.1614 | .11041 | 1 | .000 | .9450 | 1.3778 |
|  |  | 2021 | -.0151 | .07570 | 1 | .842 | -.1635 | .1333 |
|  |  | 2022 | .3060 | .05137 | 1 | .000 | .2053 | .4067 |
|  |  | 2024 | .1119 | .03720 | 1 | .003 | .0390 | .1848 |
|  |  | 2025 | 1.0029 | .05410 | 1 | .000 | .8969 | 1.1090 |
|  | 2024 | 2020 | 1.0495 | .10959 | 1 | .000 | .8347 | 1.2643 |
|  |  | 2021 | -.1270 | .07451 | 1 | .088 | -.2730 | .0191 |
|  |  | 2022 | .1941 | .04958 | 1 | .000 | .0970 | .2913 |
|  |  | 2023 | -.1119 | .03720 | 1 | .003 | -.1848 | -.0390 |
|  |  | 2025 | .8911 | .05240 | 1 | .000 | .7884 | .9938 |
|  | 2025 | 2020 | .1584 | .11634 | 1 | .173 | -.0696 | .3864 |
|  |  | 2021 | -1.0180 | .08421 | 1 | .000 | -1.1831 | -.8530 |
|  |  | 2022 | -.6969 | .06324 | 1 | .000 | -.8209 | -.5730 |
|  |  | 2023 | -1.0029 | .05410 | 1 | .000 | -1.1090 | -.8969 |
|  |  | 2024 | -.8911 | .05240 | 1 | .000 | -.9938 | -.7884 |
| 3 | 2020 | 2021 | .4340 | .47773 | 1 | .364 | -.5024 | 1.3703 |
|  |  | 2022 | .7542 | .46290 | 1 | .103 | -.1530 | 1.6615 |
|  |  | 2023 | .5887 | .45780 | 1 | .198 | -.3086 | 1.4859 |
|  |  | 2024 | -.0964 | .45800 | 1 | .833 | -.9941 | .8012 |
|  |  | 2025 | 1.7026 | .47408 | 1 | .000 | .7735 | 2.6318 |
|  | 2021 | 2020 | -.4340 | .47773 | 1 | .364 | -1.3703 | .5024 |
|  |  | 2022 | .3203 | .16290 | 1 | .049 | .0010 | .6396 |
|  |  | 2023 | .1547 | .14770 | 1 | .295 | -.1348 | .4442 |
|  |  | 2024 | -.5304 | .14843 | 1 | .000 | -.8213 | -.2395 |
|  |  | 2025 | 1.2687 | .19244 | 1 | .000 | .8915 | 1.6459 |
|  | 2022 | 2020 | -.7542 | .46290 | 1 | .103 | -1.6615 | .1530 |
|  |  | 2021 | -.3203 | .16290 | 1 | .049 | -.6396 | -.0010 |
|  |  | 2023 | -.1656 | .08853 | 1 | .061 | -.3391 | .0080 |
|  |  | 2024 | -.8507 | .08980 | 1 | .000 | -1.0267 | -.6747 |
|  |  | 2025 | .9484 | .15188 | 1 | .000 | .6507 | 1.2461 |
|  | 2023 | 2020 | -.5887 | .45780 | 1 | .198 | -1.4859 | .3086 |
|  |  | 2021 | -.1547 | .14770 | 1 | .295 | -.4442 | .1348 |
|  |  | 2022 | .1656 | .08853 | 1 | .061 | -.0080 | .3391 |
|  |  | 2024 | -.6851 | .05777 | 1 | .000 | -.7983 | -.5719 |
|  |  | 2025 | 1.1140 | .13546 | 1 | .000 | .8485 | 1.3795 |
|  | 2024 | 2020 | .0964 | .45800 | 1 | .833 | -.8012 | .9941 |
|  |  | 2021 | .5304 | .14843 | 1 | .000 | .2395 | .8213 |
|  |  | 2022 | .8507 | .08980 | 1 | .000 | .6747 | 1.0267 |
|  |  | 2023 | .6851 | .05777 | 1 | .000 | .5719 | .7983 |
|  |  | 2025 | 1.7991 | .13626 | 1 | .000 | 1.5320 | 2.0661 |
|  | 2025 | 2020 | -1.7026 | .47408 | 1 | .000 | -2.6318 | -.7735 |
|  |  | 2021 | -1.2687 | .19244 | 1 | .000 | -1.6459 | -.8915 |
|  |  | 2022 | -.9484 | .15188 | 1 | .000 | -1.2461 | -.6507 |
|  |  | 2023 | -1.1140 | .13546 | 1 | .000 | -1.3795 | -.8485 |
|  |  | 2024 | -1.7991 | .13626 | 1 | .000 | -2.0661 | -1.5320 |
| 4 | 2020 | 2021 | .2174 | .56554 | 1 | .701 | -.8911 | 1.3258 |
|  |  | 2022 | .9065 | .54456 | 1 | .096 | -.1608 | 1.9738 |
|  |  | 2023 | .6857 | .53039 | 1 | .196 | -.3539 | 1.7252 |
|  |  | 2024 | -.2631 | .53138 | 1 | .621 | -1.3046 | .7784 |
|  |  | 2025 | 1.4161 | .56062 | 1 | .012 | .3173 | 2.5149 |
|  | 2021 | 2020 | -.2174 | .56554 | 1 | .701 | -1.3258 | .8911 |
|  |  | 2022 | .6891 | .24048 | 1 | .004 | .2178 | 1.1605 |
|  |  | 2023 | .4683 | .20609 | 1 | .023 | .0644 | .8722 |
|  |  | 2024 | -.4805 | .20867 | 1 | .021 | -.8894 | -.0715 |
|  |  | 2025 | 1.1987 | .27495 | 1 | .000 | .6598 | 1.7375 |
|  | 2022 | 2020 | -.9065 | .54456 | 1 | .096 | -1.9738 | .1608 |
|  |  | 2021 | -.6891 | .24048 | 1 | .004 | -1.1605 | -.2178 |
|  |  | 2023 | -.2208 | .13857 | 1 | .111 | -.4924 | .0507 |
|  |  | 2024 | -1.1696 | .14241 | 1 | .000 | -1.4487 | -.8905 |
|  |  | 2025 | .5095 | .22873 | 1 | .026 | .0612 | .9578 |
|  | 2023 | 2020 | -.6857 | .53039 | 1 | .196 | -1.7252 | .3539 |
|  |  | 2021 | -.4683 | .20609 | 1 | .023 | -.8722 | -.0644 |
|  |  | 2022 | .2208 | .13857 | 1 | .111 | -.0507 | .4924 |
|  |  | 2024 | -.9488 | .06985 | 1 | .000 | -1.0857 | -.8118 |
|  |  | 2025 | .7304 | .19224 | 1 | .000 | .3536 | 1.1072 |
|  | 2024 | 2020 | .2631 | .53138 | 1 | .621 | -.7784 | 1.3046 |
|  |  | 2021 | .4805 | .20867 | 1 | .021 | .0715 | .8894 |
|  |  | 2022 | 1.1696 | .14241 | 1 | .000 | .8905 | 1.4487 |
|  |  | 2023 | .9488 | .06985 | 1 | .000 | .8118 | 1.0857 |
|  |  | 2025 | 1.6791 | .19500 | 1 | .000 | 1.2969 | 2.0613 |
|  | 2025 | 2020 | -1.4161 | .56062 | 1 | .012 | -2.5149 | -.3173 |
|  |  | 2021 | -1.1987 | .27495 | 1 | .000 | -1.7375 | -.6598 |
|  |  | 2022 | -.5095 | .22873 | 1 | .026 | -.9578 | -.0612 |
|  |  | 2023 | -.7304 | .19224 | 1 | .000 | -1.1072 | -.3536 |
|  |  | 2024 | -1.6791 | .19500 | 1 | .000 | -2.0613 | -1.2969 |

| **Overall Test** | | | |
| --- | --- | --- | --- |
| Age | Wald χ² | Degrees of Freedom | P |
| 1 | 266.494 | 5 | .000 |
| 2 | 459.410 | 5 | .000 |
| 3 | 282.822 | 5 | .000 |
| 4 | 235.819 | 5 | .000 |

**Estimated Marginal Means 8：Year* Age**

| **Estimate** | | | | | |
| --- | --- | --- | --- | --- | --- |
| Year | Age | Mean | Standard Error | 95% Wald Confidence Interval | |
|  |  |  |  | Lower Bound | Upper Bound |
| 2020 | 1 | 4.7014 | .19866 | 4.3277 | 5.1073 |
|  | 2 | 3.6642 | .10683 | 3.4607 | 3.8797 |
|  | 3 | 6.5022 | .45619 | 5.6668 | 7.4607 |
|  | 4 | 6.5148 | .52860 | 5.5569 | 7.6377 |
| 2021 | 1 | 6.6780 | .26185 | 6.1840 | 7.2114 |
|  | 2 | 4.8407 | .07037 | 4.7047 | 4.9806 |
|  | 3 | 6.0682 | .14252 | 5.7952 | 6.3541 |
|  | 4 | 6.2974 | .20147 | 5.9146 | 6.7049 |
| 2022 | 1 | 3.9557 | .12140 | 3.7248 | 4.2010 |
|  | 2 | 4.5196 | .04314 | 4.4358 | 4.6049 |
|  | 3 | 5.7480 | .07953 | 5.5942 | 5.9060 |
|  | 4 | 5.6083 | .13142 | 5.3565 | 5.8718 |
| 2023 | 1 | 3.6524 | .08251 | 3.4942 | 3.8177 |
|  | 2 | 4.8256 | .02802 | 4.7710 | 4.8808 |
|  | 3 | 5.9135 | .03952 | 5.8366 | 5.9915 |
|  | 4 | 5.8291 | .04411 | 5.7433 | 5.9162 |
| 2024 | 1 | 3.3155 | .07741 | 3.1672 | 3.4708 |
|  | 2 | 4.7137 | .02463 | 4.6657 | 4.7623 |
|  | 3 | 6.5986 | .04281 | 6.5153 | 6.6831 |
|  | 4 | 6.7779 | .05505 | 6.6708 | 6.8866 |
| 2025 | 1 | 4.7913 | .11432 | 4.5724 | 5.0207 |
|  | 2 | 3.8226 | .04636 | 3.7329 | 3.9146 |
|  | 3 | 4.7996 | .12978 | 4.5518 | 5.0608 |
|  | 4 | 5.0987 | .18729 | 4.7446 | 5.4793 |

| **Pairwise Comparisons** | | | | | | | | |
| --- | --- | --- | --- | --- | --- | --- | --- | --- |
| Year | (I) Age | (J) Age | Mean Difference (I-J) | Standard Error | Degrees of Freedom | P | 95% Wald Confidence Interval | |
|  |  |  |  |  |  |  | Lower Bound | Upper Bound |
| 2020 | 1 | 2 | 1.0371 | .20255 | 1 | .000 | .6401 | 1.4341 |
|  |  | 3 | -1.8008 | .48319 | 1 | .000 | -2.7479 | -.8538 |
|  |  | 4 | -1.8134 | .56672 | 1 | .001 | -2.9242 | -.7027 |
|  | 2 | 1 | -1.0371 | .20255 | 1 | .000 | -1.4341 | -.6401 |
|  |  | 3 | -2.8380 | .46254 | 1 | .000 | -3.7445 | -1.9314 |
|  |  | 4 | -2.8506 | .53896 | 1 | .000 | -3.9069 | -1.7942 |
|  | 3 | 1 | 1.8008 | .48319 | 1 | .000 | .8538 | 2.7479 |
|  |  | 2 | 2.8380 | .46254 | 1 | .000 | 1.9314 | 3.7445 |
|  |  | 4 | -.0126 | .69808 | 1 | .986 | -1.3808 | 1.3556 |
|  | 4 | 1 | 1.8134 | .56672 | 1 | .001 | .7027 | 2.9242 |
|  |  | 2 | 2.8506 | .53896 | 1 | .000 | 1.7942 | 3.9069 |
|  |  | 3 | .0126 | .69808 | 1 | .986 | -1.3556 | 1.3808 |
| 2021 | 1 | 2 | 1.8373 | .26964 | 1 | .000 | 1.3088 | 2.3658 |
|  |  | 3 | .6097 | .29387 | 1 | .038 | .0338 | 1.1857 |
|  |  | 4 | .3806 | .32875 | 1 | .247 | -.2637 | 1.0249 |
|  | 2 | 1 | -1.8373 | .26964 | 1 | .000 | -2.3658 | -1.3088 |
|  |  | 3 | -1.2276 | .15770 | 1 | .000 | -1.5366 | -.9185 |
|  |  | 4 | -1.4567 | .21282 | 1 | .000 | -1.8738 | -1.0396 |
|  | 3 | 1 | -.6097 | .29387 | 1 | .038 | -1.1857 | -.0338 |
|  |  | 2 | 1.2276 | .15770 | 1 | .000 | .9185 | 1.5366 |
|  |  | 4 | -.2291 | .24582 | 1 | .351 | -.7110 | .2527 |
|  | 4 | 1 | -.3806 | .32875 | 1 | .247 | -1.0249 | .2637 |
|  |  | 2 | 1.4567 | .21282 | 1 | .000 | 1.0396 | 1.8738 |
|  |  | 3 | .2291 | .24582 | 1 | .351 | -.2527 | .7110 |
| 2022 | 1 | 2 | -.5639 | .12783 | 1 | .000 | -.8144 | -.3133 |
|  |  | 3 | -1.7922 | .14363 | 1 | .000 | -2.0737 | -1.5107 |
|  |  | 4 | -1.6525 | .17877 | 1 | .000 | -2.0029 | -1.3021 |
|  | 2 | 1 | .5639 | .12783 | 1 | .000 | .3133 | .8144 |
|  |  | 3 | -1.2284 | .08957 | 1 | .000 | -1.4039 | -1.0528 |
|  |  | 4 | -1.0887 | .13820 | 1 | .000 | -1.3595 | -.8178 |
|  | 3 | 1 | 1.7922 | .14363 | 1 | .000 | 1.5107 | 2.0737 |
|  |  | 2 | 1.2284 | .08957 | 1 | .000 | 1.0528 | 1.4039 |
|  |  | 4 | .1397 | .15348 | 1 | .363 | -.1611 | .4405 |
|  | 4 | 1 | 1.6525 | .17877 | 1 | .000 | 1.3021 | 2.0029 |
|  |  | 2 | 1.0887 | .13820 | 1 | .000 | .8178 | 1.3595 |
|  |  | 3 | -.1397 | .15348 | 1 | .363 | -.4405 | .1611 |
| 2023 | 1 | 2 | -1.1732 | .08698 | 1 | .000 | -1.3437 | -1.0027 |
|  |  | 3 | -2.2611 | .09126 | 1 | .000 | -2.4400 | -2.0823 |
|  |  | 4 | -2.1767 | .09330 | 1 | .000 | -2.3596 | -1.9938 |
|  | 2 | 1 | 1.1732 | .08698 | 1 | .000 | 1.0027 | 1.3437 |
|  |  | 3 | -1.0879 | .04833 | 1 | .000 | -1.1827 | -.9932 |
|  |  | 4 | -1.0035 | .05212 | 1 | .000 | -1.1057 | -.9014 |
|  | 3 | 1 | 2.2611 | .09126 | 1 | .000 | 2.0823 | 2.4400 |
|  |  | 2 | 1.0879 | .04833 | 1 | .000 | .9932 | 1.1827 |
|  |  | 4 | .0844 | .05904 | 1 | .153 | -.0313 | .2001 |
|  | 4 | 1 | 2.1767 | .09330 | 1 | .000 | 1.9938 | 2.3596 |
|  |  | 2 | 1.0035 | .05212 | 1 | .000 | .9014 | 1.1057 |
|  |  | 3 | -.0844 | .05904 | 1 | .153 | -.2001 | .0313 |
| 2024 | 1 | 2 | -1.3982 | .08102 | 1 | .000 | -1.5570 | -1.2394 |
|  |  | 3 | -3.2831 | .08809 | 1 | .000 | -3.4558 | -3.1104 |
|  |  | 4 | -3.4623 | .09472 | 1 | .000 | -3.6480 | -3.2767 |
|  | 2 | 1 | 1.3982 | .08102 | 1 | .000 | 1.2394 | 1.5570 |
|  |  | 3 | -1.8849 | .04912 | 1 | .000 | -1.9812 | -1.7886 |
|  |  | 4 | -2.0641 | .06013 | 1 | .000 | -2.1820 | -1.9463 |
|  | 3 | 1 | 3.2831 | .08809 | 1 | .000 | 3.1104 | 3.4558 |
|  |  | 2 | 1.8849 | .04912 | 1 | .000 | 1.7886 | 1.9812 |
|  |  | 4 | -.1792 | .06940 | 1 | .010 | -.3152 | -.0432 |
|  | 4 | 1 | 3.4623 | .09472 | 1 | .000 | 3.2767 | 3.6480 |
|  |  | 2 | 2.0641 | .06013 | 1 | .000 | 1.9463 | 2.1820 |
|  |  | 3 | .1792 | .06940 | 1 | .010 | .0432 | .3152 |
| 2025 | 1 | 2 | .9686 | .11741 | 1 | .000 | .7385 | 1.1987 |
|  |  | 3 | -.0083 | .16856 | 1 | .961 | -.3387 | .3221 |
|  |  | 4 | -.3074 | .21784 | 1 | .158 | -.7344 | .1195 |
|  | 2 | 1 | -.9686 | .11741 | 1 | .000 | -1.1987 | -.7385 |
|  |  | 3 | -.9769 | .13572 | 1 | .000 | -1.2429 | -.7109 |
|  |  | 4 | -1.2761 | .19207 | 1 | .000 | -1.6525 | -.8996 |
|  | 3 | 1 | .0083 | .16856 | 1 | .961 | -.3221 | .3387 |
|  |  | 2 | .9769 | .13572 | 1 | .000 | .7109 | 1.2429 |
|  |  | 4 | -.2992 | .22718 | 1 | .188 | -.7444 | .1461 |
|  | 4 | 1 | .3074 | .21784 | 1 | .158 | -.1195 | .7344 |
|  |  | 2 | 1.2761 | .19207 | 1 | .000 | .8996 | 1.6525 |
|  |  | 3 | .2992 | .22718 | 1 | .188 | -.1461 | .7444 |

| **Overall Test** | | | |
| --- | --- | --- | --- |
| Year | Wald χ² | Degrees of Freedom | P |
| 2020 | 81.340 | 3 | .000 |
| 2021 | 123.729 | 3 | .000 |
| 2022 | 275.749 | 3 | .000 |
| 2023 | 1063.652 | 3 | .000 |
| 2024 | 2884.510 | 3 | .000 |
| 2025 | 139.600 | 3 | .000 |

**Estimated Marginal Means 9：Year* Diagnostic**

| **Estimate** | | | | | |
| --- | --- | --- | --- | --- | --- |
| Year | Diagnostic | Mean | Standard Error | 95% Wald Confidence Interval | |
|  |  |  |  | Lower Bound | Upper Bound |
| 2020 | 1 | 5.4144 | .17631 | 5.0796 | 5.7712 |
|  | 2 | 4.9892 | .23213 | 4.5544 | 5.4656 |
| 2021 | 1 | 5.6377 | .08916 | 5.4656 | 5.8152 |
|  | 2 | 6.2343 | .13546 | 5.9744 | 6.5055 |
| 2022 | 1 | 4.9227 | .05702 | 4.8122 | 5.0357 |
|  | 2 | 4.8768 | .07343 | 4.7350 | 5.0228 |
| 2023 | 1 | 4.9732 | .03322 | 4.9085 | 5.0387 |
|  | 2 | 4.9562 | .04710 | 4.8648 | 5.0494 |
| 2024 | 1 | 4.8635 | .03355 | 4.7982 | 4.9297 |
|  | 2 | 5.4361 | .05186 | 5.3354 | 5.5387 |
| 2025 | 1 | 4.3697 | .06040 | 4.2529 | 4.4897 |
|  | 2 | 4.8450 | .10113 | 4.6508 | 5.0473 |

| **Pairwise Comparisons** | | | | | | | | |
| --- | --- | --- | --- | --- | --- | --- | --- | --- |
| Diagnostic | (I) Year | (J) Year | Mean Difference (I-J) | Standard Error | Degrees of Freedom | P | 95% Wald Confidence Interval | |
|  |  |  |  |  |  |  | Lower Bound | Upper Bound |
| 1 | 2020 | 2021 | -.2233 | .19734 | 1 | .258 | -.6100 | .1635 |
|  |  | 2022 | .4917 | .18510 | 1 | .008 | .1289 | .8545 |
|  |  | 2023 | .4412 | .17948 | 1 | .014 | .0894 | .7930 |
|  |  | 2024 | .5509 | .17950 | 1 | .002 | .1991 | .9027 |
|  |  | 2025 | 1.0447 | .18606 | 1 | .000 | .6801 | 1.4094 |
|  | 2021 | 2020 | .2233 | .19734 | 1 | .258 | -.1635 | .6100 |
|  |  | 2022 | .7150 | .10544 | 1 | .000 | .5083 | .9217 |
|  |  | 2023 | .6645 | .09498 | 1 | .000 | .4783 | .8506 |
|  |  | 2024 | .7742 | .09500 | 1 | .000 | .5880 | .9604 |
|  |  | 2025 | 1.2680 | .10739 | 1 | .000 | 1.0575 | 1.4785 |
|  | 2022 | 2020 | -.4917 | .18510 | 1 | .008 | -.8545 | -.1289 |
|  |  | 2021 | -.7150 | .10544 | 1 | .000 | -.9217 | -.5083 |
|  |  | 2023 | -.0505 | .06533 | 1 | .439 | -.1786 | .0775 |
|  |  | 2024 | .0592 | .06526 | 1 | .364 | -.0687 | .1871 |
|  |  | 2025 | .5530 | .08270 | 1 | .000 | .3909 | .7151 |
|  | 2023 | 2020 | -.4412 | .17948 | 1 | .014 | -.7930 | -.0894 |
|  |  | 2021 | -.6645 | .09498 | 1 | .000 | -.8506 | -.4783 |
|  |  | 2022 | .0505 | .06533 | 1 | .439 | -.0775 | .1786 |
|  |  | 2024 | .1097 | .04586 | 1 | .017 | .0198 | .1996 |
|  |  | 2025 | .6035 | .06892 | 1 | .000 | .4685 | .7386 |
|  | 2024 | 2020 | -.5509 | .17950 | 1 | .002 | -.9027 | -.1991 |
|  |  | 2021 | -.7742 | .09500 | 1 | .000 | -.9604 | -.5880 |
|  |  | 2022 | -.0592 | .06526 | 1 | .364 | -.1871 | .0687 |
|  |  | 2023 | -.1097 | .04586 | 1 | .017 | -.1996 | -.0198 |
|  |  | 2025 | .4938 | .06900 | 1 | .000 | .3586 | .6291 |
|  | 2025 | 2020 | -1.0447 | .18606 | 1 | .000 | -1.4094 | -.6801 |
|  |  | 2021 | -1.2680 | .10739 | 1 | .000 | -1.4785 | -1.0575 |
|  |  | 2022 | -.5530 | .08270 | 1 | .000 | -.7151 | -.3909 |
|  |  | 2023 | -.6035 | .06892 | 1 | .000 | -.7386 | -.4685 |
|  |  | 2024 | -.4938 | .06900 | 1 | .000 | -.6291 | -.3586 |
| 2 | 2020 | 2021 | -1.2451 | .26327 | 1 | .000 | -1.7611 | -.7291 |
|  |  | 2022 | .1125 | .23956 | 1 | .639 | -.3571 | .5820 |
|  |  | 2023 | .0330 | .23300 | 1 | .887 | -.4237 | .4897 |
|  |  | 2024 | -.4469 | .23371 | 1 | .056 | -.9049 | .0112 |
|  |  | 2025 | .1442 | .24854 | 1 | .562 | -.3429 | .6314 |
|  | 2021 | 2020 | 1.2451 | .26327 | 1 | .000 | .7291 | 1.7611 |
|  |  | 2022 | 1.3575 | .14575 | 1 | .000 | 1.0719 | 1.6432 |
|  |  | 2023 | 1.2781 | .13469 | 1 | .000 | 1.0141 | 1.5421 |
|  |  | 2024 | .7982 | .13574 | 1 | .000 | .5322 | 1.0643 |
|  |  | 2025 | 1.3893 | .15995 | 1 | .000 | 1.0758 | 1.7028 |
|  | 2022 | 2020 | -.1125 | .23956 | 1 | .639 | -.5820 | .3571 |
|  |  | 2021 | -1.3575 | .14575 | 1 | .000 | -1.6432 | -1.0719 |
|  |  | 2023 | -.0795 | .07750 | 1 | .305 | -.2314 | .0724 |
|  |  | 2024 | -.5593 | .07968 | 1 | .000 | -.7155 | -.4031 |
|  |  | 2025 | .0318 | .11690 | 1 | .786 | -.1973 | .2609 |
|  | 2023 | 2020 | -.0330 | .23300 | 1 | .887 | -.4897 | .4237 |
|  |  | 2021 | -1.2781 | .13469 | 1 | .000 | -1.5421 | -1.0141 |
|  |  | 2022 | .0795 | .07750 | 1 | .305 | -.0724 | .2314 |
|  |  | 2024 | -.4798 | .05655 | 1 | .000 | -.5907 | -.3690 |
|  |  | 2025 | .1112 | .10286 | 1 | .279 | -.0904 | .3129 |
|  | 2024 | 2020 | .4469 | .23371 | 1 | .056 | -.0112 | .9049 |
|  |  | 2021 | -.7982 | .13574 | 1 | .000 | -1.0643 | -.5322 |
|  |  | 2022 | .5593 | .07968 | 1 | .000 | .4031 | .7155 |
|  |  | 2023 | .4798 | .05655 | 1 | .000 | .3690 | .5907 |
|  |  | 2025 | .5911 | .10438 | 1 | .000 | .3865 | .7957 |
|  | 2025 | 2020 | -.1442 | .24854 | 1 | .562 | -.6314 | .3429 |
|  |  | 2021 | -1.3893 | .15995 | 1 | .000 | -1.7028 | -1.0758 |
|  |  | 2022 | -.0318 | .11690 | 1 | .786 | -.2609 | .1973 |
|  |  | 2023 | -.1112 | .10286 | 1 | .279 | -.3129 | .0904 |
|  |  | 2024 | -.5911 | .10438 | 1 | .000 | -.7957 | -.3865 |

| **Overall Test** | | | |
| --- | --- | --- | --- |
| Diagnostic | Wald χ² | Degrees of Freedom | P |
| 1 | 161.502 | 5 | .000 |
| 2 | 170.039 | 5 | .000 |

**Estimated Marginal Means 10：Year* Diagnostic**

| **Estimate** | | | | | |
| --- | --- | --- | --- | --- | --- |
| Year | Diagnostic | Mean | Standard Error | 95% Wald Confidence Interval | |
|  |  |  |  | Lower Bound | Upper Bound |
| 2020 | 1 | 5.4144 | .17631 | 5.0796 | 5.7712 |
|  | 2 | 4.9892 | .23213 | 4.5544 | 5.4656 |
| 2021 | 1 | 5.6377 | .08916 | 5.4656 | 5.8152 |
|  | 2 | 6.2343 | .13546 | 5.9744 | 6.5055 |
| 2022 | 1 | 4.9227 | .05702 | 4.8122 | 5.0357 |
|  | 2 | 4.8768 | .07343 | 4.7350 | 5.0228 |
| 2023 | 1 | 4.9732 | .03322 | 4.9085 | 5.0387 |
|  | 2 | 4.9562 | .04710 | 4.8648 | 5.0494 |
| 2024 | 1 | 4.8635 | .03355 | 4.7982 | 4.9297 |
|  | 2 | 5.4361 | .05186 | 5.3354 | 5.5387 |
| 2025 | 1 | 4.3697 | .06040 | 4.2529 | 4.4897 |
|  | 2 | 4.8450 | .10113 | 4.6508 | 5.0473 |

| **Pairwise Comparisons** | | | | | | | | |
| --- | --- | --- | --- | --- | --- | --- | --- | --- |
| Year | (I) Diagnostic | (J) Diagnostic | Mean Difference (I-J) | Standard Error | Degrees of Freedom | P | 95% Wald Confidence Interval | |
|  |  |  |  |  |  |  | Lower Bound | Upper Bound |
| 2020 | 1 | 2 | .4252 | .26154 | 1 | .104 | -.0874 | .9378 |
|  | 2 | 1 | -.4252 | .26154 | 1 | .104 | -.9378 | .0874 |
| 2021 | 1 | 2 | -.5966 | .14556 | 1 | .000 | -.8819 | -.3113 |
|  | 2 | 1 | .5966 | .14556 | 1 | .000 | .3113 | .8819 |
| 2022 | 1 | 2 | .0459 | .07996 | 1 | .566 | -.1108 | .2026 |
|  | 2 | 1 | -.0459 | .07996 | 1 | .566 | -.2026 | .1108 |
| 2023 | 1 | 2 | .0170 | .05121 | 1 | .740 | -.0834 | .1174 |
|  | 2 | 1 | -.0170 | .05121 | 1 | .740 | -.1174 | .0834 |
| 2024 | 1 | 2 | -.5726 | .05400 | 1 | .000 | -.6784 | -.4668 |
|  | 2 | 1 | .5726 | .05400 | 1 | .000 | .4668 | .6784 |
| 2025 | 1 | 2 | -.4753 | .10540 | 1 | .000 | -.6819 | -.2687 |
|  | 2 | 1 | .4753 | .10540 | 1 | .000 | .2687 | .6819 |

| **Overall Test** | | | |
| --- | --- | --- | --- |
| Year | Wald χ² | Degrees of Freedom | P |
| 2020 | 2.643 | 1 | .104 |
| 2021 | 16.801 | 1 | .000 |
| 2022 | .330 | 1 | .566 |
| 2023 | .110 | 1 | .740 |
| 2024 | 112.451 | 1 | .000 |
| 2025 | 20.337 | 1 | .000 |

**Estimated Marginal Means 11：Gender* Age**

| **Estimate** | | | | | |
| --- | --- | --- | --- | --- | --- |
| Gender | Age | Mean | Standard Error | 95% Wald Confidence Interval | |
|  |  |  |  | Lower Bound | Upper Bound |
| 1 | 1 | 4.1578 | .07720 | 4.0092 | 4.3119 |
|  | 2 | 4.2768 | .03100 | 4.2165 | 4.3380 |
|  | 3 | 5.9298 | .08523 | 5.7651 | 6.0992 |
|  | 4 | 5.9955 | .10210 | 5.7987 | 6.1990 |
| 2 | 1 | 4.6454 | .09282 | 4.4670 | 4.8310 |
|  | 2 | 4.4665 | .03783 | 4.3930 | 4.5413 |
|  | 3 | 5.8840 | .08871 | 5.7127 | 6.0605 |
|  | 4 | 5.9913 | .11031 | 5.7790 | 6.2115 |

| **Pairwise Comparisons** | | | | | | | | |
| --- | --- | --- | --- | --- | --- | --- | --- | --- |
| Age | (I) Gender | (J) Gender | Mean Difference (I-J) | Standard Error | Degrees of Freedom | P | 95% Wald Confidence Interval | |
|  |  |  |  |  |  |  | Lower Bound | Upper Bound |
| 1 | 1 | 2 | -.4876 | .08959 | 1 | .000 | -.6632 | -.3120 |
|  | 2 | 1 | .4876 | .08959 | 1 | .000 | .3120 | .6632 |
| 2 | 1 | 2 | -.1897 | .04313 | 1 | .000 | -.2743 | -.1052 |
|  | 2 | 1 | .1897 | .04313 | 1 | .000 | .1052 | .2743 |
| 3 | 1 | 2 | .0457 | .07036 | 1 | .516 | -.0922 | .1836 |
|  | 2 | 1 | -.0457 | .07036 | 1 | .516 | -.1836 | .0922 |
| 4 | 1 | 2 | .0042 | .08140 | 1 | .959 | -.1554 | .1637 |
|  | 2 | 1 | -.0042 | .08140 | 1 | .959 | -.1637 | .1554 |

| **Overall Test** | | | |
| --- | --- | --- | --- |
| Age | Wald χ² | Degrees of Freedom | P |
| 1 | 29.619 | 1 | .000 |
| 2 | 19.348 | 1 | .000 |
| 3 | .422 | 1 | .516 |
| 4 | .003 | 1 | .959 |

**Estimated Marginal Means 12：Gender* Age**

| **Estimate** | | | | | |
| --- | --- | --- | --- | --- | --- |
| Gender | Age | Mean | Standard Error | 95% Wald Confidence Interval | |
|  |  |  |  | Lower Bound | Upper Bound |
| 1 | 1 | 4.1578 | .07720 | 4.0092 | 4.3119 |
|  | 2 | 4.2768 | .03100 | 4.2165 | 4.3380 |
|  | 3 | 5.9298 | .08523 | 5.7651 | 6.0992 |
|  | 4 | 5.9955 | .10210 | 5.7987 | 6.1990 |
| 2 | 1 | 4.6454 | .09282 | 4.4670 | 4.8310 |
|  | 2 | 4.4665 | .03783 | 4.3930 | 4.5413 |
|  | 3 | 5.8840 | .08871 | 5.7127 | 6.0605 |
|  | 4 | 5.9913 | .11031 | 5.7790 | 6.2115 |

| **Pairwise Comparisons** | | | | | | | | |
| --- | --- | --- | --- | --- | --- | --- | --- | --- |
| Gender | (I) Age | (J) Age | Mean Difference (I-J) | Standard Error | Degrees of Freedom | P | 95% Wald Confidence Interval | |
|  |  |  |  |  |  |  | Lower Bound | Upper Bound |
| 1 | 1 | 2 | -.1190 | .08088 | 1 | .141 | -.2775 | .0396 |
|  |  | 3 | -1.7719 | .11281 | 1 | .000 | -1.9930 | -1.5508 |
|  |  | 4 | -1.8377 | .12786 | 1 | .000 | -2.0883 | -1.5871 |
|  | 2 | 1 | .1190 | .08088 | 1 | .141 | -.0396 | .2775 |
|  |  | 3 | -1.6530 | .08701 | 1 | .000 | -1.8235 | -1.4824 |
|  |  | 4 | -1.7187 | .10516 | 1 | .000 | -1.9248 | -1.5126 |
|  | 3 | 1 | 1.7719 | .11281 | 1 | .000 | 1.5508 | 1.9930 |
|  |  | 2 | 1.6530 | .08701 | 1 | .000 | 1.4824 | 1.8235 |
|  |  | 4 | -.0657 | .13060 | 1 | .615 | -.3217 | .1902 |
|  | 4 | 1 | 1.8377 | .12786 | 1 | .000 | 1.5871 | 2.0883 |
|  |  | 2 | 1.7187 | .10516 | 1 | .000 | 1.5126 | 1.9248 |
|  |  | 3 | .0657 | .13060 | 1 | .615 | -.1902 | .3217 |
| 2 | 1 | 2 | .1789 | .09677 | 1 | .064 | -.0108 | .3686 |
|  |  | 3 | -1.2386 | .12485 | 1 | .000 | -1.4833 | -.9939 |
|  |  | 4 | -1.3459 | .14222 | 1 | .000 | -1.6246 | -1.0671 |
|  | 2 | 1 | -.1789 | .09677 | 1 | .064 | -.3686 | .0108 |
|  |  | 3 | -1.4175 | .08940 | 1 | .000 | -1.5927 | -1.2423 |
|  |  | 4 | -1.5248 | .11058 | 1 | .000 | -1.7415 | -1.3081 |
|  | 3 | 1 | 1.2386 | .12485 | 1 | .000 | .9939 | 1.4833 |
|  |  | 2 | 1.4175 | .08940 | 1 | .000 | 1.2423 | 1.5927 |
|  |  | 4 | -.1073 | .13423 | 1 | .424 | -.3704 | .1558 |
|  | 4 | 1 | 1.3459 | .14222 | 1 | .000 | 1.0671 | 1.6246 |
|  |  | 2 | 1.5248 | .11058 | 1 | .000 | 1.3081 | 1.7415 |
|  |  | 3 | .1073 | .13423 | 1 | .424 | -.1558 | .3704 |

| **Overall Test** | | | |
| --- | --- | --- | --- |
| Gender | Wald χ² | Degrees of Freedom | P |
| 1 | 597.765 | 3 | .000 |
| 2 | 397.991 | 3 | .000 |

**Estimated Marginal Means 13：Gender* Diagnostic**

| **Estimate** | | | | | |
| --- | --- | --- | --- | --- | --- |
| Gender | Diagnostic | Mean | Standard Error | 95% Wald Confidence Interval | |
|  |  |  |  | Lower Bound | Upper Bound |
| 1 | 1 | 4.8869 | .03873 | 4.8116 | 4.9634 |
|  | 2 | 5.1451 | .06252 | 5.0240 | 5.2691 |
| 2 | 1 | 5.1436 | .04604 | 5.0541 | 5.2346 |
|  | 2 | 5.2582 | .06928 | 5.1241 | 5.3957 |

| **Pairwise Comparisons** | | | | | | | | |
| --- | --- | --- | --- | --- | --- | --- | --- | --- |
| Diagnostic | (I) Gender | (J) Gender | Mean Difference (I-J) | Standard Error | Degrees of Freedom | P | 95% Wald Confidence Interval | |
|  |  |  |  |  |  |  | Lower Bound | Upper Bound |
| 1 | 1 | 2 | -.2567 | .04783 | 1 | .000 | -.3504 | -.1629 |
|  | 2 | 1 | .2567 | .04783 | 1 | .000 | .1629 | .3504 |
| 2 | 1 | 2 | -.1131 | .05818 | 1 | .052 | -.2271 | .0010 |
|  | 2 | 1 | .1131 | .05818 | 1 | .052 | -.0010 | .2271 |

| **Overall Test** | | | |
| --- | --- | --- | --- |
| Diagnostic | Wald χ² | Degrees of Freedom | P |
| 1 | 28.806 | 1 | .000 |
| 2 | 3.777 | 1 | .052 |

**Estimated Marginal Means 14：Gender* Diagnostic**

| **Estimate** | | | | | |
| --- | --- | --- | --- | --- | --- |
| Gender | Diagnostic | Mean | Standard Error | 95% Wald Confidence Interval | |
|  |  |  |  | Lower Bound | Upper Bound |
| 1 | 1 | 4.8869 | .03873 | 4.8116 | 4.9634 |
|  | 2 | 5.1451 | .06252 | 5.0240 | 5.2691 |
| 2 | 1 | 5.1436 | .04604 | 5.0541 | 5.2346 |
|  | 2 | 5.2582 | .06928 | 5.1241 | 5.3957 |

| **Pairwise Comparisons** | | | | | | | | |
| --- | --- | --- | --- | --- | --- | --- | --- | --- |
| Gender | (I) Diagnostic | (J) Diagnostic | Mean Difference (I-J) | Standard Error | Degrees of Freedom | P | 95% Wald Confidence Interval | |
|  |  |  |  |  |  |  | Lower Bound | Upper Bound |
| 1 | 1 | 2 | -.2582 | .06676 | 1 | .000 | -.3891 | -.1274 |
|  | 2 | 1 | .2582 | .06676 | 1 | .000 | .1274 | .3891 |
| 2 | 1 | 2 | -.1146 | .07155 | 1 | .109 | -.2548 | .0256 |
|  | 2 | 1 | .1146 | .07155 | 1 | .109 | -.0256 | .2548 |

| **Overall Test** | | | |
| --- | --- | --- | --- |
| Gender | Wald χ² | Degrees of Freedom | P |
| 1 | 14.960 | 1 | .000 |
| 2 | 2.565 | 1 | .109 |

**Estimated Marginal Means 15：Age* Diagnostic**

| **Estimate** | | | | | |
| --- | --- | --- | --- | --- | --- |
| Age | Diagnostic | Mean | Standard Error | 95% Wald Confidence Interval | |
|  |  |  |  | Lower Bound | Upper Bound |
| 1 | 1 | 5.3615 | .06209 | 5.2411 | 5.4845 |
|  | 2 | 3.6025 | .10835 | 3.3963 | 3.8213 |
| 2 | 1 | 4.0922 | .02810 | 4.0375 | 4.1477 |
|  | 2 | 4.6680 | .04427 | 4.5820 | 4.7555 |
| 3 | 1 | 5.3073 | .07451 | 5.1633 | 5.4554 |
|  | 2 | 6.5741 | .10430 | 6.3728 | 6.7817 |
| 4 | 1 | 5.4259 | .09701 | 5.2390 | 5.6194 |
|  | 2 | 6.6203 | .11908 | 6.3910 | 6.8579 |

| **Pairwise Comparisons** | | | | | | | | |
| --- | --- | --- | --- | --- | --- | --- | --- | --- |
| Diagnostic | (I) Age | (J) Age | Mean Difference (I-J) | Standard Error | Degrees of Freedom | P | 95% Wald Confidence Interval | |
|  |  |  |  |  |  |  | Lower Bound | Upper Bound |
| 1 | 1 | 2 | 1.2692 | .06805 | 1 | .000 | 1.1358 | 1.4026 |
|  |  | 3 | .0541 | .09690 | 1 | .576 | -.1358 | .2441 |
|  |  | 4 | -.0644 | .11499 | 1 | .576 | -.2898 | .1610 |
|  | 2 | 1 | -1.2692 | .06805 | 1 | .000 | -1.4026 | -1.1358 |
|  |  | 3 | -1.2151 | .07722 | 1 | .000 | -1.3664 | -1.0637 |
|  |  | 4 | -1.3336 | .09770 | 1 | .000 | -1.5251 | -1.1421 |
|  | 3 | 1 | -.0541 | .09690 | 1 | .576 | -.2441 | .1358 |
|  |  | 2 | 1.2151 | .07722 | 1 | .000 | 1.0637 | 1.3664 |
|  |  | 4 | -.1185 | .11798 | 1 | .315 | -.3498 | .1127 |
|  | 4 | 1 | .0644 | .11499 | 1 | .576 | -.1610 | .2898 |
|  |  | 2 | 1.3336 | .09770 | 1 | .000 | 1.1421 | 1.5251 |
|  |  | 3 | .1185 | .11798 | 1 | .315 | -.1127 | .3498 |
| 2 | 1 | 2 | -1.0654 | .10924 | 1 | .000 | -1.2795 | -.8513 |
|  |  | 3 | -2.9716 | .14142 | 1 | .000 | -3.2487 | -2.6944 |
|  |  | 4 | -3.0178 | .15504 | 1 | .000 | -3.3217 | -2.7139 |
|  | 2 | 1 | 1.0654 | .10924 | 1 | .000 | .8513 | 1.2795 |
|  |  | 3 | -1.9061 | .10136 | 1 | .000 | -2.1048 | -1.7075 |
|  |  | 4 | -1.9524 | .11955 | 1 | .000 | -2.1867 | -1.7181 |
|  | 3 | 1 | 2.9716 | .14142 | 1 | .000 | 2.6944 | 3.2487 |
|  |  | 2 | 1.9061 | .10136 | 1 | .000 | 1.7075 | 2.1048 |
|  |  | 4 | -.0462 | .14918 | 1 | .757 | -.3386 | .2462 |
|  | 4 | 1 | 3.0178 | .15504 | 1 | .000 | 2.7139 | 3.3217 |
|  |  | 2 | 1.9524 | .11955 | 1 | .000 | 1.7181 | 2.1867 |
|  |  | 3 | .0462 | .14918 | 1 | .757 | -.2462 | .3386 |

| **Overall Test** | | | |
| --- | --- | --- | --- |
| Diagnostic | Wald χ² | Degrees of Freedom | P |
| 1 | 657.484 | 3 | .000 |
| 2 | 731.262 | 3 | .000 |

**Estimated Marginal Means 16：Age* Diagnostic**

| **Estimate** | | | | | |
| --- | --- | --- | --- | --- | --- |
| Age | Diagnostic | Mean | Standard Error | 95% Wald Confidence Interval | |
|  |  |  |  | Lower Bound | Upper Bound |
| 1 | 1 | 5.3615 | .06209 | 5.2411 | 5.4845 |
|  | 2 | 3.6025 | .10835 | 3.3963 | 3.8213 |
| 2 | 1 | 4.0922 | .02810 | 4.0375 | 4.1477 |
|  | 2 | 4.6680 | .04427 | 4.5820 | 4.7555 |
| 3 | 1 | 5.3073 | .07451 | 5.1633 | 5.4554 |
|  | 2 | 6.5741 | .10430 | 6.3728 | 6.7817 |
| 4 | 1 | 5.4259 | .09701 | 5.2390 | 5.6194 |
|  | 2 | 6.6203 | .11908 | 6.3910 | 6.8579 |

| **Pairwise Comparisons** | | | | | | | | |
| --- | --- | --- | --- | --- | --- | --- | --- | --- |
| Age | (I) Diagnostic | (J) Diagnostic | Mean Difference (I-J) | Standard Error | Degrees of Freedom | P | 95% Wald Confidence Interval | |
|  |  |  |  |  |  |  | Lower Bound | Upper Bound |
| 1 | 1 | 2 | 1.7589 | .12140 | 1 | .000 | 1.5210 | 1.9969 |
|  | 2 | 1 | -1.7589 | .12140 | 1 | .000 | -1.9969 | -1.5210 |
| 2 | 1 | 2 | -.5757 | .04963 | 1 | .000 | -.6730 | -.4784 |
|  | 2 | 1 | .5757 | .04963 | 1 | .000 | .4784 | .6730 |
| 3 | 1 | 2 | -1.2668 | .08230 | 1 | .000 | -1.4281 | -1.1055 |
|  | 2 | 1 | 1.2668 | .08230 | 1 | .000 | 1.1055 | 1.4281 |
| 4 | 1 | 2 | -1.1945 | .09019 | 1 | .000 | -1.3712 | -1.0177 |
|  | 2 | 1 | 1.1945 | .09019 | 1 | .000 | 1.0177 | 1.3712 |

| **Overall Test** | | | |
| --- | --- | --- | --- |
| Age | Wald χ² | Degrees of Freedom | P |
| 1 | 209.937 | 1 | .000 |
| 2 | 134.580 | 1 | .000 |
| 3 | 236.939 | 1 | .000 |
| 4 | 175.401 | 1 | .000 |
