# Supplementary material for: The impact of COVID-19 social isolation and reduced microbial exposure on the immune system in children: a retrospective study
Source: PeerJ. 2026 Jul 7;14:e21469. doi: 10.7717/peerj.21469 (PMC13353229; doi:10.7717/peerj.21469)
Supplement: Supplemental Information 5 [file peerj-14-21469-s005.docx]

**Lymphocyte Generalized Linear Model**

For CBC analysis, patients were categorized into the following age groups:
Group 1: 0–3 months,
Group 2: 3 months–4 years,
Group 3: 4–6 years,
Group 4: 6–13 years.

Diagnostic 1 is LRTIs . Diagnostic 2 URTIs .

| **Parameter Estimate** | | | | | | | |
| --- | --- | --- | --- | --- | --- | --- | --- |
| Parameter | B | Standard Error | 95% Wald Confidence Interval | | Hypothesis Testing | | |
|  |  |  | Lower Bound | Upper Bound | Wald χ² | Degrees of Freedom | P |
| （Intercept） | .995 | .0316 | .933 | 1.057 | 989.274 | 1 | .000 |
| [Year=2020] | -.209 | .0750 | -.356 | -.062 | 7.772 | 1 | .005 |
| [Year=2021] | -.038 | .0422 | -.121 | .045 | .821 | 1 | .365 |
| [Year=2022] | -.272 | .0370 | -.345 | -.200 | 54.340 | 1 | .000 |
| [Year=2023] | -.395 | .0318 | -.457 | -.333 | 154.631 | 1 | .000 |
| [Year=2024] | -.247 | .0318 | -.310 | -.185 | 60.343 | 1 | .000 |
| [Year=2025] | 0 | . | . | . | . | . | . |
| [Male ] | .023 | .0175 | -.011 | .058 | 1.785 | 1 | .182 |
| [Female ] | 0 | . | . | . | . | . | . |
| [Age=1] | .614 | .0412 | .533 | .694 | 221.871 | 1 | .000 |
| [Age=2] | .468 | .0313 | .406 | .529 | 223.463 | 1 | .000 |
| [Age=3] | .192 | .0367 | .120 | .264 | 27.353 | 1 | .000 |
| [Age=4] | 0 | . | . | . | . | . | . |
| [LRTIs ] | .032 | .0193 | -.005 | .070 | 2.829 | 1 | .093 |
| [URTIs ] | 0 | . | . | . | . | . | . |
| [Year=2020] * [Male ] | .021 | .0334 | -.044 | .087 | .399 | 1 | .528 |
| [Year=2020] * [Female ] | 0 | . | . | . | . | . | . |
| [Year=2021] * [Male ] | -.012 | .0222 | -.055 | .032 | .282 | 1 | .595 |
| [Year=2021] * [Female ] | 0 | . | . | . | . | . | . |
| [Year=2022] * [Male ] | -.006 | .0181 | -.042 | .029 | .121 | 1 | .728 |
| [Year=2022] * [Female ] | 0 | . | . | . | . | . | . |
| [Year=2023] * [Male ] | -.010 | .0155 | -.040 | .021 | .374 | 1 | .541 |
| [Year=2023] * [Female ] | 0 | . | . | . | . | . | . |
| [Year=2024] * [Male ] | .002 | .0154 | -.028 | .032 | .022 | 1 | .882 |
| [Year=2024] * [Female ] | 0 | . | . | . | . | . | . |
| [Year=2025] * [Male ] | 0 | . | . | . | . | . | . |
| [Year=2025] * [Female ] | 0 | . | . | . | . | . | . |
| [Year=2020] * [Age=1] | -.052 | .0795 | -.208 | .104 | .424 | 1 | .515 |
| [Year=2020] * [Age=2] | -.053 | .0747 | -.199 | .094 | .497 | 1 | .481 |
| [Year=2020] * [Age=3] | -.076 | .0918 | -.256 | .103 | .693 | 1 | .405 |
| [Year=2020] * [Age=4] | 0 | . | . | . | . | . | . |
| [Year=2021] * [Age=1] | -.202 | .0500 | -.300 | -.104 | 16.356 | 1 | .000 |
| [Year=2021] * [Age=2] | -.192 | .0410 | -.273 | -.112 | 21.938 | 1 | .000 |
| [Year=2021] * [Age=3] | -.058 | .0473 | -.150 | .035 | 1.483 | 1 | .223 |
| [Year=2021] * [Age=4] | 0 | . | . | . | . | . | . |
| [Year=2022] * [Age=1] | .143 | .0439 | .057 | .230 | 10.660 | 1 | .001 |
| [Year=2022] * [Age=2] | -.080 | .0363 | -.151 | -.009 | 4.858 | 1 | .028 |
| [Year=2022] * [Age=3] | -.076 | .0416 | -.157 | .006 | 3.325 | 1 | .068 |
| [Year=2022] * [Age=4] | 0 | . | . | . | . | . | . |
| [Year=2023] * [Age=1] | .272 | .0372 | .199 | .345 | 53.684 | 1 | .000 |
| [Year=2023] * [Age=2] | -.005 | .0313 | -.067 | .056 | .027 | 1 | .869 |
| [Year=2023] * [Age=3] | -.101 | .0366 | -.173 | -.030 | 7.675 | 1 | .006 |
| [Year=2023] * [Age=4] | 0 | . | . | . | . | . | . |
| [Year=2024] * [Age=1] | .213 | .0377 | .139 | .287 | 31.908 | 1 | .000 |
| [Year=2024] * [Age=2] | -.093 | .0313 | -.154 | -.032 | 8.800 | 1 | .003 |
| [Year=2024] * [Age=3] | -.090 | .0366 | -.162 | -.018 | 6.040 | 1 | .014 |
| [Year=2024] * [Age=4] | 0 | . | . | . | . | . | . |
| [Year=2025] * [Age=1] | 0 | . | . | . | . | . | . |
| [Year=2025] * [Age=2] | 0 | . | . | . | . | . | . |
| [Year=2025] * [Age=3] | 0 | . | . | . | . | . | . |
| [Year=2025] * [Age=4] | 0 | . | . | . | . | . | . |
| [Year=2020] * [LRTIs ] | .194 | .0437 | .108 | .279 | 19.647 | 1 | .000 |
| [Year=2020] * [URTIs ] | 0 | . | . | . | . | . | . |
| [Year=2021] * [LRTIs ] | .173 | .0243 | .125 | .220 | 50.336 | 1 | .000 |
| [Year=2021] * [URTIs ] | 0 | . | . | . | . | . | . |
| [Year=2022] * [LRTIs ] | .157 | .0202 | .117 | .197 | 60.244 | 1 | .000 |
| [Year=2022] * [URTIs ] | 0 | . | . | . | . | . | . |
| [Year=2023] * [LRTIs ] | .207 | .0178 | .172 | .242 | 135.403 | 1 | .000 |
| [Year=2023] * [URTIs ] | 0 | . | . | . | . | . | . |
| [Year=2024] * [LRTIs ] | .179 | .0177 | .144 | .213 | 102.480 | 1 | .000 |
| [Year=2024] * [URTIs ] | 0 | . | . | . | . | . | . |
| [Year=2025] * [LRTIs ] | 0 | . | . | . | . | . | . |
| [Year=2025] * [URTIs ] | 0 | . | . | . | . | . | . |
| [Male ] * [Age=1] | -.071 | .0182 | -.106 | -.035 | 15.005 | 1 | .000 |
| [Male ] * [Age=2] | -.012 | .0098 | -.032 | .007 | 1.594 | 1 | .207 |
| [Male ] * [Age=3] | -.045 | .0105 | -.065 | -.024 | 17.935 | 1 | .000 |
| [Male ] * [Age=4] | 0 | . | . | . | . | . | . |
| [Female ] * [Age=1] | 0 | . | . | . | . | . | . |
| [Female ] * [Age=2] | 0 | . | . | . | . | . | . |
| [Female ] * [Age=3] | 0 | . | . | . | . | . | . |
| [Female ] * [Age=4] | 0 | . | . | . | . | . | . |
| [Male ] * [LRTIs ] | -.008 | .0074 | -.023 | .006 | 1.253 | 1 | .263 |
| [Male ] * [URTIs ] | 0 | . | . | . | . | . | . |
| [Female ] * [LRTIs ] | 0 | . | . | . | . | . | . |
| [Female ] * [URTIs ] | 0 | . | . | . | . | . | . |
| [Age=1] * [LRTIs ] | -.130 | .0253 | -.180 | -.081 | 26.579 | 1 | .000 |
| [Age=1] * [URTIs ] | 0 | . | . | . | . | . | . |
| [Age=2] * [LRTIs ] | .057 | .0100 | .037 | .076 | 32.436 | 1 | .000 |
| [Age=2] * [URTIs ] | 0 | . | . | . | . | . | . |
| [Age=3] * [LRTIs ] | .029 | .0108 | .008 | .050 | 7.391 | 1 | .007 |
| [Age=3] * [URTIs ] | 0 | . | . | . | . | . | . |
| [Age=4] * [LRTIs ] | 0 | . | . | . | . | . | . |
| [Age=4] * [URTIs ] | 0 | . | . | . | . | . | . |
| （标度） | .247 | .0012 | .245 | .249 |  |  |  |

**Estimated Marginal Means 1：Year**

| **Estimate** | | | | |
| --- | --- | --- | --- | --- |
| Year | Mean | Standard Error | 95% Wald Confidence Interval | |
|  |  |  | Lower Bound | Upper Bound |
| 2020 | 3.2248 | .07752 | 3.0764 | 3.3804 |
| 2021 | 3.4802 | .03992 | 3.4028 | 3.5593 |
| 2022 | 3.0580 | .02552 | 3.0084 | 3.1085 |
| 2023 | 2.8949 | .01465 | 2.8664 | 2.9238 |
| 2024 | 3.2176 | .01662 | 3.1852 | 3.2503 |
| 2025 | 3.7357 | .03980 | 3.6585 | 3.8145 |

| **Pairwise Comparisons** | | | | | | | |
| --- | --- | --- | --- | --- | --- | --- | --- |
| (I) Year | (J) Year | Mean Difference (I-J) | Standard Error | Degrees of Freedom | P | 95% Wald Confidence Interval | |
|  |  |  |  |  |  | Lower Bound | Upper Bound |
| 2020 | 2021 | -.2554 | .08642 | 1 | .003 | -.4248 | -.0860 |
|  | 2022 | .1667 | .08101 | 1 | .040 | .0080 | .3255 |
|  | 2023 | .3299 | .07831 | 1 | .000 | .1764 | .4833 |
|  | 2024 | .0072 | .07872 | 1 | .927 | -.1471 | .1615 |
|  | 2025 | -.5109 | .08606 | 1 | .000 | -.6796 | -.3422 |
| 2021 | 2020 | .2554 | .08642 | 1 | .003 | .0860 | .4248 |
|  | 2022 | .4222 | .04650 | 1 | .000 | .3310 | .5133 |
|  | 2023 | .5853 | .04159 | 1 | .000 | .5038 | .6668 |
|  | 2024 | .2626 | .04234 | 1 | .000 | .1796 | .3456 |
|  | 2025 | -.2555 | .05500 | 1 | .000 | -.3633 | -.1477 |
| 2022 | 2020 | -.1667 | .08101 | 1 | .040 | -.3255 | -.0080 |
|  | 2021 | -.4222 | .04650 | 1 | .000 | -.5133 | -.3310 |
|  | 2023 | .1631 | .02840 | 1 | .000 | .1074 | .2188 |
|  | 2024 | -.1595 | .02949 | 1 | .000 | -.2173 | -.1017 |
|  | 2025 | -.6776 | .04609 | 1 | .000 | -.7680 | -.5873 |
| 2023 | 2020 | -.3299 | .07831 | 1 | .000 | -.4833 | -.1764 |
|  | 2021 | -.5853 | .04159 | 1 | .000 | -.6668 | -.5038 |
|  | 2022 | -.1631 | .02840 | 1 | .000 | -.2188 | -.1074 |
|  | 2024 | -.3227 | .02087 | 1 | .000 | -.3635 | -.2818 |
|  | 2025 | -.8408 | .04115 | 1 | .000 | -.9214 | -.7601 |
| 2024 | 2020 | -.0072 | .07872 | 1 | .927 | -.1615 | .1471 |
|  | 2021 | -.2626 | .04234 | 1 | .000 | -.3456 | -.1796 |
|  | 2022 | .1595 | .02949 | 1 | .000 | .1017 | .2173 |
|  | 2023 | .3227 | .02087 | 1 | .000 | .2818 | .3635 |
|  | 2025 | -.5181 | .04192 | 1 | .000 | -.6003 | -.4359 |
| 2025 | 2020 | .5109 | .08606 | 1 | .000 | .3422 | .6796 |
|  | 2021 | .2555 | .05500 | 1 | .000 | .1477 | .3633 |
|  | 2022 | .6776 | .04609 | 1 | .000 | .5873 | .7680 |
|  | 2023 | .8408 | .04115 | 1 | .000 | .7601 | .9214 |
|  | 2024 | .5181 | .04192 | 1 | .000 | .4359 | .6003 |

| **Overall Test** | | |
| --- | --- | --- |
| Wald χ² | Degrees of Freedom | P |
| 645.921 | 5 | .000 |

**Estimated Marginal Means 2：Gender**

| **Estimate** | | | | |
| --- | --- | --- | --- | --- |
| Gender | Mean | Standard Error | 95% Wald Confidence Interval | |
|  |  |  | Lower Bound | Upper Bound |
| 1 | 3.2355 | .01994 | 3.1967 | 3.2748 |
| 2 | 3.2791 | .02279 | 3.2347 | 3.3240 |

| **Pairwise Comparisons** | | | | | | | |
| --- | --- | --- | --- | --- | --- | --- | --- |
| (I) Gender | (J) Gender | Mean Difference (I-J) | Standard Error | Degrees of Freedom | P | 95% Wald Confidence Interval | |
|  |  |  |  |  |  | Lower Bound | Upper Bound |
| 1 | 2 | -.0435 | .02363 | 1 | .065 | -.0899 | .0028 |
| 2 | 1 | .0435 | .02363 | 1 | .065 | -.0028 | .0899 |

| **Overall Test** | | |
| --- | --- | --- |
| Wald χ² | Degrees of Freedom | P |
| 3.395 | 1 | .065 |

**Estimated Marginal Means 3：Age**

| **Estimate** | | | | |
| --- | --- | --- | --- | --- |
| Age | Mean | Standard Error | 95% Wald Confidence Interval | |
|  |  |  | Lower Bound | Upper Bound |
| 1 | 4.3866 | .05618 | 4.2778 | 4.4981 |
| 2 | 3.7512 | .01823 | 3.7157 | 3.7871 |
| 3 | 2.7733 | .02927 | 2.7165 | 2.8313 |
| 4 | 2.4666 | .03173 | 2.4052 | 2.5296 |

| **Pairwise Comparisons** | | | | | | | |
| --- | --- | --- | --- | --- | --- | --- | --- |
| (I) Age | (J) Age | Mean Difference (I-J) | Standard Error | Degrees of Freedom | P | 95% Wald Confidence Interval | |
|  |  |  |  |  |  | Lower Bound | Upper Bound |
| 1 | 2 | .6353 | .05754 | 1 | .000 | .5225 | .7481 |
|  | 3 | 1.6132 | .06254 | 1 | .000 | 1.4907 | 1.7358 |
|  | 4 | 1.9200 | .06491 | 1 | .000 | 1.7927 | 2.0472 |
| 2 | 1 | -.6353 | .05754 | 1 | .000 | -.7481 | -.5225 |
|  | 3 | .9779 | .03384 | 1 | .000 | .9116 | 1.0443 |
|  | 4 | 1.2846 | .03681 | 1 | .000 | 1.2125 | 1.3568 |
| 3 | 1 | -1.6132 | .06254 | 1 | .000 | -1.7358 | -1.4907 |
|  | 2 | -.9779 | .03384 | 1 | .000 | -1.0443 | -.9116 |
|  | 4 | .3067 | .04326 | 1 | .000 | .2219 | .3915 |
| 4 | 1 | -1.9200 | .06491 | 1 | .000 | -2.0472 | -1.7927 |
|  | 2 | -1.2846 | .03681 | 1 | .000 | -1.3568 | -1.2125 |
|  | 3 | -.3067 | .04326 | 1 | .000 | -.3915 | -.2219 |

| **Overall Test** | | |
| --- | --- | --- |
| Wald χ² | Degrees of Freedom | P |
| 1951.584 | 3 | .000 |

**Estimated Marginal Means 4：Diagnostic**

| **Estimate** | | | | |
| --- | --- | --- | --- | --- |
| Diagnostic | Mean | Standard Error | 95% Wald Confidence Interval | |
|  |  |  | Lower Bound | Upper Bound |
| 1 | 3.5439 | .01973 | 3.5054 | 3.5828 |
| 2 | 2.9937 | .02649 | 2.9423 | 3.0461 |

| **Pairwise Comparisons** | | | | | | | |
| --- | --- | --- | --- | --- | --- | --- | --- |
| (I) Diagnostic | (J) Diagnostic | Mean Difference (I-J) | Standard Error | Degrees of Freedom | P | 95% Wald Confidence Interval | |
|  |  |  |  |  |  | Lower Bound | Upper Bound |
| 1 | 2 | .5502 | .03125 | 1 | .000 | .4889 | .6114 |
| 2 | 1 | -.5502 | .03125 | 1 | .000 | -.6114 | -.4889 |

| **Overall Test** | | |
| --- | --- | --- |
| Wald χ² | Degrees of Freedom | P |
| 309.935 | 1 | .000 |

**Estimated Marginal Means 5：Year* Gender**

| **Estimate** | | | | | |
| --- | --- | --- | --- | --- | --- |
| Year | Gender | Mean | Standard Error | 95% Wald Confidence Interval | |
|  |  |  |  | Lower Bound | Upper Bound |
| 2020 | 1 | 3.2384 | .08528 | 3.0755 | 3.4099 |
|  | 2 | 3.2112 | .09808 | 3.0247 | 3.4093 |
| 2021 | 1 | 3.4379 | .04722 | 3.3466 | 3.5317 |
|  | 2 | 3.5231 | .05337 | 3.4200 | 3.6292 |
| 2022 | 1 | 3.0292 | .02943 | 2.9721 | 3.0875 |
|  | 2 | 3.0871 | .03320 | 3.0227 | 3.1529 |
| 2023 | 1 | 2.8630 | .01682 | 2.8302 | 2.8962 |
|  | 2 | 2.9272 | .01865 | 2.8909 | 2.9640 |
| 2024 | 1 | 3.2009 | .01897 | 3.1640 | 3.2383 |
|  | 2 | 3.2343 | .02086 | 3.1937 | 3.2754 |
| 2025 | 1 | 3.7121 | .04504 | 3.6249 | 3.8015 |
|  | 2 | 3.7594 | .05015 | 3.6624 | 3.8590 |

| **Pairwise Comparisons** | | | | | | | | |
| --- | --- | --- | --- | --- | --- | --- | --- | --- |
| Gender | (I) Year | (J) Year | Mean Difference (I-J) | Standard Error | Degrees of Freedom | P | 95% Wald Confidence Interval | |
|  |  |  |  |  |  |  | Lower Bound | Upper Bound |
| 1 | 2020 | 2021 | -.1995 | .09680 | 1 | .039 | -.3892 | -.0098 |
|  |  | 2022 | .2092 | .08965 | 1 | .020 | .0335 | .3849 |
|  |  | 2023 | .3754 | .08644 | 1 | .000 | .2060 | .5448 |
|  |  | 2024 | .0375 | .08688 | 1 | .666 | -.1328 | .2077 |
|  |  | 2025 | -.4737 | .09533 | 1 | .000 | -.6606 | -.2869 |
|  | 2021 | 2020 | .1995 | .09680 | 1 | .039 | .0098 | .3892 |
|  |  | 2022 | .4087 | .05445 | 1 | .000 | .3019 | .5154 |
|  |  | 2023 | .5749 | .04892 | 1 | .000 | .4790 | .6707 |
|  |  | 2024 | .2369 | .04961 | 1 | .000 | .1397 | .3342 |
|  |  | 2025 | -.2742 | .06389 | 1 | .000 | -.3995 | -.1490 |
|  | 2022 | 2020 | -.2092 | .08965 | 1 | .020 | -.3849 | -.0335 |
|  |  | 2021 | -.4087 | .05445 | 1 | .000 | -.5154 | -.3019 |
|  |  | 2023 | .1662 | .03244 | 1 | .000 | .1026 | .2298 |
|  |  | 2024 | -.1717 | .03348 | 1 | .000 | -.2373 | -.1061 |
|  |  | 2025 | -.6829 | .05251 | 1 | .000 | -.7858 | -.5800 |
|  | 2023 | 2020 | -.3754 | .08644 | 1 | .000 | -.5448 | -.2060 |
|  |  | 2021 | -.5749 | .04892 | 1 | .000 | -.6707 | -.4790 |
|  |  | 2022 | -.1662 | .03244 | 1 | .000 | -.2298 | -.1026 |
|  |  | 2024 | -.3379 | .02328 | 1 | .000 | -.3835 | -.2923 |
|  |  | 2025 | -.8491 | .04687 | 1 | .000 | -.9410 | -.7572 |
|  | 2024 | 2020 | -.0375 | .08688 | 1 | .666 | -.2077 | .1328 |
|  |  | 2021 | -.2369 | .04961 | 1 | .000 | -.3342 | -.1397 |
|  |  | 2022 | .1717 | .03348 | 1 | .000 | .1061 | .2373 |
|  |  | 2023 | .3379 | .02328 | 1 | .000 | .2923 | .3835 |
|  |  | 2025 | -.5112 | .04763 | 1 | .000 | -.6045 | -.4178 |
|  | 2025 | 2020 | .4737 | .09533 | 1 | .000 | .2869 | .6606 |
|  |  | 2021 | .2742 | .06389 | 1 | .000 | .1490 | .3995 |
|  |  | 2022 | .6829 | .05251 | 1 | .000 | .5800 | .7858 |
|  |  | 2023 | .8491 | .04687 | 1 | .000 | .7572 | .9410 |
|  |  | 2024 | .5112 | .04763 | 1 | .000 | .4178 | .6045 |
| 2 | 2020 | 2021 | -.3118 | .11093 | 1 | .005 | -.5293 | -.0944 |
|  |  | 2022 | .1241 | .10302 | 1 | .228 | -.0778 | .3260 |
|  |  | 2023 | .2840 | .09951 | 1 | .004 | .0890 | .4791 |
|  |  | 2024 | -.0231 | .09992 | 1 | .817 | -.2189 | .1728 |
|  |  | 2025 | -.5482 | .10880 | 1 | .000 | -.7614 | -.3349 |
|  | 2021 | 2020 | .3118 | .11093 | 1 | .005 | .0944 | .5293 |
|  |  | 2022 | .4359 | .06151 | 1 | .000 | .3154 | .5565 |
|  |  | 2023 | .5959 | .05524 | 1 | .000 | .4876 | .7041 |
|  |  | 2024 | .2887 | .05588 | 1 | .000 | .1792 | .3983 |
|  |  | 2025 | -.2363 | .07183 | 1 | .001 | -.3771 | -.0956 |
|  | 2022 | 2020 | -.1241 | .10302 | 1 | .228 | -.3260 | .0778 |
|  |  | 2021 | -.4359 | .06151 | 1 | .000 | -.5565 | -.3154 |
|  |  | 2023 | .1599 | .03626 | 1 | .000 | .0889 | .2310 |
|  |  | 2024 | -.1472 | .03720 | 1 | .000 | -.2201 | -.0743 |
|  |  | 2025 | -.6723 | .05888 | 1 | .000 | -.7877 | -.5569 |
|  | 2023 | 2020 | -.2840 | .09951 | 1 | .004 | -.4791 | -.0890 |
|  |  | 2021 | -.5959 | .05524 | 1 | .000 | -.7041 | -.4876 |
|  |  | 2022 | -.1599 | .03626 | 1 | .000 | -.2310 | -.0889 |
|  |  | 2024 | -.3071 | .02522 | 1 | .000 | -.3566 | -.2577 |
|  |  | 2025 | -.8322 | .05247 | 1 | .000 | -.9351 | -.7294 |
|  | 2024 | 2020 | .0231 | .09992 | 1 | .817 | -.1728 | .2189 |
|  |  | 2021 | -.2887 | .05588 | 1 | .000 | -.3983 | -.1792 |
|  |  | 2022 | .1472 | .03720 | 1 | .000 | .0743 | .2201 |
|  |  | 2023 | .3071 | .02522 | 1 | .000 | .2577 | .3566 |
|  |  | 2025 | -.5251 | .05319 | 1 | .000 | -.6293 | -.4208 |
|  | 2025 | 2020 | .5482 | .10880 | 1 | .000 | .3349 | .7614 |
|  |  | 2021 | .2363 | .07183 | 1 | .001 | .0956 | .3771 |
|  |  | 2022 | .6723 | .05888 | 1 | .000 | .5569 | .7877 |
|  |  | 2023 | .8322 | .05247 | 1 | .000 | .7294 | .9351 |
|  |  | 2024 | .5251 | .05319 | 1 | .000 | .4208 | .6293 |

| **Overall Test** | | | |
| --- | --- | --- | --- |
| Gender | Wald χ² | Degrees of Freedom | P |
| 1 | 514.288 | 5 | .000 |
| 2 | 391.817 | 5 | .000 |

**Estimated Marginal Means 6：Year* Gender**

| **Estimate** | | | | | |
| --- | --- | --- | --- | --- | --- |
| Year | Gender | Mean | Standard Error | 95% Wald Confidence Interval | |
|  |  |  |  | Lower Bound | Upper Bound |
| 2020 | 1 | 3.2384 | .08528 | 3.0755 | 3.4099 |
|  | 2 | 3.2112 | .09808 | 3.0247 | 3.4093 |
| 2021 | 1 | 3.4379 | .04722 | 3.3466 | 3.5317 |
|  | 2 | 3.5231 | .05337 | 3.4200 | 3.6292 |
| 2022 | 1 | 3.0292 | .02943 | 2.9721 | 3.0875 |
|  | 2 | 3.0871 | .03320 | 3.0227 | 3.1529 |
| 2023 | 1 | 2.8630 | .01682 | 2.8302 | 2.8962 |
|  | 2 | 2.9272 | .01865 | 2.8909 | 2.9640 |
| 2024 | 1 | 3.2009 | .01897 | 3.1640 | 3.2383 |
|  | 2 | 3.2343 | .02086 | 3.1937 | 3.2754 |
| 2025 | 1 | 3.7121 | .04504 | 3.6249 | 3.8015 |
|  | 2 | 3.7594 | .05015 | 3.6624 | 3.8590 |

| **Pairwise Comparisons** | | | | | | | | |
| --- | --- | --- | --- | --- | --- | --- | --- | --- |
| Year | (I) Gender | (J) Gender | Mean Difference (I-J) | Standard Error | Degrees of Freedom | P | 95% Wald Confidence Interval | |
|  |  |  |  |  |  |  | Lower Bound | Upper Bound |
| 2020 | 1 | 2 | .0272 | .09883 | 1 | .784 | -.1666 | .2209 |
|  | 2 | 1 | -.0272 | .09883 | 1 | .784 | -.2209 | .1666 |
| 2021 | 1 | 2 | -.0852 | .06139 | 1 | .165 | -.2055 | .0351 |
|  | 2 | 1 | .0852 | .06139 | 1 | .165 | -.0351 | .2055 |
| 2022 | 1 | 2 | -.0579 | .03645 | 1 | .112 | -.1294 | .0135 |
|  | 2 | 1 | .0579 | .03645 | 1 | .112 | -.0135 | .1294 |
| 2023 | 1 | 2 | -.0642 | .02006 | 1 | .001 | -.1035 | -.0249 |
|  | 2 | 1 | .0642 | .02006 | 1 | .001 | .0249 | .1035 |
| 2024 | 1 | 2 | -.0334 | .02201 | 1 | .129 | -.0765 | .0098 |
|  | 2 | 1 | .0334 | .02201 | 1 | .129 | -.0098 | .0765 |
| 2025 | 1 | 2 | -.0473 | .05242 | 1 | .367 | -.1500 | .0554 |
|  | 2 | 1 | .0473 | .05242 | 1 | .367 | -.0554 | .1500 |

| **Overall Test** | | | |
| --- | --- | --- | --- |
| Year | Wald χ² | Degrees of Freedom | P |
| 2020 | .075 | 1 | .784 |
| 2021 | 1.926 | 1 | .165 |
| 2022 | 2.525 | 1 | .112 |
| 2023 | 10.236 | 1 | .001 |
| 2024 | 2.301 | 1 | .129 |
| 2025 | .814 | 1 | .367 |

**Estimated Marginal Means 7：Year* Age**

| **Estimate** | | | | | |
| --- | --- | --- | --- | --- | --- |
| Year | Age | Mean | Standard Error | 95% Wald Confidence Interval | |
|  |  |  |  | Lower Bound | Upper Bound |
| 2020 | 1 | 3.9780 | .13423 | 3.7234 | 4.2500 |
|  | 2 | 3.8820 | .08925 | 3.7109 | 4.0609 |
|  | 3 | 2.7928 | .15362 | 2.5073 | 3.1107 |
|  | 4 | 2.5076 | .15987 | 2.2130 | 2.8413 |
| 2021 | 1 | 3.9523 | .11861 | 3.7266 | 4.1918 |
|  | 2 | 3.8999 | .04451 | 3.8136 | 3.9881 |
|  | 3 | 3.2864 | .06071 | 3.1695 | 3.4075 |
|  | 4 | 2.8960 | .07293 | 2.7565 | 3.0425 |
| 2022 | 1 | 4.3966 | .10507 | 4.1954 | 4.6074 |
|  | 2 | 3.4344 | .02574 | 3.3843 | 3.4852 |
|  | 3 | 2.5404 | .02758 | 2.4869 | 2.5950 |
|  | 4 | 2.2798 | .04199 | 2.1990 | 2.3636 |
| 2023 | 1 | 4.5281 | .08117 | 4.3717 | 4.6900 |
|  | 2 | 3.3512 | .01530 | 3.3213 | 3.3813 |
|  | 3 | 2.2422 | .01176 | 2.2193 | 2.2654 |
|  | 4 | 2.0642 | .01223 | 2.0404 | 2.0883 |
| 2024 | 1 | 4.9067 | .09001 | 4.7334 | 5.0863 |
|  | 2 | 3.5296 | .01445 | 3.5013 | 3.5580 |
|  | 3 | 2.6075 | .01333 | 2.5815 | 2.6337 |
|  | 4 | 2.3735 | .01520 | 2.3439 | 2.4034 |
| 2025 | 1 | 4.6387 | .08720 | 4.4709 | 4.8128 |
|  | 2 | 4.5308 | .04363 | 4.4460 | 4.6171 |
|  | 3 | 3.3376 | .07059 | 3.2021 | 3.4789 |
|  | 4 | 2.7764 | .07998 | 2.6240 | 2.9377 |

| **Pairwise Comparisons** | | | | | | | | |
| --- | --- | --- | --- | --- | --- | --- | --- | --- |
| Age | (I) Year | (J) Year | Mean Difference (I-J) | Standard Error | Degrees of Freedom | P | 95% Wald Confidence Interval | |
|  |  |  |  |  |  |  | Lower Bound | Upper Bound |
| 1 | 2020 | 2021 | .0257 | .17019 | 1 | .880 | -.3079 | .3592 |
|  |  | 2022 | -.4186 | .16161 | 1 | .010 | -.7354 | -.1018 |
|  |  | 2023 | -.5500 | .14695 | 1 | .000 | -.8381 | -.2620 |
|  |  | 2024 | -.9287 | .15248 | 1 | .000 | -1.2276 | -.6298 |
|  |  | 2025 | -.6607 | .14491 | 1 | .000 | -.9447 | -.3766 |
|  | 2021 | 2020 | -.0257 | .17019 | 1 | .880 | -.3592 | .3079 |
|  |  | 2022 | -.4443 | .15111 | 1 | .003 | -.7404 | -.1481 |
|  |  | 2023 | -.5757 | .13536 | 1 | .000 | -.8410 | -.3104 |
|  |  | 2024 | -.9544 | .14124 | 1 | .000 | -1.2312 | -.6775 |
|  |  | 2025 | -.6863 | .13452 | 1 | .000 | -.9500 | -.4227 |
|  | 2022 | 2020 | .4186 | .16161 | 1 | .010 | .1018 | .7354 |
|  |  | 2021 | .4443 | .15111 | 1 | .003 | .1481 | .7404 |
|  |  | 2023 | -.1315 | .12408 | 1 | .289 | -.3746 | .1117 |
|  |  | 2024 | -.5101 | .13042 | 1 | .000 | -.7657 | -.2545 |
|  |  | 2025 | -.2421 | .12363 | 1 | .050 | -.4844 | .0003 |
|  | 2023 | 2020 | .5500 | .14695 | 1 | .000 | .2620 | .8381 |
|  |  | 2021 | .5757 | .13536 | 1 | .000 | .3104 | .8410 |
|  |  | 2022 | .1315 | .12408 | 1 | .289 | -.1117 | .3746 |
|  |  | 2024 | -.3787 | .11178 | 1 | .001 | -.5977 | -.1596 |
|  |  | 2025 | -.1106 | .10368 | 1 | .286 | -.3138 | .0926 |
|  | 2024 | 2020 | .9287 | .15248 | 1 | .000 | .6298 | 1.2276 |
|  |  | 2021 | .9544 | .14124 | 1 | .000 | .6775 | 1.2312 |
|  |  | 2022 | .5101 | .13042 | 1 | .000 | .2545 | .7657 |
|  |  | 2023 | .3787 | .11178 | 1 | .001 | .1596 | .5977 |
|  |  | 2025 | .2680 | .11149 | 1 | .016 | .0495 | .4866 |
|  | 2025 | 2020 | .6607 | .14491 | 1 | .000 | .3766 | .9447 |
|  |  | 2021 | .6863 | .13452 | 1 | .000 | .4227 | .9500 |
|  |  | 2022 | .2421 | .12363 | 1 | .050 | -.0003 | .4844 |
|  |  | 2023 | .1106 | .10368 | 1 | .286 | -.0926 | .3138 |
|  |  | 2024 | -.2680 | .11149 | 1 | .016 | -.4866 | -.0495 |
| 2 | 2020 | 2021 | -.0179 | .09971 | 1 | .858 | -.2133 | .1776 |
|  |  | 2022 | .4476 | .09286 | 1 | .000 | .2656 | .6296 |
|  |  | 2023 | .5308 | .09053 | 1 | .000 | .3534 | .7082 |
|  |  | 2024 | .3524 | .09039 | 1 | .000 | .1753 | .5296 |
|  |  | 2025 | -.6488 | .09925 | 1 | .000 | -.8433 | -.4543 |
|  | 2021 | 2020 | .0179 | .09971 | 1 | .858 | -.1776 | .2133 |
|  |  | 2022 | .4654 | .05139 | 1 | .000 | .3647 | .5662 |
|  |  | 2023 | .5487 | .04704 | 1 | .000 | .4565 | .6409 |
|  |  | 2024 | .3703 | .04677 | 1 | .000 | .2786 | .4620 |
|  |  | 2025 | -.6309 | .06228 | 1 | .000 | -.7530 | -.5088 |
|  | 2022 | 2020 | -.4476 | .09286 | 1 | .000 | -.6296 | -.2656 |
|  |  | 2021 | -.4654 | .05139 | 1 | .000 | -.5662 | -.3647 |
|  |  | 2023 | .0832 | .02990 | 1 | .005 | .0246 | .1418 |
|  |  | 2024 | -.0951 | .02947 | 1 | .001 | -.1529 | -.0374 |
|  |  | 2025 | -1.0963 | .05060 | 1 | .000 | -1.1955 | -.9972 |
|  | 2023 | 2020 | -.5308 | .09053 | 1 | .000 | -.7082 | -.3534 |
|  |  | 2021 | -.5487 | .04704 | 1 | .000 | -.6409 | -.4565 |
|  |  | 2022 | -.0832 | .02990 | 1 | .005 | -.1418 | -.0246 |
|  |  | 2024 | -.1784 | .02098 | 1 | .000 | -.2195 | -.1372 |
|  |  | 2025 | -1.1796 | .04619 | 1 | .000 | -1.2701 | -1.0890 |
|  | 2024 | 2020 | -.3524 | .09039 | 1 | .000 | -.5296 | -.1753 |
|  |  | 2021 | -.3703 | .04677 | 1 | .000 | -.4620 | -.2786 |
|  |  | 2022 | .0951 | .02947 | 1 | .001 | .0374 | .1529 |
|  |  | 2023 | .1784 | .02098 | 1 | .000 | .1372 | .2195 |
|  |  | 2025 | -1.0012 | .04591 | 1 | .000 | -1.0912 | -.9112 |
|  | 2025 | 2020 | .6488 | .09925 | 1 | .000 | .4543 | .8433 |
|  |  | 2021 | .6309 | .06228 | 1 | .000 | .5088 | .7530 |
|  |  | 2022 | 1.0963 | .05060 | 1 | .000 | .9972 | 1.1955 |
|  |  | 2023 | 1.1796 | .04619 | 1 | .000 | 1.0890 | 1.2701 |
|  |  | 2024 | 1.0012 | .04591 | 1 | .000 | .9112 | 1.0912 |
| 3 | 2020 | 2021 | -.4936 | .16511 | 1 | .003 | -.8172 | -.1700 |
|  |  | 2022 | .2524 | .15603 | 1 | .106 | -.0535 | .5582 |
|  |  | 2023 | .5505 | .15405 | 1 | .000 | .2486 | .8525 |
|  |  | 2024 | .1853 | .15414 | 1 | .229 | -.1169 | .4874 |
|  |  | 2025 | -.5448 | .16899 | 1 | .001 | -.8761 | -.2136 |
|  | 2021 | 2020 | .4936 | .16511 | 1 | .003 | .1700 | .8172 |
|  |  | 2022 | .7460 | .06656 | 1 | .000 | .6155 | .8764 |
|  |  | 2023 | 1.0442 | .06177 | 1 | .000 | .9231 | 1.1652 |
|  |  | 2024 | .6789 | .06202 | 1 | .000 | .5573 | .8004 |
|  |  | 2025 | -.0512 | .09298 | 1 | .582 | -.2334 | .1310 |
|  | 2022 | 2020 | -.2524 | .15603 | 1 | .106 | -.5582 | .0535 |
|  |  | 2021 | -.7460 | .06656 | 1 | .000 | -.8764 | -.6155 |
|  |  | 2023 | .2982 | .02990 | 1 | .000 | .2396 | .3568 |
|  |  | 2024 | -.0671 | .03046 | 1 | .028 | -.1268 | -.0074 |
|  |  | 2025 | -.7972 | .07569 | 1 | .000 | -.9456 | -.6488 |
|  | 2023 | 2020 | -.5505 | .15405 | 1 | .000 | -.8525 | -.2486 |
|  |  | 2021 | -1.0442 | .06177 | 1 | .000 | -1.1652 | -.9231 |
|  |  | 2022 | -.2982 | .02990 | 1 | .000 | -.3568 | -.2396 |
|  |  | 2024 | -.3653 | .01762 | 1 | .000 | -.3998 | -.3307 |
|  |  | 2025 | -1.0954 | .07151 | 1 | .000 | -1.2355 | -.9552 |
|  | 2024 | 2020 | -.1853 | .15414 | 1 | .229 | -.4874 | .1169 |
|  |  | 2021 | -.6789 | .06202 | 1 | .000 | -.8004 | -.5573 |
|  |  | 2022 | .0671 | .03046 | 1 | .028 | .0074 | .1268 |
|  |  | 2023 | .3653 | .01762 | 1 | .000 | .3307 | .3998 |
|  |  | 2025 | -.7301 | .07173 | 1 | .000 | -.8707 | -.5895 |
|  | 2025 | 2020 | .5448 | .16899 | 1 | .001 | .2136 | .8761 |
|  |  | 2021 | .0512 | .09298 | 1 | .582 | -.1310 | .2334 |
|  |  | 2022 | .7972 | .07569 | 1 | .000 | .6488 | .9456 |
|  |  | 2023 | 1.0954 | .07151 | 1 | .000 | .9552 | 1.2355 |
|  |  | 2024 | .7301 | .07173 | 1 | .000 | .5895 | .8707 |
| 4 | 2020 | 2021 | -.3884 | .17573 | 1 | .027 | -.7329 | -.0440 |
|  |  | 2022 | .2277 | .16526 | 1 | .168 | -.0962 | .5516 |
|  |  | 2023 | .4433 | .16034 | 1 | .006 | .1291 | .7576 |
|  |  | 2024 | .1341 | .16060 | 1 | .404 | -.1807 | .4489 |
|  |  | 2025 | -.2689 | .17870 | 1 | .132 | -.6191 | .0814 |
|  | 2021 | 2020 | .3884 | .17573 | 1 | .027 | .0440 | .7329 |
|  |  | 2022 | .6162 | .08412 | 1 | .000 | .4513 | .7811 |
|  |  | 2023 | .8318 | .07389 | 1 | .000 | .6869 | .9766 |
|  |  | 2024 | .5225 | .07441 | 1 | .000 | .3767 | .6684 |
|  |  | 2025 | .1196 | .10824 | 1 | .269 | -.0926 | .3317 |
|  | 2022 | 2020 | -.2277 | .16526 | 1 | .168 | -.5516 | .0962 |
|  |  | 2021 | -.6162 | .08412 | 1 | .000 | -.7811 | -.4513 |
|  |  | 2023 | .2156 | .04369 | 1 | .000 | .1299 | .3012 |
|  |  | 2024 | -.0936 | .04459 | 1 | .036 | -.1811 | -.0062 |
|  |  | 2025 | -.4966 | .09032 | 1 | .000 | -.6736 | -.3196 |
|  | 2023 | 2020 | -.4433 | .16034 | 1 | .006 | -.7576 | -.1291 |
|  |  | 2021 | -.8318 | .07389 | 1 | .000 | -.9766 | -.6869 |
|  |  | 2022 | -.2156 | .04369 | 1 | .000 | -.3012 | -.1299 |
|  |  | 2024 | -.3092 | .01931 | 1 | .000 | -.3471 | -.2714 |
|  |  | 2025 | -.7122 | .08091 | 1 | .000 | -.8708 | -.5536 |
|  | 2024 | 2020 | -.1341 | .16060 | 1 | .404 | -.4489 | .1807 |
|  |  | 2021 | -.5225 | .07441 | 1 | .000 | -.6684 | -.3767 |
|  |  | 2022 | .0936 | .04459 | 1 | .036 | .0062 | .1811 |
|  |  | 2023 | .3092 | .01931 | 1 | .000 | .2714 | .3471 |
|  |  | 2025 | -.4030 | .08141 | 1 | .000 | -.5625 | -.2434 |
|  | 2025 | 2020 | .2689 | .17870 | 1 | .132 | -.0814 | .6191 |
|  |  | 2021 | -.1196 | .10824 | 1 | .269 | -.3317 | .0926 |
|  |  | 2022 | .4966 | .09032 | 1 | .000 | .3196 | .6736 |
|  |  | 2023 | .7122 | .08091 | 1 | .000 | .5536 | .8708 |
|  |  | 2024 | .4030 | .08141 | 1 | .000 | .2434 | .5625 |

| **Overall Test** | | | |
| --- | --- | --- | --- |
| Age | Wald χ² | Degrees of Freedom | P |
| 1 | 68.807 | 5 | .000 |
| 2 | 760.240 | 5 | .000 |
| 3 | 818.409 | 5 | .000 |
| 4 | 407.833 | 5 | .000 |

**Estimated Marginal Means 8：Year* Age**

| **Estimate** | | | | | |
| --- | --- | --- | --- | --- | --- |
| Year | Age | Mean | Standard Error | 95% Wald Confidence Interval | |
|  |  |  |  | Lower Bound | Upper Bound |
| 2020 | 1 | 3.9780 | .13423 | 3.7234 | 4.2500 |
|  | 2 | 3.8820 | .08925 | 3.7109 | 4.0609 |
|  | 3 | 2.7928 | .15362 | 2.5073 | 3.1107 |
|  | 4 | 2.5076 | .15987 | 2.2130 | 2.8413 |
| 2021 | 1 | 3.9523 | .11861 | 3.7266 | 4.1918 |
|  | 2 | 3.8999 | .04451 | 3.8136 | 3.9881 |
|  | 3 | 3.2864 | .06071 | 3.1695 | 3.4075 |
|  | 4 | 2.8960 | .07293 | 2.7565 | 3.0425 |
| 2022 | 1 | 4.3966 | .10507 | 4.1954 | 4.6074 |
|  | 2 | 3.4344 | .02574 | 3.3843 | 3.4852 |
|  | 3 | 2.5404 | .02758 | 2.4869 | 2.5950 |
|  | 4 | 2.2798 | .04199 | 2.1990 | 2.3636 |
| 2023 | 1 | 4.5281 | .08117 | 4.3717 | 4.6900 |
|  | 2 | 3.3512 | .01530 | 3.3213 | 3.3813 |
|  | 3 | 2.2422 | .01176 | 2.2193 | 2.2654 |
|  | 4 | 2.0642 | .01223 | 2.0404 | 2.0883 |
| 2024 | 1 | 4.9067 | .09001 | 4.7334 | 5.0863 |
|  | 2 | 3.5296 | .01445 | 3.5013 | 3.5580 |
|  | 3 | 2.6075 | .01333 | 2.5815 | 2.6337 |
|  | 4 | 2.3735 | .01520 | 2.3439 | 2.4034 |
| 2025 | 1 | 4.6387 | .08720 | 4.4709 | 4.8128 |
|  | 2 | 4.5308 | .04363 | 4.4460 | 4.6171 |
|  | 3 | 3.3376 | .07059 | 3.2021 | 3.4789 |
|  | 4 | 2.7764 | .07998 | 2.6240 | 2.9377 |

| **Pairwise Comparisons** | | | | | | | | |
| --- | --- | --- | --- | --- | --- | --- | --- | --- |
| Year | (I) Age | (J) Age | Mean Difference (I-J) | Standard Error | Degrees of Freedom | P | 95% Wald Confidence Interval | |
|  |  |  |  |  |  |  | Lower Bound | Upper Bound |
| 2020 | 1 | 2 | .0960 | .14176 | 1 | .498 | -.1818 | .3739 |
|  |  | 3 | 1.1853 | .19637 | 1 | .000 | .8004 | 1.5702 |
|  |  | 4 | 1.4705 | .21301 | 1 | .000 | 1.0530 | 1.8880 |
|  | 2 | 1 | -.0960 | .14176 | 1 | .498 | -.3739 | .1818 |
|  |  | 3 | 1.0892 | .17350 | 1 | .000 | .7492 | 1.4293 |
|  |  | 4 | 1.3744 | .18487 | 1 | .000 | 1.0121 | 1.7368 |
|  | 3 | 1 | -1.1853 | .19637 | 1 | .000 | -1.5702 | -.8004 |
|  |  | 2 | -1.0892 | .17350 | 1 | .000 | -1.4293 | -.7492 |
|  |  | 4 | .2852 | .22259 | 1 | .200 | -.1511 | .7215 |
|  | 4 | 1 | -1.4705 | .21301 | 1 | .000 | -1.8880 | -1.0530 |
|  |  | 2 | -1.3744 | .18487 | 1 | .000 | -1.7368 | -1.0121 |
|  |  | 3 | -.2852 | .22259 | 1 | .200 | -.7215 | .1511 |
| 2021 | 1 | 2 | .0525 | .12596 | 1 | .677 | -.1944 | .2994 |
|  |  | 3 | .6660 | .13158 | 1 | .000 | .4081 | .9239 |
|  |  | 4 | 1.0563 | .13813 | 1 | .000 | .7856 | 1.3271 |
|  | 2 | 1 | -.0525 | .12596 | 1 | .677 | -.2994 | .1944 |
|  |  | 3 | .6135 | .07456 | 1 | .000 | .4673 | .7596 |
|  |  | 4 | 1.0039 | .08508 | 1 | .000 | .8371 | 1.1706 |
|  | 3 | 1 | -.6660 | .13158 | 1 | .000 | -.9239 | -.4081 |
|  |  | 2 | -.6135 | .07456 | 1 | .000 | -.7596 | -.4673 |
|  |  | 4 | .3904 | .09427 | 1 | .000 | .2056 | .5751 |
|  | 4 | 1 | -1.0563 | .13813 | 1 | .000 | -1.3271 | -.7856 |
|  |  | 2 | -1.0039 | .08508 | 1 | .000 | -1.1706 | -.8371 |
|  |  | 3 | -.3904 | .09427 | 1 | .000 | -.5751 | -.2056 |
| 2022 | 1 | 2 | .9622 | .10761 | 1 | .000 | .7513 | 1.1731 |
|  |  | 3 | 1.8562 | .10809 | 1 | .000 | 1.6444 | 2.0681 |
|  |  | 4 | 2.1168 | .11298 | 1 | .000 | 1.8954 | 2.3382 |
|  | 2 | 1 | -.9622 | .10761 | 1 | .000 | -1.1731 | -.7513 |
|  |  | 3 | .8940 | .03729 | 1 | .000 | .8209 | .9671 |
|  |  | 4 | 1.1546 | .04911 | 1 | .000 | 1.0583 | 1.2508 |
|  | 3 | 1 | -1.8562 | .10809 | 1 | .000 | -2.0681 | -1.6444 |
|  |  | 2 | -.8940 | .03729 | 1 | .000 | -.9671 | -.8209 |
|  |  | 4 | .2606 | .05012 | 1 | .000 | .1623 | .3588 |
|  | 4 | 1 | -2.1168 | .11298 | 1 | .000 | -2.3382 | -1.8954 |
|  |  | 2 | -1.1546 | .04911 | 1 | .000 | -1.2508 | -1.0583 |
|  |  | 3 | -.2606 | .05012 | 1 | .000 | -.3588 | -.1623 |
| 2023 | 1 | 2 | 1.1769 | .08249 | 1 | .000 | 1.0152 | 1.3385 |
|  |  | 3 | 2.2858 | .08194 | 1 | .000 | 2.1252 | 2.4464 |
|  |  | 4 | 2.4638 | .08201 | 1 | .000 | 2.3031 | 2.6246 |
|  | 2 | 1 | -1.1769 | .08249 | 1 | .000 | -1.3385 | -1.0152 |
|  |  | 3 | 1.1090 | .01924 | 1 | .000 | 1.0713 | 1.1467 |
|  |  | 4 | 1.2869 | .01953 | 1 | .000 | 1.2487 | 1.3252 |
|  | 3 | 1 | -2.2858 | .08194 | 1 | .000 | -2.4464 | -2.1252 |
|  |  | 2 | -1.1090 | .01924 | 1 | .000 | -1.1467 | -1.0713 |
|  |  | 4 | .1780 | .01692 | 1 | .000 | .1448 | .2111 |
|  | 4 | 1 | -2.4638 | .08201 | 1 | .000 | -2.6246 | -2.3031 |
|  |  | 2 | -1.2869 | .01953 | 1 | .000 | -1.3252 | -1.2487 |
|  |  | 3 | -.1780 | .01692 | 1 | .000 | -.2111 | -.1448 |
| 2024 | 1 | 2 | 1.3772 | .09105 | 1 | .000 | 1.1987 | 1.5556 |
|  |  | 3 | 2.2992 | .09085 | 1 | .000 | 2.1212 | 2.4773 |
|  |  | 4 | 2.5333 | .09119 | 1 | .000 | 2.3545 | 2.7120 |
|  | 2 | 1 | -1.3772 | .09105 | 1 | .000 | -1.5556 | -1.1987 |
|  |  | 3 | .9221 | .01954 | 1 | .000 | .8838 | .9604 |
|  |  | 4 | 1.1561 | .02089 | 1 | .000 | 1.1152 | 1.1970 |
|  | 3 | 1 | -2.2992 | .09085 | 1 | .000 | -2.4773 | -2.1212 |
|  |  | 2 | -.9221 | .01954 | 1 | .000 | -.9604 | -.8838 |
|  |  | 4 | .2340 | .02009 | 1 | .000 | .1946 | .2734 |
|  | 4 | 1 | -2.5333 | .09119 | 1 | .000 | -2.7120 | -2.3545 |
|  |  | 2 | -1.1561 | .02089 | 1 | .000 | -1.1970 | -1.1152 |
|  |  | 3 | -.2340 | .02009 | 1 | .000 | -.2734 | -.1946 |
| 2025 | 1 | 2 | .1079 | .09147 | 1 | .238 | -.0714 | .2872 |
|  |  | 3 | 1.3011 | .11003 | 1 | .000 | 1.0854 | 1.5167 |
|  |  | 4 | 1.8623 | .11869 | 1 | .000 | 1.6296 | 2.0949 |
|  | 2 | 1 | -.1079 | .09147 | 1 | .238 | -.2872 | .0714 |
|  |  | 3 | 1.1932 | .08149 | 1 | .000 | 1.0334 | 1.3529 |
|  |  | 4 | 1.7543 | .09121 | 1 | .000 | 1.5756 | 1.9331 |
|  | 3 | 1 | -1.3011 | .11003 | 1 | .000 | -1.5167 | -1.0854 |
|  |  | 2 | -1.1932 | .08149 | 1 | .000 | -1.3529 | -1.0334 |
|  |  | 4 | .5612 | .10671 | 1 | .000 | .3520 | .7703 |
|  | 4 | 1 | -1.8623 | .11869 | 1 | .000 | -2.0949 | -1.6296 |
|  |  | 2 | -1.7543 | .09121 | 1 | .000 | -1.9331 | -1.5756 |
|  |  | 3 | -.5612 | .10671 | 1 | .000 | -.7703 | -.3520 |

| **Overall Test** | | | |
| --- | --- | --- | --- |
| Year | Wald χ² | Degrees of Freedom | P |
| 2020 | 86.320 | 3 | .000 |
| 2021 | 175.206 | 3 | .000 |
| 2022 | 1030.621 | 3 | .000 |
| 2023 | 5452.763 | 3 | .000 |
| 2024 | 4079.172 | 3 | .000 |
| 2025 | 512.457 | 3 | .000 |

**Estimated Marginal Means 9：Year* Diagnostic**

| **Estimate** | | | | | |
| --- | --- | --- | --- | --- | --- |
| Year | Diagnostic | Mean | Standard Error | 95% Wald Confidence Interval | |
|  |  |  |  | Lower Bound | Upper Bound |
| 2020 | 1 | 3.5832 | .09319 | 3.4051 | 3.7706 |
|  | 2 | 2.9022 | .10515 | 2.7033 | 3.1158 |
| 2021 | 1 | 3.8268 | .04739 | 3.7350 | 3.9208 |
|  | 2 | 3.1650 | .05322 | 3.0624 | 3.2711 |
| 2022 | 1 | 3.3365 | .03028 | 3.2777 | 3.3964 |
|  | 2 | 2.8028 | .03299 | 2.7389 | 2.8682 |
| 2023 | 1 | 3.2383 | .01692 | 3.2053 | 3.2716 |
|  | 2 | 2.5880 | .01950 | 2.5500 | 2.6265 |
| 2024 | 1 | 3.5487 | .01910 | 3.5115 | 3.5864 |
|  | 2 | 2.9173 | .02195 | 2.8746 | 2.9607 |
| 2025 | 1 | 3.7680 | .04181 | 3.6869 | 3.8508 |
|  | 2 | 3.7037 | .05960 | 3.5887 | 3.8223 |

| **Pairwise Comparisons** | | | | | | | | |
| --- | --- | --- | --- | --- | --- | --- | --- | --- |
| Diagnostic | (I) Year | (J) Year | Mean Difference (I-J) | Standard Error | Degrees of Freedom | P | 95% Wald Confidence Interval | |
|  |  |  |  |  |  |  | Lower Bound | Upper Bound |
| 1 | 2020 | 2021 | -.2436 | .10443 | 1 | .020 | -.4483 | -.0389 |
|  |  | 2022 | .2467 | .09785 | 1 | .012 | .0549 | .4384 |
|  |  | 2023 | .3449 | .09472 | 1 | .000 | .1593 | .5305 |
|  |  | 2024 | .0345 | .09511 | 1 | .717 | -.1519 | .2209 |
|  |  | 2025 | -.1848 | .10189 | 1 | .070 | -.3845 | .0149 |
|  | 2021 | 2020 | .2436 | .10443 | 1 | .020 | .0389 | .4483 |
|  |  | 2022 | .4903 | .05602 | 1 | .000 | .3805 | .6001 |
|  |  | 2023 | .5885 | .05019 | 1 | .000 | .4902 | .6869 |
|  |  | 2024 | .2781 | .05090 | 1 | .000 | .1783 | .3778 |
|  |  | 2025 | .0588 | .06301 | 1 | .351 | -.0647 | .1823 |
|  | 2022 | 2020 | -.2467 | .09785 | 1 | .012 | -.4384 | -.0549 |
|  |  | 2021 | -.4903 | .05602 | 1 | .000 | -.6001 | -.3805 |
|  |  | 2023 | .0982 | .03435 | 1 | .004 | .0309 | .1656 |
|  |  | 2024 | -.2122 | .03533 | 1 | .000 | -.2814 | -.1430 |
|  |  | 2025 | -.4315 | .05137 | 1 | .000 | -.5322 | -.3308 |
|  | 2023 | 2020 | -.3449 | .09472 | 1 | .000 | -.5305 | -.1593 |
|  |  | 2021 | -.5885 | .05019 | 1 | .000 | -.6869 | -.4902 |
|  |  | 2022 | -.0982 | .03435 | 1 | .004 | -.1656 | -.0309 |
|  |  | 2024 | -.3104 | .02487 | 1 | .000 | -.3592 | -.2617 |
|  |  | 2025 | -.5297 | .04507 | 1 | .000 | -.6181 | -.4414 |
|  | 2024 | 2020 | -.0345 | .09511 | 1 | .717 | -.2209 | .1519 |
|  |  | 2021 | -.2781 | .05090 | 1 | .000 | -.3778 | -.1783 |
|  |  | 2022 | .2122 | .03533 | 1 | .000 | .1430 | .2814 |
|  |  | 2023 | .3104 | .02487 | 1 | .000 | .2617 | .3592 |
|  |  | 2025 | -.2193 | .04589 | 1 | .000 | -.3092 | -.1293 |
|  | 2025 | 2020 | .1848 | .10189 | 1 | .070 | -.0149 | .3845 |
|  |  | 2021 | -.0588 | .06301 | 1 | .351 | -.1823 | .0647 |
|  |  | 2022 | .4315 | .05137 | 1 | .000 | .3308 | .5322 |
|  |  | 2023 | .5297 | .04507 | 1 | .000 | .4414 | .6181 |
|  |  | 2024 | .2193 | .04589 | 1 | .000 | .1293 | .3092 |
| 2 | 2020 | 2021 | -.2628 | .11573 | 1 | .023 | -.4896 | -.0359 |
|  |  | 2022 | .0994 | .10837 | 1 | .359 | -.1130 | .3118 |
|  |  | 2023 | .3143 | .10520 | 1 | .003 | .1081 | .5204 |
|  |  | 2024 | -.0151 | .10551 | 1 | .886 | -.2219 | .1917 |
|  |  | 2025 | -.8014 | .11807 | 1 | .000 | -1.0328 | -.5700 |
|  | 2021 | 2020 | .2628 | .11573 | 1 | .023 | .0359 | .4896 |
|  |  | 2022 | .3622 | .05947 | 1 | .000 | .2456 | .4787 |
|  |  | 2023 | .5770 | .05347 | 1 | .000 | .4722 | .6818 |
|  |  | 2024 | .2477 | .05408 | 1 | .000 | .1417 | .3537 |
|  |  | 2025 | -.5387 | .07586 | 1 | .000 | -.6873 | -.3900 |
|  | 2022 | 2020 | -.0994 | .10837 | 1 | .359 | -.3118 | .1130 |
|  |  | 2021 | -.3622 | .05947 | 1 | .000 | -.4787 | -.2456 |
|  |  | 2023 | .2148 | .03429 | 1 | .000 | .1476 | .2820 |
|  |  | 2024 | -.1145 | .03533 | 1 | .001 | -.1838 | -.0453 |
|  |  | 2025 | -.9009 | .06426 | 1 | .000 | -1.0268 | -.7749 |
|  | 2023 | 2020 | -.3143 | .10520 | 1 | .003 | -.5204 | -.1081 |
|  |  | 2021 | -.5770 | .05347 | 1 | .000 | -.6818 | -.4722 |
|  |  | 2022 | -.2148 | .03429 | 1 | .000 | -.2820 | -.1476 |
|  |  | 2024 | -.3293 | .02361 | 1 | .000 | -.3756 | -.2831 |
|  |  | 2025 | -1.1157 | .05884 | 1 | .000 | -1.2310 | -1.0004 |
|  | 2024 | 2020 | .0151 | .10551 | 1 | .886 | -.1917 | .2219 |
|  |  | 2021 | -.2477 | .05408 | 1 | .000 | -.3537 | -.1417 |
|  |  | 2022 | .1145 | .03533 | 1 | .001 | .0453 | .1838 |
|  |  | 2023 | .3293 | .02361 | 1 | .000 | .2831 | .3756 |
|  |  | 2025 | -.7863 | .05929 | 1 | .000 | -.9025 | -.6701 |
|  | 2025 | 2020 | .8014 | .11807 | 1 | .000 | .5700 | 1.0328 |
|  |  | 2021 | .5387 | .07586 | 1 | .000 | .3900 | .6873 |
|  |  | 2022 | .9009 | .06426 | 1 | .000 | .7749 | 1.0268 |
|  |  | 2023 | 1.1157 | .05884 | 1 | .000 | 1.0004 | 1.2310 |
|  |  | 2024 | .7863 | .05929 | 1 | .000 | .6701 | .9025 |

| **Overall Test** | | | |
| --- | --- | --- | --- |
| Diagnostic | Wald χ² | Degrees of Freedom | P |
| 1 | 321.521 | 5 | .000 |
| 2 | 527.897 | 5 | .000 |

**Estimated Marginal Means 10：Year* Diagnostic**

| **Estimate** | | | | | |
| --- | --- | --- | --- | --- | --- |
| Year | Diagnostic | Mean | Standard Error | 95% Wald Confidence Interval | |
|  |  |  |  | Lower Bound | Upper Bound |
| 2020 | 1 | 3.5832 | .09319 | 3.4051 | 3.7706 |
|  | 2 | 2.9022 | .10515 | 2.7033 | 3.1158 |
| 2021 | 1 | 3.8268 | .04739 | 3.7350 | 3.9208 |
|  | 2 | 3.1650 | .05322 | 3.0624 | 3.2711 |
| 2022 | 1 | 3.3365 | .03028 | 3.2777 | 3.3964 |
|  | 2 | 2.8028 | .03299 | 2.7389 | 2.8682 |
| 2023 | 1 | 3.2383 | .01692 | 3.2053 | 3.2716 |
|  | 2 | 2.5880 | .01950 | 2.5500 | 2.6265 |
| 2024 | 1 | 3.5487 | .01910 | 3.5115 | 3.5864 |
|  | 2 | 2.9173 | .02195 | 2.8746 | 2.9607 |
| 2025 | 1 | 3.7680 | .04181 | 3.6869 | 3.8508 |
|  | 2 | 3.7037 | .05960 | 3.5887 | 3.8223 |

| **Pairwise Comparisons** | | | | | | | | |
| --- | --- | --- | --- | --- | --- | --- | --- | --- |
| Year | (I) Diagnostic | (J) Diagnostic | Mean Difference (I-J) | Standard Error | Degrees of Freedom | P | 95% Wald Confidence Interval | |
|  |  |  |  |  |  |  | Lower Bound | Upper Bound |
| 2020 | 1 | 2 | .6809 | .12802 | 1 | .000 | .4300 | .9319 |
|  | 2 | 1 | -.6809 | .12802 | 1 | .000 | -.9319 | -.4300 |
| 2021 | 1 | 2 | .6618 | .06315 | 1 | .000 | .5380 | .7856 |
|  | 2 | 1 | -.6618 | .06315 | 1 | .000 | -.7856 | -.5380 |
| 2022 | 1 | 2 | .5337 | .03829 | 1 | .000 | .4587 | .6088 |
|  | 2 | 1 | -.5337 | .03829 | 1 | .000 | -.6088 | -.4587 |
| 2023 | 1 | 2 | .6503 | .02266 | 1 | .000 | .6059 | .6947 |
|  | 2 | 1 | -.6503 | .02266 | 1 | .000 | -.6947 | -.6059 |
| 2024 | 1 | 2 | .6314 | .02507 | 1 | .000 | .5823 | .6805 |
|  | 2 | 1 | -.6314 | .02507 | 1 | .000 | -.6805 | -.5823 |
| 2025 | 1 | 2 | .0643 | .06555 | 1 | .326 | -.0642 | .1928 |
|  | 2 | 1 | -.0643 | .06555 | 1 | .326 | -.1928 | .0642 |

| **Overall Test** | | | |
| --- | --- | --- | --- |
| Year | Wald χ² | Degrees of Freedom | P |
| 2020 | 28.290 | 1 | .000 |
| 2021 | 109.836 | 1 | .000 |
| 2022 | 194.285 | 1 | .000 |
| 2023 | 823.424 | 1 | .000 |
| 2024 | 634.356 | 1 | .000 |
| 2025 | .963 | 1 | .326 |

**Estimated Marginal Means 11：Gender* Age**

| **Estimate** | | | | | |
| --- | --- | --- | --- | --- | --- |
| Gender | Age | Mean | Standard Error | 95% Wald Confidence Interval | |
|  |  |  |  | Lower Bound | Upper Bound |
| 1 | 1 | 4.2739 | .06170 | 4.1547 | 4.3966 |
|  | 2 | 3.7627 | .02155 | 3.7207 | 3.8052 |
|  | 3 | 2.7373 | .03092 | 2.6774 | 2.7986 |
|  | 4 | 2.4895 | .03354 | 2.4247 | 2.5561 |
| 2 | 1 | 4.5021 | .07035 | 4.3663 | 4.6421 |
|  | 2 | 3.7398 | .02483 | 3.6914 | 3.7888 |
|  | 3 | 2.8097 | .03312 | 2.7456 | 2.8754 |
|  | 4 | 2.4438 | .03509 | 2.3760 | 2.5136 |

| **Pairwise Comparisons** | | | | | | | | |
| --- | --- | --- | --- | --- | --- | --- | --- | --- |
| Age | (I) Gender | (J) Gender | Mean Difference (I-J) | Standard Error | Degrees of Freedom | P | 95% Wald Confidence Interval | |
|  |  |  |  |  |  |  | Lower Bound | Upper Bound |
| 1 | 1 | 2 | -.2282 | .06956 | 1 | .001 | -.3645 | -.0918 |
|  | 2 | 1 | .2282 | .06956 | 1 | .001 | .0918 | .3645 |
| 2 | 1 | 2 | .0229 | .02887 | 1 | .427 | -.0336 | .0795 |
|  | 2 | 1 | -.0229 | .02887 | 1 | .427 | -.0795 | .0336 |
| 3 | 1 | 2 | -.0724 | .02599 | 1 | .005 | -.1233 | -.0215 |
|  | 2 | 1 | .0724 | .02599 | 1 | .005 | .0215 | .1233 |
| 4 | 1 | 2 | .0457 | .02623 | 1 | .081 | -.0057 | .0971 |
|  | 2 | 1 | -.0457 | .02623 | 1 | .081 | -.0971 | .0057 |

| **Overall Test** | | | |
| --- | --- | --- | --- |
| Age | Wald χ² | Degrees of Freedom | P |
| 1 | 10.761 | 1 | .001 |
| 2 | .631 | 1 | .427 |
| 3 | 7.762 | 1 | .005 |
| 4 | 3.035 | 1 | .081 |

**Estimated Marginal Means 12：Gender* Age**

| **Estimate** | | | | | |
| --- | --- | --- | --- | --- | --- |
| Gender | Age | Mean | Standard Error | 95% Wald Confidence Interval | |
|  |  |  |  | Lower Bound | Upper Bound |
| 1 | 1 | 4.2739 | .06170 | 4.1547 | 4.3966 |
|  | 2 | 3.7627 | .02155 | 3.7207 | 3.8052 |
|  | 3 | 2.7373 | .03092 | 2.6774 | 2.7986 |
|  | 4 | 2.4895 | .03354 | 2.4247 | 2.5561 |
| 2 | 1 | 4.5021 | .07035 | 4.3663 | 4.6421 |
|  | 2 | 3.7398 | .02483 | 3.6914 | 3.7888 |
|  | 3 | 2.8097 | .03312 | 2.7456 | 2.8754 |
|  | 4 | 2.4438 | .03509 | 2.3760 | 2.5136 |

| **Pairwise Comparisons** | | | | | | | | |
| --- | --- | --- | --- | --- | --- | --- | --- | --- |
| Gender | (I) Age | (J) Age | Mean Difference (I-J) | Standard Error | Degrees of Freedom | P | 95% Wald Confidence Interval | |
|  |  |  |  |  |  |  | Lower Bound | Upper Bound |
| 1 | 1 | 2 | .5112 | .06363 | 1 | .000 | .3865 | .6359 |
|  |  | 3 | 1.5366 | .06801 | 1 | .000 | 1.4033 | 1.6699 |
|  |  | 4 | 1.7844 | .07046 | 1 | .000 | 1.6463 | 1.9225 |
|  | 2 | 1 | -.5112 | .06363 | 1 | .000 | -.6359 | -.3865 |
|  |  | 3 | 1.0254 | .03540 | 1 | .000 | .9560 | 1.0948 |
|  |  | 4 | 1.2732 | .03901 | 1 | .000 | 1.1967 | 1.3496 |
|  | 3 | 1 | -1.5366 | .06801 | 1 | .000 | -1.6699 | -1.4033 |
|  |  | 2 | -1.0254 | .03540 | 1 | .000 | -1.0948 | -.9560 |
|  |  | 4 | .2478 | .04475 | 1 | .000 | .1601 | .3355 |
|  | 4 | 1 | -1.7844 | .07046 | 1 | .000 | -1.9225 | -1.6463 |
|  |  | 2 | -1.2732 | .03901 | 1 | .000 | -1.3496 | -1.1967 |
|  |  | 3 | -.2478 | .04475 | 1 | .000 | -.3355 | -.1601 |
| 2 | 1 | 2 | .7623 | .07206 | 1 | .000 | .6211 | .9036 |
|  |  | 3 | 1.6924 | .07608 | 1 | .000 | 1.5433 | 1.8415 |
|  |  | 4 | 2.0583 | .07797 | 1 | .000 | 1.9055 | 2.2111 |
|  | 2 | 1 | -.7623 | .07206 | 1 | .000 | -.9036 | -.6211 |
|  |  | 3 | .9300 | .03747 | 1 | .000 | .8566 | 1.0035 |
|  |  | 4 | 1.2959 | .03998 | 1 | .000 | 1.2176 | 1.3743 |
|  | 3 | 1 | -1.6924 | .07608 | 1 | .000 | -1.8415 | -1.5433 |
|  |  | 2 | -.9300 | .03747 | 1 | .000 | -1.0035 | -.8566 |
|  |  | 4 | .3659 | .04598 | 1 | .000 | .2758 | .4560 |
|  | 4 | 1 | -2.0583 | .07797 | 1 | .000 | -2.2111 | -1.9055 |
|  |  | 2 | -1.2959 | .03998 | 1 | .000 | -1.3743 | -1.2176 |
|  |  | 3 | -.3659 | .04598 | 1 | .000 | -.4560 | -.2758 |

| **Overall Test** | | | |
| --- | --- | --- | --- |
| Gender | Wald χ² | Degrees of Freedom | P |
| 1 | 1709.317 | 3 | .000 |
| 2 | 1583.905 | 3 | .000 |

**Estimated Marginal Means 13：Gender* Diagnostic**

| **Estimate** | | | | | |
| --- | --- | --- | --- | --- | --- |
| Gender | Diagnostic | Mean | Standard Error | 95% Wald Confidence Interval | |
|  |  |  |  | Lower Bound | Upper Bound |
| 1 | 1 | 3.5130 | .02223 | 3.4697 | 3.5569 |
|  | 2 | 2.9799 | .02823 | 2.9251 | 3.0358 |
| 2 | 1 | 3.5750 | .02520 | 3.5260 | 3.6248 |
|  | 2 | 3.0076 | .03085 | 2.9477 | 3.0687 |

| **Pairwise Comparisons** | | | | | | | | |
| --- | --- | --- | --- | --- | --- | --- | --- | --- |
| Diagnostic | (I) Gender | (J) Gender | Mean Difference (I-J) | Standard Error | Degrees of Freedom | P | 95% Wald Confidence Interval | |
|  |  |  |  |  |  |  | Lower Bound | Upper Bound |
| 1 | 1 | 2 | -.0620 | .02642 | 1 | .019 | -.1138 | -.0102 |
|  | 2 | 1 | .0620 | .02642 | 1 | .019 | .0102 | .1138 |
| 2 | 1 | 2 | -.0277 | .02626 | 1 | .292 | -.0791 | .0238 |
|  | 2 | 1 | .0277 | .02626 | 1 | .292 | -.0238 | .0791 |

| **Overall Test** | | | |
| --- | --- | --- | --- |
| Diagnostic | Wald χ² | Degrees of Freedom | P |
| 1 | 5.505 | 1 | .019 |
| 2 | 1.110 | 1 | .292 |

**Estimated Marginal Means 14：Gender* Diagnostic**

| **Estimate** | | | | | |
| --- | --- | --- | --- | --- | --- |
| Gender | Diagnostic | Mean | Standard Error | 95% Wald Confidence Interval | |
|  |  |  |  | Lower Bound | Upper Bound |
| 1 | 1 | 3.5130 | .02223 | 3.4697 | 3.5569 |
|  | 2 | 2.9799 | .02823 | 2.9251 | 3.0358 |
| 2 | 1 | 3.5750 | .02520 | 3.5260 | 3.6248 |
|  | 2 | 3.0076 | .03085 | 2.9477 | 3.0687 |

| **Pairwise Comparisons** | | | | | | | | |
| --- | --- | --- | --- | --- | --- | --- | --- | --- |
| Gender | (I) Diagnostic | (J) Diagnostic | Mean Difference (I-J) | Standard Error | Degrees of Freedom | P | 95% Wald Confidence Interval | |
|  |  |  |  |  |  |  | Lower Bound | Upper Bound |
| 1 | 1 | 2 | .5331 | .03256 | 1 | .000 | .4693 | .5969 |
|  | 2 | 1 | -.5331 | .03256 | 1 | .000 | -.5969 | -.4693 |
| 2 | 1 | 2 | .5674 | .03426 | 1 | .000 | .5003 | .6346 |
|  | 2 | 1 | -.5674 | .03426 | 1 | .000 | -.6346 | -.5003 |

| **Overall Test** | | | |
| --- | --- | --- | --- |
| Gender | Wald χ² | Degrees of Freedom | P |
| 1 | 268.129 | 1 | .000 |
| 2 | 274.297 | 1 | .000 |

**Estimated Marginal Means 15：Age* Diagnostic**

| **Estimate** | | | | | |
| --- | --- | --- | --- | --- | --- |
| Age | Diagnostic | Mean | Standard Error | 95% Wald Confidence Interval | |
|  |  |  |  | Lower Bound | Upper Bound |
| 1 | 1 | 4.4966 | .04110 | 4.4167 | 4.5778 |
|  | 2 | 4.2792 | .10034 | 4.0870 | 4.4805 |
| 2 | 1 | 4.2221 | .02274 | 4.1777 | 4.2669 |
|  | 2 | 3.3329 | .02508 | 3.2841 | 3.3824 |
| 3 | 1 | 3.0788 | .03408 | 3.0128 | 3.1464 |
|  | 2 | 2.4981 | .03100 | 2.4381 | 2.5596 |
| 4 | 1 | 2.6986 | .03838 | 2.6244 | 2.7749 |
|  | 2 | 2.2545 | .03150 | 2.1936 | 2.3171 |

| **Pairwise Comparisons** | | | | | | | | |
| --- | --- | --- | --- | --- | --- | --- | --- | --- |
| Diagnostic | (I) Age | (J) Age | Mean Difference (I-J) | Standard Error | Degrees of Freedom | P | 95% Wald Confidence Interval | |
|  |  |  |  |  |  |  | Lower Bound | Upper Bound |
| 1 | 1 | 2 | .2745 | .04683 | 1 | .000 | .1827 | .3663 |
|  |  | 3 | 1.4177 | .05332 | 1 | .000 | 1.3132 | 1.5222 |
|  |  | 4 | 1.7980 | .05611 | 1 | .000 | 1.6880 | 1.9079 |
|  | 2 | 1 | -.2745 | .04683 | 1 | .000 | -.3663 | -.1827 |
|  |  | 3 | 1.1432 | .03917 | 1 | .000 | 1.0665 | 1.2200 |
|  |  | 4 | 1.5235 | .04204 | 1 | .000 | 1.4411 | 1.6059 |
|  | 3 | 1 | -1.4177 | .05332 | 1 | .000 | -1.5222 | -1.3132 |
|  |  | 2 | -1.1432 | .03917 | 1 | .000 | -1.2200 | -1.0665 |
|  |  | 4 | .3802 | .04918 | 1 | .000 | .2838 | .4766 |
|  | 4 | 1 | -1.7980 | .05611 | 1 | .000 | -1.9079 | -1.6880 |
|  |  | 2 | -1.5235 | .04204 | 1 | .000 | -1.6059 | -1.4411 |
|  |  | 3 | -.3802 | .04918 | 1 | .000 | -.4766 | -.2838 |
| 2 | 1 | 2 | .9463 | .09861 | 1 | .000 | .7530 | 1.1396 |
|  |  | 3 | 1.7811 | .10148 | 1 | .000 | 1.5822 | 1.9800 |
|  |  | 4 | 2.0247 | .10322 | 1 | .000 | 1.8224 | 2.2270 |
|  | 2 | 1 | -.9463 | .09861 | 1 | .000 | -1.1396 | -.7530 |
|  |  | 3 | .8348 | .03403 | 1 | .000 | .7681 | .9015 |
|  |  | 4 | 1.0784 | .03712 | 1 | .000 | 1.0056 | 1.1511 |
|  | 3 | 1 | -1.7811 | .10148 | 1 | .000 | -1.9800 | -1.5822 |
|  |  | 2 | -.8348 | .03403 | 1 | .000 | -.9015 | -.7681 |
|  |  | 4 | .2436 | .04196 | 1 | .000 | .1613 | .3258 |
|  | 4 | 1 | -2.0247 | .10322 | 1 | .000 | -2.2270 | -1.8224 |
|  |  | 2 | -1.0784 | .03712 | 1 | .000 | -1.1511 | -1.0056 |
|  |  | 3 | -.2436 | .04196 | 1 | .000 | -.3258 | -.1613 |

| **Overall Test** | | | |
| --- | --- | --- | --- |
| Diagnostic | Wald χ² | Degrees of Freedom | P |
| 1 | 2044.329 | 3 | .000 |
| 2 | 1272.203 | 3 | .000 |

**Estimated Marginal Means 16：Age* Diagnostic**

| **Estimate** | | | | | |
| --- | --- | --- | --- | --- | --- |
| Age | Diagnostic | Mean | Standard Error | 95% Wald Confidence Interval | |
|  |  |  |  | Lower Bound | Upper Bound |
| 1 | 1 | 4.4966 | .04110 | 4.4167 | 4.5778 |
|  | 2 | 4.2792 | .10034 | 4.0870 | 4.4805 |
| 2 | 1 | 4.2221 | .02274 | 4.1777 | 4.2669 |
|  | 2 | 3.3329 | .02508 | 3.2841 | 3.3824 |
| 3 | 1 | 3.0788 | .03408 | 3.0128 | 3.1464 |
|  | 2 | 2.4981 | .03100 | 2.4381 | 2.5596 |
| 4 | 1 | 2.6986 | .03838 | 2.6244 | 2.7749 |
|  | 2 | 2.2545 | .03150 | 2.1936 | 2.3171 |

| **Pairwise Comparisons** | | | | | | | | |
| --- | --- | --- | --- | --- | --- | --- | --- | --- |
| Age | (I) Diagnostic | (J) Diagnostic | Mean Difference (I-J) | Standard Error | Degrees of Freedom | P | 95% Wald Confidence Interval | |
|  |  |  |  |  |  |  | Lower Bound | Upper Bound |
| 1 | 1 | 2 | .2173 | .10638 | 1 | .041 | .0088 | .4258 |
|  | 2 | 1 | -.2173 | .10638 | 1 | .041 | -.4258 | -.0088 |
| 2 | 1 | 2 | .8891 | .03195 | 1 | .000 | .8265 | .9518 |
|  | 2 | 1 | -.8891 | .03195 | 1 | .000 | -.9518 | -.8265 |
| 3 | 1 | 2 | .5807 | .02866 | 1 | .000 | .5246 | .6369 |
|  | 2 | 1 | -.5807 | .02866 | 1 | .000 | -.6369 | -.5246 |
| 4 | 1 | 2 | .4441 | .02927 | 1 | .000 | .3867 | .5014 |
|  | 2 | 1 | -.4441 | .02927 | 1 | .000 | -.5014 | -.3867 |

| **Overall Test** | | | |
| --- | --- | --- | --- |
| Age | Wald χ² | Degrees of Freedom | P |
| 1 | 4.173 | 1 | .041 |
| 2 | 774.564 | 1 | .000 |
| 3 | 410.582 | 1 | .000 |
| 4 | 230.206 | 1 | .000 |
