# Supplementary material for: The impact of COVID-19 social isolation and reduced microbial exposure on the immune system in children: a retrospective study
Source: PeerJ. 2026 Jul 7;14:e21469. doi: 10.7717/peerj.21469 (PMC13353229; doi:10.7717/peerj.21469)
Supplement: Supplemental Information 6 [file peerj-14-21469-s006.docx]

**Monocyte cell Generalized Linear Model**

For CBC analysis, patients were categorized into the following age groups:
Group 1: 0–3 months,
Group 2: 3 months–4 years,
Group 3: 4–6 years,
Group 4: 6–13 years.

Diagnostic 1 is LRTIs . Diagnostic 2 URTIs .

| **Parameter Estimate** | | | | | | | |
| --- | --- | --- | --- | --- | --- | --- | --- |
| Parameter | B | Standard Error | 95% Wald Confidence Interval | | Hypothesis Testing | | |
|  |  |  | Lower Bound | Upper Bound | Wald χ² | Degrees of Freedom | P |
| （Intercept） | -.617 | .0309 | -.677 | -.556 | 398.671 | 1 | .000 |
| [Year=2020] | .074 | .0728 | -.068 | .217 | 1.045 | 1 | .307 |
| [Year=2021] | .223 | .0408 | .143 | .303 | 29.925 | 1 | .000 |
| [Year=2022] | .078 | .0359 | .008 | .149 | 4.765 | 1 | .029 |
| [Year=2023] | .155 | .0310 | .094 | .216 | 25.086 | 1 | .000 |
| [Year=2024] | .224 | .0310 | .163 | .285 | 52.119 | 1 | .000 |
| [Year=2025] | 0 | . | . | . | . | . | . |
| [Male ] | .055 | .0169 | .022 | .088 | 10.464 | 1 | .001 |
| [Female ] | 0 | . | . | . | . | . | . |
| [Age=1] | .424 | .0399 | .346 | .502 | 112.796 | 1 | .000 |
| [Age=2] | .145 | .0302 | .086 | .204 | 23.127 | 1 | .000 |
| [Age=3] | .049 | .0355 | -.020 | .119 | 1.941 | 1 | .164 |
| [Age=4] | 0 | . | . | . | . | . | . |
| [LRTIs ] | -.046 | .0186 | -.082 | -.009 | 6.051 | 1 | .014 |
| [URTIs ] | 0 | . | . | . | . | . | . |
| [Year=2020] * [Male ] | .014 | .0323 | -.050 | .077 | .177 | 1 | .674 |
| [Year=2020] * [Female ] | 0 | . | . | . | . | . | . |
| [Year=2021] * [Male ] | .030 | .0215 | -.012 | .072 | 1.992 | 1 | .158 |
| [Year=2021] * [Female ] | 0 | . | . | . | . | . | . |
| [Year=2022] * [Male ] | .047 | .0175 | .013 | .081 | 7.203 | 1 | .007 |
| [Year=2022] * [Female ] | 0 | . | . | . | . | . | . |
| [Year=2023] * [Male ] | .036 | .0150 | .006 | .065 | 5.640 | 1 | .018 |
| [Year=2023] * [Female ] | 0 | . | . | . | . | . | . |
| [Year=2024] * [Male ] | .031 | .0149 | .002 | .061 | 4.462 | 1 | .035 |
| [Year=2024] * [Female ] | 0 | . | . | . | . | . | . |
| [Year=2025] * [Male ] | 0 | . | . | . | . | . | . |
| [Year=2025] * [Female ] | 0 | . | . | . | . | . | . |
| [Year=2020] * [Age=1] | -.224 | .0764 | -.374 | -.075 | 8.616 | 1 | .003 |
| [Year=2020] * [Age=2] | -.087 | .0719 | -.228 | .053 | 1.480 | 1 | .224 |
| [Year=2020] * [Age=3] | -.085 | .0886 | -.258 | .089 | .919 | 1 | .338 |
| [Year=2020] * [Age=4] | 0 | . | . | . | . | . | . |
| [Year=2021] * [Age=1] | -.051 | .0484 | -.146 | .043 | 1.125 | 1 | .289 |
| [Year=2021] * [Age=2] | .016 | .0395 | -.062 | .093 | .158 | 1 | .691 |
| [Year=2021] * [Age=3] | -.028 | .0457 | -.118 | .061 | .383 | 1 | .536 |
| [Year=2021] * [Age=4] | 0 | . | . | . | . | . | . |
| [Year=2022] * [Age=1] | -.094 | .0424 | -.177 | -.011 | 4.931 | 1 | .026 |
| [Year=2022] * [Age=2] | .045 | .0350 | -.024 | .113 | 1.622 | 1 | .203 |
| [Year=2022] * [Age=3] | .052 | .0402 | -.026 | .131 | 1.691 | 1 | .193 |
| [Year=2022] * [Age=4] | 0 | . | . | . | . | . | . |
| [Year=2023] * [Age=1] | -.215 | .0358 | -.285 | -.144 | 35.940 | 1 | .000 |
| [Year=2023] * [Age=2] | -.019 | .0301 | -.078 | .040 | .413 | 1 | .520 |
| [Year=2023] * [Age=3] | -.027 | .0353 | -.096 | .042 | .589 | 1 | .443 |
| [Year=2023] * [Age=4] | 0 | . | . | . | . | . | . |
| [Year=2024] * [Age=1] | -.208 | .0363 | -.279 | -.137 | 32.927 | 1 | .000 |
| [Year=2024] * [Age=2] | -.034 | .0301 | -.093 | .025 | 1.300 | 1 | .254 |
| [Year=2024] * [Age=3] | .000 | .0353 | -.070 | .069 | .000 | 1 | .995 |
| [Year=2024] * [Age=4] | 0 | . | . | . | . | . | . |
| [Year=2025] * [Age=1] | 0 | . | . | . | . | . | . |
| [Year=2025] * [Age=2] | 0 | . | . | . | . | . | . |
| [Year=2025] * [Age=3] | 0 | . | . | . | . | . | . |
| [Year=2025] * [Age=4] | 0 | . | . | . | . | . | . |
| [Year=2020] * [LRTIs ] | .046 | .0420 | -.036 | .129 | 1.220 | 1 | .269 |
| [Year=2020] * [URTIs ] | 0 | . | . | . | . | . | . |
| [Year=2021] * [LRTIs ] | -.173 | .0235 | -.219 | -.127 | 54.192 | 1 | .000 |
| [Year=2021] * [URTIs ] | 0 | . | . | . | . | . | . |
| [Year=2022] * [LRTIs ] | -.071 | .0195 | -.109 | -.033 | 13.227 | 1 | .000 |
| [Year=2022] * [URTIs ] | 0 | . | . | . | . | . | . |
| [Year=2023] * [LRTIs ] | -.037 | .0171 | -.071 | -.004 | 4.753 | 1 | .029 |
| [Year=2023] * [URTIs ] | 0 | . | . | . | . | . | . |
| [Year=2024] * [LRTIs ] | -.139 | .0170 | -.172 | -.106 | 66.672 | 1 | .000 |
| [Year=2024] * [URTIs ] | 0 | . | . | . | . | . | . |
| [Year=2025] * [LRTIs ] | 0 | . | . | . | . | . | . |
| [Year=2025] * [URTIs ] | 0 | . | . | . | . | . | . |
| [Male ] * [Age=1] | -.097 | .0176 | -.132 | -.063 | 30.526 | 1 | .000 |
| [Male ] * [Age=2] | -.046 | .0095 | -.065 | -.027 | 23.383 | 1 | .000 |
| [Male ] * [Age=3] | -.012 | .0102 | -.032 | .008 | 1.340 | 1 | .247 |
| [Male ] * [Age=4] | 0 | . | . | . | . | . | . |
| [Female ] * [Age=1] | 0 | . | . | . | . | . | . |
| [Female ] * [Age=2] | 0 | . | . | . | . | . | . |
| [Female ] * [Age=3] | 0 | . | . | . | . | . | . |
| [Female ] * [Age=4] | 0 | . | . | . | . | . | . |
| [Male ] * [LRTIs ] | .005 | .0071 | -.009 | .019 | .428 | 1 | .513 |
| [Male ] * [URTIs ] | 0 | . | . | . | . | . | . |
| [Female ] * [LRTIs ] | 0 | . | . | . | . | . | . |
| [Female ] * [URTIs ] | 0 | . | . | . | . | . | . |
| [Age=1] * [LRTIs ] | .219 | .0245 | .171 | .267 | 79.665 | 1 | .000 |
| [Age=1] * [URTIs ] | 0 | . | . | . | . | . | . |
| [Age=2] * [LRTIs ] | .047 | .0096 | .028 | .066 | 23.971 | 1 | .000 |
| [Age=2] * [URTIs ] | 0 | . | . | . | . | . | . |
| [Age=3] * [LRTIs ] | .008 | .0104 | -.013 | .028 | .562 | 1 | .454 |
| [Age=3] * [URTIs ] | 0 | . | . | . | . | . | . |
| [Age=4] * [LRTIs ] | 0 | . | . | . | . | . | . |
| [Age=4] * [URTIs ] | 0 | . | . | . | . | . | . |
| （标度） | .231 | .0011 | .228 | .233 |  |  |  |

**Estimated Marginal Means 1：Year**

| **Estimate** | | | | |
| --- | --- | --- | --- | --- |
| Year | Mean | Standard Error | 95% Wald Confidence Interval | |
|  |  |  | Lower Bound | Upper Bound |
| 2020 | .6463 | .01505 | .6175 | .6765 |
| 2021 | .7365 | .00820 | .7206 | .7527 |
| 2022 | .6875 | .00552 | .6767 | .6984 |
| 2023 | .7028 | .00341 | .6961 | .7095 |
| 2024 | .7172 | .00359 | .7102 | .7243 |
| 2025 | .6430 | .00667 | .6300 | .6562 |

| **Pairwise Comparisons** | | | | | | | |
| --- | --- | --- | --- | --- | --- | --- | --- |
| (I) Year | (J) Year | Mean Difference (I-J) | Standard Error | Degrees of Freedom | P | 95% Wald Confidence Interval | |
|  |  |  |  |  |  | Lower Bound | Upper Bound |
| 2020 | 2021 | -.0901 | .01696 | 1 | .000 | -.1234 | -.0569 |
|  | 2022 | -.0411 | .01591 | 1 | .010 | -.0723 | -.0099 |
|  | 2023 | -.0564 | .01531 | 1 | .000 | -.0864 | -.0264 |
|  | 2024 | -.0709 | .01535 | 1 | .000 | -.1010 | -.0408 |
|  | 2025 | .0034 | .01627 | 1 | .835 | -.0285 | .0353 |
| 2021 | 2020 | .0901 | .01696 | 1 | .000 | .0569 | .1234 |
|  | 2022 | .0490 | .00968 | 1 | .000 | .0300 | .0680 |
|  | 2023 | .0337 | .00865 | 1 | .000 | .0168 | .0507 |
|  | 2024 | .0192 | .00873 | 1 | .028 | .0021 | .0363 |
|  | 2025 | .0935 | .01028 | 1 | .000 | .0734 | .1137 |
| 2022 | 2020 | .0411 | .01591 | 1 | .010 | .0099 | .0723 |
|  | 2021 | -.0490 | .00968 | 1 | .000 | -.0680 | -.0300 |
|  | 2023 | -.0153 | .00628 | 1 | .015 | -.0276 | -.0030 |
|  | 2024 | -.0298 | .00638 | 1 | .000 | -.0423 | -.0173 |
|  | 2025 | .0445 | .00843 | 1 | .000 | .0280 | .0610 |
| 2023 | 2020 | .0564 | .01531 | 1 | .000 | .0264 | .0864 |
|  | 2021 | -.0337 | .00865 | 1 | .000 | -.0507 | -.0168 |
|  | 2022 | .0153 | .00628 | 1 | .015 | .0030 | .0276 |
|  | 2024 | -.0145 | .00467 | 1 | .002 | -.0236 | -.0053 |
|  | 2025 | .0598 | .00722 | 1 | .000 | .0457 | .0740 |
| 2024 | 2020 | .0709 | .01535 | 1 | .000 | .0408 | .1010 |
|  | 2021 | -.0192 | .00873 | 1 | .028 | -.0363 | -.0021 |
|  | 2022 | .0298 | .00638 | 1 | .000 | .0173 | .0423 |
|  | 2023 | .0145 | .00467 | 1 | .002 | .0053 | .0236 |
|  | 2025 | .0743 | .00732 | 1 | .000 | .0600 | .0886 |
| 2025 | 2020 | -.0034 | .01627 | 1 | .835 | -.0353 | .0285 |
|  | 2021 | -.0935 | .01028 | 1 | .000 | -.1137 | -.0734 |
|  | 2022 | -.0445 | .00843 | 1 | .000 | -.0610 | -.0280 |
|  | 2023 | -.0598 | .00722 | 1 | .000 | -.0740 | -.0457 |
|  | 2024 | -.0743 | .00732 | 1 | .000 | -.0886 | -.0600 |

| **Overall Test** | | |
| --- | --- | --- |
| Wald χ² | Degrees of Freedom | P |
| 142.963 | 5 | .000 |

**Estimated Marginal Means 2：Gender**

| **Estimate** | | | | |
| --- | --- | --- | --- | --- |
| Gender | Mean | Standard Error | 95% Wald Confidence Interval | |
|  |  |  | Lower Bound | Upper Bound |
| 1 | .7035 | .00420 | .6953 | .7118 |
| 2 | .6729 | .00453 | .6640 | .6818 |

| **Pairwise Comparisons** | | | | | | | |
| --- | --- | --- | --- | --- | --- | --- | --- |
| (I) Gender | (J) Gender | Mean Difference (I-J) | Standard Error | Degrees of Freedom | P | 95% Wald Confidence Interval | |
|  |  |  |  |  |  | Lower Bound | Upper Bound |
| 1 | 2 | .0306 | .00480 | 1 | .000 | .0212 | .0400 |
| 2 | 1 | -.0306 | .00480 | 1 | .000 | -.0400 | -.0212 |

| **Overall Test** | | |
| --- | --- | --- |
| Wald χ² | Degrees of Freedom | P |
| 40.829 | 1 | .000 |

**Estimated Marginal Means 3：Age**

| **Estimate** | | | | |
| --- | --- | --- | --- | --- |
| Age | Mean | Standard Error | 95% Wald Confidence Interval | |
|  |  |  | Lower Bound | Upper Bound |
| 1 | .8599 | .01071 | .8391 | .8811 |
| 2 | .6901 | .00324 | .6837 | .6964 |
| 3 | .6246 | .00637 | .6123 | .6372 |
| 4 | .6045 | .00751 | .5900 | .6194 |

| **Pairwise Comparisons** | | | | | | | |
| --- | --- | --- | --- | --- | --- | --- | --- |
| (I) Age | (J) Age | Mean Difference (I-J) | Standard Error | Degrees of Freedom | P | 95% Wald Confidence Interval | |
|  |  |  |  |  |  | Lower Bound | Upper Bound |
| 1 | 2 | .1698 | .01093 | 1 | .000 | .1484 | .1912 |
|  | 3 | .2352 | .01229 | 1 | .000 | .2112 | .2593 |
|  | 4 | .2553 | .01315 | 1 | .000 | .2296 | .2811 |
| 2 | 1 | -.1698 | .01093 | 1 | .000 | -.1912 | -.1484 |
|  | 3 | .0654 | .00703 | 1 | .000 | .0517 | .0792 |
|  | 4 | .0855 | .00820 | 1 | .000 | .0695 | .1016 |
| 3 | 1 | -.2352 | .01229 | 1 | .000 | -.2593 | -.2112 |
|  | 2 | -.0654 | .00703 | 1 | .000 | -.0792 | -.0517 |
|  | 4 | .0201 | .00986 | 1 | .042 | .0008 | .0394 |
| 4 | 1 | -.2553 | .01315 | 1 | .000 | -.2811 | -.2296 |
|  | 2 | -.0855 | .00820 | 1 | .000 | -.1016 | -.0695 |
|  | 3 | -.0201 | .00986 | 1 | .042 | -.0394 | -.0008 |

| **Overall Test** | | |
| --- | --- | --- |
| Wald χ² | Degrees of Freedom | P |
| 466.528 | 3 | .000 |

**Estimated Marginal Means 4：Diagnostic**

| **Estimate** | | | | |
| --- | --- | --- | --- | --- |
| Diagnostic | Mean | Standard Error | 95% Wald Confidence Interval | |
|  |  |  | Lower Bound | Upper Bound |
| 1 | .6752 | .00362 | .6682 | .6824 |
| 2 | .7010 | .00603 | .6893 | .7129 |

| **Pairwise Comparisons** | | | | | | | |
| --- | --- | --- | --- | --- | --- | --- | --- |
| (I) Diagnostic | (J) Diagnostic | Mean Difference (I-J) | Standard Error | Degrees of Freedom | P | 95% Wald Confidence Interval | |
|  |  |  |  |  |  | Lower Bound | Upper Bound |
| 1 | 2 | -.0257 | .00667 | 1 | .000 | -.0388 | -.0127 |
| 2 | 1 | .0257 | .00667 | 1 | .000 | .0127 | .0388 |

| **Overall Test** | | |
| --- | --- | --- |
| Wald χ² | Degrees of Freedom | P |
| 14.899 | 1 | .000 |

**Estimated Marginal Means 5：Year* Gender**

| **Estimate** | | | | | |
| --- | --- | --- | --- | --- | --- |
| Year | Gender | Mean | Standard Error | 95% Wald Confidence Interval | |
|  |  |  |  | Lower Bound | Upper Bound |
| 2020 | 1 | .6567 | .01677 | .6246 | .6904 |
|  | 2 | .6362 | .01879 | .6004 | .6741 |
| 2021 | 1 | .7546 | .01004 | .7352 | .7745 |
|  | 2 | .7188 | .01055 | .6984 | .7398 |
| 2022 | 1 | .7102 | .00663 | .6973 | .7233 |
|  | 2 | .6654 | .00691 | .6520 | .6791 |
| 2023 | 1 | .7220 | .00406 | .7141 | .7300 |
|  | 2 | .6841 | .00421 | .6759 | .6924 |
| 2024 | 1 | .7353 | .00421 | .7271 | .7436 |
|  | 2 | .6997 | .00438 | .6911 | .7083 |
| 2025 | 1 | .6488 | .00765 | .6340 | .6640 |
|  | 2 | .6371 | .00826 | .6211 | .6535 |

| **Pairwise Comparisons** | | | | | | | | |
| --- | --- | --- | --- | --- | --- | --- | --- | --- |
| Gender | (I) Year | (J) Year | Mean Difference (I-J) | Standard Error | Degrees of Freedom | P | 95% Wald Confidence Interval | |
|  |  |  |  |  |  |  | Lower Bound | Upper Bound |
| 1 | 2020 | 2021 | -.0979 | .01938 | 1 | .000 | -.1359 | -.0599 |
|  |  | 2022 | -.0535 | .01791 | 1 | .003 | -.0886 | -.0184 |
|  |  | 2023 | -.0653 | .01714 | 1 | .000 | -.0989 | -.0317 |
|  |  | 2024 | -.0786 | .01717 | 1 | .000 | -.1122 | -.0449 |
|  |  | 2025 | .0079 | .01824 | 1 | .666 | -.0279 | .0436 |
|  | 2021 | 2020 | .0979 | .01938 | 1 | .000 | .0599 | .1359 |
|  |  | 2022 | .0444 | .01175 | 1 | .000 | .0213 | .0674 |
|  |  | 2023 | .0326 | .01053 | 1 | .002 | .0120 | .0532 |
|  |  | 2024 | .0193 | .01058 | 1 | .068 | -.0014 | .0400 |
|  |  | 2025 | .1057 | .01234 | 1 | .000 | .0816 | .1299 |
|  | 2022 | 2020 | .0535 | .01791 | 1 | .003 | .0184 | .0886 |
|  |  | 2021 | -.0444 | .01175 | 1 | .000 | -.0674 | -.0213 |
|  |  | 2023 | -.0118 | .00745 | 1 | .114 | -.0264 | .0028 |
|  |  | 2024 | -.0251 | .00752 | 1 | .001 | -.0398 | -.0103 |
|  |  | 2025 | .0614 | .00986 | 1 | .000 | .0420 | .0807 |
|  | 2023 | 2020 | .0653 | .01714 | 1 | .000 | .0317 | .0989 |
|  |  | 2021 | -.0326 | .01053 | 1 | .002 | -.0532 | -.0120 |
|  |  | 2022 | .0118 | .00745 | 1 | .114 | -.0028 | .0264 |
|  |  | 2024 | -.0133 | .00538 | 1 | .013 | -.0239 | -.0028 |
|  |  | 2025 | .0731 | .00839 | 1 | .000 | .0567 | .0896 |
|  | 2024 | 2020 | .0786 | .01717 | 1 | .000 | .0449 | .1122 |
|  |  | 2021 | -.0193 | .01058 | 1 | .068 | -.0400 | .0014 |
|  |  | 2022 | .0251 | .00752 | 1 | .001 | .0103 | .0398 |
|  |  | 2023 | .0133 | .00538 | 1 | .013 | .0028 | .0239 |
|  |  | 2025 | .0864 | .00845 | 1 | .000 | .0699 | .1030 |
|  | 2025 | 2020 | -.0079 | .01824 | 1 | .666 | -.0436 | .0279 |
|  |  | 2021 | -.1057 | .01234 | 1 | .000 | -.1299 | -.0816 |
|  |  | 2022 | -.0614 | .00986 | 1 | .000 | -.0807 | -.0420 |
|  |  | 2023 | -.0731 | .00839 | 1 | .000 | -.0896 | -.0567 |
|  |  | 2024 | -.0864 | .00845 | 1 | .000 | -.1030 | -.0699 |
| 2 | 2020 | 2021 | -.0827 | .02139 | 1 | .000 | -.1246 | -.0407 |
|  |  | 2022 | -.0293 | .01993 | 1 | .142 | -.0684 | .0098 |
|  |  | 2023 | -.0479 | .01920 | 1 | .013 | -.0856 | -.0103 |
|  |  | 2024 | -.0635 | .01923 | 1 | .001 | -.1012 | -.0258 |
|  |  | 2025 | -.0010 | .02029 | 1 | .962 | -.0407 | .0388 |
|  | 2021 | 2020 | .0827 | .02139 | 1 | .000 | .0407 | .1246 |
|  |  | 2022 | .0534 | .01233 | 1 | .000 | .0292 | .0775 |
|  |  | 2023 | .0347 | .01106 | 1 | .002 | .0131 | .0564 |
|  |  | 2024 | .0192 | .01110 | 1 | .084 | -.0026 | .0409 |
|  |  | 2025 | .0817 | .01311 | 1 | .000 | .0560 | .1074 |
|  | 2022 | 2020 | .0293 | .01993 | 1 | .142 | -.0098 | .0684 |
|  |  | 2021 | -.0534 | .01233 | 1 | .000 | -.0775 | -.0292 |
|  |  | 2023 | -.0186 | .00770 | 1 | .016 | -.0337 | -.0035 |
|  |  | 2024 | -.0342 | .00776 | 1 | .000 | -.0494 | -.0190 |
|  |  | 2025 | .0283 | .01054 | 1 | .007 | .0077 | .0490 |
|  | 2023 | 2020 | .0479 | .01920 | 1 | .013 | .0103 | .0856 |
|  |  | 2021 | -.0347 | .01106 | 1 | .002 | -.0564 | -.0131 |
|  |  | 2022 | .0186 | .00770 | 1 | .016 | .0035 | .0337 |
|  |  | 2024 | -.0156 | .00547 | 1 | .004 | -.0263 | -.0049 |
|  |  | 2025 | .0470 | .00906 | 1 | .000 | .0292 | .0647 |
|  | 2024 | 2020 | .0635 | .01923 | 1 | .001 | .0258 | .1012 |
|  |  | 2021 | -.0192 | .01110 | 1 | .084 | -.0409 | .0026 |
|  |  | 2022 | .0342 | .00776 | 1 | .000 | .0190 | .0494 |
|  |  | 2023 | .0156 | .00547 | 1 | .004 | .0049 | .0263 |
|  |  | 2025 | .0625 | .00912 | 1 | .000 | .0447 | .0804 |
|  | 2025 | 2020 | .0010 | .02029 | 1 | .962 | -.0388 | .0407 |
|  |  | 2021 | -.0817 | .01311 | 1 | .000 | -.1074 | -.0560 |
|  |  | 2022 | -.0283 | .01054 | 1 | .007 | -.0490 | -.0077 |
|  |  | 2023 | -.0470 | .00906 | 1 | .000 | -.0647 | -.0292 |
|  |  | 2024 | -.0625 | .00912 | 1 | .000 | -.0804 | -.0447 |

| **Overall Test** | | | |
| --- | --- | --- | --- |
| Gender | Wald χ² | Degrees of Freedom | P |
| 1 | 133.716 | 5 | .000 |
| 2 | 73.238 | 5 | .000 |

**Estimated Marginal Means 6：Year* Gender**

| **Estimate** | | | | | |
| --- | --- | --- | --- | --- | --- |
| Year | Gender | Mean | Standard Error | 95% Wald Confidence Interval | |
|  |  |  |  | Lower Bound | Upper Bound |
| 2020 | 1 | .6567 | .01677 | .6246 | .6904 |
|  | 2 | .6362 | .01879 | .6004 | .6741 |
| 2021 | 1 | .7546 | .01004 | .7352 | .7745 |
|  | 2 | .7188 | .01055 | .6984 | .7398 |
| 2022 | 1 | .7102 | .00663 | .6973 | .7233 |
|  | 2 | .6654 | .00691 | .6520 | .6791 |
| 2023 | 1 | .7220 | .00406 | .7141 | .7300 |
|  | 2 | .6841 | .00421 | .6759 | .6924 |
| 2024 | 1 | .7353 | .00421 | .7271 | .7436 |
|  | 2 | .6997 | .00438 | .6911 | .7083 |
| 2025 | 1 | .6488 | .00765 | .6340 | .6640 |
|  | 2 | .6371 | .00826 | .6211 | .6535 |

| **Pairwise Comparisons** | | | | | | | | |
| --- | --- | --- | --- | --- | --- | --- | --- | --- |
| Year | (I) Gender | (J) Gender | Mean Difference (I-J) | Standard Error | Degrees of Freedom | P | 95% Wald Confidence Interval | |
|  |  |  |  |  |  |  | Lower Bound | Upper Bound |
| 2020 | 1 | 2 | .0205 | .01911 | 1 | .282 | -.0169 | .0580 |
|  | 2 | 1 | -.0205 | .01911 | 1 | .282 | -.0580 | .0169 |
| 2021 | 1 | 2 | .0358 | .01250 | 1 | .004 | .0113 | .0603 |
|  | 2 | 1 | -.0358 | .01250 | 1 | .004 | -.0603 | -.0113 |
| 2022 | 1 | 2 | .0448 | .00788 | 1 | .000 | .0293 | .0602 |
|  | 2 | 1 | -.0448 | .00788 | 1 | .000 | -.0602 | -.0293 |
| 2023 | 1 | 2 | .0379 | .00469 | 1 | .000 | .0287 | .0471 |
|  | 2 | 1 | -.0379 | .00469 | 1 | .000 | -.0471 | -.0287 |
| 2024 | 1 | 2 | .0356 | .00473 | 1 | .000 | .0264 | .0449 |
|  | 2 | 1 | -.0356 | .00473 | 1 | .000 | -.0449 | -.0264 |
| 2025 | 1 | 2 | .0117 | .00870 | 1 | .178 | -.0053 | .0288 |
|  | 2 | 1 | -.0117 | .00870 | 1 | .178 | -.0288 | .0053 |

| **Overall Test** | | | |
| --- | --- | --- | --- |
| Year | Wald χ² | Degrees of Freedom | P |
| 2020 | 1.156 | 1 | .282 |
| 2021 | 8.188 | 1 | .004 |
| 2022 | 32.254 | 1 | .000 |
| 2023 | 65.255 | 1 | .000 |
| 2024 | 56.788 | 1 | .000 |
| 2025 | 1.814 | 1 | .178 |

**Estimated Marginal Means 7：Year* Age**

| **Estimate** | | | | | |
| --- | --- | --- | --- | --- | --- |
| Year | Age | Mean | Standard Error | 95% Wald Confidence Interval | |
|  |  |  |  | Lower Bound | Upper Bound |
| 2020 | 1 | .7815 | .02530 | .7334 | .8327 |
|  | 2 | .6387 | .01422 | .6114 | .6672 |
|  | 3 | .5803 | .03085 | .5229 | .6441 |
|  | 4 | .6025 | .03708 | .5340 | .6797 |
| 2021 | 1 | .9741 | .02876 | .9193 | 1.0322 |
|  | 2 | .7425 | .00820 | .7266 | .7587 |
|  | 3 | .6439 | .01147 | .6218 | .6668 |
|  | 4 | .6317 | .01533 | .6024 | .6625 |
| 2022 | 1 | .8568 | .01968 | .8191 | .8963 |
|  | 2 | .7015 | .00508 | .6917 | .7116 |
|  | 3 | .6407 | .00673 | .6277 | .6540 |
|  | 4 | .5800 | .01032 | .5601 | .6005 |
| 2023 | 1 | .8294 | .01425 | .8019 | .8578 |
|  | 2 | .7186 | .00317 | .7125 | .7249 |
|  | 3 | .6463 | .00327 | .6399 | .6527 |
|  | 4 | .6333 | .00363 | .6262 | .6404 |
| 2024 | 1 | .8481 | .01510 | .8190 | .8782 |
|  | 2 | .7192 | .00285 | .7137 | .7248 |
|  | 3 | .6744 | .00333 | .6680 | .6810 |
|  | 4 | .6434 | .00397 | .6356 | .6512 |
| 2025 | 1 | .8810 | .01612 | .8500 | .9132 |
|  | 2 | .6280 | .00580 | .6167 | .6394 |
|  | 3 | .5691 | .01164 | .5468 | .5924 |
|  | 4 | .5428 | .01511 | .5139 | .5732 |

| **Pairwise Comparisons** | | | | | | | | |
| --- | --- | --- | --- | --- | --- | --- | --- | --- |
| Age | (I) Year | (J) Year | Mean Difference (I-J) | Standard Error | Degrees of Freedom | P | 95% Wald Confidence Interval | |
|  |  |  |  |  |  |  | Lower Bound | Upper Bound |
| 1 | 2020 | 2021 | -.1926 | .03617 | 1 | .000 | -.2635 | -.1217 |
|  |  | 2022 | -.0753 | .03047 | 1 | .013 | -.1351 | -.0156 |
|  |  | 2023 | -.0479 | .02735 | 1 | .080 | -.1015 | .0057 |
|  |  | 2024 | -.0666 | .02785 | 1 | .017 | -.1212 | -.0120 |
|  |  | 2025 | -.0995 | .02714 | 1 | .000 | -.1527 | -.0463 |
|  | 2021 | 2020 | .1926 | .03617 | 1 | .000 | .1217 | .2635 |
|  |  | 2022 | .1173 | .03324 | 1 | .000 | .0522 | .1825 |
|  |  | 2023 | .1447 | .03041 | 1 | .000 | .0851 | .2043 |
|  |  | 2024 | .1261 | .03087 | 1 | .000 | .0656 | .1866 |
|  |  | 2025 | .0931 | .03011 | 1 | .002 | .0341 | .1521 |
|  | 2022 | 2020 | .0753 | .03047 | 1 | .013 | .0156 | .1351 |
|  |  | 2021 | -.1173 | .03324 | 1 | .000 | -.1825 | -.0522 |
|  |  | 2023 | .0274 | .02286 | 1 | .231 | -.0174 | .0722 |
|  |  | 2024 | .0088 | .02343 | 1 | .709 | -.0372 | .0547 |
|  |  | 2025 | -.0242 | .02312 | 1 | .296 | -.0695 | .0211 |
|  | 2023 | 2020 | .0479 | .02735 | 1 | .080 | -.0057 | .1015 |
|  |  | 2021 | -.1447 | .03041 | 1 | .000 | -.2043 | -.0851 |
|  |  | 2022 | -.0274 | .02286 | 1 | .231 | -.0722 | .0174 |
|  |  | 2024 | -.0187 | .01916 | 1 | .330 | -.0562 | .0189 |
|  |  | 2025 | -.0516 | .01883 | 1 | .006 | -.0885 | -.0147 |
|  | 2024 | 2020 | .0666 | .02785 | 1 | .017 | .0120 | .1212 |
|  |  | 2021 | -.1261 | .03087 | 1 | .000 | -.1866 | -.0656 |
|  |  | 2022 | -.0088 | .02343 | 1 | .709 | -.0547 | .0372 |
|  |  | 2023 | .0187 | .01916 | 1 | .330 | -.0189 | .0562 |
|  |  | 2025 | -.0329 | .01956 | 1 | .092 | -.0713 | .0054 |
|  | 2025 | 2020 | .0995 | .02714 | 1 | .000 | .0463 | .1527 |
|  |  | 2021 | -.0931 | .03011 | 1 | .002 | -.1521 | -.0341 |
|  |  | 2022 | .0242 | .02312 | 1 | .296 | -.0211 | .0695 |
|  |  | 2023 | .0516 | .01883 | 1 | .006 | .0147 | .0885 |
|  |  | 2024 | .0329 | .01956 | 1 | .092 | -.0054 | .0713 |
| 2 | 2020 | 2021 | -.1038 | .01641 | 1 | .000 | -.1359 | -.0716 |
|  |  | 2022 | -.0628 | .01509 | 1 | .000 | -.0924 | -.0333 |
|  |  | 2023 | -.0799 | .01456 | 1 | .000 | -.1085 | -.0514 |
|  |  | 2024 | -.0805 | .01449 | 1 | .000 | -.1089 | -.0521 |
|  |  | 2025 | .0107 | .01534 | 1 | .484 | -.0193 | .0408 |
|  | 2021 | 2020 | .1038 | .01641 | 1 | .000 | .0716 | .1359 |
|  |  | 2022 | .0409 | .00964 | 1 | .000 | .0220 | .0598 |
|  |  | 2023 | .0238 | .00878 | 1 | .007 | .0066 | .0410 |
|  |  | 2024 | .0232 | .00867 | 1 | .007 | .0062 | .0402 |
|  |  | 2025 | .1145 | .01004 | 1 | .000 | .0948 | .1342 |
|  | 2022 | 2020 | .0628 | .01509 | 1 | .000 | .0333 | .0924 |
|  |  | 2021 | -.0409 | .00964 | 1 | .000 | -.0598 | -.0220 |
|  |  | 2023 | -.0171 | .00598 | 1 | .004 | -.0288 | -.0054 |
|  |  | 2024 | -.0177 | .00582 | 1 | .002 | -.0291 | -.0063 |
|  |  | 2025 | .0736 | .00770 | 1 | .000 | .0585 | .0887 |
|  | 2023 | 2020 | .0799 | .01456 | 1 | .000 | .0514 | .1085 |
|  |  | 2021 | -.0238 | .00878 | 1 | .007 | -.0410 | -.0066 |
|  |  | 2022 | .0171 | .00598 | 1 | .004 | .0054 | .0288 |
|  |  | 2024 | -.0006 | .00425 | 1 | .893 | -.0089 | .0077 |
|  |  | 2025 | .0907 | .00660 | 1 | .000 | .0777 | .1036 |
|  | 2024 | 2020 | .0805 | .01449 | 1 | .000 | .0521 | .1089 |
|  |  | 2021 | -.0232 | .00867 | 1 | .007 | -.0402 | -.0062 |
|  |  | 2022 | .0177 | .00582 | 1 | .002 | .0063 | .0291 |
|  |  | 2023 | .0006 | .00425 | 1 | .893 | -.0077 | .0089 |
|  |  | 2025 | .0912 | .00645 | 1 | .000 | .0786 | .1039 |
|  | 2025 | 2020 | -.0107 | .01534 | 1 | .484 | -.0408 | .0193 |
|  |  | 2021 | -.1145 | .01004 | 1 | .000 | -.1342 | -.0948 |
|  |  | 2022 | -.0736 | .00770 | 1 | .000 | -.0887 | -.0585 |
|  |  | 2023 | -.0907 | .00660 | 1 | .000 | -.1036 | -.0777 |
|  |  | 2024 | -.0912 | .00645 | 1 | .000 | -.1039 | -.0786 |
| 3 | 2020 | 2021 | -.0636 | .03290 | 1 | .053 | -.1281 | .0009 |
|  |  | 2022 | -.0604 | .03156 | 1 | .056 | -.1222 | .0015 |
|  |  | 2023 | -.0659 | .03102 | 1 | .034 | -.1267 | -.0051 |
|  |  | 2024 | -.0941 | .03102 | 1 | .002 | -.1549 | -.0333 |
|  |  | 2025 | .0112 | .03296 | 1 | .734 | -.0534 | .0758 |
|  | 2021 | 2020 | .0636 | .03290 | 1 | .053 | -.0009 | .1281 |
|  |  | 2022 | .0032 | .01327 | 1 | .809 | -.0228 | .0292 |
|  |  | 2023 | -.0023 | .01191 | 1 | .845 | -.0257 | .0210 |
|  |  | 2024 | -.0305 | .01191 | 1 | .010 | -.0539 | -.0072 |
|  |  | 2025 | .0748 | .01631 | 1 | .000 | .0428 | .1068 |
|  | 2022 | 2020 | .0604 | .03156 | 1 | .056 | -.0015 | .1222 |
|  |  | 2021 | -.0032 | .01327 | 1 | .809 | -.0292 | .0228 |
|  |  | 2023 | -.0055 | .00746 | 1 | .458 | -.0202 | .0091 |
|  |  | 2024 | -.0337 | .00746 | 1 | .000 | -.0483 | -.0191 |
|  |  | 2025 | .0716 | .01342 | 1 | .000 | .0453 | .0979 |
|  | 2023 | 2020 | .0659 | .03102 | 1 | .034 | .0051 | .1267 |
|  |  | 2021 | .0023 | .01191 | 1 | .845 | -.0210 | .0257 |
|  |  | 2022 | .0055 | .00746 | 1 | .458 | -.0091 | .0202 |
|  |  | 2024 | -.0282 | .00463 | 1 | .000 | -.0373 | -.0191 |
|  |  | 2025 | .0771 | .01208 | 1 | .000 | .0535 | .1008 |
|  | 2024 | 2020 | .0941 | .03102 | 1 | .002 | .0333 | .1549 |
|  |  | 2021 | .0305 | .01191 | 1 | .010 | .0072 | .0539 |
|  |  | 2022 | .0337 | .00746 | 1 | .000 | .0191 | .0483 |
|  |  | 2023 | .0282 | .00463 | 1 | .000 | .0191 | .0373 |
|  |  | 2025 | .1053 | .01208 | 1 | .000 | .0817 | .1290 |
|  | 2025 | 2020 | -.0112 | .03296 | 1 | .734 | -.0758 | .0534 |
|  |  | 2021 | -.0748 | .01631 | 1 | .000 | -.1068 | -.0428 |
|  |  | 2022 | -.0716 | .01342 | 1 | .000 | -.0979 | -.0453 |
|  |  | 2023 | -.0771 | .01208 | 1 | .000 | -.1008 | -.0535 |
|  |  | 2024 | -.1053 | .01208 | 1 | .000 | -.1290 | -.0817 |
| 4 | 2020 | 2021 | -.0292 | .04012 | 1 | .466 | -.1078 | .0494 |
|  |  | 2022 | .0226 | .03847 | 1 | .558 | -.0529 | .0980 |
|  |  | 2023 | -.0308 | .03725 | 1 | .409 | -.1038 | .0423 |
|  |  | 2024 | -.0408 | .03729 | 1 | .273 | -.1139 | .0322 |
|  |  | 2025 | .0597 | .04003 | 1 | .136 | -.0187 | .1382 |
|  | 2021 | 2020 | .0292 | .04012 | 1 | .466 | -.0494 | .1078 |
|  |  | 2022 | .0518 | .01848 | 1 | .005 | .0156 | .0880 |
|  |  | 2023 | -.0015 | .01574 | 1 | .922 | -.0324 | .0293 |
|  |  | 2024 | -.0116 | .01582 | 1 | .462 | -.0426 | .0194 |
|  |  | 2025 | .0890 | .02152 | 1 | .000 | .0468 | .1311 |
|  | 2022 | 2020 | -.0226 | .03847 | 1 | .558 | -.0980 | .0529 |
|  |  | 2021 | -.0518 | .01848 | 1 | .005 | -.0880 | -.0156 |
|  |  | 2023 | -.0533 | .01093 | 1 | .000 | -.0747 | -.0319 |
|  |  | 2024 | -.0634 | .01105 | 1 | .000 | -.0851 | -.0418 |
|  |  | 2025 | .0372 | .01829 | 1 | .042 | .0013 | .0730 |
|  | 2023 | 2020 | .0308 | .03725 | 1 | .409 | -.0423 | .1038 |
|  |  | 2021 | .0015 | .01574 | 1 | .922 | -.0293 | .0324 |
|  |  | 2022 | .0533 | .01093 | 1 | .000 | .0319 | .0747 |
|  |  | 2024 | -.0101 | .00533 | 1 | .058 | -.0205 | .0004 |
|  |  | 2025 | .0905 | .01553 | 1 | .000 | .0601 | .1210 |
|  | 2024 | 2020 | .0408 | .03729 | 1 | .273 | -.0322 | .1139 |
|  |  | 2021 | .0116 | .01582 | 1 | .462 | -.0194 | .0426 |
|  |  | 2022 | .0634 | .01105 | 1 | .000 | .0418 | .0851 |
|  |  | 2023 | .0101 | .00533 | 1 | .058 | -.0004 | .0205 |
|  |  | 2025 | .1006 | .01562 | 1 | .000 | .0700 | .1312 |
|  | 2025 | 2020 | -.0597 | .04003 | 1 | .136 | -.1382 | .0187 |
|  |  | 2021 | -.0890 | .02152 | 1 | .000 | -.1311 | -.0468 |
|  |  | 2022 | -.0372 | .01829 | 1 | .042 | -.0730 | -.0013 |
|  |  | 2023 | -.0905 | .01553 | 1 | .000 | -.1210 | -.0601 |
|  |  | 2024 | -.1006 | .01562 | 1 | .000 | -.1312 | -.0700 |

| **Overall Test** | | | |
| --- | --- | --- | --- |
| Age | Wald χ² | Degrees of Freedom | P |
| 1 | 36.130 | 5 | .000 |
| 2 | 260.318 | 5 | .000 |
| 3 | 108.241 | 5 | .000 |
| 4 | 69.251 | 5 | .000 |

**Estimated Marginal Means 8：Year* Age**

| **Estimate** | | | | | |
| --- | --- | --- | --- | --- | --- |
| Year | Age | Mean | Standard Error | 95% Wald Confidence Interval | |
|  |  |  |  | Lower Bound | Upper Bound |
| 2020 | 1 | .7815 | .02530 | .7334 | .8327 |
|  | 2 | .6387 | .01422 | .6114 | .6672 |
|  | 3 | .5803 | .03085 | .5229 | .6441 |
|  | 4 | .6025 | .03708 | .5340 | .6797 |
| 2021 | 1 | .9741 | .02876 | .9193 | 1.0322 |
|  | 2 | .7425 | .00820 | .7266 | .7587 |
|  | 3 | .6439 | .01147 | .6218 | .6668 |
|  | 4 | .6317 | .01533 | .6024 | .6625 |
| 2022 | 1 | .8568 | .01968 | .8191 | .8963 |
|  | 2 | .7015 | .00508 | .6917 | .7116 |
|  | 3 | .6407 | .00673 | .6277 | .6540 |
|  | 4 | .5800 | .01032 | .5601 | .6005 |
| 2023 | 1 | .8294 | .01425 | .8019 | .8578 |
|  | 2 | .7186 | .00317 | .7125 | .7249 |
|  | 3 | .6463 | .00327 | .6399 | .6527 |
|  | 4 | .6333 | .00363 | .6262 | .6404 |
| 2024 | 1 | .8481 | .01510 | .8190 | .8782 |
|  | 2 | .7192 | .00285 | .7137 | .7248 |
|  | 3 | .6744 | .00333 | .6680 | .6810 |
|  | 4 | .6434 | .00397 | .6356 | .6512 |
| 2025 | 1 | .8810 | .01612 | .8500 | .9132 |
|  | 2 | .6280 | .00580 | .6167 | .6394 |
|  | 3 | .5691 | .01164 | .5468 | .5924 |
|  | 4 | .5428 | .01511 | .5139 | .5732 |

| **Pairwise Comparisons** | | | | | | | | |
| --- | --- | --- | --- | --- | --- | --- | --- | --- |
| Year | (I) Age | (J) Age | Mean Difference (I-J) | Standard Error | Degrees of Freedom | P | 95% Wald Confidence Interval | |
|  |  |  |  |  |  |  | Lower Bound | Upper Bound |
| 2020 | 1 | 2 | .1428 | .02587 | 1 | .000 | .0921 | .1935 |
|  |  | 3 | .2011 | .03841 | 1 | .000 | .1259 | .2764 |
|  |  | 4 | .1790 | .04559 | 1 | .000 | .0896 | .2683 |
|  | 2 | 1 | -.1428 | .02587 | 1 | .000 | -.1935 | -.0921 |
|  |  | 3 | .0584 | .03326 | 1 | .079 | -.0068 | .1236 |
|  |  | 4 | .0362 | .03987 | 1 | .364 | -.0420 | .1143 |
|  | 3 | 1 | -.2011 | .03841 | 1 | .000 | -.2764 | -.1259 |
|  |  | 2 | -.0584 | .03326 | 1 | .079 | -.1236 | .0068 |
|  |  | 4 | -.0222 | .04839 | 1 | .647 | -.1170 | .0727 |
|  | 4 | 1 | -.1790 | .04559 | 1 | .000 | -.2683 | -.0896 |
|  |  | 2 | -.0362 | .03987 | 1 | .364 | -.1143 | .0420 |
|  |  | 3 | .0222 | .04839 | 1 | .647 | -.0727 | .1170 |
| 2021 | 1 | 2 | .2317 | .02974 | 1 | .000 | .1734 | .2900 |
|  |  | 3 | .3302 | .03063 | 1 | .000 | .2702 | .3902 |
|  |  | 4 | .3424 | .03244 | 1 | .000 | .2788 | .4060 |
|  | 2 | 1 | -.2317 | .02974 | 1 | .000 | -.2900 | -.1734 |
|  |  | 3 | .0985 | .01396 | 1 | .000 | .0712 | .1259 |
|  |  | 4 | .1107 | .01732 | 1 | .000 | .0768 | .1447 |
|  | 3 | 1 | -.3302 | .03063 | 1 | .000 | -.3902 | -.2702 |
|  |  | 2 | -.0985 | .01396 | 1 | .000 | -.1259 | -.0712 |
|  |  | 4 | .0122 | .01907 | 1 | .522 | -.0252 | .0496 |
|  | 4 | 1 | -.3424 | .03244 | 1 | .000 | -.4060 | -.2788 |
|  |  | 2 | -.1107 | .01732 | 1 | .000 | -.1447 | -.0768 |
|  |  | 3 | -.0122 | .01907 | 1 | .522 | -.0496 | .0252 |
| 2022 | 1 | 2 | .1553 | .02022 | 1 | .000 | .1156 | .1949 |
|  |  | 3 | .2161 | .02067 | 1 | .000 | .1756 | .2566 |
|  |  | 4 | .2769 | .02220 | 1 | .000 | .2333 | .3204 |
|  | 2 | 1 | -.1553 | .02022 | 1 | .000 | -.1949 | -.1156 |
|  |  | 3 | .0608 | .00833 | 1 | .000 | .0445 | .0772 |
|  |  | 4 | .1216 | .01148 | 1 | .000 | .0991 | .1441 |
|  | 3 | 1 | -.2161 | .02067 | 1 | .000 | -.2566 | -.1756 |
|  |  | 2 | -.0608 | .00833 | 1 | .000 | -.0772 | -.0445 |
|  |  | 4 | .0608 | .01230 | 1 | .000 | .0367 | .0849 |
|  | 4 | 1 | -.2769 | .02220 | 1 | .000 | -.3204 | -.2333 |
|  |  | 2 | -.1216 | .01148 | 1 | .000 | -.1441 | -.0991 |
|  |  | 3 | -.0608 | .01230 | 1 | .000 | -.0849 | -.0367 |
| 2023 | 1 | 2 | .1108 | .01457 | 1 | .000 | .0822 | .1393 |
|  |  | 3 | .1831 | .01460 | 1 | .000 | .1545 | .2118 |
|  |  | 4 | .1961 | .01468 | 1 | .000 | .1674 | .2249 |
|  | 2 | 1 | -.1108 | .01457 | 1 | .000 | -.1393 | -.0822 |
|  |  | 3 | .0724 | .00454 | 1 | .000 | .0635 | .0813 |
|  |  | 4 | .0854 | .00481 | 1 | .000 | .0760 | .0948 |
|  | 3 | 1 | -.1831 | .01460 | 1 | .000 | -.2118 | -.1545 |
|  |  | 2 | -.0724 | .00454 | 1 | .000 | -.0813 | -.0635 |
|  |  | 4 | .0130 | .00488 | 1 | .008 | .0034 | .0225 |
|  | 4 | 1 | -.1961 | .01468 | 1 | .000 | -.2249 | -.1674 |
|  |  | 2 | -.0854 | .00481 | 1 | .000 | -.0948 | -.0760 |
|  |  | 3 | -.0130 | .00488 | 1 | .008 | -.0225 | -.0034 |
| 2024 | 1 | 2 | .1288 | .01534 | 1 | .000 | .0988 | .1589 |
|  |  | 3 | .1736 | .01543 | 1 | .000 | .1434 | .2039 |
|  |  | 4 | .2047 | .01559 | 1 | .000 | .1742 | .2353 |
|  | 2 | 1 | -.1288 | .01534 | 1 | .000 | -.1589 | -.0988 |
|  |  | 3 | .0448 | .00435 | 1 | .000 | .0362 | .0533 |
|  |  | 4 | .0759 | .00487 | 1 | .000 | .0663 | .0854 |
|  | 3 | 1 | -.1736 | .01543 | 1 | .000 | -.2039 | -.1434 |
|  |  | 2 | -.0448 | .00435 | 1 | .000 | -.0533 | -.0362 |
|  |  | 4 | .0311 | .00515 | 1 | .000 | .0210 | .0412 |
|  | 4 | 1 | -.2047 | .01559 | 1 | .000 | -.2353 | -.1742 |
|  |  | 2 | -.0759 | .00487 | 1 | .000 | -.0854 | -.0663 |
|  |  | 3 | -.0311 | .00515 | 1 | .000 | -.0412 | -.0210 |
| 2025 | 1 | 2 | .2530 | .01633 | 1 | .000 | .2210 | .2850 |
|  |  | 3 | .3119 | .01949 | 1 | .000 | .2737 | .3501 |
|  |  | 4 | .3382 | .02204 | 1 | .000 | .2950 | .3814 |
|  | 2 | 1 | -.2530 | .01633 | 1 | .000 | -.2850 | -.2210 |
|  |  | 3 | .0589 | .01279 | 1 | .000 | .0338 | .0839 |
|  |  | 4 | .0852 | .01614 | 1 | .000 | .0536 | .1168 |
|  | 3 | 1 | -.3119 | .01949 | 1 | .000 | -.3501 | -.2737 |
|  |  | 2 | -.0589 | .01279 | 1 | .000 | -.0839 | -.0338 |
|  |  | 4 | .0264 | .01905 | 1 | .167 | -.0110 | .0637 |
|  | 4 | 1 | -.3382 | .02204 | 1 | .000 | -.3814 | -.2950 |
|  |  | 2 | -.0852 | .01614 | 1 | .000 | -.1168 | -.0536 |
|  |  | 3 | -.0264 | .01905 | 1 | .167 | -.0637 | .0110 |

| **Overall Test** | | | |
| --- | --- | --- | --- |
| Year | Wald χ² | Degrees of Freedom | P |
| 2020 | 39.222 | 3 | .000 |
| 2021 | 162.776 | 3 | .000 |
| 2022 | 224.511 | 3 | .000 |
| 2023 | 516.895 | 3 | .000 |
| 2024 | 377.376 | 3 | .000 |
| 2025 | 314.721 | 3 | .000 |

**Estimated Marginal Means 9：Year* Diagnostic**

| **Estimate** | | | | | |
| --- | --- | --- | --- | --- | --- |
| Year | Diagnostic | Mean | Standard Error | 95% Wald Confidence Interval | |
|  |  |  |  | Lower Bound | Upper Bound |
| 2020 | 1 | .6698 | .01672 | .6378 | .7034 |
|  | 2 | .6237 | .02188 | .5823 | .6681 |
| 2021 | 1 | .6838 | .00821 | .6679 | .7001 |
|  | 2 | .7932 | .01299 | .7681 | .8191 |
| 2022 | 1 | .6718 | .00593 | .6603 | .6835 |
|  | 2 | .7035 | .00793 | .6881 | .7192 |
| 2023 | 1 | .6984 | .00355 | .6915 | .7054 |
|  | 2 | .7072 | .00510 | .6972 | .7172 |
| 2024 | 1 | .6775 | .00352 | .6706 | .6844 |
|  | 2 | .7593 | .00555 | .7485 | .7703 |
| 2025 | 1 | .6510 | .00692 | .6376 | .6647 |
|  | 2 | .6350 | .00999 | .6157 | .6549 |

| **Pairwise Comparisons** | | | | | | | | |
| --- | --- | --- | --- | --- | --- | --- | --- | --- |
| Diagnostic | (I) Year | (J) Year | Mean Difference (I-J) | Standard Error | Degrees of Freedom | P | 95% Wald Confidence Interval | |
|  |  |  |  |  |  |  | Lower Bound | Upper Bound |
| 1 | 2020 | 2021 | -.0140 | .01860 | 1 | .451 | -.0505 | .0224 |
|  |  | 2022 | -.0020 | .01771 | 1 | .910 | -.0367 | .0327 |
|  |  | 2023 | -.0286 | .01710 | 1 | .094 | -.0621 | .0049 |
|  |  | 2024 | -.0077 | .01708 | 1 | .653 | -.0412 | .0258 |
|  |  | 2025 | .0188 | .01805 | 1 | .298 | -.0166 | .0542 |
|  | 2021 | 2020 | .0140 | .01860 | 1 | .451 | -.0224 | .0505 |
|  |  | 2022 | .0120 | .01008 | 1 | .233 | -.0077 | .0318 |
|  |  | 2023 | -.0146 | .00892 | 1 | .102 | -.0321 | .0029 |
|  |  | 2024 | .0063 | .00890 | 1 | .477 | -.0111 | .0238 |
|  |  | 2025 | .0328 | .01070 | 1 | .002 | .0118 | .0538 |
|  | 2022 | 2020 | .0020 | .01771 | 1 | .910 | -.0327 | .0367 |
|  |  | 2021 | -.0120 | .01008 | 1 | .233 | -.0318 | .0077 |
|  |  | 2023 | -.0266 | .00683 | 1 | .000 | -.0400 | -.0132 |
|  |  | 2024 | -.0057 | .00680 | 1 | .403 | -.0190 | .0076 |
|  |  | 2025 | .0208 | .00907 | 1 | .022 | .0030 | .0386 |
|  | 2023 | 2020 | .0286 | .01710 | 1 | .094 | -.0049 | .0621 |
|  |  | 2021 | .0146 | .00892 | 1 | .102 | -.0029 | .0321 |
|  |  | 2022 | .0266 | .00683 | 1 | .000 | .0132 | .0400 |
|  |  | 2024 | .0209 | .00486 | 1 | .000 | .0114 | .0305 |
|  |  | 2025 | .0474 | .00777 | 1 | .000 | .0322 | .0626 |
|  | 2024 | 2020 | .0077 | .01708 | 1 | .653 | -.0258 | .0412 |
|  |  | 2021 | -.0063 | .00890 | 1 | .477 | -.0238 | .0111 |
|  |  | 2022 | .0057 | .00680 | 1 | .403 | -.0076 | .0190 |
|  |  | 2023 | -.0209 | .00486 | 1 | .000 | -.0305 | -.0114 |
|  |  | 2025 | .0265 | .00775 | 1 | .001 | .0113 | .0417 |
|  | 2025 | 2020 | -.0188 | .01805 | 1 | .298 | -.0542 | .0166 |
|  |  | 2021 | -.0328 | .01070 | 1 | .002 | -.0538 | -.0118 |
|  |  | 2022 | -.0208 | .00907 | 1 | .022 | -.0386 | -.0030 |
|  |  | 2023 | -.0474 | .00777 | 1 | .000 | -.0626 | -.0322 |
|  |  | 2024 | -.0265 | .00775 | 1 | .001 | -.0417 | -.0113 |
| 2 | 2020 | 2021 | -.1695 | .02491 | 1 | .000 | -.2183 | -.1207 |
|  |  | 2022 | -.0798 | .02285 | 1 | .000 | -.1246 | -.0350 |
|  |  | 2023 | -.0835 | .02202 | 1 | .000 | -.1266 | -.0403 |
|  |  | 2024 | -.1356 | .02209 | 1 | .000 | -.1789 | -.0923 |
|  |  | 2025 | -.0113 | .02356 | 1 | .632 | -.0575 | .0349 |
|  | 2021 | 2020 | .1695 | .02491 | 1 | .000 | .1207 | .2183 |
|  |  | 2022 | .0897 | .01441 | 1 | .000 | .0615 | .1179 |
|  |  | 2023 | .0860 | .01305 | 1 | .000 | .0605 | .1116 |
|  |  | 2024 | .0339 | .01316 | 1 | .010 | .0081 | .0596 |
|  |  | 2025 | .1582 | .01549 | 1 | .000 | .1278 | .1886 |
|  | 2022 | 2020 | .0798 | .02285 | 1 | .000 | .0350 | .1246 |
|  |  | 2021 | -.0897 | .01441 | 1 | .000 | -.1179 | -.0615 |
|  |  | 2023 | -.0037 | .00843 | 1 | .663 | -.0202 | .0128 |
|  |  | 2024 | -.0559 | .00862 | 1 | .000 | -.0728 | -.0390 |
|  |  | 2025 | .0685 | .01192 | 1 | .000 | .0451 | .0919 |
|  | 2023 | 2020 | .0835 | .02202 | 1 | .000 | .0403 | .1266 |
|  |  | 2021 | -.0860 | .01305 | 1 | .000 | -.1116 | -.0605 |
|  |  | 2022 | .0037 | .00843 | 1 | .663 | -.0128 | .0202 |
|  |  | 2024 | -.0522 | .00606 | 1 | .000 | -.0641 | -.0403 |
|  |  | 2025 | .0722 | .01025 | 1 | .000 | .0521 | .0923 |
|  | 2024 | 2020 | .1356 | .02209 | 1 | .000 | .0923 | .1789 |
|  |  | 2021 | -.0339 | .01316 | 1 | .010 | -.0596 | -.0081 |
|  |  | 2022 | .0559 | .00862 | 1 | .000 | .0390 | .0728 |
|  |  | 2023 | .0522 | .00606 | 1 | .000 | .0403 | .0641 |
|  |  | 2025 | .1244 | .01039 | 1 | .000 | .1040 | .1447 |
|  | 2025 | 2020 | .0113 | .02356 | 1 | .632 | -.0349 | .0575 |
|  |  | 2021 | -.1582 | .01549 | 1 | .000 | -.1886 | -.1278 |
|  |  | 2022 | -.0685 | .01192 | 1 | .000 | -.0919 | -.0451 |
|  |  | 2023 | -.0722 | .01025 | 1 | .000 | -.0923 | -.0521 |
|  |  | 2024 | -.1244 | .01039 | 1 | .000 | -.1447 | -.1040 |

| **Overall Test** | | | |
| --- | --- | --- | --- |
| Diagnostic | Wald χ² | Degrees of Freedom | P |
| 1 | 47.411 | 5 | .000 |
| 2 | 229.211 | 5 | .000 |

**Estimated Marginal Means 10：Year* Diagnostic**

| **Estimate** | | | | | |
| --- | --- | --- | --- | --- | --- |
| Year | Diagnostic | Mean | Standard Error | 95% Wald Confidence Interval | |
|  |  |  |  | Lower Bound | Upper Bound |
| 2020 | 1 | .6698 | .01672 | .6378 | .7034 |
|  | 2 | .6237 | .02188 | .5823 | .6681 |
| 2021 | 1 | .6838 | .00821 | .6679 | .7001 |
|  | 2 | .7932 | .01299 | .7681 | .8191 |
| 2022 | 1 | .6718 | .00593 | .6603 | .6835 |
|  | 2 | .7035 | .00793 | .6881 | .7192 |
| 2023 | 1 | .6984 | .00355 | .6915 | .7054 |
|  | 2 | .7072 | .00510 | .6972 | .7172 |
| 2024 | 1 | .6775 | .00352 | .6706 | .6844 |
|  | 2 | .7593 | .00555 | .7485 | .7703 |
| 2025 | 1 | .6510 | .00692 | .6376 | .6647 |
|  | 2 | .6350 | .00999 | .6157 | .6549 |

| **Pairwise Comparisons** | | | | | | | | |
| --- | --- | --- | --- | --- | --- | --- | --- | --- |
| Year | (I) Diagnostic | (J) Diagnostic | Mean Difference (I-J) | Standard Error | Degrees of Freedom | P | 95% Wald Confidence Interval | |
|  |  |  |  |  |  |  | Lower Bound | Upper Bound |
| 2020 | 1 | 2 | .0461 | .02503 | 1 | .065 | -.0029 | .0952 |
|  | 2 | 1 | -.0461 | .02503 | 1 | .065 | -.0952 | .0029 |
| 2021 | 1 | 2 | -.1094 | .01382 | 1 | .000 | -.1364 | -.0823 |
|  | 2 | 1 | .1094 | .01382 | 1 | .000 | .0823 | .1364 |
| 2022 | 1 | 2 | -.0317 | .00855 | 1 | .000 | -.0484 | -.0149 |
|  | 2 | 1 | .0317 | .00855 | 1 | .000 | .0149 | .0484 |
| 2023 | 1 | 2 | -.0087 | .00552 | 1 | .114 | -.0195 | .0021 |
|  | 2 | 1 | .0087 | .00552 | 1 | .114 | -.0021 | .0195 |
| 2024 | 1 | 2 | -.0818 | .00574 | 1 | .000 | -.0931 | -.0706 |
|  | 2 | 1 | .0818 | .00574 | 1 | .000 | .0706 | .0931 |
| 2025 | 1 | 2 | .0161 | .01089 | 1 | .141 | -.0053 | .0374 |
|  | 2 | 1 | -.0161 | .01089 | 1 | .141 | -.0374 | .0053 |

| **Overall Test** | | | |
| --- | --- | --- | --- |
| Year | Wald χ² | Degrees of Freedom | P |
| 2020 | 3.395 | 1 | .065 |
| 2021 | 62.634 | 1 | .000 |
| 2022 | 13.726 | 1 | .000 |
| 2023 | 2.499 | 1 | .114 |
| 2024 | 203.173 | 1 | .000 |
| 2025 | 2.172 | 1 | .141 |

**Estimated Marginal Means 11：Gender* Age**

| **Estimate** | | | | | |
| --- | --- | --- | --- | --- | --- |
| Gender | Age | Mean | Standard Error | 95% Wald Confidence Interval | |
|  |  |  |  | Lower Bound | Upper Bound |
| 1 | 1 | .8538 | .01199 | .8307 | .8776 |
|  | 2 | .7031 | .00386 | .6956 | .7107 |
|  | 3 | .6474 | .00708 | .6336 | .6614 |
|  | 4 | .6303 | .00818 | .6144 | .6465 |
| 2 | 1 | .8659 | .01312 | .8406 | .8920 |
|  | 2 | .6773 | .00438 | .6687 | .6859 |
|  | 3 | .6027 | .00685 | .5894 | .6163 |
|  | 4 | .5799 | .00806 | .5643 | .5959 |

| **Pairwise Comparisons** | | | | | | | | |
| --- | --- | --- | --- | --- | --- | --- | --- | --- |
| Age | (I) Gender | (J) Gender | Mean Difference (I-J) | Standard Error | Degrees of Freedom | P | 95% Wald Confidence Interval | |
|  |  |  |  |  |  |  | Lower Bound | Upper Bound |
| 1 | 1 | 2 | -.0121 | .01313 | 1 | .356 | -.0378 | .0136 |
|  | 2 | 1 | .0121 | .01313 | 1 | .356 | -.0136 | .0378 |
| 2 | 1 | 2 | .0258 | .00512 | 1 | .000 | .0158 | .0358 |
|  | 2 | 1 | -.0258 | .00512 | 1 | .000 | -.0358 | -.0158 |
| 3 | 1 | 2 | .0447 | .00564 | 1 | .000 | .0336 | .0558 |
|  | 2 | 1 | -.0447 | .00564 | 1 | .000 | -.0558 | -.0336 |
| 4 | 1 | 2 | .0504 | .00620 | 1 | .000 | .0382 | .0625 |
|  | 2 | 1 | -.0504 | .00620 | 1 | .000 | -.0625 | -.0382 |

| **Overall Test** | | | |
| --- | --- | --- | --- |
| Age | Wald χ² | Degrees of Freedom | P |
| 1 | .851 | 1 | .356 |
| 2 | 25.357 | 1 | .000 |
| 3 | 62.709 | 1 | .000 |
| 4 | 65.998 | 1 | .000 |

**Estimated Marginal Means 12：Gender* Age**

| **Estimate** | | | | | |
| --- | --- | --- | --- | --- | --- |
| Gender | Age | Mean | Standard Error | 95% Wald Confidence Interval | |
|  |  |  |  | Lower Bound | Upper Bound |
| 1 | 1 | .8538 | .01199 | .8307 | .8776 |
|  | 2 | .7031 | .00386 | .6956 | .7107 |
|  | 3 | .6474 | .00708 | .6336 | .6614 |
|  | 4 | .6303 | .00818 | .6144 | .6465 |
| 2 | 1 | .8659 | .01312 | .8406 | .8920 |
|  | 2 | .6773 | .00438 | .6687 | .6859 |
|  | 3 | .6027 | .00685 | .5894 | .6163 |
|  | 4 | .5799 | .00806 | .5643 | .5959 |

| **Pairwise Comparisons** | | | | | | | | |
| --- | --- | --- | --- | --- | --- | --- | --- | --- |
| Gender | (I) Age | (J) Age | Mean Difference (I-J) | Standard Error | Degrees of Freedom | P | 95% Wald Confidence Interval | |
|  |  |  |  |  |  |  | Lower Bound | Upper Bound |
| 1 | 1 | 2 | .1507 | .01228 | 1 | .000 | .1267 | .1748 |
|  |  | 3 | .2065 | .01368 | 1 | .000 | .1796 | .2333 |
|  |  | 4 | .2236 | .01453 | 1 | .000 | .1951 | .2521 |
|  | 2 | 1 | -.1507 | .01228 | 1 | .000 | -.1748 | -.1267 |
|  |  | 3 | .0557 | .00763 | 1 | .000 | .0408 | .0707 |
|  |  | 4 | .0728 | .00887 | 1 | .000 | .0554 | .0902 |
|  | 3 | 1 | -.2065 | .01368 | 1 | .000 | -.2333 | -.1796 |
|  |  | 2 | -.0557 | .00763 | 1 | .000 | -.0707 | -.0408 |
|  |  | 4 | .0171 | .01061 | 1 | .107 | -.0037 | .0379 |
|  | 4 | 1 | -.2236 | .01453 | 1 | .000 | -.2521 | -.1951 |
|  |  | 2 | -.0728 | .00887 | 1 | .000 | -.0902 | -.0554 |
|  |  | 3 | -.0171 | .01061 | 1 | .107 | -.0379 | .0037 |
| 2 | 1 | 2 | .1887 | .01341 | 1 | .000 | .1624 | .2149 |
|  |  | 3 | .2633 | .01448 | 1 | .000 | .2349 | .2916 |
|  |  | 4 | .2861 | .01526 | 1 | .000 | .2562 | .3160 |
|  | 2 | 1 | -.1887 | .01341 | 1 | .000 | -.2149 | -.1624 |
|  |  | 3 | .0746 | .00740 | 1 | .000 | .0601 | .0891 |
|  |  | 4 | .0974 | .00857 | 1 | .000 | .0806 | .1142 |
|  | 3 | 1 | -.2633 | .01448 | 1 | .000 | -.2916 | -.2349 |
|  |  | 2 | -.0746 | .00740 | 1 | .000 | -.0891 | -.0601 |
|  |  | 4 | .0228 | .01008 | 1 | .024 | .0031 | .0426 |
|  | 4 | 1 | -.2861 | .01526 | 1 | .000 | -.3160 | -.2562 |
|  |  | 2 | -.0974 | .00857 | 1 | .000 | -.1142 | -.0806 |
|  |  | 3 | -.0228 | .01008 | 1 | .024 | -.0426 | -.0031 |

| **Overall Test** | | | |
| --- | --- | --- | --- |
| Gender | Wald χ² | Degrees of Freedom | P |
| 1 | 290.105 | 3 | .000 |
| 2 | 450.032 | 3 | .000 |

**Estimated Marginal Means 13：Gender* Diagnostic**

| **Estimate** | | | | | |
| --- | --- | --- | --- | --- | --- |
| Gender | Diagnostic | Mean | Standard Error | 95% Wald Confidence Interval | |
|  |  |  |  | Lower Bound | Upper Bound |
| 1 | 1 | .6913 | .00421 | .6831 | .6996 |
|  | 2 | .7159 | .00659 | .7032 | .7290 |
| 2 | 1 | .6596 | .00448 | .6509 | .6685 |
|  | 2 | .6864 | .00683 | .6731 | .6999 |

| **Pairwise Comparisons** | | | | | | | | |
| --- | --- | --- | --- | --- | --- | --- | --- | --- |
| Diagnostic | (I) Gender | (J) Gender | Mean Difference (I-J) | Standard Error | Degrees of Freedom | P | 95% Wald Confidence Interval | |
|  |  |  |  |  |  |  | Lower Bound | Upper Bound |
| 1 | 1 | 2 | .0317 | .00484 | 1 | .000 | .0222 | .0411 |
|  | 2 | 1 | -.0317 | .00484 | 1 | .000 | -.0411 | -.0222 |
| 2 | 1 | 2 | .0296 | .00591 | 1 | .000 | .0180 | .0412 |
|  | 2 | 1 | -.0296 | .00591 | 1 | .000 | -.0412 | -.0180 |

| **Overall Test** | | | |
| --- | --- | --- | --- |
| Diagnostic | Wald χ² | Degrees of Freedom | P |
| 1 | 42.784 | 1 | .000 |
| 2 | 25.053 | 1 | .000 |

**Estimated Marginal Means 14：Gender* Diagnostic**

| **Estimate** | | | | | |
| --- | --- | --- | --- | --- | --- |
| Gender | Diagnostic | Mean | Standard Error | 95% Wald Confidence Interval | |
|  |  |  |  | Lower Bound | Upper Bound |
| 1 | 1 | .6913 | .00421 | .6831 | .6996 |
|  | 2 | .7159 | .00659 | .7032 | .7290 |
| 2 | 1 | .6596 | .00448 | .6509 | .6685 |
|  | 2 | .6864 | .00683 | .6731 | .6999 |

| **Pairwise Comparisons** | | | | | | | | |
| --- | --- | --- | --- | --- | --- | --- | --- | --- |
| Gender | (I) Diagnostic | (J) Diagnostic | Mean Difference (I-J) | Standard Error | Degrees of Freedom | P | 95% Wald Confidence Interval | |
|  |  |  |  |  |  |  | Lower Bound | Upper Bound |
| 1 | 1 | 2 | -.0247 | .00712 | 1 | .001 | -.0386 | -.0107 |
|  | 2 | 1 | .0247 | .00712 | 1 | .001 | .0107 | .0386 |
| 2 | 1 | 2 | -.0267 | .00711 | 1 | .000 | -.0407 | -.0128 |
|  | 2 | 1 | .0267 | .00711 | 1 | .000 | .0128 | .0407 |

| **Overall Test** | | | |
| --- | --- | --- | --- |
| Gender | Wald χ² | Degrees of Freedom | P |
| 1 | 12.040 | 1 | .001 |
| 2 | 14.172 | 1 | .000 |

**Estimated Marginal Means 15：Age* Diagnostic**

| **Estimate** | | | | | |
| --- | --- | --- | --- | --- | --- |
| Age | Diagnostic | Mean | Standard Error | 95% Wald Confidence Interval | |
|  |  |  |  | Lower Bound | Upper Bound |
| 1 | 1 | .9098 | .00801 | .8942 | .9256 |
|  | 2 | .8127 | .01855 | .7771 | .8499 |
| 2 | 1 | .6701 | .00349 | .6633 | .6770 |
|  | 2 | .7106 | .00516 | .7006 | .7208 |
| 3 | 1 | .5948 | .00635 | .5824 | .6073 |
|  | 2 | .6560 | .00787 | .6408 | .6716 |
| 4 | 1 | .5734 | .00785 | .5582 | .5890 |
|  | 2 | .6374 | .00861 | .6207 | .6545 |

| **Pairwise Comparisons** | | | | | | | | |
| --- | --- | --- | --- | --- | --- | --- | --- | --- |
| Diagnostic | (I) Age | (J) Age | Mean Difference (I-J) | Standard Error | Degrees of Freedom | P | 95% Wald Confidence Interval | |
|  |  |  |  |  |  |  | Lower Bound | Upper Bound |
| 1 | 1 | 2 | .2397 | .00873 | 1 | .000 | .2226 | .2568 |
|  |  | 3 | .3150 | .01022 | 1 | .000 | .2950 | .3350 |
|  |  | 4 | .3364 | .01120 | 1 | .000 | .3144 | .3583 |
|  | 2 | 1 | -.2397 | .00873 | 1 | .000 | -.2568 | -.2226 |
|  |  | 3 | .0753 | .00696 | 1 | .000 | .0617 | .0890 |
|  |  | 4 | .0967 | .00818 | 1 | .000 | .0807 | .1127 |
|  | 3 | 1 | -.3150 | .01022 | 1 | .000 | -.3350 | -.2950 |
|  |  | 2 | -.0753 | .00696 | 1 | .000 | -.0890 | -.0617 |
|  |  | 4 | .0214 | .00970 | 1 | .028 | .0024 | .0404 |
|  | 4 | 1 | -.3364 | .01120 | 1 | .000 | -.3583 | -.3144 |
|  |  | 2 | -.0967 | .00818 | 1 | .000 | -.1127 | -.0807 |
|  |  | 3 | -.0214 | .00970 | 1 | .028 | -.0404 | -.0024 |
| 2 | 1 | 2 | .1021 | .01827 | 1 | .000 | .0663 | .1379 |
|  |  | 3 | .1567 | .01927 | 1 | .000 | .1189 | .1944 |
|  |  | 4 | .1753 | .01991 | 1 | .000 | .1363 | .2143 |
|  | 2 | 1 | -.1021 | .01827 | 1 | .000 | -.1379 | -.0663 |
|  |  | 3 | .0546 | .00812 | 1 | .000 | .0387 | .0706 |
|  |  | 4 | .0733 | .00930 | 1 | .000 | .0550 | .0915 |
|  | 3 | 1 | -.1567 | .01927 | 1 | .000 | -.1944 | -.1189 |
|  |  | 2 | -.0546 | .00812 | 1 | .000 | -.0706 | -.0387 |
|  |  | 4 | .0186 | .01106 | 1 | .092 | -.0031 | .0403 |
|  | 4 | 1 | -.1753 | .01991 | 1 | .000 | -.2143 | -.1363 |
|  |  | 2 | -.0733 | .00930 | 1 | .000 | -.0915 | -.0550 |
|  |  | 3 | -.0186 | .01106 | 1 | .092 | -.0403 | .0031 |

| **Overall Test** | | | |
| --- | --- | --- | --- |
| Diagnostic | Wald χ² | Degrees of Freedom | P |
| 1 | 1163.853 | 3 | .000 |
| 2 | 133.525 | 3 | .000 |

**Estimated Marginal Means 16：Age* Diagnostic**

| **Estimate** | | | | | |
| --- | --- | --- | --- | --- | --- |
| Age | Diagnostic | Mean | Standard Error | 95% Wald Confidence Interval | |
|  |  |  |  | Lower Bound | Upper Bound |
| 1 | 1 | .9098 | .00801 | .8942 | .9256 |
|  | 2 | .8127 | .01855 | .7771 | .8499 |
| 2 | 1 | .6701 | .00349 | .6633 | .6770 |
|  | 2 | .7106 | .00516 | .7006 | .7208 |
| 3 | 1 | .5948 | .00635 | .5824 | .6073 |
|  | 2 | .6560 | .00787 | .6408 | .6716 |
| 4 | 1 | .5734 | .00785 | .5582 | .5890 |
|  | 2 | .6374 | .00861 | .6207 | .6545 |

| **Pairwise Comparisons** | | | | | | | | |
| --- | --- | --- | --- | --- | --- | --- | --- | --- |
| Age | (I) Diagnostic | (J) Diagnostic | Mean Difference (I-J) | Standard Error | Degrees of Freedom | P | 95% Wald Confidence Interval | |
|  |  |  |  |  |  |  | Lower Bound | Upper Bound |
| 1 | 1 | 2 | .0971 | .01979 | 1 | .000 | .0583 | .1359 |
|  | 2 | 1 | -.0971 | .01979 | 1 | .000 | -.1359 | -.0583 |
| 2 | 1 | 2 | -.0405 | .00589 | 1 | .000 | -.0521 | -.0290 |
|  | 2 | 1 | .0405 | .00589 | 1 | .000 | .0290 | .0521 |
| 3 | 1 | 2 | -.0612 | .00637 | 1 | .000 | -.0737 | -.0488 |
|  | 2 | 1 | .0612 | .00637 | 1 | .000 | .0488 | .0737 |
| 4 | 1 | 2 | -.0640 | .00676 | 1 | .000 | -.0772 | -.0507 |
|  | 2 | 1 | .0640 | .00676 | 1 | .000 | .0507 | .0772 |

| **Overall Test** | | | |
| --- | --- | --- | --- |
| Age | Wald χ² | Degrees of Freedom | P |
| 1 | 24.070 | 1 | .000 |
| 2 | 47.424 | 1 | .000 |
| 3 | 92.509 | 1 | .000 |
| 4 | 89.562 | 1 | .000 |
