# Supplementary material for: The impact of COVID-19 social isolation and reduced microbial exposure on the immune system in children: a retrospective study
Source: PeerJ. 2026 Jul 7;14:e21469. doi: 10.7717/peerj.21469 (PMC13353229; doi:10.7717/peerj.21469)
Supplement: Supplemental Information 7 [file peerj-14-21469-s007.docx]

**Eosinophils Generalized Linear Model**

For CBC analysis, patients were categorized into the following age groups:
Group 1: 0–3 months,
Group 2: 3 months–4 years,
Group 3: 4–6 years,
Group 4: 6–13 years.

Diagnostic 1 is LRTIs . Diagnostic 2 URTIs .

| **Parameter Estimate** | | | | | | | |
| --- | --- | --- | --- | --- | --- | --- | --- |
| Parameter | B | Standard Error | 95% Wald Confidence Interval | | Hypothesis Testing | | |
|  |  |  | Lower Bound | Upper Bound | Wald χ² | Degrees of Freedom | P |
| （Intercept） | -2.058 | .0666 | -2.189 | -1.928 | 953.957 | 1 | .000 |
| [Year=2020] | .054 | .1531 | -.246 | .355 | .127 | 1 | .722 |
| [Year=2021] | .200 | .0860 | .032 | .369 | 5.412 | 1 | .020 |
| [Year=2022] | -.236 | .0758 | -.384 | -.087 | 9.686 | 1 | .002 |
| [Year=2023] | -.455 | .0666 | -.585 | -.324 | 46.646 | 1 | .000 |
| [Year=2024] | -.071 | .0666 | -.201 | .060 | 1.125 | 1 | .289 |
| [Year=2025] | 0 | . | . | . | . | . | . |
| [Male ] | .132 | .0337 | .066 | .198 | 15.463 | 1 | .000 |
| [Female ] | 0 | . | . | . | . | . | . |
| [Age=1] | 1.169 | .0821 | 1.009 | 1.330 | 202.811 | 1 | .000 |
| [Age=2] | .062 | .0644 | -.064 | .188 | .936 | 1 | .333 |
| [Age=3] | .046 | .0754 | -.101 | .194 | .378 | 1 | .539 |
| [Age=4] | 0 | . | . | . | . | . | . |
| [LRTIs ] | .270 | .0374 | .197 | .344 | 52.107 | 1 | .000 |
| [URTIs ] | 0 | . | . | . | . | . | . |
| [Year=2020] * [Male ] | .055 | .0637 | -.070 | .180 | .747 | 1 | .387 |
| [Year=2020] * [Female ] | 0 | . | . | . | . | . | . |
| [Year=2021] * [Male ] | .114 | .0424 | .031 | .197 | 7.289 | 1 | .007 |
| [Year=2021] * [Female ] | 0 | . | . | . | . | . | . |
| [Year=2022] * [Male ] | .104 | .0346 | .036 | .172 | 9.039 | 1 | .003 |
| [Year=2022] * [Female ] | 0 | . | . | . | . | . | . |
| [Year=2023] * [Male ] | .087 | .0299 | .029 | .146 | 8.530 | 1 | .003 |
| [Year=2023] * [Female ] | 0 | . | . | . | . | . | . |
| [Year=2024] * [Male ] | .067 | .0296 | .009 | .125 | 5.159 | 1 | .023 |
| [Year=2024] * [Female ] | 0 | . | . | . | . | . | . |
| [Year=2025] * [Male ] | 0 | . | . | . | . | . | . |
| [Year=2025] * [Female ] | 0 | . | . | . | . | . | . |
| [Year=2020] * [Age=1] | -.652 | .1527 | -.951 | -.353 | 18.225 | 1 | .000 |
| [Year=2020] * [Age=2] | -.414 | .1463 | -.701 | -.128 | 8.021 | 1 | .005 |
| [Year=2020] * [Age=3] | -.259 | .1792 | -.610 | .092 | 2.091 | 1 | .148 |
| [Year=2020] * [Age=4] | 0 | . | . | . | . | . | . |
| [Year=2021] * [Age=1] | -.897 | .0971 | -1.087 | -.706 | 85.209 | 1 | .000 |
| [Year=2021] * [Age=2] | -.265 | .0815 | -.425 | -.106 | 10.603 | 1 | .001 |
| [Year=2021] * [Age=3] | -.184 | .0940 | -.369 | .000 | 3.849 | 1 | .050 |
| [Year=2021] * [Age=4] | 0 | . | . | . | . | . | . |
| [Year=2022] * [Age=1] | -.161 | .0862 | -.330 | .008 | 3.499 | 1 | .061 |
| [Year=2022] * [Age=2] | -.090 | .0729 | -.233 | .052 | 1.537 | 1 | .215 |
| [Year=2022] * [Age=3] | -.157 | .0839 | -.321 | .007 | 3.512 | 1 | .061 |
| [Year=2022] * [Age=4] | 0 | . | . | . | . | . | . |
| [Year=2023] * [Age=1] | .097 | .0740 | -.048 | .242 | 1.717 | 1 | .190 |
| [Year=2023] * [Age=2] | .179 | .0640 | .054 | .305 | 7.837 | 1 | .005 |
| [Year=2023] * [Age=3] | -.093 | .0749 | -.240 | .054 | 1.530 | 1 | .216 |
| [Year=2023] * [Age=4] | 0 | . | . | . | . | . | . |
| [Year=2024] * [Age=1] | -.063 | .0751 | -.210 | .084 | .708 | 1 | .400 |
| [Year=2024] * [Age=2] | -.098 | .0640 | -.223 | .028 | 2.339 | 1 | .126 |
| [Year=2024] * [Age=3] | -.132 | .0750 | -.279 | .015 | 3.089 | 1 | .079 |
| [Year=2024] * [Age=4] | 0 | . | . | . | . | . | . |
| [Year=2025] * [Age=1] | 0 | . | . | . | . | . | . |
| [Year=2025] * [Age=2] | 0 | . | . | . | . | . | . |
| [Year=2025] * [Age=3] | 0 | . | . | . | . | . | . |
| [Year=2025] * [Age=4] | 0 | . | . | . | . | . | . |
| [Year=2020] * [LRTIs ] | .302 | .0819 | .142 | .462 | 13.606 | 1 | .000 |
| [Year=2020] * [URTIs ] | 0 | . | . | . | . | . | . |
| [Year=2021] * [LRTIs ] | .386 | .0469 | .294 | .478 | 67.927 | 1 | .000 |
| [Year=2021] * [URTIs ] | 0 | . | . | . | . | . | . |
| [Year=2022] * [LRTIs ] | .183 | .0391 | .106 | .259 | 21.808 | 1 | .000 |
| [Year=2022] * [URTIs ] | 0 | . | . | . | . | . | . |
| [Year=2023] * [LRTIs ] | .182 | .0346 | .115 | .250 | 27.784 | 1 | .000 |
| [Year=2023] * [URTIs ] | 0 | . | . | . | . | . | . |
| [Year=2024] * [LRTIs ] | .145 | .0343 | .077 | .212 | 17.759 | 1 | .000 |
| [Year=2024] * [URTIs ] | 0 | . | . | . | . | . | . |
| [Year=2025] * [LRTIs ] | 0 | . | . | . | . | . | . |
| [Year=2025] * [URTIs ] | 0 | . | . | . | . | . | . |
| [Male ] * [Age=1] | -.201 | .0345 | -.268 | -.133 | 33.950 | 1 | .000 |
| [Male ] * [Age=2] | -.067 | .0188 | -.104 | -.030 | 12.787 | 1 | .000 |
| [Male ] * [Age=3] | -.023 | .0201 | -.062 | .017 | 1.257 | 1 | .262 |
| [Male ] * [Age=4] | 0 | . | . | . | . | . | . |
| [Female ] * [Age=1] | 0 | . | . | . | . | . | . |
| [Female ] * [Age=2] | 0 | . | . | . | . | . | . |
| [Female ] * [Age=3] | 0 | . | . | . | . | . | . |
| [Female ] * [Age=4] | 0 | . | . | . | . | . | . |
| [Male ] * [LRTIs ] | .028 | .0141 | .001 | .056 | 3.987 | 1 | .046 |
| [Male ] * [URTIs ] | 0 | . | . | . | . | . | . |
| [Female ] * [LRTIs ] | 0 | . | . | . | . | . | . |
| [Female ] * [URTIs ] | 0 | . | . | . | . | . | . |
| [Age=1] * [LRTIs ] | -.518 | .0477 | -.611 | -.424 | 117.583 | 1 | .000 |
| [Age=1] * [URTIs ] | 0 | . | . | . | . | . | . |
| [Age=2] * [LRTIs ] | -.065 | .0190 | -.102 | -.028 | 11.785 | 1 | .001 |
| [Age=2] * [URTIs ] | 0 | . | . | . | . | . | . |
| [Age=3] * [LRTIs ] | .041 | .0205 | .000 | .081 | 3.910 | 1 | .048 |
| [Age=3] * [URTIs ] | 0 | . | . | . | . | . | . |
| [Age=4] * [LRTIs ] | 0 | . | . | . | . | . | . |
| [Age=4] * [URTIs ] | 0 | . | . | . | . | . | . |
| （标度） | .867 | .0039 | .859 | .875 |  |  |  |

**Estimated Marginal Means 1：Year**

| **Estimate** | | | | |
| --- | --- | --- | --- | --- |
| Year | Mean | Standard Error | 95% Wald Confidence Interval | |
|  |  |  | Lower Bound | Upper Bound |
| 2020 | .1767 | .00844 | .1610 | .1941 |
| 2021 | .2185 | .00479 | .2094 | .2281 |
| 2022 | .1605 | .00255 | .1556 | .1656 |
| 2023 | .1483 | .00142 | .1455 | .1511 |
| 2024 | .1877 | .00181 | .1842 | .1913 |
| 2025 | .1950 | .00427 | .1868 | .2036 |

| **Pairwise Comparisons** | | | | | | | |
| --- | --- | --- | --- | --- | --- | --- | --- |
| (I) Year | (J) Year | Mean Difference (I-J) | Standard Error | Degrees of Freedom | P | 95% Wald Confidence Interval | |
|  |  |  |  |  |  | Lower Bound | Upper Bound |
| 2020 | 2021 | -.0418 | .00963 | 1 | .000 | -.0607 | -.0229 |
|  | 2022 | .0162 | .00876 | 1 | .064 | -.0010 | .0334 |
|  | 2023 | .0285 | .00851 | 1 | .001 | .0118 | .0451 |
|  | 2024 | -.0110 | .00859 | 1 | .200 | -.0278 | .0058 |
|  | 2025 | -.0183 | .00937 | 1 | .051 | -.0366 | .0001 |
| 2021 | 2020 | .0418 | .00963 | 1 | .000 | .0229 | .0607 |
|  | 2022 | .0580 | .00533 | 1 | .000 | .0476 | .0684 |
|  | 2023 | .0702 | .00491 | 1 | .000 | .0606 | .0799 |
|  | 2024 | .0308 | .00503 | 1 | .000 | .0209 | .0406 |
|  | 2025 | .0235 | .00627 | 1 | .000 | .0112 | .0358 |
| 2022 | 2020 | -.0162 | .00876 | 1 | .064 | -.0334 | .0010 |
|  | 2021 | -.0580 | .00533 | 1 | .000 | -.0684 | -.0476 |
|  | 2023 | .0122 | .00281 | 1 | .000 | .0067 | .0178 |
|  | 2024 | -.0272 | .00303 | 1 | .000 | -.0331 | -.0213 |
|  | 2025 | -.0345 | .00485 | 1 | .000 | -.0440 | -.0250 |
| 2023 | 2020 | -.0285 | .00851 | 1 | .001 | -.0451 | -.0118 |
|  | 2021 | -.0702 | .00491 | 1 | .000 | -.0799 | -.0606 |
|  | 2022 | -.0122 | .00281 | 1 | .000 | -.0178 | -.0067 |
|  | 2024 | -.0395 | .00218 | 1 | .000 | -.0437 | -.0352 |
|  | 2025 | -.0467 | .00439 | 1 | .000 | -.0553 | -.0381 |
| 2024 | 2020 | .0110 | .00859 | 1 | .200 | -.0058 | .0278 |
|  | 2021 | -.0308 | .00503 | 1 | .000 | -.0406 | -.0209 |
|  | 2022 | .0272 | .00303 | 1 | .000 | .0213 | .0331 |
|  | 2023 | .0395 | .00218 | 1 | .000 | .0352 | .0437 |
|  | 2025 | -.0073 | .00453 | 1 | .108 | -.0161 | .0016 |
| 2025 | 2020 | .0183 | .00937 | 1 | .051 | -.0001 | .0366 |
|  | 2021 | -.0235 | .00627 | 1 | .000 | -.0358 | -.0112 |
|  | 2022 | .0345 | .00485 | 1 | .000 | .0250 | .0440 |
|  | 2023 | .0467 | .00439 | 1 | .000 | .0381 | .0553 |
|  | 2024 | .0073 | .00453 | 1 | .108 | -.0016 | .0161 |

| **Overall Test** | | |
| --- | --- | --- |
| Wald χ² | Degrees of Freedom | P |
| 513.093 | 5 | .000 |

**Estimated Marginal Means 2：Gender**

| **Estimate** | | | | |
| --- | --- | --- | --- | --- |
| Gender | Mean | Standard Error | 95% Wald Confidence Interval | |
|  |  |  | Lower Bound | Upper Bound |
| 1 | .1932 | .00231 | .1887 | .1978 |
| 2 | .1671 | .00226 | .1628 | .1716 |

| **Pairwise Comparisons** | | | | | | | |
| --- | --- | --- | --- | --- | --- | --- | --- |
| (I) Gender | (J) Gender | Mean Difference (I-J) | Standard Error | Degrees of Freedom | P | 95% Wald Confidence Interval | |
|  |  |  |  |  |  | Lower Bound | Upper Bound |
| 1 | 2 | .0261 | .00246 | 1 | .000 | .0213 | .0309 |
| 2 | 1 | -.0261 | .00246 | 1 | .000 | -.0309 | -.0213 |

| **Overall Test** | | |
| --- | --- | --- |
| Wald χ² | Degrees of Freedom | P |
| 112.494 | 1 | .000 |

**Estimated Marginal Means 3：Age**

| **Estimate** | | | | |
| --- | --- | --- | --- | --- |
| Age | Mean | Standard Error | 95% Wald Confidence Interval | |
|  |  |  | Lower Bound | Upper Bound |
| 1 | .2814 | .00676 | .2684 | .2949 |
| 2 | .1469 | .00135 | .1443 | .1496 |
| 3 | .1524 | .00309 | .1465 | .1586 |
| 4 | .1655 | .00423 | .1574 | .1740 |

| **Pairwise Comparisons** | | | | | | | |
| --- | --- | --- | --- | --- | --- | --- | --- |
| (I) Age | (J) Age | Mean Difference (I-J) | Standard Error | Degrees of Freedom | P | 95% Wald Confidence Interval | |
|  |  |  |  |  |  | Lower Bound | Upper Bound |
| 1 | 2 | .1344 | .00679 | 1 | .000 | .1211 | .1477 |
|  | 3 | .1289 | .00732 | 1 | .000 | .1146 | .1433 |
|  | 4 | .1159 | .00792 | 1 | .000 | .1004 | .1314 |
| 2 | 1 | -.1344 | .00679 | 1 | .000 | -.1477 | -.1211 |
|  | 3 | -.0055 | .00331 | 1 | .098 | -.0120 | .0010 |
|  | 4 | -.0185 | .00441 | 1 | .000 | -.0272 | -.0099 |
| 3 | 1 | -.1289 | .00732 | 1 | .000 | -.1433 | -.1146 |
|  | 2 | .0055 | .00331 | 1 | .098 | -.0010 | .0120 |
|  | 4 | -.0130 | .00521 | 1 | .012 | -.0233 | -.0028 |
| 4 | 1 | -.1159 | .00792 | 1 | .000 | -.1314 | -.1004 |
|  | 2 | .0185 | .00441 | 1 | .000 | .0099 | .0272 |
|  | 3 | .0130 | .00521 | 1 | .012 | .0028 | .0233 |

| **Overall Test** | | |
| --- | --- | --- |
| Wald χ² | Degrees of Freedom | P |
| 403.333 | 3 | .000 |

**Estimated Marginal Means 4：Diagnostic**

| **Estimate** | | | | |
| --- | --- | --- | --- | --- |
| Diagnostic | Mean | Standard Error | 95% Wald Confidence Interval | |
|  |  |  | Lower Bound | Upper Bound |
| 1 | .2139 | .00222 | .2096 | .2183 |
| 2 | .1510 | .00260 | .1460 | .1561 |

| **Pairwise Comparisons** | | | | | | | |
| --- | --- | --- | --- | --- | --- | --- | --- |
| (I) Diagnostic | (J) Diagnostic | Mean Difference (I-J) | Standard Error | Degrees of Freedom | P | 95% Wald Confidence Interval | |
|  |  |  |  |  |  | Lower Bound | Upper Bound |
| 1 | 2 | .0629 | .00314 | 1 | .000 | .0568 | .0691 |
| 2 | 1 | -.0629 | .00314 | 1 | .000 | -.0691 | -.0568 |

| **Overall Test** | | |
| --- | --- | --- |
| Wald χ² | Degrees of Freedom | P |
| 402.297 | 1 | .000 |

**Estimated Marginal Means 5：Year* Gender**

| **Estimate** | | | | | |
| --- | --- | --- | --- | --- | --- |
| Year | Gender | Mean | Standard Error | 95% Wald Confidence Interval | |
|  |  |  |  | Lower Bound | Upper Bound |
| 2020 | 1 | .1885 | .00980 | .1703 | .2087 |
|  | 2 | .1657 | .00990 | .1474 | .1863 |
| 2021 | 1 | .2401 | .00624 | .2282 | .2527 |
|  | 2 | .1989 | .00576 | .1879 | .2105 |
| 2022 | 1 | .1755 | .00324 | .1692 | .1819 |
|  | 2 | .1469 | .00301 | .1411 | .1529 |
| 2023 | 1 | .1607 | .00178 | .1573 | .1643 |
|  | 2 | .1368 | .00167 | .1336 | .1401 |
| 2024 | 1 | .2015 | .00223 | .1971 | .2059 |
|  | 2 | .1750 | .00212 | .1709 | .1792 |
| 2025 | 1 | .2023 | .00497 | .1928 | .2123 |
|  | 2 | .1880 | .00505 | .1783 | .1981 |

| **Pairwise Comparisons** | | | | | | | | |
| --- | --- | --- | --- | --- | --- | --- | --- | --- |
| Gender | (I) Year | (J) Year | Mean Difference (I-J) | Standard Error | Degrees of Freedom | P | 95% Wald Confidence Interval | |
|  |  |  |  |  |  |  | Lower Bound | Upper Bound |
| 1 | 2020 | 2021 | -.0516 | .01155 | 1 | .000 | -.0742 | -.0289 |
|  |  | 2022 | .0131 | .01027 | 1 | .204 | -.0071 | .0332 |
|  |  | 2023 | .0278 | .00992 | 1 | .005 | .0083 | .0472 |
|  |  | 2024 | -.0129 | .01001 | 1 | .196 | -.0326 | .0067 |
|  |  | 2025 | -.0138 | .01088 | 1 | .204 | -.0352 | .0075 |
|  | 2021 | 2020 | .0516 | .01155 | 1 | .000 | .0289 | .0742 |
|  |  | 2022 | .0646 | .00690 | 1 | .000 | .0511 | .0782 |
|  |  | 2023 | .0794 | .00637 | 1 | .000 | .0669 | .0919 |
|  |  | 2024 | .0386 | .00649 | 1 | .000 | .0259 | .0514 |
|  |  | 2025 | .0378 | .00783 | 1 | .000 | .0224 | .0531 |
|  | 2022 | 2020 | -.0131 | .01027 | 1 | .204 | -.0332 | .0071 |
|  |  | 2021 | -.0646 | .00690 | 1 | .000 | -.0782 | -.0511 |
|  |  | 2023 | .0147 | .00353 | 1 | .000 | .0078 | .0217 |
|  |  | 2024 | -.0260 | .00376 | 1 | .000 | -.0334 | -.0186 |
|  |  | 2025 | -.0269 | .00579 | 1 | .000 | -.0382 | -.0155 |
|  | 2023 | 2020 | -.0278 | .00992 | 1 | .005 | -.0472 | -.0083 |
|  |  | 2021 | -.0794 | .00637 | 1 | .000 | -.0919 | -.0669 |
|  |  | 2022 | -.0147 | .00353 | 1 | .000 | -.0217 | -.0078 |
|  |  | 2024 | -.0407 | .00264 | 1 | .000 | -.0459 | -.0356 |
|  |  | 2025 | -.0416 | .00515 | 1 | .000 | -.0517 | -.0315 |
|  | 2024 | 2020 | .0129 | .01001 | 1 | .196 | -.0067 | .0326 |
|  |  | 2021 | -.0386 | .00649 | 1 | .000 | -.0514 | -.0259 |
|  |  | 2022 | .0260 | .00376 | 1 | .000 | .0186 | .0334 |
|  |  | 2023 | .0407 | .00264 | 1 | .000 | .0356 | .0459 |
|  |  | 2025 | -.0009 | .00532 | 1 | .868 | -.0113 | .0095 |
|  | 2025 | 2020 | .0138 | .01088 | 1 | .204 | -.0075 | .0352 |
|  |  | 2021 | -.0378 | .00783 | 1 | .000 | -.0531 | -.0224 |
|  |  | 2022 | .0269 | .00579 | 1 | .000 | .0155 | .0382 |
|  |  | 2023 | .0416 | .00515 | 1 | .000 | .0315 | .0517 |
|  |  | 2024 | .0009 | .00532 | 1 | .868 | -.0095 | .0113 |
| 2 | 2020 | 2021 | -.0332 | .01139 | 1 | .004 | -.0555 | -.0109 |
|  |  | 2022 | .0188 | .01030 | 1 | .067 | -.0014 | .0390 |
|  |  | 2023 | .0289 | .01002 | 1 | .004 | .0093 | .0485 |
|  |  | 2024 | -.0092 | .01010 | 1 | .360 | -.0290 | .0105 |
|  |  | 2025 | -.0222 | .01099 | 1 | .043 | -.0438 | -.0007 |
|  | 2021 | 2020 | .0332 | .01139 | 1 | .004 | .0109 | .0555 |
|  |  | 2022 | .0520 | .00637 | 1 | .000 | .0395 | .0645 |
|  |  | 2023 | .0621 | .00588 | 1 | .000 | .0506 | .0736 |
|  |  | 2024 | .0239 | .00600 | 1 | .000 | .0122 | .0357 |
|  |  | 2025 | .0110 | .00752 | 1 | .145 | -.0038 | .0257 |
|  | 2022 | 2020 | -.0188 | .01030 | 1 | .067 | -.0390 | .0014 |
|  |  | 2021 | -.0520 | .00637 | 1 | .000 | -.0645 | -.0395 |
|  |  | 2023 | .0101 | .00327 | 1 | .002 | .0037 | .0165 |
|  |  | 2024 | -.0281 | .00349 | 1 | .000 | -.0349 | -.0213 |
|  |  | 2025 | -.0411 | .00575 | 1 | .000 | -.0523 | -.0298 |
|  | 2023 | 2020 | -.0289 | .01002 | 1 | .004 | -.0485 | -.0093 |
|  |  | 2021 | -.0621 | .00588 | 1 | .000 | -.0736 | -.0506 |
|  |  | 2022 | -.0101 | .00327 | 1 | .002 | -.0165 | -.0037 |
|  |  | 2024 | -.0382 | .00244 | 1 | .000 | -.0429 | -.0334 |
|  |  | 2025 | -.0511 | .00523 | 1 | .000 | -.0614 | -.0409 |
|  | 2024 | 2020 | .0092 | .01010 | 1 | .360 | -.0105 | .0290 |
|  |  | 2021 | -.0239 | .00600 | 1 | .000 | -.0357 | -.0122 |
|  |  | 2022 | .0281 | .00349 | 1 | .000 | .0213 | .0349 |
|  |  | 2023 | .0382 | .00244 | 1 | .000 | .0334 | .0429 |
|  |  | 2025 | -.0130 | .00538 | 1 | .016 | -.0235 | -.0024 |
|  | 2025 | 2020 | .0222 | .01099 | 1 | .043 | .0007 | .0438 |
|  |  | 2021 | -.0110 | .00752 | 1 | .145 | -.0257 | .0038 |
|  |  | 2022 | .0411 | .00575 | 1 | .000 | .0298 | .0523 |
|  |  | 2023 | .0511 | .00523 | 1 | .000 | .0409 | .0614 |
|  |  | 2024 | .0130 | .00538 | 1 | .016 | .0024 | .0235 |

| **Overall Test** | | | |
| --- | --- | --- | --- |
| Gender | Wald χ² | Degrees of Freedom | P |
| 1 | 364.321 | 5 | .000 |
| 2 | 365.341 | 5 | .000 |

**Estimated Marginal Means 6：Year* Gender**

| **Estimate** | | | | | |
| --- | --- | --- | --- | --- | --- |
| Year | Gender | Mean | Standard Error | 95% Wald Confidence Interval | |
|  |  |  |  | Lower Bound | Upper Bound |
| 2020 | 1 | .1885 | .00980 | .1703 | .2087 |
|  | 2 | .1657 | .00990 | .1474 | .1863 |
| 2021 | 1 | .2401 | .00624 | .2282 | .2527 |
|  | 2 | .1989 | .00576 | .1879 | .2105 |
| 2022 | 1 | .1755 | .00324 | .1692 | .1819 |
|  | 2 | .1469 | .00301 | .1411 | .1529 |
| 2023 | 1 | .1607 | .00178 | .1573 | .1643 |
|  | 2 | .1368 | .00167 | .1336 | .1401 |
| 2024 | 1 | .2015 | .00223 | .1971 | .2059 |
|  | 2 | .1750 | .00212 | .1709 | .1792 |
| 2025 | 1 | .2023 | .00497 | .1928 | .2123 |
|  | 2 | .1880 | .00505 | .1783 | .1981 |

| **Pairwise Comparisons** | | | | | | | | |
| --- | --- | --- | --- | --- | --- | --- | --- | --- |
| Year | (I) Gender | (J) Gender | Mean Difference (I-J) | Standard Error | Degrees of Freedom | P | 95% Wald Confidence Interval | |
|  |  |  |  |  |  |  | Lower Bound | Upper Bound |
| 2020 | 1 | 2 | .0228 | .01025 | 1 | .026 | .0027 | .0429 |
|  | 2 | 1 | -.0228 | .01025 | 1 | .026 | -.0429 | -.0027 |
| 2021 | 1 | 2 | .0412 | .00727 | 1 | .000 | .0269 | .0554 |
|  | 2 | 1 | -.0412 | .00727 | 1 | .000 | -.0554 | -.0269 |
| 2022 | 1 | 2 | .0286 | .00362 | 1 | .000 | .0215 | .0357 |
|  | 2 | 1 | -.0286 | .00362 | 1 | .000 | -.0357 | -.0215 |
| 2023 | 1 | 2 | .0239 | .00195 | 1 | .000 | .0201 | .0277 |
|  | 2 | 1 | -.0239 | .00195 | 1 | .000 | -.0277 | -.0201 |
| 2024 | 1 | 2 | .0265 | .00242 | 1 | .000 | .0217 | .0312 |
|  | 2 | 1 | -.0265 | .00242 | 1 | .000 | -.0312 | -.0217 |
| 2025 | 1 | 2 | .0144 | .00523 | 1 | .006 | .0041 | .0247 |
|  | 2 | 1 | -.0144 | .00523 | 1 | .006 | -.0247 | -.0041 |

| **Overall Test** | | | |
| --- | --- | --- | --- |
| Year | Wald χ² | Degrees of Freedom | P |
| 2020 | 4.949 | 1 | .026 |
| 2021 | 32.062 | 1 | .000 |
| 2022 | 62.211 | 1 | .000 |
| 2023 | 150.773 | 1 | .000 |
| 2024 | 119.376 | 1 | .000 |
| 2025 | 7.567 | 1 | .006 |

**Estimated Marginal Means 7：Year* Age**

| **Estimate** | | | | | |
| --- | --- | --- | --- | --- | --- |
| Year | Age | Mean | Standard Error | 95% Wald Confidence Interval | |
|  |  |  |  | Lower Bound | Upper Bound |
| 2020 | 1 | .2325 | .01450 | .2058 | .2628 |
|  | 2 | .1306 | .00568 | .1200 | .1422 |
|  | 3 | .1619 | .01697 | .1318 | .1988 |
|  | 4 | .1985 | .02504 | .1550 | .2542 |
| 2021 | 1 | .2263 | .01278 | .2026 | .2527 |
|  | 2 | .1885 | .00407 | .1806 | .1966 |
|  | 3 | .2168 | .00757 | .2025 | .2322 |
|  | 4 | .2467 | .01189 | .2245 | .2711 |
| 2022 | 1 | .2743 | .01239 | .2511 | .2997 |
|  | 2 | .1304 | .00186 | .1269 | .1341 |
|  | 3 | .1295 | .00268 | .1243 | .1348 |
|  | 4 | .1433 | .00503 | .1338 | .1535 |
| 2023 | 1 | .2829 | .00961 | .2646 | .3024 |
|  | 2 | .1361 | .00119 | .1338 | .1384 |
|  | 3 | .1100 | .00110 | .1079 | .1122 |
|  | 4 | .1142 | .00129 | .1117 | .1168 |
| 2024 | 1 | .3437 | .01174 | .3215 | .3676 |
|  | 2 | .1471 | .00115 | .1449 | .1494 |
|  | 3 | .1509 | .00146 | .1480 | .1538 |
|  | 4 | .1629 | .00198 | .1590 | .1668 |
| 2025 | 1 | .3535 | .01276 | .3293 | .3794 |
|  | 2 | .1566 | .00287 | .1511 | .1623 |
|  | 3 | .1662 | .00709 | .1528 | .1807 |
|  | 4 | .1572 | .00945 | .1397 | .1769 |

| **Pairwise Comparisons** | | | | | | | | |
| --- | --- | --- | --- | --- | --- | --- | --- | --- |
| Age | (I) Year | (J) Year | Mean Difference (I-J) | Standard Error | Degrees of Freedom | P | 95% Wald Confidence Interval | |
|  |  |  |  |  |  |  | Lower Bound | Upper Bound |
| 1 | 2020 | 2021 | .0063 | .01841 | 1 | .734 | -.0298 | .0424 |
|  |  | 2022 | -.0418 | .01805 | 1 | .021 | -.0772 | -.0064 |
|  |  | 2023 | -.0503 | .01626 | 1 | .002 | -.0822 | -.0185 |
|  |  | 2024 | -.1112 | .01765 | 1 | .000 | -.1458 | -.0766 |
|  |  | 2025 | -.1210 | .01737 | 1 | .000 | -.1550 | -.0869 |
|  | 2021 | 2020 | -.0063 | .01841 | 1 | .734 | -.0424 | .0298 |
|  |  | 2022 | -.0481 | .01694 | 1 | .005 | -.0813 | -.0148 |
|  |  | 2023 | -.0566 | .01503 | 1 | .000 | -.0861 | -.0272 |
|  |  | 2024 | -.1175 | .01651 | 1 | .000 | -.1499 | -.0851 |
|  |  | 2025 | -.1272 | .01643 | 1 | .000 | -.1594 | -.0950 |
|  | 2022 | 2020 | .0418 | .01805 | 1 | .021 | .0064 | .0772 |
|  |  | 2021 | .0481 | .01694 | 1 | .005 | .0148 | .0813 |
|  |  | 2023 | -.0086 | .01458 | 1 | .557 | -.0371 | .0200 |
|  |  | 2024 | -.0694 | .01611 | 1 | .000 | -.1010 | -.0379 |
|  |  | 2025 | -.0792 | .01595 | 1 | .000 | -.1104 | -.0479 |
|  | 2023 | 2020 | .0503 | .01626 | 1 | .002 | .0185 | .0822 |
|  |  | 2021 | .0566 | .01503 | 1 | .000 | .0272 | .0861 |
|  |  | 2022 | .0086 | .01458 | 1 | .557 | -.0200 | .0371 |
|  |  | 2024 | -.0609 | .01406 | 1 | .000 | -.0884 | -.0333 |
|  |  | 2025 | -.0706 | .01391 | 1 | .000 | -.0979 | -.0434 |
|  | 2024 | 2020 | .1112 | .01765 | 1 | .000 | .0766 | .1458 |
|  |  | 2021 | .1175 | .01651 | 1 | .000 | .0851 | .1499 |
|  |  | 2022 | .0694 | .01611 | 1 | .000 | .0379 | .1010 |
|  |  | 2023 | .0609 | .01406 | 1 | .000 | .0333 | .0884 |
|  |  | 2025 | -.0097 | .01555 | 1 | .531 | -.0402 | .0207 |
|  | 2025 | 2020 | .1210 | .01737 | 1 | .000 | .0869 | .1550 |
|  |  | 2021 | .1272 | .01643 | 1 | .000 | .0950 | .1594 |
|  |  | 2022 | .0792 | .01595 | 1 | .000 | .0479 | .1104 |
|  |  | 2023 | .0706 | .01391 | 1 | .000 | .0434 | .0979 |
|  |  | 2024 | .0097 | .01555 | 1 | .531 | -.0207 | .0402 |
| 2 | 2020 | 2021 | -.0578 | .00699 | 1 | .000 | -.0715 | -.0441 |
|  |  | 2022 | .0002 | .00597 | 1 | .976 | -.0115 | .0119 |
|  |  | 2023 | -.0054 | .00580 | 1 | .349 | -.0168 | .0059 |
|  |  | 2024 | -.0165 | .00579 | 1 | .004 | -.0278 | -.0051 |
|  |  | 2025 | -.0260 | .00635 | 1 | .000 | -.0384 | -.0135 |
|  | 2021 | 2020 | .0578 | .00699 | 1 | .000 | .0441 | .0715 |
|  |  | 2022 | .0580 | .00448 | 1 | .000 | .0492 | .0668 |
|  |  | 2023 | .0524 | .00424 | 1 | .000 | .0441 | .0607 |
|  |  | 2024 | .0414 | .00423 | 1 | .000 | .0331 | .0496 |
|  |  | 2025 | .0318 | .00498 | 1 | .000 | .0221 | .0416 |
|  | 2022 | 2020 | -.0002 | .00597 | 1 | .976 | -.0119 | .0115 |
|  |  | 2021 | -.0580 | .00448 | 1 | .000 | -.0668 | -.0492 |
|  |  | 2023 | -.0056 | .00220 | 1 | .011 | -.0099 | -.0013 |
|  |  | 2024 | -.0167 | .00218 | 1 | .000 | -.0209 | -.0124 |
|  |  | 2025 | -.0262 | .00342 | 1 | .000 | -.0329 | -.0195 |
|  | 2023 | 2020 | .0054 | .00580 | 1 | .349 | -.0059 | .0168 |
|  |  | 2021 | -.0524 | .00424 | 1 | .000 | -.0607 | -.0441 |
|  |  | 2022 | .0056 | .00220 | 1 | .011 | .0013 | .0099 |
|  |  | 2024 | -.0110 | .00164 | 1 | .000 | -.0143 | -.0078 |
|  |  | 2025 | -.0205 | .00310 | 1 | .000 | -.0266 | -.0145 |
|  | 2024 | 2020 | .0165 | .00579 | 1 | .004 | .0051 | .0278 |
|  |  | 2021 | -.0414 | .00423 | 1 | .000 | -.0496 | -.0331 |
|  |  | 2022 | .0167 | .00218 | 1 | .000 | .0124 | .0209 |
|  |  | 2023 | .0110 | .00164 | 1 | .000 | .0078 | .0143 |
|  |  | 2025 | -.0095 | .00309 | 1 | .002 | -.0156 | -.0035 |
|  | 2025 | 2020 | .0260 | .00635 | 1 | .000 | .0135 | .0384 |
|  |  | 2021 | -.0318 | .00498 | 1 | .000 | -.0416 | -.0221 |
|  |  | 2022 | .0262 | .00342 | 1 | .000 | .0195 | .0329 |
|  |  | 2023 | .0205 | .00310 | 1 | .000 | .0145 | .0266 |
|  |  | 2024 | .0095 | .00309 | 1 | .002 | .0035 | .0156 |
| 3 | 2020 | 2021 | -.0549 | .01857 | 1 | .003 | -.0913 | -.0186 |
|  |  | 2022 | .0324 | .01717 | 1 | .059 | -.0012 | .0661 |
|  |  | 2023 | .0519 | .01700 | 1 | .002 | .0186 | .0852 |
|  |  | 2024 | .0110 | .01702 | 1 | .518 | -.0224 | .0444 |
|  |  | 2025 | -.0043 | .01838 | 1 | .815 | -.0403 | .0317 |
|  | 2021 | 2020 | .0549 | .01857 | 1 | .003 | .0186 | .0913 |
|  |  | 2022 | .0874 | .00802 | 1 | .000 | .0716 | .1031 |
|  |  | 2023 | .1068 | .00765 | 1 | .000 | .0918 | .1218 |
|  |  | 2024 | .0660 | .00770 | 1 | .000 | .0509 | .0810 |
|  |  | 2025 | .0507 | .01036 | 1 | .000 | .0304 | .0710 |
|  | 2022 | 2020 | -.0324 | .01717 | 1 | .059 | -.0661 | .0012 |
|  |  | 2021 | -.0874 | .00802 | 1 | .000 | -.1031 | -.0716 |
|  |  | 2023 | .0195 | .00289 | 1 | .000 | .0138 | .0251 |
|  |  | 2024 | -.0214 | .00303 | 1 | .000 | -.0273 | -.0155 |
|  |  | 2025 | -.0367 | .00757 | 1 | .000 | -.0515 | -.0219 |
|  | 2023 | 2020 | -.0519 | .01700 | 1 | .002 | -.0852 | -.0186 |
|  |  | 2021 | -.1068 | .00765 | 1 | .000 | -.1218 | -.0918 |
|  |  | 2022 | -.0195 | .00289 | 1 | .000 | -.0251 | -.0138 |
|  |  | 2024 | -.0409 | .00182 | 1 | .000 | -.0444 | -.0373 |
|  |  | 2025 | -.0562 | .00717 | 1 | .000 | -.0702 | -.0421 |
|  | 2024 | 2020 | -.0110 | .01702 | 1 | .518 | -.0444 | .0224 |
|  |  | 2021 | -.0660 | .00770 | 1 | .000 | -.0810 | -.0509 |
|  |  | 2022 | .0214 | .00303 | 1 | .000 | .0155 | .0273 |
|  |  | 2023 | .0409 | .00182 | 1 | .000 | .0373 | .0444 |
|  |  | 2025 | -.0153 | .00722 | 1 | .034 | -.0295 | -.0011 |
|  | 2025 | 2020 | .0043 | .01838 | 1 | .815 | -.0317 | .0403 |
|  |  | 2021 | -.0507 | .01036 | 1 | .000 | -.0710 | -.0304 |
|  |  | 2022 | .0367 | .00757 | 1 | .000 | .0219 | .0515 |
|  |  | 2023 | .0562 | .00717 | 1 | .000 | .0421 | .0702 |
|  |  | 2024 | .0153 | .00722 | 1 | .034 | .0011 | .0295 |
| 4 | 2020 | 2021 | -.0482 | .02771 | 1 | .082 | -.1025 | .0061 |
|  |  | 2022 | .0551 | .02553 | 1 | .031 | .0051 | .1052 |
|  |  | 2023 | .0843 | .02507 | 1 | .001 | .0351 | .1334 |
|  |  | 2024 | .0356 | .02511 | 1 | .156 | -.0136 | .0848 |
|  |  | 2025 | .0412 | .02676 | 1 | .123 | -.0112 | .0937 |
|  | 2021 | 2020 | .0482 | .02771 | 1 | .082 | -.0061 | .1025 |
|  |  | 2022 | .1033 | .01289 | 1 | .000 | .0781 | .1286 |
|  |  | 2023 | .1325 | .01195 | 1 | .000 | .1091 | .1559 |
|  |  | 2024 | .0838 | .01203 | 1 | .000 | .0603 | .1074 |
|  |  | 2025 | .0895 | .01517 | 1 | .000 | .0597 | .1192 |
|  | 2022 | 2020 | -.0551 | .02553 | 1 | .031 | -.1052 | -.0051 |
|  |  | 2021 | -.1033 | .01289 | 1 | .000 | -.1286 | -.0781 |
|  |  | 2023 | .0292 | .00518 | 1 | .000 | .0190 | .0393 |
|  |  | 2024 | -.0195 | .00539 | 1 | .000 | -.0301 | -.0090 |
|  |  | 2025 | -.0139 | .01070 | 1 | .195 | -.0348 | .0071 |
|  | 2023 | 2020 | -.0843 | .02507 | 1 | .001 | -.1334 | -.0351 |
|  |  | 2021 | -.1325 | .01195 | 1 | .000 | -.1559 | -.1091 |
|  |  | 2022 | -.0292 | .00518 | 1 | .000 | -.0393 | -.0190 |
|  |  | 2024 | -.0487 | .00234 | 1 | .000 | -.0533 | -.0441 |
|  |  | 2025 | -.0430 | .00954 | 1 | .000 | -.0617 | -.0243 |
|  | 2024 | 2020 | -.0356 | .02511 | 1 | .156 | -.0848 | .0136 |
|  |  | 2021 | -.0838 | .01203 | 1 | .000 | -.1074 | -.0603 |
|  |  | 2022 | .0195 | .00539 | 1 | .000 | .0090 | .0301 |
|  |  | 2023 | .0487 | .00234 | 1 | .000 | .0441 | .0533 |
|  |  | 2025 | .0056 | .00965 | 1 | .559 | -.0133 | .0245 |
|  | 2025 | 2020 | -.0412 | .02676 | 1 | .123 | -.0937 | .0112 |
|  |  | 2021 | -.0895 | .01517 | 1 | .000 | -.1192 | -.0597 |
|  |  | 2022 | .0139 | .01070 | 1 | .195 | -.0071 | .0348 |
|  |  | 2023 | .0430 | .00954 | 1 | .000 | .0243 | .0617 |
|  |  | 2024 | -.0056 | .00965 | 1 | .559 | -.0245 | .0133 |

| **Overall Test** | | | |
| --- | --- | --- | --- |
| Age | Wald χ² | Degrees of Freedom | P |
| 1 | 105.498 | 5 | .000 |
| 2 | 243.675 | 5 | .000 |
| 3 | 685.407 | 5 | .000 |
| 4 | 549.984 | 5 | .000 |

**Estimated Marginal Means 8：Year* Age**

| **Estimate** | | | | | |
| --- | --- | --- | --- | --- | --- |
| Year | Age | Mean | Standard Error | 95% Wald Confidence Interval | |
|  |  |  |  | Lower Bound | Upper Bound |
| 2020 | 1 | .2325 | .01450 | .2058 | .2628 |
|  | 2 | .1306 | .00568 | .1200 | .1422 |
|  | 3 | .1619 | .01697 | .1318 | .1988 |
|  | 4 | .1985 | .02504 | .1550 | .2542 |
| 2021 | 1 | .2263 | .01278 | .2026 | .2527 |
|  | 2 | .1885 | .00407 | .1806 | .1966 |
|  | 3 | .2168 | .00757 | .2025 | .2322 |
|  | 4 | .2467 | .01189 | .2245 | .2711 |
| 2022 | 1 | .2743 | .01239 | .2511 | .2997 |
|  | 2 | .1304 | .00186 | .1269 | .1341 |
|  | 3 | .1295 | .00268 | .1243 | .1348 |
|  | 4 | .1433 | .00503 | .1338 | .1535 |
| 2023 | 1 | .2829 | .00961 | .2646 | .3024 |
|  | 2 | .1361 | .00119 | .1338 | .1384 |
|  | 3 | .1100 | .00110 | .1079 | .1122 |
|  | 4 | .1142 | .00129 | .1117 | .1168 |
| 2024 | 1 | .3437 | .01174 | .3215 | .3676 |
|  | 2 | .1471 | .00115 | .1449 | .1494 |
|  | 3 | .1509 | .00146 | .1480 | .1538 |
|  | 4 | .1629 | .00198 | .1590 | .1668 |
| 2025 | 1 | .3535 | .01276 | .3293 | .3794 |
|  | 2 | .1566 | .00287 | .1511 | .1623 |
|  | 3 | .1662 | .00709 | .1528 | .1807 |
|  | 4 | .1572 | .00945 | .1397 | .1769 |

| **Pairwise Comparisons** | | | | | | | | |
| --- | --- | --- | --- | --- | --- | --- | --- | --- |
| Year | (I) Age | (J) Age | Mean Difference (I-J) | Standard Error | Degrees of Freedom | P | 95% Wald Confidence Interval | |
|  |  |  |  |  |  |  | Lower Bound | Upper Bound |
| 2020 | 1 | 2 | .1019 | .01431 | 1 | .000 | .0738 | .1300 |
|  |  | 3 | .0706 | .02131 | 1 | .001 | .0289 | .1124 |
|  |  | 4 | .0341 | .02859 | 1 | .234 | -.0220 | .0901 |
|  | 2 | 1 | -.1019 | .01431 | 1 | .000 | -.1300 | -.0738 |
|  |  | 3 | -.0313 | .01756 | 1 | .075 | -.0657 | .0032 |
|  |  | 4 | -.0678 | .02553 | 1 | .008 | -.1179 | -.0178 |
|  | 3 | 1 | -.0706 | .02131 | 1 | .001 | -.1124 | -.0289 |
|  |  | 2 | .0313 | .01756 | 1 | .075 | -.0032 | .0657 |
|  |  | 4 | -.0366 | .03016 | 1 | .225 | -.0957 | .0225 |
|  | 4 | 1 | -.0341 | .02859 | 1 | .234 | -.0901 | .0220 |
|  |  | 2 | .0678 | .02553 | 1 | .008 | .0178 | .1179 |
|  |  | 3 | .0366 | .03016 | 1 | .225 | -.0225 | .0957 |
| 2021 | 1 | 2 | .0378 | .01337 | 1 | .005 | .0116 | .0640 |
|  |  | 3 | .0094 | .01463 | 1 | .519 | -.0193 | .0381 |
|  |  | 4 | -.0204 | .01717 | 1 | .234 | -.0541 | .0132 |
|  | 2 | 1 | -.0378 | .01337 | 1 | .005 | -.0640 | -.0116 |
|  |  | 3 | -.0284 | .00854 | 1 | .001 | -.0451 | -.0116 |
|  |  | 4 | -.0582 | .01251 | 1 | .000 | -.0828 | -.0337 |
|  | 3 | 1 | -.0094 | .01463 | 1 | .519 | -.0381 | .0193 |
|  |  | 2 | .0284 | .00854 | 1 | .001 | .0116 | .0451 |
|  |  | 4 | -.0299 | .01393 | 1 | .032 | -.0572 | -.0026 |
|  | 4 | 1 | .0204 | .01717 | 1 | .234 | -.0132 | .0541 |
|  |  | 2 | .0582 | .01251 | 1 | .000 | .0337 | .0828 |
|  |  | 3 | .0299 | .01393 | 1 | .032 | .0026 | .0572 |
| 2022 | 1 | 2 | .1439 | .01249 | 1 | .000 | .1194 | .1683 |
|  |  | 3 | .1448 | .01262 | 1 | .000 | .1201 | .1696 |
|  |  | 4 | .1310 | .01334 | 1 | .000 | .1048 | .1571 |
|  | 2 | 1 | -.1439 | .01249 | 1 | .000 | -.1683 | -.1194 |
|  |  | 3 | .0010 | .00323 | 1 | .763 | -.0053 | .0073 |
|  |  | 4 | -.0129 | .00534 | 1 | .016 | -.0234 | -.0024 |
|  | 3 | 1 | -.1448 | .01262 | 1 | .000 | -.1696 | -.1201 |
|  |  | 2 | -.0010 | .00323 | 1 | .763 | -.0073 | .0053 |
|  |  | 4 | -.0139 | .00568 | 1 | .015 | -.0250 | -.0027 |
|  | 4 | 1 | -.1310 | .01334 | 1 | .000 | -.1571 | -.1048 |
|  |  | 2 | .0129 | .00534 | 1 | .016 | .0024 | .0234 |
|  |  | 3 | .0139 | .00568 | 1 | .015 | .0027 | .0250 |
| 2023 | 1 | 2 | .1468 | .00968 | 1 | .000 | .1278 | .1658 |
|  |  | 3 | .1729 | .00967 | 1 | .000 | .1539 | .1918 |
|  |  | 4 | .1687 | .00969 | 1 | .000 | .1497 | .1877 |
|  | 2 | 1 | -.1468 | .00968 | 1 | .000 | -.1658 | -.1278 |
|  |  | 3 | .0261 | .00161 | 1 | .000 | .0229 | .0292 |
|  |  | 4 | .0219 | .00175 | 1 | .000 | .0184 | .0253 |
|  | 3 | 1 | -.1729 | .00967 | 1 | .000 | -.1918 | -.1539 |
|  |  | 2 | -.0261 | .00161 | 1 | .000 | -.0292 | -.0229 |
|  |  | 4 | -.0042 | .00169 | 1 | .013 | -.0075 | -.0009 |
|  | 4 | 1 | -.1687 | .00969 | 1 | .000 | -.1877 | -.1497 |
|  |  | 2 | -.0219 | .00175 | 1 | .000 | -.0253 | -.0184 |
|  |  | 3 | .0042 | .00169 | 1 | .013 | .0009 | .0075 |
| 2024 | 1 | 2 | .1966 | .01179 | 1 | .000 | .1735 | .2198 |
|  |  | 3 | .1929 | .01182 | 1 | .000 | .1697 | .2160 |
|  |  | 4 | .1809 | .01190 | 1 | .000 | .1576 | .2042 |
|  | 2 | 1 | -.1966 | .01179 | 1 | .000 | -.2198 | -.1735 |
|  |  | 3 | -.0038 | .00185 | 1 | .041 | -.0074 | -.0002 |
|  |  | 4 | -.0158 | .00228 | 1 | .000 | -.0202 | -.0113 |
|  | 3 | 1 | -.1929 | .01182 | 1 | .000 | -.2160 | -.1697 |
|  |  | 2 | .0038 | .00185 | 1 | .041 | .0002 | .0074 |
|  |  | 4 | -.0120 | .00245 | 1 | .000 | -.0168 | -.0072 |
|  | 4 | 1 | -.1809 | .01190 | 1 | .000 | -.2042 | -.1576 |
|  |  | 2 | .0158 | .00228 | 1 | .000 | .0113 | .0202 |
|  |  | 3 | .0120 | .00245 | 1 | .000 | .0072 | .0168 |
| 2025 | 1 | 2 | .1969 | .01263 | 1 | .000 | .1721 | .2216 |
|  |  | 3 | .1873 | .01428 | 1 | .000 | .1593 | .2153 |
|  |  | 4 | .1963 | .01572 | 1 | .000 | .1655 | .2271 |
|  | 2 | 1 | -.1969 | .01263 | 1 | .000 | -.2216 | -.1721 |
|  |  | 3 | -.0096 | .00752 | 1 | .204 | -.0243 | .0052 |
|  |  | 4 | -.0006 | .00982 | 1 | .950 | -.0199 | .0186 |
|  | 3 | 1 | -.1873 | .01428 | 1 | .000 | -.2153 | -.1593 |
|  |  | 2 | .0096 | .00752 | 1 | .204 | -.0052 | .0243 |
|  |  | 4 | .0090 | .01178 | 1 | .447 | -.0141 | .0320 |
|  | 4 | 1 | -.1963 | .01572 | 1 | .000 | -.2271 | -.1655 |
|  |  | 2 | .0006 | .00982 | 1 | .950 | -.0186 | .0199 |
|  |  | 3 | -.0090 | .01178 | 1 | .447 | -.0320 | .0141 |

| **Overall Test** | | | |
| --- | --- | --- | --- |
| Year | Wald χ² | Degrees of Freedom | P |
| 2020 | 56.549 | 3 | .000 |
| 2021 | 32.038 | 3 | .000 |
| 2022 | 138.962 | 3 | .000 |
| 2023 | 573.578 | 3 | .000 |
| 2024 | 316.322 | 3 | .000 |
| 2025 | 243.221 | 3 | .000 |

**Estimated Marginal Means 9：Year* Diagnostic**

| **Estimate** | | | | | |
| --- | --- | --- | --- | --- | --- |
| Year | Diagnostic | Mean | Standard Error | 95% Wald Confidence Interval | |
|  |  |  |  | Lower Bound | Upper Bound |
| 2020 | 1 | .2214 | .01064 | .2015 | .2433 |
|  | 2 | .1411 | .01011 | .1226 | .1624 |
| 2021 | 1 | .2856 | .00659 | .2729 | .2988 |
|  | 2 | .1672 | .00543 | .1569 | .1782 |
| 2022 | 1 | .1895 | .00323 | .1833 | .1959 |
|  | 2 | .1360 | .00307 | .1301 | .1422 |
| 2023 | 1 | .1750 | .00173 | .1716 | .1784 |
|  | 2 | .1257 | .00180 | .1222 | .1292 |
| 2024 | 1 | .2174 | .00222 | .2131 | .2218 |
|  | 2 | .1621 | .00227 | .1577 | .1667 |
| 2025 | 1 | .2101 | .00458 | .2013 | .2192 |
|  | 2 | .1810 | .00594 | .1698 | .1931 |

| **Pairwise Comparisons** | | | | | | | | |
| --- | --- | --- | --- | --- | --- | --- | --- | --- |
| Diagnostic | (I) Year | (J) Year | Mean Difference (I-J) | Standard Error | Degrees of Freedom | P | 95% Wald Confidence Interval | |
|  |  |  |  |  |  |  | Lower Bound | Upper Bound |
| 1 | 2020 | 2021 | -.0642 | .01251 | 1 | .000 | -.0887 | -.0396 |
|  |  | 2022 | .0319 | .01111 | 1 | .004 | .0102 | .0537 |
|  |  | 2023 | .0464 | .01078 | 1 | .000 | .0253 | .0676 |
|  |  | 2024 | .0040 | .01087 | 1 | .711 | -.0173 | .0253 |
|  |  | 2025 | .0114 | .01156 | 1 | .326 | -.0113 | .0340 |
|  | 2021 | 2020 | .0642 | .01251 | 1 | .000 | .0396 | .0887 |
|  |  | 2022 | .0961 | .00732 | 1 | .000 | .0818 | .1105 |
|  |  | 2023 | .1106 | .00680 | 1 | .000 | .0973 | .1239 |
|  |  | 2024 | .0682 | .00693 | 1 | .000 | .0546 | .0818 |
|  |  | 2025 | .0755 | .00801 | 1 | .000 | .0598 | .0912 |
|  | 2022 | 2020 | -.0319 | .01111 | 1 | .004 | -.0537 | -.0102 |
|  |  | 2021 | -.0961 | .00732 | 1 | .000 | -.1105 | -.0818 |
|  |  | 2023 | .0145 | .00363 | 1 | .000 | .0074 | .0216 |
|  |  | 2024 | -.0279 | .00387 | 1 | .000 | -.0355 | -.0203 |
|  |  | 2025 | -.0206 | .00558 | 1 | .000 | -.0315 | -.0097 |
|  | 2023 | 2020 | -.0464 | .01078 | 1 | .000 | -.0676 | -.0253 |
|  |  | 2021 | -.1106 | .00680 | 1 | .000 | -.1239 | -.0973 |
|  |  | 2022 | -.0145 | .00363 | 1 | .000 | -.0216 | -.0074 |
|  |  | 2024 | -.0424 | .00274 | 1 | .000 | -.0478 | -.0370 |
|  |  | 2025 | -.0351 | .00489 | 1 | .000 | -.0447 | -.0255 |
|  | 2024 | 2020 | -.0040 | .01087 | 1 | .711 | -.0253 | .0173 |
|  |  | 2021 | -.0682 | .00693 | 1 | .000 | -.0818 | -.0546 |
|  |  | 2022 | .0279 | .00387 | 1 | .000 | .0203 | .0355 |
|  |  | 2023 | .0424 | .00274 | 1 | .000 | .0370 | .0478 |
|  |  | 2025 | .0073 | .00508 | 1 | .149 | -.0026 | .0173 |
|  | 2025 | 2020 | -.0114 | .01156 | 1 | .326 | -.0340 | .0113 |
|  |  | 2021 | -.0755 | .00801 | 1 | .000 | -.0912 | -.0598 |
|  |  | 2022 | .0206 | .00558 | 1 | .000 | .0097 | .0315 |
|  |  | 2023 | .0351 | .00489 | 1 | .000 | .0255 | .0447 |
|  |  | 2024 | -.0073 | .00508 | 1 | .149 | -.0173 | .0026 |
| 2 | 2020 | 2021 | -.0261 | .01130 | 1 | .021 | -.0483 | -.0040 |
|  |  | 2022 | .0051 | .01042 | 1 | .626 | -.0153 | .0255 |
|  |  | 2023 | .0154 | .01013 | 1 | .128 | -.0044 | .0353 |
|  |  | 2024 | -.0210 | .01020 | 1 | .039 | -.0410 | -.0011 |
|  |  | 2025 | -.0399 | .01149 | 1 | .001 | -.0625 | -.0174 |
|  | 2021 | 2020 | .0261 | .01130 | 1 | .021 | .0040 | .0483 |
|  |  | 2022 | .0312 | .00594 | 1 | .000 | .0196 | .0429 |
|  |  | 2023 | .0416 | .00544 | 1 | .000 | .0309 | .0522 |
|  |  | 2024 | .0051 | .00555 | 1 | .358 | -.0058 | .0160 |
|  |  | 2025 | -.0138 | .00766 | 1 | .072 | -.0288 | .0012 |
|  | 2022 | 2020 | -.0051 | .01042 | 1 | .626 | -.0255 | .0153 |
|  |  | 2021 | -.0312 | .00594 | 1 | .000 | -.0429 | -.0196 |
|  |  | 2023 | .0103 | .00318 | 1 | .001 | .0041 | .0166 |
|  |  | 2024 | -.0261 | .00339 | 1 | .000 | -.0328 | -.0195 |
|  |  | 2025 | -.0450 | .00632 | 1 | .000 | -.0574 | -.0326 |
|  | 2023 | 2020 | -.0154 | .01013 | 1 | .128 | -.0353 | .0044 |
|  |  | 2021 | -.0416 | .00544 | 1 | .000 | -.0522 | -.0309 |
|  |  | 2022 | -.0103 | .00318 | 1 | .001 | -.0166 | -.0041 |
|  |  | 2024 | -.0365 | .00236 | 1 | .000 | -.0411 | -.0319 |
|  |  | 2025 | -.0554 | .00586 | 1 | .000 | -.0669 | -.0439 |
|  | 2024 | 2020 | .0210 | .01020 | 1 | .039 | .0011 | .0410 |
|  |  | 2021 | -.0051 | .00555 | 1 | .358 | -.0160 | .0058 |
|  |  | 2022 | .0261 | .00339 | 1 | .000 | .0195 | .0328 |
|  |  | 2023 | .0365 | .00236 | 1 | .000 | .0319 | .0411 |
|  |  | 2025 | -.0189 | .00595 | 1 | .001 | -.0306 | -.0072 |
|  | 2025 | 2020 | .0399 | .01149 | 1 | .001 | .0174 | .0625 |
|  |  | 2021 | .0138 | .00766 | 1 | .072 | -.0012 | .0288 |
|  |  | 2022 | .0450 | .00632 | 1 | .000 | .0326 | .0574 |
|  |  | 2023 | .0554 | .00586 | 1 | .000 | .0439 | .0669 |
|  |  | 2024 | .0189 | .00595 | 1 | .001 | .0072 | .0306 |

| **Overall Test** | | | |
| --- | --- | --- | --- |
| Diagnostic | Wald χ² | Degrees of Freedom | P |
| 1 | 456.235 | 5 | .000 |
| 2 | 315.134 | 5 | .000 |

**Estimated Marginal Means 10：Year* Diagnostic**

| **Estimate** | | | | | |
| --- | --- | --- | --- | --- | --- |
| Year | Diagnostic | Mean | Standard Error | 95% Wald Confidence Interval | |
|  |  |  |  | Lower Bound | Upper Bound |
| 2020 | 1 | .2214 | .01064 | .2015 | .2433 |
|  | 2 | .1411 | .01011 | .1226 | .1624 |
| 2021 | 1 | .2856 | .00659 | .2729 | .2988 |
|  | 2 | .1672 | .00543 | .1569 | .1782 |
| 2022 | 1 | .1895 | .00323 | .1833 | .1959 |
|  | 2 | .1360 | .00307 | .1301 | .1422 |
| 2023 | 1 | .1750 | .00173 | .1716 | .1784 |
|  | 2 | .1257 | .00180 | .1222 | .1292 |
| 2024 | 1 | .2174 | .00222 | .2131 | .2218 |
|  | 2 | .1621 | .00227 | .1577 | .1667 |
| 2025 | 1 | .2101 | .00458 | .2013 | .2192 |
|  | 2 | .1810 | .00594 | .1698 | .1931 |

| **Pairwise Comparisons** | | | | | | | | |
| --- | --- | --- | --- | --- | --- | --- | --- | --- |
| Year | (I) Diagnostic | (J) Diagnostic | Mean Difference (I-J) | Standard Error | Degrees of Freedom | P | 95% Wald Confidence Interval | |
|  |  |  |  |  |  |  | Lower Bound | Upper Bound |
| 2020 | 1 | 2 | .0803 | .01275 | 1 | .000 | .0553 | .1053 |
|  | 2 | 1 | -.0803 | .01275 | 1 | .000 | -.1053 | -.0553 |
| 2021 | 1 | 2 | .1183 | .00756 | 1 | .000 | .1035 | .1332 |
|  | 2 | 1 | -.1183 | .00756 | 1 | .000 | -.1332 | -.1035 |
| 2022 | 1 | 2 | .0535 | .00381 | 1 | .000 | .0460 | .0609 |
|  | 2 | 1 | -.0535 | .00381 | 1 | .000 | -.0609 | -.0460 |
| 2023 | 1 | 2 | .0493 | .00219 | 1 | .000 | .0450 | .0536 |
|  | 2 | 1 | -.0493 | .00219 | 1 | .000 | -.0536 | -.0450 |
| 2024 | 1 | 2 | .0553 | .00275 | 1 | .000 | .0499 | .0607 |
|  | 2 | 1 | -.0553 | .00275 | 1 | .000 | -.0607 | -.0499 |
| 2025 | 1 | 2 | .0290 | .00650 | 1 | .000 | .0163 | .0418 |
|  | 2 | 1 | -.0290 | .00650 | 1 | .000 | -.0418 | -.0163 |

| **Overall Test** | | | |
| --- | --- | --- | --- |
| Year | Wald χ² | Degrees of Freedom | P |
| 2020 | 39.675 | 1 | .000 |
| 2021 | 244.996 | 1 | .000 |
| 2022 | 197.077 | 1 | .000 |
| 2023 | 507.570 | 1 | .000 |
| 2024 | 402.564 | 1 | .000 |
| 2025 | 19.961 | 1 | .000 |

**Estimated Marginal Means 11：Gender* Age**

| **Estimate** | | | | | |
| --- | --- | --- | --- | --- | --- |
| Gender | Age | Mean | Standard Error | 95% Wald Confidence Interval | |
|  |  |  |  | Lower Bound | Upper Bound |
| 1 | 1 | .2837 | .00763 | .2692 | .2991 |
|  | 2 | .1584 | .00170 | .1551 | .1618 |
|  | 3 | .1680 | .00365 | .1610 | .1754 |
|  | 4 | .1845 | .00491 | .1751 | .1944 |
| 2 | 1 | .2790 | .00823 | .2633 | .2956 |
|  | 2 | .1363 | .00173 | .1329 | .1397 |
|  | 3 | .1382 | .00311 | .1323 | .1445 |
|  | 4 | .1484 | .00421 | .1404 | .1569 |

| **Pairwise Comparisons** | | | | | | | | |
| --- | --- | --- | --- | --- | --- | --- | --- | --- |
| Age | (I) Gender | (J) Gender | Mean Difference (I-J) | Standard Error | Degrees of Freedom | P | 95% Wald Confidence Interval | |
|  |  |  |  |  |  |  | Lower Bound | Upper Bound |
| 1 | 1 | 2 | .0048 | .00835 | 1 | .569 | -.0116 | .0211 |
|  | 2 | 1 | -.0048 | .00835 | 1 | .569 | -.0211 | .0116 |
| 2 | 1 | 2 | .0222 | .00214 | 1 | .000 | .0180 | .0263 |
|  | 2 | 1 | -.0222 | .00214 | 1 | .000 | -.0263 | -.0180 |
| 3 | 1 | 2 | .0298 | .00277 | 1 | .000 | .0244 | .0352 |
|  | 2 | 1 | -.0298 | .00277 | 1 | .000 | -.0352 | -.0244 |
| 4 | 1 | 2 | .0361 | .00343 | 1 | .000 | .0294 | .0428 |
|  | 2 | 1 | -.0361 | .00343 | 1 | .000 | -.0428 | -.0294 |

| **Overall Test** | | | |
| --- | --- | --- | --- |
| Age | Wald χ² | Degrees of Freedom | P |
| 1 | .324 | 1 | .569 |
| 2 | 107.296 | 1 | .000 |
| 3 | 115.547 | 1 | .000 |
| 4 | 110.821 | 1 | .000 |

**Estimated Marginal Means 12：Gender* Age**

| **Estimate** | | | | | |
| --- | --- | --- | --- | --- | --- |
| Gender | Age | Mean | Standard Error | 95% Wald Confidence Interval | |
|  |  |  |  | Lower Bound | Upper Bound |
| 1 | 1 | .2837 | .00763 | .2692 | .2991 |
|  | 2 | .1584 | .00170 | .1551 | .1618 |
|  | 3 | .1680 | .00365 | .1610 | .1754 |
|  | 4 | .1845 | .00491 | .1751 | .1944 |
| 2 | 1 | .2790 | .00823 | .2633 | .2956 |
|  | 2 | .1363 | .00173 | .1329 | .1397 |
|  | 3 | .1382 | .00311 | .1323 | .1445 |
|  | 4 | .1484 | .00421 | .1404 | .1569 |

| **Pairwise Comparisons** | | | | | | | | |
| --- | --- | --- | --- | --- | --- | --- | --- | --- |
| Gender | (I) Age | (J) Age | Mean Difference (I-J) | Standard Error | Degrees of Freedom | P | 95% Wald Confidence Interval | |
|  |  |  |  |  |  |  | Lower Bound | Upper Bound |
| 1 | 1 | 2 | .1253 | .00770 | 1 | .000 | .1102 | .1404 |
|  |  | 3 | .1157 | .00833 | 1 | .000 | .0994 | .1320 |
|  |  | 4 | .0992 | .00901 | 1 | .000 | .0816 | .1169 |
|  | 2 | 1 | -.1253 | .00770 | 1 | .000 | -.1404 | -.1102 |
|  |  | 3 | -.0096 | .00382 | 1 | .012 | -.0171 | -.0021 |
|  |  | 4 | -.0261 | .00508 | 1 | .000 | -.0360 | -.0161 |
|  | 3 | 1 | -.1157 | .00833 | 1 | .000 | -.1320 | -.0994 |
|  |  | 2 | .0096 | .00382 | 1 | .012 | .0021 | .0171 |
|  |  | 4 | -.0165 | .00597 | 1 | .006 | -.0282 | -.0047 |
|  | 4 | 1 | -.0992 | .00901 | 1 | .000 | -.1169 | -.0816 |
|  |  | 2 | .0261 | .00508 | 1 | .000 | .0161 | .0360 |
|  |  | 3 | .0165 | .00597 | 1 | .006 | .0047 | .0282 |
| 2 | 1 | 2 | .1427 | .00823 | 1 | .000 | .1266 | .1588 |
|  |  | 3 | .1408 | .00863 | 1 | .000 | .1238 | .1577 |
|  |  | 4 | .1306 | .00910 | 1 | .000 | .1128 | .1484 |
|  | 2 | 1 | -.1427 | .00823 | 1 | .000 | -.1588 | -.1266 |
|  |  | 3 | -.0020 | .00326 | 1 | .548 | -.0083 | .0044 |
|  |  | 4 | -.0121 | .00428 | 1 | .005 | -.0205 | -.0037 |
|  | 3 | 1 | -.1408 | .00863 | 1 | .000 | -.1577 | -.1238 |
|  |  | 2 | .0020 | .00326 | 1 | .548 | -.0044 | .0083 |
|  |  | 4 | -.0102 | .00499 | 1 | .042 | -.0199 | -.0004 |
|  | 4 | 1 | -.1306 | .00910 | 1 | .000 | -.1484 | -.1128 |
|  |  | 2 | .0121 | .00428 | 1 | .005 | .0037 | .0205 |
|  |  | 3 | .0102 | .00499 | 1 | .042 | .0004 | .0199 |

| **Overall Test** | | | |
| --- | --- | --- | --- |
| Gender | Wald χ² | Degrees of Freedom | P |
| 1 | 284.168 | 3 | .000 |
| 2 | 305.493 | 3 | .000 |

**Estimated Marginal Means 13：Gender* Diagnostic**

| **Estimate** | | | | | |
| --- | --- | --- | --- | --- | --- |
| Gender | Diagnostic | Mean | Standard Error | 95% Wald Confidence Interval | |
|  |  |  |  | Lower Bound | Upper Bound |
| 1 | 1 | .2316 | .00275 | .2263 | .2371 |
|  | 2 | .1612 | .00295 | .1555 | .1671 |
| 2 | 1 | .1975 | .00261 | .1925 | .2027 |
|  | 2 | .1414 | .00282 | .1360 | .1470 |

| **Pairwise Comparisons** | | | | | | | | |
| --- | --- | --- | --- | --- | --- | --- | --- | --- |
| Diagnostic | (I) Gender | (J) Gender | Mean Difference (I-J) | Standard Error | Degrees of Freedom | P | 95% Wald Confidence Interval | |
|  |  |  |  |  |  |  | Lower Bound | Upper Bound |
| 1 | 1 | 2 | .0341 | .00301 | 1 | .000 | .0282 | .0400 |
|  | 2 | 1 | -.0341 | .00301 | 1 | .000 | -.0400 | -.0282 |
| 2 | 1 | 2 | .0198 | .00251 | 1 | .000 | .0149 | .0247 |
|  | 2 | 1 | -.0198 | .00251 | 1 | .000 | -.0247 | -.0149 |

| **Overall Test** | | | |
| --- | --- | --- | --- |
| Diagnostic | Wald χ² | Degrees of Freedom | P |
| 1 | 127.995 | 1 | .000 |
| 2 | 62.157 | 1 | .000 |

**Estimated Marginal Means 14：Gender* Diagnostic**

| **Estimate** | | | | | |
| --- | --- | --- | --- | --- | --- |
| Gender | Diagnostic | Mean | Standard Error | 95% Wald Confidence Interval | |
|  |  |  |  | Lower Bound | Upper Bound |
| 1 | 1 | .2316 | .00275 | .2263 | .2371 |
|  | 2 | .1612 | .00295 | .1555 | .1671 |
| 2 | 1 | .1975 | .00261 | .1925 | .2027 |
|  | 2 | .1414 | .00282 | .1360 | .1470 |

| **Pairwise Comparisons** | | | | | | | | |
| --- | --- | --- | --- | --- | --- | --- | --- | --- |
| Gender | (I) Diagnostic | (J) Diagnostic | Mean Difference (I-J) | Standard Error | Degrees of Freedom | P | 95% Wald Confidence Interval | |
|  |  |  |  |  |  |  | Lower Bound | Upper Bound |
| 1 | 1 | 2 | .0704 | .00355 | 1 | .000 | .0634 | .0774 |
|  | 2 | 1 | -.0704 | .00355 | 1 | .000 | -.0774 | -.0634 |
| 2 | 1 | 2 | .0561 | .00322 | 1 | .000 | .0498 | .0624 |
|  | 2 | 1 | -.0561 | .00322 | 1 | .000 | -.0624 | -.0498 |

| **Overall Test** | | | |
| --- | --- | --- | --- |
| Gender | Wald χ² | Degrees of Freedom | P |
| 1 | 392.411 | 1 | .000 |
| 2 | 304.468 | 1 | .000 |

**Estimated Marginal Means 15：Age* Diagnostic**

| **Estimate** | | | | | |
| --- | --- | --- | --- | --- | --- |
| Age | Diagnostic | Mean | Standard Error | 95% Wald Confidence Interval | |
|  |  |  |  | Lower Bound | Upper Bound |
| 1 | 1 | .2767 | .00476 | .2675 | .2861 |
|  | 2 | .2861 | .01259 | .2625 | .3119 |
| 2 | 1 | .1812 | .00187 | .1775 | .1849 |
|  | 2 | .1192 | .00168 | .1159 | .1225 |
| 3 | 1 | .1981 | .00414 | .1902 | .2064 |
|  | 2 | .1173 | .00281 | .1119 | .1229 |
| 4 | 1 | .2108 | .00569 | .1999 | .2222 |
|  | 2 | .1299 | .00371 | .1228 | .1374 |

| **Pairwise Comparisons** | | | | | | | | |
| --- | --- | --- | --- | --- | --- | --- | --- | --- |
| Diagnostic | (I) Age | (J) Age | Mean Difference (I-J) | Standard Error | Degrees of Freedom | P | 95% Wald Confidence Interval | |
|  |  |  |  |  |  |  | Lower Bound | Upper Bound |
| 1 | 1 | 2 | .0955 | .00510 | 1 | .000 | .0855 | .1055 |
|  |  | 3 | .0785 | .00630 | 1 | .000 | .0662 | .0909 |
|  |  | 4 | .0659 | .00741 | 1 | .000 | .0514 | .0804 |
|  | 2 | 1 | -.0955 | .00510 | 1 | .000 | -.1055 | -.0855 |
|  |  | 3 | -.0170 | .00441 | 1 | .000 | -.0256 | -.0083 |
|  |  | 4 | -.0296 | .00582 | 1 | .000 | -.0410 | -.0182 |
|  | 3 | 1 | -.0785 | .00630 | 1 | .000 | -.0909 | -.0662 |
|  |  | 2 | .0170 | .00441 | 1 | .000 | .0083 | .0256 |
|  |  | 4 | -.0126 | .00687 | 1 | .066 | -.0261 | .0008 |
|  | 4 | 1 | -.0659 | .00741 | 1 | .000 | -.0804 | -.0514 |
|  |  | 2 | .0296 | .00582 | 1 | .000 | .0182 | .0410 |
|  |  | 3 | .0126 | .00687 | 1 | .066 | -.0008 | .0261 |
| 2 | 1 | 2 | .1670 | .01240 | 1 | .000 | .1427 | .1913 |
|  |  | 3 | .1689 | .01258 | 1 | .000 | .1442 | .1935 |
|  |  | 4 | .1562 | .01283 | 1 | .000 | .1311 | .1814 |
|  | 2 | 1 | -.1670 | .01240 | 1 | .000 | -.1913 | -.1427 |
|  |  | 3 | .0019 | .00284 | 1 | .497 | -.0036 | .0075 |
|  |  | 4 | -.0107 | .00375 | 1 | .004 | -.0181 | -.0034 |
|  | 3 | 1 | -.1689 | .01258 | 1 | .000 | -.1935 | -.1442 |
|  |  | 2 | -.0019 | .00284 | 1 | .497 | -.0075 | .0036 |
|  |  | 4 | -.0126 | .00435 | 1 | .004 | -.0212 | -.0041 |
|  | 4 | 1 | -.1562 | .01283 | 1 | .000 | -.1814 | -.1311 |
|  |  | 2 | .0107 | .00375 | 1 | .004 | .0034 | .0181 |
|  |  | 3 | .0126 | .00435 | 1 | .004 | .0041 | .0212 |

| **Overall Test** | | | |
| --- | --- | --- | --- |
| Diagnostic | Wald χ² | Degrees of Freedom | P |
| 1 | 363.931 | 3 | .000 |
| 2 | 190.157 | 3 | .000 |

**Estimated Marginal Means 16：Age* Diagnostic**

| **Estimate** | | | | | |
| --- | --- | --- | --- | --- | --- |
| Age | Diagnostic | Mean | Standard Error | 95% Wald Confidence Interval | |
|  |  |  |  | Lower Bound | Upper Bound |
| 1 | 1 | .2767 | .00476 | .2675 | .2861 |
|  | 2 | .2861 | .01259 | .2625 | .3119 |
| 2 | 1 | .1812 | .00187 | .1775 | .1849 |
|  | 2 | .1192 | .00168 | .1159 | .1225 |
| 3 | 1 | .1981 | .00414 | .1902 | .2064 |
|  | 2 | .1173 | .00281 | .1119 | .1229 |
| 4 | 1 | .2108 | .00569 | .1999 | .2222 |
|  | 2 | .1299 | .00371 | .1228 | .1374 |

| **Pairwise Comparisons** | | | | | | | | |
| --- | --- | --- | --- | --- | --- | --- | --- | --- |
| Age | (I) Diagnostic | (J) Diagnostic | Mean Difference (I-J) | Standard Error | Degrees of Freedom | P | 95% Wald Confidence Interval | |
|  |  |  |  |  |  |  | Lower Bound | Upper Bound |
| 1 | 1 | 2 | -.0095 | .01324 | 1 | .474 | -.0354 | .0165 |
|  | 2 | 1 | .0095 | .01324 | 1 | .474 | -.0165 | .0354 |
| 2 | 1 | 2 | .0620 | .00237 | 1 | .000 | .0573 | .0666 |
|  | 2 | 1 | -.0620 | .00237 | 1 | .000 | -.0666 | -.0573 |
| 3 | 1 | 2 | .0809 | .00325 | 1 | .000 | .0745 | .0872 |
|  | 2 | 1 | -.0809 | .00325 | 1 | .000 | -.0872 | -.0745 |
| 4 | 1 | 2 | .0809 | .00413 | 1 | .000 | .0728 | .0890 |
|  | 2 | 1 | -.0809 | .00413 | 1 | .000 | -.0890 | -.0728 |

| **Overall Test** | | | |
| --- | --- | --- | --- |
| Age | Wald χ² | Degrees of Freedom | P |
| 1 | .513 | 1 | .474 |
| 2 | 686.247 | 1 | .000 |
| 3 | 620.971 | 1 | .000 |
| 4 | 384.013 | 1 | .000 |
