# Supplementary material for: The impact of COVID-19 social isolation and reduced microbial exposure on the immune system in children: a retrospective study
Source: PeerJ. 2026 Jul 7;14:e21469. doi: 10.7717/peerj.21469 (PMC13353229; doi:10.7717/peerj.21469)
Supplement: Supplemental Information 8 [file peerj-14-21469-s008.docx]

**Basophils Generalized Linear Model**

For CBC analysis, patients were categorized into the following age groups:
Group 1: 0–3 months,
Group 2: 3 months–4 years,
Group 3: 4–6 years,
Group 4: 6–13 years.

Diagnostic 1 is LRTIs . Diagnostic 2 URTIs .

| **Parameter Estimate** | | | | | | | |
| --- | --- | --- | --- | --- | --- | --- | --- |
| Parameter | B | Standard Error | 95% Wald Confidence Interval | | Hypothesis Testing | | |
|  |  |  | Lower Bound | Upper Bound | Wald χ² | Degrees of Freedom | P |
| （Intercept） | -3.639 | .0417 | -3.721 | -3.558 | 7629.978 | 1 | .000 |
| [Year=2020] | -.283 | .0983 | -.476 | -.091 | 8.314 | 1 | .004 |
| [Year=2021] | -.165 | .0558 | -.274 | -.056 | 8.775 | 1 | .003 |
| [Year=2022] | -.273 | .0485 | -.368 | -.178 | 31.677 | 1 | .000 |
| [Year=2023] | -.426 | .0418 | -.508 | -.344 | 103.922 | 1 | .000 |
| [Year=2024] | -.394 | .0419 | -.476 | -.311 | 88.297 | 1 | .000 |
| [Year=2025] | 0 | . | . | . | . | . | . |
| [Male ] | .049 | .0239 | .002 | .096 | 4.213 | 1 | .040 |
| [Female ] | 0 | . | . | . | . | . | . |
| [Age=1] | .120 | .0566 | .009 | .230 | 4.466 | 1 | .035 |
| [Age=2] | .015 | .0411 | -.066 | .095 | .129 | 1 | .719 |
| [Age=3] | -.036 | .0483 | -.131 | .059 | .551 | 1 | .458 |
| [Age=4] | 0 | . | . | . | . | . | . |
| [LRTIs ] | -.106 | .0263 | -.157 | -.054 | 16.145 | 1 | .000 |
| [URTIs ] | 0 | . | . | . | . | . | . |
| [Year=2020] * [Male ] | .138 | .0470 | .046 | .230 | 8.630 | 1 | .003 |
| [Year=2020] * [Female ] | 0 | . | . | . | . | . | . |
| [Year=2021] * [Male ] | .126 | .0300 | .067 | .184 | 17.549 | 1 | .000 |
| [Year=2021] * [Female ] | 0 | . | . | . | . | . | . |
| [Year=2022] * [Male ] | .093 | .0247 | .044 | .141 | 14.080 | 1 | .000 |
| [Year=2022] * [Female ] | 0 | . | . | . | . | . | . |
| [Year=2023] * [Male ] | .130 | .0214 | .088 | .172 | 36.678 | 1 | .000 |
| [Year=2023] * [Female ] | 0 | . | . | . | . | . | . |
| [Year=2024] * [Male ] | .098 | .0212 | .056 | .139 | 21.114 | 1 | .000 |
| [Year=2024] * [Female ] | 0 | . | . | . | . | . | . |
| [Year=2025] * [Male ] | 0 | . | . | . | . | . | . |
| [Year=2025] * [Female ] | 0 | . | . | . | . | . | . |
| [Year=2020] * [Age=1] | .039 | .1016 | -.160 | .238 | .148 | 1 | .701 |
| [Year=2020] * [Age=2] | -.134 | .0959 | -.322 | .054 | 1.943 | 1 | .163 |
| [Year=2020] * [Age=3] | .085 | .1198 | -.150 | .320 | .503 | 1 | .478 |
| [Year=2020] * [Age=4] | 0 | . | . | . | . | . | . |
| [Year=2021] * [Age=1] | -.369 | .0656 | -.497 | -.240 | 31.607 | 1 | .000 |
| [Year=2021] * [Age=2] | -.206 | .0536 | -.311 | -.101 | 14.802 | 1 | .000 |
| [Year=2021] * [Age=3] | -.054 | .0617 | -.175 | .067 | .771 | 1 | .380 |
| [Year=2021] * [Age=4] | 0 | . | . | . | . | . | . |
| [Year=2022] * [Age=1] | -.192 | .0584 | -.307 | -.078 | 10.846 | 1 | .001 |
| [Year=2022] * [Age=2] | -.143 | .0475 | -.236 | -.050 | 9.085 | 1 | .003 |
| [Year=2022] * [Age=3] | .004 | .0544 | -.102 | .111 | .006 | 1 | .936 |
| [Year=2022] * [Age=4] | 0 | . | . | . | . | . | . |
| [Year=2023] * [Age=1] | -.128 | .0497 | -.226 | -.031 | 6.642 | 1 | .010 |
| [Year=2023] * [Age=2] | -.091 | .0411 | -.172 | -.010 | 4.907 | 1 | .027 |
| [Year=2023] * [Age=3] | .034 | .0481 | -.060 | .129 | .514 | 1 | .474 |
| [Year=2023] * [Age=4] | 0 | . | . | . | . | . | . |
| [Year=2024] * [Age=1] | -.157 | .0506 | -.257 | -.058 | 9.666 | 1 | .002 |
| [Year=2024] * [Age=2] | -.104 | .0411 | -.185 | -.024 | 6.426 | 1 | .011 |
| [Year=2024] * [Age=3] | .055 | .0481 | -.039 | .149 | 1.308 | 1 | .253 |
| [Year=2024] * [Age=4] | 0 | . | . | . | . | . | . |
| [Year=2025] * [Age=1] | 0 | . | . | . | . | . | . |
| [Year=2025] * [Age=2] | 0 | . | . | . | . | . | . |
| [Year=2025] * [Age=3] | 0 | . | . | . | . | . | . |
| [Year=2025] * [Age=4] | 0 | . | . | . | . | . | . |
| [Year=2020] * [LRTIs ] | .099 | .0632 | -.025 | .223 | 2.451 | 1 | .117 |
| [Year=2020] * [URTIs ] | 0 | . | . | . | . | . | . |
| [Year=2021] * [LRTIs ] | .240 | .0330 | .175 | .305 | 52.860 | 1 | .000 |
| [Year=2021] * [URTIs ] | 0 | . | . | . | . | . | . |
| [Year=2022] * [LRTIs ] | .198 | .0276 | .143 | .252 | 51.163 | 1 | .000 |
| [Year=2022] * [URTIs ] | 0 | . | . | . | . | . | . |
| [Year=2023] * [LRTIs ] | .173 | .0244 | .125 | .221 | 50.205 | 1 | .000 |
| [Year=2023] * [URTIs ] | 0 | . | . | . | . | . | . |
| [Year=2024] * [LRTIs ] | .174 | .0243 | .127 | .222 | 51.503 | 1 | .000 |
| [Year=2024] * [URTIs ] | 0 | . | . | . | . | . | . |
| [Year=2025] * [LRTIs ] | 0 | . | . | . | . | . | . |
| [Year=2025] * [URTIs ] | 0 | . | . | . | . | . | . |
| [Male ] * [Age=1] | -.140 | .0251 | -.189 | -.091 | 31.071 | 1 | .000 |
| [Male ] * [Age=2] | -.068 | .0133 | -.094 | -.042 | 26.495 | 1 | .000 |
| [Male ] * [Age=3] | -.039 | .0141 | -.066 | -.011 | 7.637 | 1 | .006 |
| [Male ] * [Age=4] | 0 | . | . | . | . | . | . |
| [Female ] * [Age=1] | 0 | . | . | . | . | . | . |
| [Female ] * [Age=2] | 0 | . | . | . | . | . | . |
| [Female ] * [Age=3] | 0 | . | . | . | . | . | . |
| [Female ] * [Age=4] | 0 | . | . | . | . | . | . |
| [Male ] * [LRTIs ] | -.004 | .0101 | -.024 | .016 | .141 | 1 | .707 |
| [Male ] * [URTIs ] | 0 | . | . | . | . | . | . |
| [Female ] * [LRTIs ] | 0 | . | . | . | . | . | . |
| [Female ] * [URTIs ] | 0 | . | . | . | . | . | . |
| [Age=1] * [LRTIs ] | .285 | .0368 | .213 | .357 | 59.970 | 1 | .000 |
| [Age=1] * [URTIs ] | 0 | . | . | . | . | . | . |
| [Age=2] * [LRTIs ] | .016 | .0134 | -.011 | .042 | 1.374 | 1 | .241 |
| [Age=2] * [URTIs ] | 0 | . | . | . | . | . | . |
| [Age=3] * [LRTIs ] | -.020 | .0144 | -.048 | .008 | 2.005 | 1 | .157 |
| [Age=3] * [URTIs ] | 0 | . | . | . | . | . | . |
| [Age=4] * [LRTIs ] | 0 | . | . | . | . | . | . |
| [Age=4] * [URTIs ] | 0 | . | . | . | . | . | . |
| （标度） | .371 | .0020 | .367 | .375 |  |  |  |

**Estimated Marginal Means 1：Year**

| **Estimate** | | | | |
| --- | --- | --- | --- | --- |
| Year | Mean | Standard Error | 95% Wald Confidence Interval | |
|  |  |  | Lower Bound | Upper Bound |
| 2020 | .0222 | .00074 | .0208 | .0237 |
| 2021 | .0228 | .00035 | .0222 | .0235 |
| 2022 | .0213 | .00024 | .0208 | .0217 |
| 2023 | .0190 | .00014 | .0188 | .0193 |
| 2024 | .0193 | .00014 | .0190 | .0196 |
| 2025 | .0263 | .00037 | .0255 | .0270 |

| **Pairwise Comparisons** | | | | | | | |
| --- | --- | --- | --- | --- | --- | --- | --- |
| (I) Year | (J) Year | Mean Difference (I-J) | Standard Error | Degrees of Freedom | P | 95% Wald Confidence Interval | |
|  |  |  |  |  |  | Lower Bound | Upper Bound |
| 2020 | 2021 | -.0006 | .00081 | 1 | .437 | -.0022 | .0010 |
|  | 2022 | .0009 | .00077 | 1 | .218 | -.0006 | .0025 |
|  | 2023 | .0032 | .00074 | 1 | .000 | .0017 | .0046 |
|  | 2024 | .0029 | .00075 | 1 | .000 | .0015 | .0044 |
|  | 2025 | -.0040 | .00081 | 1 | .000 | -.0056 | -.0025 |
| 2021 | 2020 | .0006 | .00081 | 1 | .437 | -.0010 | .0022 |
|  | 2022 | .0016 | .00041 | 1 | .000 | .0008 | .0024 |
|  | 2023 | .0038 | .00037 | 1 | .000 | .0031 | .0045 |
|  | 2024 | .0036 | .00037 | 1 | .000 | .0028 | .0043 |
|  | 2025 | -.0034 | .00049 | 1 | .000 | -.0044 | -.0024 |
| 2022 | 2020 | -.0009 | .00077 | 1 | .218 | -.0025 | .0006 |
|  | 2021 | -.0016 | .00041 | 1 | .000 | -.0024 | -.0008 |
|  | 2023 | .0022 | .00027 | 1 | .000 | .0017 | .0027 |
|  | 2024 | .0020 | .00027 | 1 | .000 | .0015 | .0025 |
|  | 2025 | -.0050 | .00043 | 1 | .000 | -.0058 | -.0041 |
| 2023 | 2020 | -.0032 | .00074 | 1 | .000 | -.0046 | -.0017 |
|  | 2021 | -.0038 | .00037 | 1 | .000 | -.0045 | -.0031 |
|  | 2022 | -.0022 | .00027 | 1 | .000 | -.0027 | -.0017 |
|  | 2024 | -.0002 | .00019 | 1 | .224 | -.0006 | .0001 |
|  | 2025 | -.0072 | .00038 | 1 | .000 | -.0080 | -.0065 |
| 2024 | 2020 | -.0029 | .00075 | 1 | .000 | -.0044 | -.0015 |
|  | 2021 | -.0036 | .00037 | 1 | .000 | -.0043 | -.0028 |
|  | 2022 | -.0020 | .00027 | 1 | .000 | -.0025 | -.0015 |
|  | 2023 | .0002 | .00019 | 1 | .224 | -.0001 | .0006 |
|  | 2025 | -.0070 | .00039 | 1 | .000 | -.0077 | -.0062 |
| 2025 | 2020 | .0040 | .00081 | 1 | .000 | .0025 | .0056 |
|  | 2021 | .0034 | .00049 | 1 | .000 | .0024 | .0044 |
|  | 2022 | .0050 | .00043 | 1 | .000 | .0041 | .0058 |
|  | 2023 | .0072 | .00038 | 1 | .000 | .0065 | .0080 |
|  | 2024 | .0070 | .00039 | 1 | .000 | .0062 | .0077 |

| **Overall Test** | | |
| --- | --- | --- |
| Wald χ² | Degrees of Freedom | P |
| 486.598 | 5 | .000 |

**Estimated Marginal Means 2：Gender**

| **Estimate** | | | | |
| --- | --- | --- | --- | --- |
| Gender | Mean | Standard Error | 95% Wald Confidence Interval | |
|  |  |  | Lower Bound | Upper Bound |
| 1 | .0226 | .00019 | .0222 | .0230 |
| 2 | .0208 | .00020 | .0204 | .0212 |

| **Pairwise Comparisons** | | | | | | | |
| --- | --- | --- | --- | --- | --- | --- | --- |
| (I) Gender | (J) Gender | Mean Difference (I-J) | Standard Error | Degrees of Freedom | P | 95% Wald Confidence Interval | |
|  |  |  |  |  |  | Lower Bound | Upper Bound |
| 1 | 2 | .0018 | .00021 | 1 | .000 | .0014 | .0022 |
| 2 | 1 | -.0018 | .00021 | 1 | .000 | -.0022 | -.0014 |

| **Overall Test** | | |
| --- | --- | --- |
| Wald χ² | Degrees of Freedom | P |
| 69.835 | 1 | .000 |

**Estimated Marginal Means 3：Age**

| **Estimate** | | | | |
| --- | --- | --- | --- | --- |
| Age | Mean | Standard Error | 95% Wald Confidence Interval | |
|  |  |  | Lower Bound | Upper Bound |
| 1 | .0236 | .00044 | .0228 | .0245 |
| 2 | .0197 | .00014 | .0194 | .0200 |
| 3 | .0213 | .00031 | .0207 | .0219 |
| 4 | .0223 | .00037 | .0216 | .0230 |

| **Pairwise Comparisons** | | | | | | | |
| --- | --- | --- | --- | --- | --- | --- | --- |
| (I) Age | (J) Age | Mean Difference (I-J) | Standard Error | Degrees of Freedom | P | 95% Wald Confidence Interval | |
|  |  |  |  |  |  | Lower Bound | Upper Bound |
| 1 | 2 | .0039 | .00045 | 1 | .000 | .0030 | .0048 |
|  | 3 | .0023 | .00052 | 1 | .000 | .0013 | .0033 |
|  | 4 | .0013 | .00057 | 1 | .021 | .0002 | .0024 |
| 2 | 1 | -.0039 | .00045 | 1 | .000 | -.0048 | -.0030 |
|  | 3 | -.0016 | .00033 | 1 | .000 | -.0023 | -.0010 |
|  | 4 | -.0026 | .00039 | 1 | .000 | -.0034 | -.0018 |
| 3 | 1 | -.0023 | .00052 | 1 | .000 | -.0033 | -.0013 |
|  | 2 | .0016 | .00033 | 1 | .000 | .0010 | .0023 |
|  | 4 | -.0010 | .00048 | 1 | .041 | -.0019 | .0000 |
| 4 | 1 | -.0013 | .00057 | 1 | .021 | -.0024 | -.0002 |
|  | 2 | .0026 | .00039 | 1 | .000 | .0018 | .0034 |
|  | 3 | .0010 | .00048 | 1 | .041 | .0000 | .0019 |

| **Overall Test** | | |
| --- | --- | --- |
| Wald χ² | Degrees of Freedom | P |
| 121.920 | 3 | .000 |

**Estimated Marginal Means 4：Diagnostic**

| **Estimate** | | | | |
| --- | --- | --- | --- | --- |
| Diagnostic | Mean | Standard Error | 95% Wald Confidence Interval | |
|  |  |  | Lower Bound | Upper Bound |
| 1 | .0229 | .00017 | .0226 | .0232 |
| 2 | .0205 | .00026 | .0200 | .0211 |

| **Pairwise Comparisons** | | | | | | | |
| --- | --- | --- | --- | --- | --- | --- | --- |
| (I) Diagnostic | (J) Diagnostic | Mean Difference (I-J) | Standard Error | Degrees of Freedom | P | 95% Wald Confidence Interval | |
|  |  |  |  |  |  | Lower Bound | Upper Bound |
| 1 | 2 | .0024 | .00030 | 1 | .000 | .0018 | .0030 |
| 2 | 1 | -.0024 | .00030 | 1 | .000 | -.0030 | -.0018 |

| **Overall Test** | | |
| --- | --- | --- |
| Wald χ² | Degrees of Freedom | P |
| 62.342 | 1 | .000 |

**Estimated Marginal Means 5：Year* Gender**

| **Estimate** | | | | | |
| --- | --- | --- | --- | --- | --- |
| Year | Gender | Mean | Standard Error | 95% Wald Confidence Interval | |
|  |  |  |  | Lower Bound | Upper Bound |
| 2020 | 1 | .0236 | .00087 | .0220 | .0254 |
|  | 2 | .0209 | .00089 | .0192 | .0227 |
| 2021 | 1 | .0241 | .00044 | .0233 | .0250 |
|  | 2 | .0216 | .00044 | .0208 | .0225 |
| 2022 | 1 | .0221 | .00029 | .0216 | .0227 |
|  | 2 | .0205 | .00030 | .0199 | .0210 |
| 2023 | 1 | .0202 | .00017 | .0198 | .0205 |
|  | 2 | .0180 | .00016 | .0177 | .0183 |
| 2024 | 1 | .0201 | .00017 | .0198 | .0204 |
|  | 2 | .0185 | .00017 | .0182 | .0188 |
| 2025 | 1 | .0261 | .00042 | .0253 | .0269 |
|  | 2 | .0265 | .00048 | .0255 | .0274 |

| **Pairwise Comparisons** | | | | | | | | |
| --- | --- | --- | --- | --- | --- | --- | --- | --- |
| Gender | (I) Year | (J) Year | Mean Difference (I-J) | Standard Error | Degrees of Freedom | P | 95% Wald Confidence Interval | |
|  |  |  |  |  |  |  | Lower Bound | Upper Bound |
| 1 | 2020 | 2021 | -.0005 | .00096 | 1 | .590 | -.0024 | .0014 |
|  |  | 2022 | .0015 | .00091 | 1 | .095 | -.0003 | .0033 |
|  |  | 2023 | .0035 | .00088 | 1 | .000 | .0017 | .0052 |
|  |  | 2024 | .0035 | .00088 | 1 | .000 | .0018 | .0053 |
|  |  | 2025 | -.0024 | .00095 | 1 | .010 | -.0043 | -.0006 |
|  | 2021 | 2020 | .0005 | .00096 | 1 | .590 | -.0014 | .0024 |
|  |  | 2022 | .0020 | .00051 | 1 | .000 | .0010 | .0030 |
|  |  | 2023 | .0040 | .00045 | 1 | .000 | .0031 | .0049 |
|  |  | 2024 | .0041 | .00045 | 1 | .000 | .0032 | .0049 |
|  |  | 2025 | -.0019 | .00059 | 1 | .001 | -.0031 | -.0008 |
|  | 2022 | 2020 | -.0015 | .00091 | 1 | .095 | -.0033 | .0003 |
|  |  | 2021 | -.0020 | .00051 | 1 | .000 | -.0030 | -.0010 |
|  |  | 2023 | .0019 | .00032 | 1 | .000 | .0013 | .0026 |
|  |  | 2024 | .0020 | .00032 | 1 | .000 | .0014 | .0026 |
|  |  | 2025 | -.0040 | .00049 | 1 | .000 | -.0049 | -.0030 |
|  | 2023 | 2020 | -.0035 | .00088 | 1 | .000 | -.0052 | -.0017 |
|  |  | 2021 | -.0040 | .00045 | 1 | .000 | -.0049 | -.0031 |
|  |  | 2022 | -.0019 | .00032 | 1 | .000 | -.0026 | -.0013 |
|  |  | 2024 | .0001 | .00022 | 1 | .689 | -.0003 | .0005 |
|  |  | 2025 | -.0059 | .00043 | 1 | .000 | -.0067 | -.0050 |
|  | 2024 | 2020 | -.0035 | .00088 | 1 | .000 | -.0053 | -.0018 |
|  |  | 2021 | -.0041 | .00045 | 1 | .000 | -.0049 | -.0032 |
|  |  | 2022 | -.0020 | .00032 | 1 | .000 | -.0026 | -.0014 |
|  |  | 2023 | -.0001 | .00022 | 1 | .689 | -.0005 | .0003 |
|  |  | 2025 | -.0060 | .00043 | 1 | .000 | -.0068 | -.0051 |
|  | 2025 | 2020 | .0024 | .00095 | 1 | .010 | .0006 | .0043 |
|  |  | 2021 | .0019 | .00059 | 1 | .001 | .0008 | .0031 |
|  |  | 2022 | .0040 | .00049 | 1 | .000 | .0030 | .0049 |
|  |  | 2023 | .0059 | .00043 | 1 | .000 | .0050 | .0067 |
|  |  | 2024 | .0060 | .00043 | 1 | .000 | .0051 | .0068 |
| 2 | 2020 | 2021 | -.0007 | .00098 | 1 | .462 | -.0026 | .0012 |
|  |  | 2022 | .0004 | .00093 | 1 | .643 | -.0014 | .0023 |
|  |  | 2023 | .0029 | .00090 | 1 | .001 | .0011 | .0047 |
|  |  | 2024 | .0024 | .00090 | 1 | .008 | .0006 | .0042 |
|  |  | 2025 | -.0056 | .00099 | 1 | .000 | -.0075 | -.0036 |
|  | 2021 | 2020 | .0007 | .00098 | 1 | .462 | -.0012 | .0026 |
|  |  | 2022 | .0012 | .00052 | 1 | .027 | .0001 | .0022 |
|  |  | 2023 | .0036 | .00046 | 1 | .000 | .0027 | .0045 |
|  |  | 2024 | .0031 | .00046 | 1 | .000 | .0022 | .0040 |
|  |  | 2025 | -.0048 | .00064 | 1 | .000 | -.0061 | -.0036 |
|  | 2022 | 2020 | -.0004 | .00093 | 1 | .643 | -.0023 | .0014 |
|  |  | 2021 | -.0012 | .00052 | 1 | .027 | -.0022 | -.0001 |
|  |  | 2023 | .0025 | .00032 | 1 | .000 | .0018 | .0031 |
|  |  | 2024 | .0020 | .00033 | 1 | .000 | .0013 | .0026 |
|  |  | 2025 | -.0060 | .00056 | 1 | .000 | -.0071 | -.0049 |
|  | 2023 | 2020 | -.0029 | .00090 | 1 | .001 | -.0047 | -.0011 |
|  |  | 2021 | -.0036 | .00046 | 1 | .000 | -.0045 | -.0027 |
|  |  | 2022 | -.0025 | .00032 | 1 | .000 | -.0031 | -.0018 |
|  |  | 2024 | -.0005 | .00021 | 1 | .016 | -.0009 | -.0001 |
|  |  | 2025 | -.0085 | .00050 | 1 | .000 | -.0094 | -.0075 |
|  | 2024 | 2020 | -.0024 | .00090 | 1 | .008 | -.0042 | -.0006 |
|  |  | 2021 | -.0031 | .00046 | 1 | .000 | -.0040 | -.0022 |
|  |  | 2022 | -.0020 | .00033 | 1 | .000 | -.0026 | -.0013 |
|  |  | 2023 | .0005 | .00021 | 1 | .016 | .0001 | .0009 |
|  |  | 2025 | -.0080 | .00050 | 1 | .000 | -.0089 | -.0070 |
|  | 2025 | 2020 | .0056 | .00099 | 1 | .000 | .0036 | .0075 |
|  |  | 2021 | .0048 | .00064 | 1 | .000 | .0036 | .0061 |
|  |  | 2022 | .0060 | .00056 | 1 | .000 | .0049 | .0071 |
|  |  | 2023 | .0085 | .00050 | 1 | .000 | .0075 | .0094 |
|  |  | 2024 | .0080 | .00050 | 1 | .000 | .0070 | .0089 |

| **Overall Test** | | | |
| --- | --- | --- | --- |
| Gender | Wald χ² | Degrees of Freedom | P |
| 1 | 294.856 | 5 | .000 |
| 2 | 361.680 | 5 | .000 |

**Estimated Marginal Means 6：Year* Gender**

| **Estimate** | | | | | |
| --- | --- | --- | --- | --- | --- |
| Year | Gender | Mean | Standard Error | 95% Wald Confidence Interval | |
|  |  |  |  | Lower Bound | Upper Bound |
| 2020 | 1 | .0236 | .00087 | .0220 | .0254 |
|  | 2 | .0209 | .00089 | .0192 | .0227 |
| 2021 | 1 | .0241 | .00044 | .0233 | .0250 |
|  | 2 | .0216 | .00044 | .0208 | .0225 |
| 2022 | 1 | .0221 | .00029 | .0216 | .0227 |
|  | 2 | .0205 | .00030 | .0199 | .0210 |
| 2023 | 1 | .0202 | .00017 | .0198 | .0205 |
|  | 2 | .0180 | .00016 | .0177 | .0183 |
| 2024 | 1 | .0201 | .00017 | .0198 | .0204 |
|  | 2 | .0185 | .00017 | .0182 | .0188 |
| 2025 | 1 | .0261 | .00042 | .0253 | .0269 |
|  | 2 | .0265 | .00048 | .0255 | .0274 |

| **Pairwise Comparisons** | | | | | | | | |
| --- | --- | --- | --- | --- | --- | --- | --- | --- |
| Year | (I) Gender | (J) Gender | Mean Difference (I-J) | Standard Error | Degrees of Freedom | P | 95% Wald Confidence Interval | |
|  |  |  |  |  |  |  | Lower Bound | Upper Bound |
| 2020 | 1 | 2 | .0027 | .00096 | 1 | .004 | .0009 | .0046 |
|  | 2 | 1 | -.0027 | .00096 | 1 | .004 | -.0046 | -.0009 |
| 2021 | 1 | 2 | .0025 | .00053 | 1 | .000 | .0015 | .0036 |
|  | 2 | 1 | -.0025 | .00053 | 1 | .000 | -.0036 | -.0015 |
| 2022 | 1 | 2 | .0017 | .00034 | 1 | .000 | .0010 | .0023 |
|  | 2 | 1 | -.0017 | .00034 | 1 | .000 | -.0023 | -.0010 |
| 2023 | 1 | 2 | .0022 | .00018 | 1 | .000 | .0018 | .0025 |
|  | 2 | 1 | -.0022 | .00018 | 1 | .000 | -.0025 | -.0018 |
| 2024 | 1 | 2 | .0016 | .00018 | 1 | .000 | .0012 | .0020 |
|  | 2 | 1 | -.0016 | .00018 | 1 | .000 | -.0020 | -.0012 |
| 2025 | 1 | 2 | -.0004 | .00050 | 1 | .444 | -.0014 | .0006 |
|  | 2 | 1 | .0004 | .00050 | 1 | .444 | -.0006 | .0014 |

| **Overall Test** | | | |
| --- | --- | --- | --- |
| Year | Wald χ² | Degrees of Freedom | P |
| 2020 | 8.224 | 1 | .004 |
| 2021 | 22.620 | 1 | .000 |
| 2022 | 23.756 | 1 | .000 |
| 2023 | 145.463 | 1 | .000 |
| 2024 | 78.070 | 1 | .000 |
| 2025 | .587 | 1 | .444 |

**Estimated Marginal Means 7：Year* Age**

| **Estimate** | | | | | |
| --- | --- | --- | --- | --- | --- |
| Year | Age | Mean | Standard Error | 95% Wald Confidence Interval | |
|  |  |  |  | Lower Bound | Upper Bound |
| 2020 | 1 | .0273 | .00129 | .0248 | .0299 |
|  | 2 | .0187 | .00063 | .0175 | .0200 |
|  | 3 | .0221 | .00166 | .0190 | .0256 |
|  | 4 | .0216 | .00174 | .0185 | .0253 |
| 2021 | 1 | .0218 | .00089 | .0201 | .0236 |
|  | 2 | .0209 | .00032 | .0203 | .0215 |
|  | 3 | .0230 | .00055 | .0220 | .0242 |
|  | 4 | .0260 | .00085 | .0244 | .0277 |
| 2022 | 1 | .0224 | .00074 | .0210 | .0239 |
|  | 2 | .0192 | .00020 | .0188 | .0196 |
|  | 3 | .0211 | .00030 | .0205 | .0217 |
|  | 4 | .0224 | .00054 | .0214 | .0235 |
| 2023 | 1 | .0207 | .00055 | .0196 | .0218 |
|  | 2 | .0175 | .00011 | .0173 | .0177 |
|  | 3 | .0188 | .00013 | .0185 | .0191 |
|  | 4 | .0194 | .00015 | .0191 | .0197 |
| 2024 | 1 | .0204 | .00055 | .0194 | .0215 |
|  | 2 | .0176 | .00010 | .0174 | .0178 |
|  | 3 | .0195 | .00013 | .0193 | .0198 |
|  | 4 | .0197 | .00017 | .0194 | .0200 |
| 2025 | 1 | .0309 | .00080 | .0294 | .0325 |
|  | 2 | .0252 | .00034 | .0246 | .0259 |
|  | 3 | .0239 | .00067 | .0226 | .0253 |
|  | 4 | .0255 | .00096 | .0237 | .0275 |

| **Pairwise Comparisons** | | | | | | | | |
| --- | --- | --- | --- | --- | --- | --- | --- | --- |
| Age | (I) Year | (J) Year | Mean Difference (I-J) | Standard Error | Degrees of Freedom | P | 95% Wald Confidence Interval | |
|  |  |  |  |  |  |  | Lower Bound | Upper Bound |
| 1 | 2020 | 2021 | .0055 | .00147 | 1 | .000 | .0026 | .0084 |
|  |  | 2022 | .0048 | .00141 | 1 | .001 | .0021 | .0076 |
|  |  | 2023 | .0066 | .00132 | 1 | .000 | .0040 | .0092 |
|  |  | 2024 | .0068 | .00133 | 1 | .000 | .0042 | .0095 |
|  |  | 2025 | -.0037 | .00136 | 1 | .007 | -.0063 | -.0010 |
|  | 2021 | 2020 | -.0055 | .00147 | 1 | .000 | -.0084 | -.0026 |
|  |  | 2022 | -.0007 | .00109 | 1 | .539 | -.0028 | .0015 |
|  |  | 2023 | .0011 | .00096 | 1 | .251 | -.0008 | .0030 |
|  |  | 2024 | .0014 | .00098 | 1 | .167 | -.0006 | .0033 |
|  |  | 2025 | -.0091 | .00105 | 1 | .000 | -.0112 | -.0071 |
|  | 2022 | 2020 | -.0048 | .00141 | 1 | .001 | -.0076 | -.0021 |
|  |  | 2021 | .0007 | .00109 | 1 | .539 | -.0015 | .0028 |
|  |  | 2023 | .0018 | .00085 | 1 | .037 | .0001 | .0034 |
|  |  | 2024 | .0020 | .00086 | 1 | .019 | .0003 | .0037 |
|  |  | 2025 | -.0085 | .00098 | 1 | .000 | -.0104 | -.0066 |
|  | 2023 | 2020 | -.0066 | .00132 | 1 | .000 | -.0092 | -.0040 |
|  |  | 2021 | -.0011 | .00096 | 1 | .251 | -.0030 | .0008 |
|  |  | 2022 | -.0018 | .00085 | 1 | .037 | -.0034 | -.0001 |
|  |  | 2024 | .0002 | .00070 | 1 | .724 | -.0011 | .0016 |
|  |  | 2025 | -.0103 | .00083 | 1 | .000 | -.0119 | -.0086 |
|  | 2024 | 2020 | -.0068 | .00133 | 1 | .000 | -.0095 | -.0042 |
|  |  | 2021 | -.0014 | .00098 | 1 | .167 | -.0033 | .0006 |
|  |  | 2022 | -.0020 | .00086 | 1 | .019 | -.0037 | -.0003 |
|  |  | 2023 | -.0002 | .00070 | 1 | .724 | -.0016 | .0011 |
|  |  | 2025 | -.0105 | .00086 | 1 | .000 | -.0122 | -.0088 |
|  | 2025 | 2020 | .0037 | .00136 | 1 | .007 | .0010 | .0063 |
|  |  | 2021 | .0091 | .00105 | 1 | .000 | .0071 | .0112 |
|  |  | 2022 | .0085 | .00098 | 1 | .000 | .0066 | .0104 |
|  |  | 2023 | .0103 | .00083 | 1 | .000 | .0086 | .0119 |
|  |  | 2024 | .0105 | .00086 | 1 | .000 | .0088 | .0122 |
| 2 | 2020 | 2021 | -.0022 | .00071 | 1 | .002 | -.0036 | -.0008 |
|  |  | 2022 | -.0005 | .00066 | 1 | .432 | -.0018 | .0008 |
|  |  | 2023 | .0012 | .00064 | 1 | .058 | .0000 | .0025 |
|  |  | 2024 | .0011 | .00064 | 1 | .073 | -.0001 | .0024 |
|  |  | 2025 | -.0065 | .00072 | 1 | .000 | -.0079 | -.0051 |
|  | 2021 | 2020 | .0022 | .00071 | 1 | .002 | .0008 | .0036 |
|  |  | 2022 | .0017 | .00038 | 1 | .000 | .0009 | .0024 |
|  |  | 2023 | .0034 | .00034 | 1 | .000 | .0027 | .0041 |
|  |  | 2024 | .0033 | .00034 | 1 | .000 | .0027 | .0040 |
|  |  | 2025 | -.0043 | .00047 | 1 | .000 | -.0052 | -.0034 |
|  | 2022 | 2020 | .0005 | .00066 | 1 | .432 | -.0008 | .0018 |
|  |  | 2021 | -.0017 | .00038 | 1 | .000 | -.0024 | -.0009 |
|  |  | 2023 | .0017 | .00023 | 1 | .000 | .0013 | .0022 |
|  |  | 2024 | .0017 | .00022 | 1 | .000 | .0012 | .0021 |
|  |  | 2025 | -.0060 | .00039 | 1 | .000 | -.0068 | -.0052 |
|  | 2023 | 2020 | -.0012 | .00064 | 1 | .058 | -.0025 | .0000 |
|  |  | 2021 | -.0034 | .00034 | 1 | .000 | -.0041 | -.0027 |
|  |  | 2022 | -.0017 | .00023 | 1 | .000 | -.0022 | -.0013 |
|  |  | 2024 | -.0001 | .00015 | 1 | .632 | -.0004 | .0002 |
|  |  | 2025 | -.0077 | .00035 | 1 | .000 | -.0084 | -.0070 |
|  | 2024 | 2020 | -.0011 | .00064 | 1 | .073 | -.0024 | .0001 |
|  |  | 2021 | -.0033 | .00034 | 1 | .000 | -.0040 | -.0027 |
|  |  | 2022 | -.0017 | .00022 | 1 | .000 | -.0021 | -.0012 |
|  |  | 2023 | .0001 | .00015 | 1 | .632 | -.0002 | .0004 |
|  |  | 2025 | -.0077 | .00035 | 1 | .000 | -.0083 | -.0070 |
|  | 2025 | 2020 | .0065 | .00072 | 1 | .000 | .0051 | .0079 |
|  |  | 2021 | .0043 | .00047 | 1 | .000 | .0034 | .0052 |
|  |  | 2022 | .0060 | .00039 | 1 | .000 | .0052 | .0068 |
|  |  | 2023 | .0077 | .00035 | 1 | .000 | .0070 | .0084 |
|  |  | 2024 | .0077 | .00035 | 1 | .000 | .0070 | .0083 |
| 3 | 2020 | 2021 | -.0010 | .00175 | 1 | .575 | -.0044 | .0024 |
|  |  | 2022 | .0009 | .00169 | 1 | .575 | -.0024 | .0043 |
|  |  | 2023 | .0033 | .00167 | 1 | .050 | .0000 | .0065 |
|  |  | 2024 | .0025 | .00167 | 1 | .126 | -.0007 | .0058 |
|  |  | 2025 | -.0018 | .00179 | 1 | .306 | -.0053 | .0017 |
|  | 2021 | 2020 | .0010 | .00175 | 1 | .575 | -.0024 | .0044 |
|  |  | 2022 | .0019 | .00063 | 1 | .002 | .0007 | .0032 |
|  |  | 2023 | .0043 | .00057 | 1 | .000 | .0031 | .0054 |
|  |  | 2024 | .0035 | .00057 | 1 | .000 | .0024 | .0046 |
|  |  | 2025 | -.0009 | .00087 | 1 | .326 | -.0026 | .0009 |
|  | 2022 | 2020 | -.0009 | .00169 | 1 | .575 | -.0043 | .0024 |
|  |  | 2021 | -.0019 | .00063 | 1 | .002 | -.0032 | -.0007 |
|  |  | 2023 | .0023 | .00033 | 1 | .000 | .0017 | .0030 |
|  |  | 2024 | .0016 | .00033 | 1 | .000 | .0010 | .0022 |
|  |  | 2025 | -.0028 | .00074 | 1 | .000 | -.0042 | -.0013 |
|  | 2023 | 2020 | -.0033 | .00167 | 1 | .050 | -.0065 | .0000 |
|  |  | 2021 | -.0043 | .00057 | 1 | .000 | -.0054 | -.0031 |
|  |  | 2022 | -.0023 | .00033 | 1 | .000 | -.0030 | -.0017 |
|  |  | 2024 | -.0007 | .00019 | 1 | .000 | -.0011 | -.0004 |
|  |  | 2025 | -.0051 | .00069 | 1 | .000 | -.0065 | -.0038 |
|  | 2024 | 2020 | -.0025 | .00167 | 1 | .126 | -.0058 | .0007 |
|  |  | 2021 | -.0035 | .00057 | 1 | .000 | -.0046 | -.0024 |
|  |  | 2022 | -.0016 | .00033 | 1 | .000 | -.0022 | -.0010 |
|  |  | 2023 | .0007 | .00019 | 1 | .000 | .0004 | .0011 |
|  |  | 2025 | -.0044 | .00069 | 1 | .000 | -.0057 | -.0030 |
|  | 2025 | 2020 | .0018 | .00179 | 1 | .306 | -.0017 | .0053 |
|  |  | 2021 | .0009 | .00087 | 1 | .326 | -.0009 | .0026 |
|  |  | 2022 | .0028 | .00074 | 1 | .000 | .0013 | .0042 |
|  |  | 2023 | .0051 | .00069 | 1 | .000 | .0038 | .0065 |
|  |  | 2024 | .0044 | .00069 | 1 | .000 | .0030 | .0057 |
| 4 | 2020 | 2021 | -.0043 | .00193 | 1 | .025 | -.0081 | -.0005 |
|  |  | 2022 | -.0008 | .00182 | 1 | .656 | -.0044 | .0028 |
|  |  | 2023 | .0023 | .00175 | 1 | .197 | -.0012 | .0057 |
|  |  | 2024 | .0019 | .00175 | 1 | .272 | -.0015 | .0053 |
|  |  | 2025 | -.0039 | .00198 | 1 | .050 | -.0078 | .0000 |
|  | 2021 | 2020 | .0043 | .00193 | 1 | .025 | .0005 | .0081 |
|  |  | 2022 | .0035 | .00100 | 1 | .000 | .0016 | .0055 |
|  |  | 2023 | .0066 | .00086 | 1 | .000 | .0049 | .0083 |
|  |  | 2024 | .0063 | .00086 | 1 | .000 | .0046 | .0079 |
|  |  | 2025 | .0005 | .00128 | 1 | .720 | -.0020 | .0030 |
|  | 2022 | 2020 | .0008 | .00182 | 1 | .656 | -.0028 | .0044 |
|  |  | 2021 | -.0035 | .00100 | 1 | .000 | -.0055 | -.0016 |
|  |  | 2023 | .0031 | .00056 | 1 | .000 | .0020 | .0042 |
|  |  | 2024 | .0027 | .00056 | 1 | .000 | .0016 | .0038 |
|  |  | 2025 | -.0031 | .00110 | 1 | .005 | -.0052 | -.0009 |
|  | 2023 | 2020 | -.0023 | .00175 | 1 | .197 | -.0057 | .0012 |
|  |  | 2021 | -.0066 | .00086 | 1 | .000 | -.0083 | -.0049 |
|  |  | 2022 | -.0031 | .00056 | 1 | .000 | -.0042 | -.0020 |
|  |  | 2024 | -.0003 | .00022 | 1 | .133 | -.0008 | .0001 |
|  |  | 2025 | -.0061 | .00097 | 1 | .000 | -.0080 | -.0042 |
|  | 2024 | 2020 | -.0019 | .00175 | 1 | .272 | -.0053 | .0015 |
|  |  | 2021 | -.0063 | .00086 | 1 | .000 | -.0079 | -.0046 |
|  |  | 2022 | -.0027 | .00056 | 1 | .000 | -.0038 | -.0016 |
|  |  | 2023 | .0003 | .00022 | 1 | .133 | -.0001 | .0008 |
|  |  | 2025 | -.0058 | .00097 | 1 | .000 | -.0077 | -.0039 |
|  | 2025 | 2020 | .0039 | .00198 | 1 | .050 | .0000 | .0078 |
|  |  | 2021 | -.0005 | .00128 | 1 | .720 | -.0030 | .0020 |
|  |  | 2022 | .0031 | .00110 | 1 | .005 | .0009 | .0052 |
|  |  | 2023 | .0061 | .00097 | 1 | .000 | .0042 | .0080 |
|  |  | 2024 | .0058 | .00097 | 1 | .000 | .0039 | .0077 |

| **Overall Test** | | | |
| --- | --- | --- | --- |
| Age | Wald χ² | Degrees of Freedom | P |
| 1 | 194.173 | 5 | .000 |
| 2 | 614.446 | 5 | .000 |
| 3 | 143.198 | 5 | .000 |
| 4 | 121.953 | 5 | .000 |

**Estimated Marginal Means 8：Year* Age**

| **Estimate** | | | | | |
| --- | --- | --- | --- | --- | --- |
| Year | Age | Mean | Standard Error | 95% Wald Confidence Interval | |
|  |  |  |  | Lower Bound | Upper Bound |
| 2020 | 1 | .0273 | .00129 | .0248 | .0299 |
|  | 2 | .0187 | .00063 | .0175 | .0200 |
|  | 3 | .0221 | .00166 | .0190 | .0256 |
|  | 4 | .0216 | .00174 | .0185 | .0253 |
| 2021 | 1 | .0218 | .00089 | .0201 | .0236 |
|  | 2 | .0209 | .00032 | .0203 | .0215 |
|  | 3 | .0230 | .00055 | .0220 | .0242 |
|  | 4 | .0260 | .00085 | .0244 | .0277 |
| 2022 | 1 | .0224 | .00074 | .0210 | .0239 |
|  | 2 | .0192 | .00020 | .0188 | .0196 |
|  | 3 | .0211 | .00030 | .0205 | .0217 |
|  | 4 | .0224 | .00054 | .0214 | .0235 |
| 2023 | 1 | .0207 | .00055 | .0196 | .0218 |
|  | 2 | .0175 | .00011 | .0173 | .0177 |
|  | 3 | .0188 | .00013 | .0185 | .0191 |
|  | 4 | .0194 | .00015 | .0191 | .0197 |
| 2024 | 1 | .0204 | .00055 | .0194 | .0215 |
|  | 2 | .0176 | .00010 | .0174 | .0178 |
|  | 3 | .0195 | .00013 | .0193 | .0198 |
|  | 4 | .0197 | .00017 | .0194 | .0200 |
| 2025 | 1 | .0309 | .00080 | .0294 | .0325 |
|  | 2 | .0252 | .00034 | .0246 | .0259 |
|  | 3 | .0239 | .00067 | .0226 | .0253 |
|  | 4 | .0255 | .00096 | .0237 | .0275 |

| **Pairwise Comparisons** | | | | | | | | |
| --- | --- | --- | --- | --- | --- | --- | --- | --- |
| Year | (I) Age | (J) Age | Mean Difference (I-J) | Standard Error | Degrees of Freedom | P | 95% Wald Confidence Interval | |
|  |  |  |  |  |  |  | Lower Bound | Upper Bound |
| 2020 | 1 | 2 | .0085 | .00132 | 1 | .000 | .0060 | .0111 |
|  |  | 3 | .0052 | .00194 | 1 | .007 | .0014 | .0090 |
|  |  | 4 | .0056 | .00217 | 1 | .010 | .0014 | .0099 |
|  | 2 | 1 | -.0085 | .00132 | 1 | .000 | -.0111 | -.0060 |
|  |  | 3 | -.0034 | .00173 | 1 | .053 | -.0067 | .0000 |
|  |  | 4 | -.0029 | .00185 | 1 | .114 | -.0066 | .0007 |
|  | 3 | 1 | -.0052 | .00194 | 1 | .007 | -.0090 | -.0014 |
|  |  | 2 | .0034 | .00173 | 1 | .053 | .0000 | .0067 |
|  |  | 4 | .0004 | .00241 | 1 | .860 | -.0043 | .0051 |
|  | 4 | 1 | -.0056 | .00217 | 1 | .010 | -.0099 | -.0014 |
|  |  | 2 | .0029 | .00185 | 1 | .114 | -.0007 | .0066 |
|  |  | 3 | -.0004 | .00241 | 1 | .860 | -.0051 | .0043 |
| 2021 | 1 | 2 | .0009 | .00094 | 1 | .352 | -.0010 | .0027 |
|  |  | 3 | -.0013 | .00103 | 1 | .216 | -.0033 | .0007 |
|  |  | 4 | -.0042 | .00121 | 1 | .001 | -.0066 | -.0018 |
|  | 2 | 1 | -.0009 | .00094 | 1 | .352 | -.0027 | .0010 |
|  |  | 3 | -.0022 | .00063 | 1 | .001 | -.0034 | -.0009 |
|  |  | 4 | -.0051 | .00090 | 1 | .000 | -.0068 | -.0033 |
|  | 3 | 1 | .0013 | .00103 | 1 | .216 | -.0007 | .0033 |
|  |  | 2 | .0022 | .00063 | 1 | .001 | .0009 | .0034 |
|  |  | 4 | -.0029 | .00100 | 1 | .003 | -.0049 | -.0010 |
|  | 4 | 1 | .0042 | .00121 | 1 | .001 | .0018 | .0066 |
|  |  | 2 | .0051 | .00090 | 1 | .000 | .0033 | .0068 |
|  |  | 3 | .0029 | .00100 | 1 | .003 | .0010 | .0049 |
| 2022 | 1 | 2 | .0032 | .00076 | 1 | .000 | .0017 | .0047 |
|  |  | 3 | .0013 | .00079 | 1 | .095 | -.0002 | .0029 |
|  |  | 4 | .0000 | .00091 | 1 | .994 | -.0018 | .0018 |
|  | 2 | 1 | -.0032 | .00076 | 1 | .000 | -.0047 | -.0017 |
|  |  | 3 | -.0019 | .00036 | 1 | .000 | -.0026 | -.0012 |
|  |  | 4 | -.0032 | .00057 | 1 | .000 | -.0043 | -.0021 |
|  | 3 | 1 | -.0013 | .00079 | 1 | .095 | -.0029 | .0002 |
|  |  | 2 | .0019 | .00036 | 1 | .000 | .0012 | .0026 |
|  |  | 4 | -.0013 | .00061 | 1 | .030 | -.0025 | -.0001 |
|  | 4 | 1 | .0000 | .00091 | 1 | .994 | -.0018 | .0018 |
|  |  | 2 | .0032 | .00057 | 1 | .000 | .0021 | .0043 |
|  |  | 3 | .0013 | .00061 | 1 | .030 | .0001 | .0025 |
| 2023 | 1 | 2 | .0032 | .00056 | 1 | .000 | .0021 | .0043 |
|  |  | 3 | .0019 | .00056 | 1 | .001 | .0008 | .0030 |
|  |  | 4 | .0013 | .00057 | 1 | .024 | .0002 | .0024 |
|  | 2 | 1 | -.0032 | .00056 | 1 | .000 | -.0043 | -.0021 |
|  |  | 3 | -.0013 | .00017 | 1 | .000 | -.0016 | -.0010 |
|  |  | 4 | -.0019 | .00019 | 1 | .000 | -.0023 | -.0015 |
|  | 3 | 1 | -.0019 | .00056 | 1 | .001 | -.0030 | -.0008 |
|  |  | 2 | .0013 | .00017 | 1 | .000 | .0010 | .0016 |
|  |  | 4 | -.0006 | .00020 | 1 | .004 | -.0010 | -.0002 |
|  | 4 | 1 | -.0013 | .00057 | 1 | .024 | -.0024 | -.0002 |
|  |  | 2 | .0019 | .00019 | 1 | .000 | .0015 | .0023 |
|  |  | 3 | .0006 | .00020 | 1 | .004 | .0002 | .0010 |
| 2024 | 1 | 2 | .0029 | .00055 | 1 | .000 | .0018 | .0039 |
|  |  | 3 | .0009 | .00056 | 1 | .108 | -.0002 | .0020 |
|  |  | 4 | .0007 | .00057 | 1 | .221 | -.0004 | .0018 |
|  | 2 | 1 | -.0029 | .00055 | 1 | .000 | -.0039 | -.0018 |
|  |  | 3 | -.0019 | .00017 | 1 | .000 | -.0023 | -.0016 |
|  |  | 4 | -.0022 | .00019 | 1 | .000 | -.0025 | -.0018 |
|  | 3 | 1 | -.0009 | .00056 | 1 | .108 | -.0020 | .0002 |
|  |  | 2 | .0019 | .00017 | 1 | .000 | .0016 | .0023 |
|  |  | 4 | -.0002 | .00021 | 1 | .334 | -.0006 | .0002 |
|  | 4 | 1 | -.0007 | .00057 | 1 | .221 | -.0018 | .0004 |
|  |  | 2 | .0022 | .00019 | 1 | .000 | .0018 | .0025 |
|  |  | 3 | .0002 | .00021 | 1 | .334 | -.0002 | .0006 |
| 2025 | 1 | 2 | .0057 | .00083 | 1 | .000 | .0041 | .0073 |
|  |  | 3 | .0070 | .00102 | 1 | .000 | .0050 | .0090 |
|  |  | 4 | .0054 | .00126 | 1 | .000 | .0029 | .0079 |
|  | 2 | 1 | -.0057 | .00083 | 1 | .000 | -.0073 | -.0041 |
|  |  | 3 | .0013 | .00074 | 1 | .072 | -.0001 | .0028 |
|  |  | 4 | -.0003 | .00102 | 1 | .774 | -.0023 | .0017 |
|  | 3 | 1 | -.0070 | .00102 | 1 | .000 | -.0090 | -.0050 |
|  |  | 2 | -.0013 | .00074 | 1 | .072 | -.0028 | .0001 |
|  |  | 4 | -.0016 | .00117 | 1 | .167 | -.0039 | .0007 |
|  | 4 | 1 | -.0054 | .00126 | 1 | .000 | -.0079 | -.0029 |
|  |  | 2 | .0003 | .00102 | 1 | .774 | -.0017 | .0023 |
|  |  | 3 | .0016 | .00117 | 1 | .167 | -.0007 | .0039 |

| **Overall Test** | | | |
| --- | --- | --- | --- |
| Year | Wald χ² | Degrees of Freedom | P |
| 2020 | 43.409 | 3 | .000 |
| 2021 | 37.542 | 3 | .000 |
| 2022 | 60.951 | 3 | .000 |
| 2023 | 132.606 | 3 | .000 |
| 2024 | 209.126 | 3 | .000 |
| 2025 | 55.752 | 3 | .000 |

**Estimated Marginal Means 9：Year* Diagnostic**

| **Estimate** | | | | | |
| --- | --- | --- | --- | --- | --- |
| Year | Diagnostic | Mean | Standard Error | 95% Wald Confidence Interval | |
|  |  |  |  | Lower Bound | Upper Bound |
| 2020 | 1 | .0229 | .00077 | .0215 | .0245 |
|  | 2 | .0215 | .00115 | .0194 | .0239 |
| 2021 | 1 | .0253 | .00041 | .0245 | .0261 |
|  | 2 | .0206 | .00048 | .0197 | .0216 |
| 2022 | 1 | .0230 | .00028 | .0225 | .0236 |
|  | 2 | .0196 | .00032 | .0190 | .0203 |
| 2023 | 1 | .0204 | .00015 | .0201 | .0207 |
|  | 2 | .0178 | .00020 | .0174 | .0182 |
| 2024 | 1 | .0206 | .00016 | .0203 | .0209 |
|  | 2 | .0180 | .00020 | .0176 | .0184 |
| 2025 | 1 | .0258 | .00038 | .0250 | .0265 |
|  | 2 | .0268 | .00058 | .0256 | .0279 |

| **Pairwise Comparisons** | | | | | | | | |
| --- | --- | --- | --- | --- | --- | --- | --- | --- |
| Diagnostic | (I) Year | (J) Year | Mean Difference (I-J) | Standard Error | Degrees of Freedom | P | 95% Wald Confidence Interval | |
|  |  |  |  |  |  |  | Lower Bound | Upper Bound |
| 1 | 2020 | 2021 | -.0024 | .00087 | 1 | .006 | -.0041 | -.0007 |
|  |  | 2022 | -.0001 | .00081 | 1 | .871 | -.0017 | .0015 |
|  |  | 2023 | .0025 | .00078 | 1 | .001 | .0010 | .0041 |
|  |  | 2024 | .0023 | .00078 | 1 | .004 | .0007 | .0038 |
|  |  | 2025 | -.0029 | .00085 | 1 | .001 | -.0045 | -.0012 |
|  | 2021 | 2020 | .0024 | .00087 | 1 | .006 | .0007 | .0041 |
|  |  | 2022 | .0022 | .00049 | 1 | .000 | .0013 | .0032 |
|  |  | 2023 | .0049 | .00043 | 1 | .000 | .0040 | .0057 |
|  |  | 2024 | .0046 | .00043 | 1 | .000 | .0038 | .0055 |
|  |  | 2025 | -.0005 | .00056 | 1 | .375 | -.0016 | .0006 |
|  | 2022 | 2020 | .0001 | .00081 | 1 | .871 | -.0015 | .0017 |
|  |  | 2021 | -.0022 | .00049 | 1 | .000 | -.0032 | -.0013 |
|  |  | 2023 | .0027 | .00031 | 1 | .000 | .0020 | .0033 |
|  |  | 2024 | .0024 | .00032 | 1 | .000 | .0018 | .0030 |
|  |  | 2025 | -.0027 | .00047 | 1 | .000 | -.0037 | -.0018 |
|  | 2023 | 2020 | -.0025 | .00078 | 1 | .001 | -.0041 | -.0010 |
|  |  | 2021 | -.0049 | .00043 | 1 | .000 | -.0057 | -.0040 |
|  |  | 2022 | -.0027 | .00031 | 1 | .000 | -.0033 | -.0020 |
|  |  | 2024 | -.0003 | .00021 | 1 | .233 | -.0007 | .0002 |
|  |  | 2025 | -.0054 | .00041 | 1 | .000 | -.0062 | -.0046 |
|  | 2024 | 2020 | -.0023 | .00078 | 1 | .004 | -.0038 | -.0007 |
|  |  | 2021 | -.0046 | .00043 | 1 | .000 | -.0055 | -.0038 |
|  |  | 2022 | -.0024 | .00032 | 1 | .000 | -.0030 | -.0018 |
|  |  | 2023 | .0003 | .00021 | 1 | .233 | -.0002 | .0007 |
|  |  | 2025 | -.0051 | .00041 | 1 | .000 | -.0059 | -.0043 |
|  | 2025 | 2020 | .0029 | .00085 | 1 | .001 | .0012 | .0045 |
|  |  | 2021 | .0005 | .00056 | 1 | .375 | -.0006 | .0016 |
|  |  | 2022 | .0027 | .00047 | 1 | .000 | .0018 | .0037 |
|  |  | 2023 | .0054 | .00041 | 1 | .000 | .0046 | .0062 |
|  |  | 2024 | .0051 | .00041 | 1 | .000 | .0043 | .0059 |
| 2 | 2020 | 2021 | .0009 | .00122 | 1 | .460 | -.0015 | .0033 |
|  |  | 2022 | .0019 | .00118 | 1 | .104 | -.0004 | .0042 |
|  |  | 2023 | .0037 | .00115 | 1 | .001 | .0015 | .0060 |
|  |  | 2024 | .0035 | .00115 | 1 | .002 | .0013 | .0058 |
|  |  | 2025 | -.0052 | .00126 | 1 | .000 | -.0077 | -.0027 |
|  | 2021 | 2020 | -.0009 | .00122 | 1 | .460 | -.0033 | .0015 |
|  |  | 2022 | .0010 | .00054 | 1 | .060 | .0000 | .0021 |
|  |  | 2023 | .0028 | .00048 | 1 | .000 | .0019 | .0038 |
|  |  | 2024 | .0026 | .00048 | 1 | .000 | .0017 | .0036 |
|  |  | 2025 | -.0061 | .00071 | 1 | .000 | -.0075 | -.0047 |
|  | 2022 | 2020 | -.0019 | .00118 | 1 | .104 | -.0042 | .0004 |
|  |  | 2021 | -.0010 | .00054 | 1 | .060 | -.0021 | .0000 |
|  |  | 2023 | .0018 | .00033 | 1 | .000 | .0012 | .0025 |
|  |  | 2024 | .0016 | .00033 | 1 | .000 | .0010 | .0023 |
|  |  | 2025 | -.0071 | .00062 | 1 | .000 | -.0083 | -.0059 |
|  | 2023 | 2020 | -.0037 | .00115 | 1 | .001 | -.0060 | -.0015 |
|  |  | 2021 | -.0028 | .00048 | 1 | .000 | -.0038 | -.0019 |
|  |  | 2022 | -.0018 | .00033 | 1 | .000 | -.0025 | -.0012 |
|  |  | 2024 | -.0002 | .00022 | 1 | .348 | -.0006 | .0002 |
|  |  | 2025 | -.0090 | .00057 | 1 | .000 | -.0101 | -.0078 |
|  | 2024 | 2020 | -.0035 | .00115 | 1 | .002 | -.0058 | -.0013 |
|  |  | 2021 | -.0026 | .00048 | 1 | .000 | -.0036 | -.0017 |
|  |  | 2022 | -.0016 | .00033 | 1 | .000 | -.0023 | -.0010 |
|  |  | 2023 | .0002 | .00022 | 1 | .348 | -.0002 | .0006 |
|  |  | 2025 | -.0088 | .00057 | 1 | .000 | -.0099 | -.0076 |
|  | 2025 | 2020 | .0052 | .00126 | 1 | .000 | .0027 | .0077 |
|  |  | 2021 | .0061 | .00071 | 1 | .000 | .0047 | .0075 |
|  |  | 2022 | .0071 | .00062 | 1 | .000 | .0059 | .0083 |
|  |  | 2023 | .0090 | .00057 | 1 | .000 | .0078 | .0101 |
|  |  | 2024 | .0088 | .00057 | 1 | .000 | .0076 | .0099 |

| **Overall Test** | | | |
| --- | --- | --- | --- |
| Diagnostic | Wald χ² | Degrees of Freedom | P |
| 1 | 328.829 | 5 | .000 |
| 2 | 296.230 | 5 | .000 |

**Estimated Marginal Means 10：Year* Diagnostic**

| **Estimate** | | | | | |
| --- | --- | --- | --- | --- | --- |
| Year | Diagnostic | Mean | Standard Error | 95% Wald Confidence Interval | |
|  |  |  |  | Lower Bound | Upper Bound |
| 2020 | 1 | .0229 | .00077 | .0215 | .0245 |
|  | 2 | .0215 | .00115 | .0194 | .0239 |
| 2021 | 1 | .0253 | .00041 | .0245 | .0261 |
|  | 2 | .0206 | .00048 | .0197 | .0216 |
| 2022 | 1 | .0230 | .00028 | .0225 | .0236 |
|  | 2 | .0196 | .00032 | .0190 | .0203 |
| 2023 | 1 | .0204 | .00015 | .0201 | .0207 |
|  | 2 | .0178 | .00020 | .0174 | .0182 |
| 2024 | 1 | .0206 | .00016 | .0203 | .0209 |
|  | 2 | .0180 | .00020 | .0176 | .0184 |
| 2025 | 1 | .0258 | .00038 | .0250 | .0265 |
|  | 2 | .0268 | .00058 | .0256 | .0279 |

| **Pairwise Comparisons** | | | | | | | | |
| --- | --- | --- | --- | --- | --- | --- | --- | --- |
| Year | (I) Diagnostic | (J) Diagnostic | Mean Difference (I-J) | Standard Error | Degrees of Freedom | P | 95% Wald Confidence Interval | |
|  |  |  |  |  |  |  | Lower Bound | Upper Bound |
| 2020 | 1 | 2 | .0014 | .00130 | 1 | .296 | -.0012 | .0039 |
|  | 2 | 1 | -.0014 | .00130 | 1 | .296 | -.0039 | .0012 |
| 2021 | 1 | 2 | .0046 | .00056 | 1 | .000 | .0035 | .0057 |
|  | 2 | 1 | -.0046 | .00056 | 1 | .000 | -.0057 | -.0035 |
| 2022 | 1 | 2 | .0034 | .00036 | 1 | .000 | .0027 | .0041 |
|  | 2 | 1 | -.0034 | .00036 | 1 | .000 | -.0041 | -.0027 |
| 2023 | 1 | 2 | .0026 | .00021 | 1 | .000 | .0022 | .0030 |
|  | 2 | 1 | -.0026 | .00021 | 1 | .000 | -.0030 | -.0022 |
| 2024 | 1 | 2 | .0026 | .00021 | 1 | .000 | .0022 | .0031 |
|  | 2 | 1 | -.0026 | .00021 | 1 | .000 | -.0031 | -.0022 |
| 2025 | 1 | 2 | -.0010 | .00064 | 1 | .125 | -.0022 | .0003 |
|  | 2 | 1 | .0010 | .00064 | 1 | .125 | -.0003 | .0022 |

| **Overall Test** | | | |
| --- | --- | --- | --- |
| Year | Wald χ² | Degrees of Freedom | P |
| 2020 | 1.090 | 1 | .296 |
| 2021 | 68.477 | 1 | .000 |
| 2022 | 88.034 | 1 | .000 |
| 2023 | 144.544 | 1 | .000 |
| 2024 | 150.436 | 1 | .000 |
| 2025 | 2.357 | 1 | .125 |

**Estimated Marginal Means 11：Gender* Age**

| **Estimate** | | | | | |
| --- | --- | --- | --- | --- | --- |
| Gender | Age | Mean | Standard Error | 95% Wald Confidence Interval | |
|  |  |  |  | Lower Bound | Upper Bound |
| 1 | 1 | .0237 | .00049 | .0227 | .0246 |
|  | 2 | .0205 | .00017 | .0201 | .0208 |
|  | 3 | .0225 | .00034 | .0218 | .0232 |
|  | 4 | .0240 | .00041 | .0232 | .0248 |
| 2 | 1 | .0236 | .00053 | .0225 | .0246 |
|  | 2 | .0190 | .00018 | .0186 | .0193 |
|  | 3 | .0202 | .00033 | .0196 | .0209 |
|  | 4 | .0207 | .00039 | .0200 | .0215 |

| **Pairwise Comparisons** | | | | | | | | |
| --- | --- | --- | --- | --- | --- | --- | --- | --- |
| Age | (I) Gender | (J) Gender | Mean Difference (I-J) | Standard Error | Degrees of Freedom | P | 95% Wald Confidence Interval | |
|  |  |  |  |  |  |  | Lower Bound | Upper Bound |
| 1 | 1 | 2 | .0001 | .00051 | 1 | .837 | -.0009 | .0011 |
|  | 2 | 1 | -.0001 | .00051 | 1 | .837 | -.0011 | .0009 |
| 2 | 1 | 2 | .0015 | .00021 | 1 | .000 | .0011 | .0019 |
|  | 2 | 1 | -.0015 | .00021 | 1 | .000 | -.0019 | -.0011 |
| 3 | 1 | 2 | .0023 | .00027 | 1 | .000 | .0017 | .0028 |
|  | 2 | 1 | -.0023 | .00027 | 1 | .000 | -.0028 | -.0017 |
| 4 | 1 | 2 | .0032 | .00032 | 1 | .000 | .0026 | .0039 |
|  | 2 | 1 | -.0032 | .00032 | 1 | .000 | -.0039 | -.0026 |

| **Overall Test** | | | |
| --- | --- | --- | --- |
| Age | Wald χ² | Degrees of Freedom | P |
| 1 | .042 | 1 | .837 |
| 2 | 49.797 | 1 | .000 |
| 3 | 68.070 | 1 | .000 |
| 4 | 100.459 | 1 | .000 |

**Estimated Marginal Means 12：Gender* Age**

| **Estimate** | | | | | |
| --- | --- | --- | --- | --- | --- |
| Gender | Age | Mean | Standard Error | 95% Wald Confidence Interval | |
|  |  |  |  | Lower Bound | Upper Bound |
| 1 | 1 | .0237 | .00049 | .0227 | .0246 |
|  | 2 | .0205 | .00017 | .0201 | .0208 |
|  | 3 | .0225 | .00034 | .0218 | .0232 |
|  | 4 | .0240 | .00041 | .0232 | .0248 |
| 2 | 1 | .0236 | .00053 | .0225 | .0246 |
|  | 2 | .0190 | .00018 | .0186 | .0193 |
|  | 3 | .0202 | .00033 | .0196 | .0209 |
|  | 4 | .0207 | .00039 | .0200 | .0215 |

| **Pairwise Comparisons** | | | | | | | | |
| --- | --- | --- | --- | --- | --- | --- | --- | --- |
| Gender | (I) Age | (J) Age | Mean Difference (I-J) | Standard Error | Degrees of Freedom | P | 95% Wald Confidence Interval | |
|  |  |  |  |  |  |  | Lower Bound | Upper Bound |
| 1 | 1 | 2 | .0032 | .00050 | 1 | .000 | .0022 | .0042 |
|  |  | 3 | .0012 | .00058 | 1 | .039 | .0001 | .0023 |
|  |  | 4 | -.0003 | .00064 | 1 | .642 | -.0015 | .0010 |
|  | 2 | 1 | -.0032 | .00050 | 1 | .000 | -.0042 | -.0022 |
|  |  | 3 | -.0020 | .00036 | 1 | .000 | -.0027 | -.0013 |
|  |  | 4 | -.0035 | .00043 | 1 | .000 | -.0044 | -.0027 |
|  | 3 | 1 | -.0012 | .00058 | 1 | .039 | -.0023 | -.0001 |
|  |  | 2 | .0020 | .00036 | 1 | .000 | .0013 | .0027 |
|  |  | 4 | -.0015 | .00053 | 1 | .005 | -.0025 | -.0005 |
|  | 4 | 1 | .0003 | .00064 | 1 | .642 | -.0010 | .0015 |
|  |  | 2 | .0035 | .00043 | 1 | .000 | .0027 | .0044 |
|  |  | 3 | .0015 | .00053 | 1 | .005 | .0005 | .0025 |
| 2 | 1 | 2 | .0046 | .00054 | 1 | .000 | .0035 | .0057 |
|  |  | 3 | .0033 | .00060 | 1 | .000 | .0022 | .0045 |
|  |  | 4 | .0028 | .00065 | 1 | .000 | .0015 | .0041 |
|  | 2 | 1 | -.0046 | .00054 | 1 | .000 | -.0057 | -.0035 |
|  |  | 3 | -.0013 | .00034 | 1 | .000 | -.0019 | -.0006 |
|  |  | 4 | -.0018 | .00040 | 1 | .000 | -.0026 | -.0010 |
|  | 3 | 1 | -.0033 | .00060 | 1 | .000 | -.0045 | -.0022 |
|  |  | 2 | .0013 | .00034 | 1 | .000 | .0006 | .0019 |
|  |  | 4 | -.0005 | .00048 | 1 | .280 | -.0015 | .0004 |
|  | 4 | 1 | -.0028 | .00065 | 1 | .000 | -.0041 | -.0015 |
|  |  | 2 | .0018 | .00040 | 1 | .000 | .0010 | .0026 |
|  |  | 3 | .0005 | .00048 | 1 | .280 | -.0004 | .0015 |

| **Overall Test** | | | |
| --- | --- | --- | --- |
| Gender | Wald χ² | Degrees of Freedom | P |
| 1 | 113.360 | 3 | .000 |
| 2 | 91.151 | 3 | .000 |

**Estimated Marginal Means 13：Gender* Diagnostic**

| **Estimate** | | | | | |
| --- | --- | --- | --- | --- | --- |
| Gender | Diagnostic | Mean | Standard Error | 95% Wald Confidence Interval | |
|  |  |  |  | Lower Bound | Upper Bound |
| 1 | 1 | .0239 | .00020 | .0235 | .0242 |
|  | 2 | .0214 | .00029 | .0208 | .0220 |
| 2 | 1 | .0220 | .00020 | .0216 | .0224 |
|  | 2 | .0197 | .00029 | .0191 | .0203 |

| **Pairwise Comparisons** | | | | | | | | |
| --- | --- | --- | --- | --- | --- | --- | --- | --- |
| Diagnostic | (I) Gender | (J) Gender | Mean Difference (I-J) | Standard Error | Degrees of Freedom | P | 95% Wald Confidence Interval | |
|  |  |  |  |  |  |  | Lower Bound | Upper Bound |
| 1 | 1 | 2 | .0018 | .00023 | 1 | .000 | .0014 | .0023 |
|  | 2 | 1 | -.0018 | .00023 | 1 | .000 | -.0023 | -.0014 |
| 2 | 1 | 2 | .0017 | .00025 | 1 | .000 | .0013 | .0022 |
|  | 2 | 1 | -.0017 | .00025 | 1 | .000 | -.0022 | -.0013 |

| **Overall Test** | | | |
| --- | --- | --- | --- |
| Diagnostic | Wald χ² | Degrees of Freedom | P |
| 1 | 63.263 | 1 | .000 |
| 2 | 49.677 | 1 | .000 |

**Estimated Marginal Means 14：Gender* Diagnostic**

| **Estimate** | | | | | |
| --- | --- | --- | --- | --- | --- |
| Gender | Diagnostic | Mean | Standard Error | 95% Wald Confidence Interval | |
|  |  |  |  | Lower Bound | Upper Bound |
| 1 | 1 | .0239 | .00020 | .0235 | .0242 |
|  | 2 | .0214 | .00029 | .0208 | .0220 |
| 2 | 1 | .0220 | .00020 | .0216 | .0224 |
|  | 2 | .0197 | .00029 | .0191 | .0203 |

| **Pairwise Comparisons** | | | | | | | | |
| --- | --- | --- | --- | --- | --- | --- | --- | --- |
| Gender | (I) Diagnostic | (J) Diagnostic | Mean Difference (I-J) | Standard Error | Degrees of Freedom | P | 95% Wald Confidence Interval | |
|  |  |  |  |  |  |  | Lower Bound | Upper Bound |
| 1 | 1 | 2 | .0024 | .00033 | 1 | .000 | .0018 | .0031 |
|  | 2 | 1 | -.0024 | .00033 | 1 | .000 | -.0031 | -.0018 |
| 2 | 1 | 2 | .0023 | .00031 | 1 | .000 | .0017 | .0029 |
|  | 2 | 1 | -.0023 | .00031 | 1 | .000 | -.0029 | -.0017 |

| **Overall Test** | | | |
| --- | --- | --- | --- |
| Gender | Wald χ² | Degrees of Freedom | P |
| 1 | 55.451 | 1 | .000 |
| 2 | 55.076 | 1 | .000 |

**Estimated Marginal Means 15：Age* Diagnostic**

| **Estimate** | | | | | |
| --- | --- | --- | --- | --- | --- |
| Age | Diagnostic | Mean | Standard Error | 95% Wald Confidence Interval | |
|  |  |  |  | Lower Bound | Upper Bound |
| 1 | 1 | .0278 | .00034 | .0271 | .0285 |
|  | 2 | .0201 | .00070 | .0188 | .0215 |
| 2 | 1 | .0202 | .00016 | .0199 | .0206 |
|  | 2 | .0192 | .00020 | .0188 | .0196 |
| 3 | 1 | .0215 | .00031 | .0209 | .0222 |
|  | 2 | .0211 | .00037 | .0204 | .0219 |
| 4 | 1 | .0227 | .00041 | .0220 | .0236 |
|  | 2 | .0219 | .00040 | .0211 | .0227 |

| **Pairwise Comparisons** | | | | | | | | |
| --- | --- | --- | --- | --- | --- | --- | --- | --- |
| Diagnostic | (I) Age | (J) Age | Mean Difference (I-J) | Standard Error | Degrees of Freedom | P | 95% Wald Confidence Interval | |
|  |  |  |  |  |  |  | Lower Bound | Upper Bound |
| 1 | 1 | 2 | .0075 | .00038 | 1 | .000 | .0068 | .0083 |
|  |  | 3 | .0063 | .00046 | 1 | .000 | .0053 | .0072 |
|  |  | 4 | .0050 | .00054 | 1 | .000 | .0040 | .0061 |
|  | 2 | 1 | -.0075 | .00038 | 1 | .000 | -.0083 | -.0068 |
|  |  | 3 | -.0013 | .00034 | 1 | .000 | -.0020 | -.0006 |
|  |  | 4 | -.0025 | .00042 | 1 | .000 | -.0033 | -.0017 |
|  | 3 | 1 | -.0063 | .00046 | 1 | .000 | -.0072 | -.0053 |
|  |  | 2 | .0013 | .00034 | 1 | .000 | .0006 | .0020 |
|  |  | 4 | -.0012 | .00050 | 1 | .016 | -.0022 | -.0002 |
|  | 4 | 1 | -.0050 | .00054 | 1 | .000 | -.0061 | -.0040 |
|  |  | 2 | .0025 | .00042 | 1 | .000 | .0017 | .0033 |
|  |  | 3 | .0012 | .00050 | 1 | .016 | .0002 | .0022 |
| 2 | 1 | 2 | .0009 | .00069 | 1 | .176 | -.0004 | .0023 |
|  |  | 3 | -.0010 | .00074 | 1 | .161 | -.0025 | .0004 |
|  |  | 4 | -.0018 | .00077 | 1 | .021 | -.0033 | -.0003 |
|  | 2 | 1 | -.0009 | .00069 | 1 | .176 | -.0023 | .0004 |
|  |  | 3 | -.0020 | .00036 | 1 | .000 | -.0027 | -.0013 |
|  |  | 4 | -.0027 | .00041 | 1 | .000 | -.0035 | -.0019 |
|  | 3 | 1 | .0010 | .00074 | 1 | .161 | -.0004 | .0025 |
|  |  | 2 | .0020 | .00036 | 1 | .000 | .0013 | .0027 |
|  |  | 4 | -.0007 | .00050 | 1 | .140 | -.0017 | .0002 |
|  | 4 | 1 | .0018 | .00077 | 1 | .021 | .0003 | .0033 |
|  |  | 2 | .0027 | .00041 | 1 | .000 | .0019 | .0035 |
|  |  | 3 | .0007 | .00050 | 1 | .140 | -.0002 | .0017 |

| **Overall Test** | | | |
| --- | --- | --- | --- |
| Diagnostic | Wald χ² | Degrees of Freedom | P |
| 1 | 410.228 | 3 | .000 |
| 2 | 63.714 | 3 | .000 |

**Estimated Marginal Means 16：Age* Diagnostic**

| **Estimate** | | | | | |
| --- | --- | --- | --- | --- | --- |
| Age | Diagnostic | Mean | Standard Error | 95% Wald Confidence Interval | |
|  |  |  |  | Lower Bound | Upper Bound |
| 1 | 1 | .0278 | .00034 | .0271 | .0285 |
|  | 2 | .0201 | .00070 | .0188 | .0215 |
| 2 | 1 | .0202 | .00016 | .0199 | .0206 |
|  | 2 | .0192 | .00020 | .0188 | .0196 |
| 3 | 1 | .0215 | .00031 | .0209 | .0222 |
|  | 2 | .0211 | .00037 | .0204 | .0219 |
| 4 | 1 | .0227 | .00041 | .0220 | .0236 |
|  | 2 | .0219 | .00040 | .0211 | .0227 |

| **Pairwise Comparisons** | | | | | | | | |
| --- | --- | --- | --- | --- | --- | --- | --- | --- |
| Age | (I) Diagnostic | (J) Diagnostic | Mean Difference (I-J) | Standard Error | Degrees of Freedom | P | 95% Wald Confidence Interval | |
|  |  |  |  |  |  |  | Lower Bound | Upper Bound |
| 1 | 1 | 2 | .0077 | .00076 | 1 | .000 | .0062 | .0092 |
|  | 2 | 1 | -.0077 | .00076 | 1 | .000 | -.0092 | -.0062 |
| 2 | 1 | 2 | .0011 | .00024 | 1 | .000 | .0006 | .0016 |
|  | 2 | 1 | -.0011 | .00024 | 1 | .000 | -.0016 | -.0006 |
| 3 | 1 | 2 | .0004 | .00031 | 1 | .181 | -.0002 | .0010 |
|  | 2 | 1 | -.0004 | .00031 | 1 | .181 | -.0010 | .0002 |
| 4 | 1 | 2 | .0009 | .00035 | 1 | .012 | .0002 | .0016 |
|  | 2 | 1 | -.0009 | .00035 | 1 | .012 | -.0016 | -.0002 |

| **Overall Test** | | | |
| --- | --- | --- | --- |
| Age | Wald χ² | Degrees of Freedom | P |
| 1 | 101.785 | 1 | .000 |
| 2 | 19.980 | 1 | .000 |
| 3 | 1.790 | 1 | .181 |
| 4 | 6.246 | 1 | .012 |
