# Supplementary material for: The impact of COVID-19 social isolation and reduced microbial exposure on the immune system in children: a retrospective study
Source: PeerJ. 2026 Jul 7;14:e21469. doi: 10.7717/peerj.21469 (PMC13353229; doi:10.7717/peerj.21469)
Supplement: Supplemental Information 9 [file peerj-14-21469-s009.docx]

**Immunoglobulin A Generalized Linear Model**

For immunoglobulin analysis, patients were grouped as follows:
Group 1: 0–1 year,
Group 2: 1–4 years,
Group 3: 4–6 years,
Group 4: 6–13 years.

| **Parameter Estimate** | | | | | | | |
| --- | --- | --- | --- | --- | --- | --- | --- |
| Parameter | B | Standard Error | 95% Wald Confidence Interval | | Hypothesis Testing | | |
|  |  |  | Lower Bound | Upper Bound | Wald χ² | Degrees of Freedom | P |
| （Intercept） | .436 | .0800 | .280 | .593 | 29.754 | 1 | .000 |
| [Year=2020] | .009 | .1613 | -.307 | .325 | .003 | 1 | .955 |
| [Year=2021] | -.104 | .1175 | -.335 | .126 | .790 | 1 | .374 |
| [Year=2022] | .033 | .1028 | -.169 | .234 | .101 | 1 | .751 |
| [Year=2023] | -.027 | .0837 | -.191 | .137 | .108 | 1 | .743 |
| [Year=2024] | .120 | .0843 | -.045 | .285 | 2.019 | 1 | .155 |
| [Year=2025] | 0 | . | . | . | . | . | . |
| [Gender=1] | .093 | .0592 | -.023 | .210 | 2.488 | 1 | .115 |
| [Gender=2] | 0 | . | . | . | . | . | . |
| [Age=1] | -2.038 | .0845 | -2.203 | -1.872 | 581.465 | 1 | .000 |
| [Age=2] | -.953 | .0815 | -1.112 | -.793 | 136.543 | 1 | .000 |
| [Age=3] | -.389 | .0915 | -.569 | -.210 | 18.125 | 1 | .000 |
| [Age=4] | 0 | . | . | . | . | . | . |
| [Diagnostic=1] | .112 | .0649 | -.015 | .239 | 2.982 | 1 | .084 |
| [Diagnostic=2] | 0 | . | . | . | . | . | . |
| [Year=2020] * [Gender=1] | -.156 | .0923 | -.337 | .025 | 2.870 | 1 | .090 |
| [Year=2020] * [Gender=2] | 0 | . | . | . | . | . | . |
| [Year=2021] * [Gender=1] | -.065 | .0655 | -.194 | .063 | .999 | 1 | .318 |
| [Year=2021] * [Gender=2] | 0 | . | . | . | . | . | . |
| [Year=2022] * [Gender=1] | -.014 | .0501 | -.113 | .084 | .083 | 1 | .773 |
| [Year=2022] * [Gender=2] | 0 | . | . | . | . | . | . |
| [Year=2023] * [Gender=1] | -.016 | .0495 | -.113 | .081 | .107 | 1 | .744 |
| [Year=2023] * [Gender=2] | 0 | . | . | . | . | . | . |
| [Year=2024] * [Gender=1] | -.058 | .0485 | -.153 | .037 | 1.416 | 1 | .234 |
| [Year=2024] * [Gender=2] | 0 | . | . | . | . | . | . |
| [Year=2025] * [Gender=1] | 0 | . | . | . | . | . | . |
| [Year=2025] * [Gender=2] | 0 | . | . | . | . | . | . |
| [Year=2020] * [Age=1] | -.138 | .1600 | -.451 | .176 | .741 | 1 | .389 |
| [Year=2020] * [Age=2] | -.035 | .1559 | -.341 | .270 | .051 | 1 | .821 |
| [Year=2020] * [Age=3] | .090 | .2095 | -.321 | .500 | .183 | 1 | .669 |
| [Year=2020] * [Age=4] | 0 | . | . | . | . | . | . |
| [Year=2021] * [Age=1] | -.053 | .1216 | -.291 | .186 | .188 | 1 | .664 |
| [Year=2021] * [Age=2] | .160 | .1173 | -.070 | .390 | 1.858 | 1 | .173 |
| [Year=2021] * [Age=3] | .141 | .1305 | -.115 | .396 | 1.159 | 1 | .282 |
| [Year=2021] * [Age=4] | 0 | . | . | . | . | . | . |
| [Year=2022] * [Age=1] | -.087 | .1058 | -.294 | .120 | .676 | 1 | .411 |
| [Year=2022] * [Age=2] | .027 | .1029 | -.175 | .229 | .070 | 1 | .792 |
| [Year=2022] * [Age=3] | -.006 | .1135 | -.228 | .217 | .003 | 1 | .960 |
| [Year=2022] * [Age=4] | 0 | . | . | . | . | . | . |
| [Year=2023] * [Age=1] | -.194 | .0884 | -.368 | -.021 | 4.834 | 1 | .028 |
| [Year=2023] * [Age=2] | -.039 | .0843 | -.205 | .126 | .218 | 1 | .641 |
| [Year=2023] * [Age=3] | .059 | .0953 | -.128 | .245 | .378 | 1 | .539 |
| [Year=2023] * [Age=4] | 0 | . | . | . | . | . | . |
| [Year=2024] * [Age=1] | -.213 | .0879 | -.385 | -.041 | 5.862 | 1 | .015 |
| [Year=2024] * [Age=2] | -.130 | .0852 | -.297 | .037 | 2.325 | 1 | .127 |
| [Year=2024] * [Age=3] | -.037 | .0962 | -.226 | .151 | .150 | 1 | .698 |
| [Year=2024] * [Age=4] | 0 | . | . | . | . | . | . |
| [Year=2025] * [Age=1] | 0 | . | . | . | . | . | . |
| [Year=2025] * [Age=2] | 0 | . | . | . | . | . | . |
| [Year=2025] * [Age=3] | 0 | . | . | . | . | . | . |
| [Year=2025] * [Age=4] | 0 | . | . | . | . | . | . |
| [Year=2020] * [Diagnostic=1] | -.018 | .0970 | -.208 | .172 | .034 | 1 | .853 |
| [Year=2020] * [Diagnostic=2] | 0 | . | . | . | . | . | . |
| [Year=2021] * [Diagnostic=1] | .071 | .0704 | -.067 | .209 | 1.014 | 1 | .314 |
| [Year=2021] * [Diagnostic=2] | 0 | . | . | . | . | . | . |
| [Year=2022] * [Diagnostic=1] | -.125 | .0554 | -.234 | -.017 | 5.119 | 1 | .024 |
| [Year=2022] * [Diagnostic=2] | 0 | . | . | . | . | . | . |
| [Year=2023] * [Diagnostic=1] | -.098 | .0526 | -.201 | .005 | 3.463 | 1 | .063 |
| [Year=2023] * [Diagnostic=2] | 0 | . | . | . | . | . | . |
| [Year=2024] * [Diagnostic=1] | -.134 | .0535 | -.239 | -.029 | 6.280 | 1 | .012 |
| [Year=2024] * [Diagnostic=2] | 0 | . | . | . | . | . | . |
| [Year=2025] * [Diagnostic=1] | 0 | . | . | . | . | . | . |
| [Year=2025] * [Diagnostic=2] | 0 | . | . | . | . | . | . |
| [Gender=1] * [Age=1] | .062 | .0509 | -.038 | .162 | 1.488 | 1 | .222 |
| [Gender=1] * [Age=2] | -.003 | .0489 | -.099 | .093 | .005 | 1 | .946 |
| [Gender=1] * [Age=3] | .009 | .0542 | -.098 | .115 | .026 | 1 | .873 |
| [Gender=1] * [Age=4] | 0 | . | . | . | . | . | . |
| [Gender=2] * [Age=1] | 0 | . | . | . | . | . | . |
| [Gender=2] * [Age=2] | 0 | . | . | . | . | . | . |
| [Gender=2] * [Age=3] | 0 | . | . | . | . | . | . |
| [Gender=2] * [Age=4] | 0 | . | . | . | . | . | . |
| [Gender=1] * [Diagnostic=1] | .050 | .0332 | -.015 | .115 | 2.282 | 1 | .131 |
| [Gender=1] * [Diagnostic=2] | 0 | . | . | . | . | . | . |
| [Gender=2] * [Diagnostic=1] | 0 | . | . | . | . | . | . |
| [Gender=2] * [Diagnostic=2] | 0 | . | . | . | . | . | . |
| [Age=1] * [Diagnostic=1] | .022 | .0589 | -.093 | .138 | .144 | 1 | .704 |
| [Age=1] * [Diagnostic=2] | 0 | . | . | . | . | . | . |
| [Age=2] * [Diagnostic=1] | -.203 | .0536 | -.308 | -.098 | 14.381 | 1 | .000 |
| [Age=2] * [Diagnostic=2] | 0 | . | . | . | . | . | . |
| [Age=3] * [Diagnostic=1] | -.023 | .0595 | -.140 | .093 | .155 | 1 | .694 |
| [Age=3] * [Diagnostic=2] | 0 | . | . | . | . | . | . |
| [Age=4] * [Diagnostic=1] | 0 | . | . | . | . | . | . |
| [Age=4] * [Diagnostic=2] | 0 | . | . | . | . | . | . |
| （标度） | .388 | .0058 | .377 | .400 |  |  |  |

**Estimated Marginal Means 1：Year**

| **Estimate** | | | | |
| --- | --- | --- | --- | --- |
| Year | Mean | Standard Error | 95% Wald Confidence Interval | |
|  |  |  | Lower Bound | Upper Bound |
| 2020 | .6642 | .03569 | .5978 | .7380 |
| 2021 | .7047 | .02252 | .6619 | .7502 |
| 2022 | .6950 | .01600 | .6643 | .7271 |
| 2023 | .6450 | .01066 | .6244 | .6662 |
| 2024 | .6830 | .01190 | .6601 | .7067 |
| 2025 | .7333 | .01787 | .6991 | .7691 |

| **Pairwise Comparisons** | | | | | | | |
| --- | --- | --- | --- | --- | --- | --- | --- |
| (I) Year | (J) Year | Mean Difference (I-J) | Standard Error | Degrees of Freedom | P | 95% Wald Confidence Interval | |
|  |  |  |  |  |  | Lower Bound | Upper Bound |
| 2020 | 2021 | -.0404 | .04215 | 1 | .337 | -.1231 | .0422 |
|  | 2022 | -.0307 | .03909 | 1 | .432 | -.1074 | .0459 |
|  | 2023 | .0192 | .03725 | 1 | .605 | -.0538 | .0923 |
|  | 2024 | -.0188 | .03764 | 1 | .618 | -.0925 | .0550 |
|  | 2025 | -.0690 | .03985 | 1 | .083 | -.1471 | .0091 |
| 2021 | 2020 | .0404 | .04215 | 1 | .337 | -.0422 | .1231 |
|  | 2022 | .0097 | .02746 | 1 | .724 | -.0441 | .0635 |
|  | 2023 | .0597 | .02483 | 1 | .016 | .0110 | .1084 |
|  | 2024 | .0217 | .02536 | 1 | .393 | -.0280 | .0714 |
|  | 2025 | -.0286 | .02867 | 1 | .319 | -.0848 | .0276 |
| 2022 | 2020 | .0307 | .03909 | 1 | .432 | -.0459 | .1074 |
|  | 2021 | -.0097 | .02746 | 1 | .724 | -.0635 | .0441 |
|  | 2023 | .0500 | .01911 | 1 | .009 | .0125 | .0874 |
|  | 2024 | .0120 | .01979 | 1 | .545 | -.0268 | .0508 |
|  | 2025 | -.0383 | .02391 | 1 | .109 | -.0852 | .0086 |
| 2023 | 2020 | -.0192 | .03725 | 1 | .605 | -.0923 | .0538 |
|  | 2021 | -.0597 | .02483 | 1 | .016 | -.1084 | -.0110 |
|  | 2022 | -.0500 | .01911 | 1 | .009 | -.0874 | -.0125 |
|  | 2024 | -.0380 | .01585 | 1 | .017 | -.0691 | -.0069 |
|  | 2025 | -.0883 | .02077 | 1 | .000 | -.1290 | -.0476 |
| 2024 | 2020 | .0188 | .03764 | 1 | .618 | -.0550 | .0925 |
|  | 2021 | -.0217 | .02536 | 1 | .393 | -.0714 | .0280 |
|  | 2022 | -.0120 | .01979 | 1 | .545 | -.0508 | .0268 |
|  | 2023 | .0380 | .01585 | 1 | .017 | .0069 | .0691 |
|  | 2025 | -.0503 | .02144 | 1 | .019 | -.0923 | -.0083 |
| 2025 | 2020 | .0690 | .03985 | 1 | .083 | -.0091 | .1471 |
|  | 2021 | .0286 | .02867 | 1 | .319 | -.0276 | .0848 |
|  | 2022 | .0383 | .02391 | 1 | .109 | -.0086 | .0852 |
|  | 2023 | .0883 | .02077 | 1 | .000 | .0476 | .1290 |
|  | 2024 | .0503 | .02144 | 1 | .019 | .0083 | .0923 |

| **Overall Test** | | |
| --- | --- | --- |
| Wald χ² | Degrees of Freedom | P |
| 22.118 | 5 | .000 |

**Estimated Marginal Means 2：Gender**

| **Estimate** | | | | |
| --- | --- | --- | --- | --- |
| Gender | Mean | Standard Error | 95% Wald Confidence Interval | |
|  |  |  | Lower Bound | Upper Bound |
| 1 | .7163 | .01081 | .6954 | .7378 |
| 2 | .6588 | .01203 | .6356 | .6828 |

| **Pairwise Comparisons** | | | | | | | |
| --- | --- | --- | --- | --- | --- | --- | --- |
| (I) Gender | (J) Gender | Mean Difference (I-J) | Standard Error | Degrees of Freedom | P | 95% Wald Confidence Interval | |
|  |  |  |  |  |  | Lower Bound | Upper Bound |
| 1 | 2 | .0576 | .01506 | 1 | .000 | .0280 | .0871 |
| 2 | 1 | -.0576 | .01506 | 1 | .000 | -.0871 | -.0280 |

| **Overall Test** | | |
| --- | --- | --- |
| Wald χ² | Degrees of Freedom | P |
| 14.595 | 1 | .000 |

**Estimated Marginal Means 3：Age**

| **Estimate** | | | | |
| --- | --- | --- | --- | --- |
| Age | Mean | Standard Error | 95% Wald Confidence Interval | |
|  |  |  | Lower Bound | Upper Bound |
| 1 | .2011 | .00405 | .1933 | .2092 |
| 2 | .5750 | .00896 | .5577 | .5929 |
| 3 | 1.1616 | .03484 | 1.0952 | 1.2319 |
| 4 | 1.6578 | .05140 | 1.5601 | 1.7617 |

| **Pairwise Comparisons** | | | | | | | |
| --- | --- | --- | --- | --- | --- | --- | --- |
| (I) Age | (J) Age | Mean Difference (I-J) | Standard Error | Degrees of Freedom | P | 95% Wald Confidence Interval | |
|  |  |  |  |  |  | Lower Bound | Upper Bound |
| 1 | 2 | -.3739 | .00978 | 1 | .000 | -.3931 | -.3548 |
|  | 3 | -.9605 | .03499 | 1 | .000 | -1.0291 | -.8919 |
|  | 4 | -1.4568 | .05156 | 1 | .000 | -1.5578 | -1.3557 |
| 2 | 1 | .3739 | .00978 | 1 | .000 | .3548 | .3931 |
|  | 3 | -.5865 | .03594 | 1 | .000 | -.6570 | -.5161 |
|  | 4 | -1.0828 | .05215 | 1 | .000 | -1.1850 | -.9806 |
| 3 | 1 | .9605 | .03499 | 1 | .000 | .8919 | 1.0291 |
|  | 2 | .5865 | .03594 | 1 | .000 | .5161 | .6570 |
|  | 4 | -.4963 | .06218 | 1 | .000 | -.6182 | -.3744 |
| 4 | 1 | 1.4568 | .05156 | 1 | .000 | 1.3557 | 1.5578 |
|  | 2 | 1.0828 | .05215 | 1 | .000 | .9806 | 1.1850 |
|  | 3 | .4963 | .06218 | 1 | .000 | .3744 | .6182 |

| **Overall Test** | | |
| --- | --- | --- |
| Wald χ² | Degrees of Freedom | P |
| 2852.269 | 3 | .000 |

**Estimated Marginal Means 4：Diagnostic**

| **Estimate** | | | | |
| --- | --- | --- | --- | --- |
| Diagnostic | Mean | Standard Error | 95% Wald Confidence Interval | |
|  |  |  | Lower Bound | Upper Bound |
| 1 | .6992 | .01330 | .6737 | .7258 |
| 2 | .6748 | .00997 | .6556 | .6947 |

| **Pairwise Comparisons** | | | | | | | |
| --- | --- | --- | --- | --- | --- | --- | --- |
| (I) Diagnostic | (J) Diagnostic | Mean Difference (I-J) | Standard Error | Degrees of Freedom | P | 95% Wald Confidence Interval | |
|  |  |  |  |  |  | Lower Bound | Upper Bound |
| 1 | 2 | .0244 | .01581 | 1 | .123 | -.0066 | .0554 |
| 2 | 1 | -.0244 | .01581 | 1 | .123 | -.0554 | .0066 |

| **Overall Test** | | |
| --- | --- | --- |
| Wald χ² | Degrees of Freedom | P |
| 2.380 | 1 | .123 |

**Estimated Marginal Means 5：Year* Gender**

| **Estimate** | | | | | |
| --- | --- | --- | --- | --- | --- |
| Year | Gender | Mean | Standard Error | 95% Wald Confidence Interval | |
|  |  |  |  | Lower Bound | Upper Bound |
| 2020 | 1 | .6573 | .04098 | .5817 | .7427 |
|  | 2 | .6712 | .04928 | .5813 | .7751 |
| 2021 | 1 | .7298 | .02593 | .6807 | .7824 |
|  | 2 | .6804 | .03180 | .6209 | .7457 |
| 2022 | 1 | .7383 | .01957 | .7009 | .7777 |
|  | 2 | .6542 | .01954 | .6170 | .6936 |
| 2023 | 1 | .6846 | .01410 | .6575 | .7128 |
|  | 2 | .6076 | .01450 | .5799 | .6367 |
| 2024 | 1 | .7101 | .01496 | .6813 | .7400 |
|  | 2 | .6570 | .01591 | .6265 | .6889 |
| 2025 | 1 | .7846 | .02348 | .7399 | .8320 |
|  | 2 | .6852 | .02318 | .6413 | .7322 |

| **Pairwise Comparisons** | | | | | | | | |
| --- | --- | --- | --- | --- | --- | --- | --- | --- |
| Gender | (I) Year | (J) Year | Mean Difference (I-J) | Standard Error | Degrees of Freedom | P | 95% Wald Confidence Interval | |
|  |  |  |  |  |  |  | Lower Bound | Upper Bound |
| 1 | 2020 | 2021 | -.0725 | .04837 | 1 | .134 | -.1673 | .0223 |
|  |  | 2022 | -.0810 | .04517 | 1 | .073 | -.1695 | .0075 |
|  |  | 2023 | -.0273 | .04322 | 1 | .527 | -.1120 | .0574 |
|  |  | 2024 | -.0528 | .04345 | 1 | .225 | -.1379 | .0324 |
|  |  | 2025 | -.1273 | .04698 | 1 | .007 | -.2194 | -.0352 |
|  | 2021 | 2020 | .0725 | .04837 | 1 | .134 | -.0223 | .1673 |
|  |  | 2022 | -.0085 | .03215 | 1 | .791 | -.0715 | .0545 |
|  |  | 2023 | .0452 | .02928 | 1 | .123 | -.0122 | .1025 |
|  |  | 2024 | .0197 | .02970 | 1 | .507 | -.0385 | .0779 |
|  |  | 2025 | -.0549 | .03471 | 1 | .114 | -.1229 | .0132 |
|  | 2022 | 2020 | .0810 | .04517 | 1 | .073 | -.0075 | .1695 |
|  |  | 2021 | .0085 | .03215 | 1 | .791 | -.0545 | .0715 |
|  |  | 2023 | .0537 | .02359 | 1 | .023 | .0075 | .0999 |
|  |  | 2024 | .0283 | .02401 | 1 | .239 | -.0188 | .0753 |
|  |  | 2025 | -.0463 | .03003 | 1 | .123 | -.1052 | .0125 |
|  | 2023 | 2020 | .0273 | .04322 | 1 | .527 | -.0574 | .1120 |
|  |  | 2021 | -.0452 | .02928 | 1 | .123 | -.1025 | .0122 |
|  |  | 2022 | -.0537 | .02359 | 1 | .023 | -.0999 | -.0075 |
|  |  | 2024 | -.0254 | .02005 | 1 | .204 | -.0647 | .0138 |
|  |  | 2025 | -.1000 | .02705 | 1 | .000 | -.1530 | -.0470 |
|  | 2024 | 2020 | .0528 | .04345 | 1 | .225 | -.0324 | .1379 |
|  |  | 2021 | -.0197 | .02970 | 1 | .507 | -.0779 | .0385 |
|  |  | 2022 | -.0283 | .02401 | 1 | .239 | -.0753 | .0188 |
|  |  | 2023 | .0254 | .02005 | 1 | .204 | -.0138 | .0647 |
|  |  | 2025 | -.0746 | .02745 | 1 | .007 | -.1284 | -.0208 |
|  | 2025 | 2020 | .1273 | .04698 | 1 | .007 | .0352 | .2194 |
|  |  | 2021 | .0549 | .03471 | 1 | .114 | -.0132 | .1229 |
|  |  | 2022 | .0463 | .03003 | 1 | .123 | -.0125 | .1052 |
|  |  | 2023 | .1000 | .02705 | 1 | .000 | .0470 | .1530 |
|  |  | 2024 | .0746 | .02745 | 1 | .007 | .0208 | .1284 |
| 2 | 2020 | 2021 | -.0092 | .05829 | 1 | .875 | -.1234 | .1050 |
|  |  | 2022 | .0170 | .05268 | 1 | .746 | -.0862 | .1203 |
|  |  | 2023 | .0636 | .05124 | 1 | .215 | -.0368 | .1640 |
|  |  | 2024 | .0143 | .05158 | 1 | .782 | -.0868 | .1154 |
|  |  | 2025 | -.0140 | .05417 | 1 | .796 | -.1202 | .0922 |
|  | 2021 | 2020 | .0092 | .05829 | 1 | .875 | -.1050 | .1234 |
|  |  | 2022 | .0263 | .03640 | 1 | .471 | -.0451 | .0976 |
|  |  | 2023 | .0728 | .03446 | 1 | .035 | .0053 | .1403 |
|  |  | 2024 | .0235 | .03479 | 1 | .500 | -.0447 | .0917 |
|  |  | 2025 | -.0048 | .03877 | 1 | .901 | -.0808 | .0712 |
|  | 2022 | 2020 | -.0170 | .05268 | 1 | .746 | -.1203 | .0862 |
|  |  | 2021 | -.0263 | .03640 | 1 | .471 | -.0976 | .0451 |
|  |  | 2023 | .0465 | .02369 | 1 | .049 | .0001 | .0930 |
|  |  | 2024 | -.0028 | .02421 | 1 | .909 | -.0502 | .0447 |
|  |  | 2025 | -.0311 | .02964 | 1 | .294 | -.0892 | .0270 |
|  | 2023 | 2020 | -.0636 | .05124 | 1 | .215 | -.1640 | .0368 |
|  |  | 2021 | -.0728 | .03446 | 1 | .035 | -.1403 | -.0053 |
|  |  | 2022 | -.0465 | .02369 | 1 | .049 | -.0930 | -.0001 |
|  |  | 2024 | -.0493 | .02089 | 1 | .018 | -.0903 | -.0084 |
|  |  | 2025 | -.0776 | .02699 | 1 | .004 | -.1305 | -.0247 |
|  | 2024 | 2020 | -.0143 | .05158 | 1 | .782 | -.1154 | .0868 |
|  |  | 2021 | -.0235 | .03479 | 1 | .500 | -.0917 | .0447 |
|  |  | 2022 | .0028 | .02421 | 1 | .909 | -.0447 | .0502 |
|  |  | 2023 | .0493 | .02089 | 1 | .018 | .0084 | .0903 |
|  |  | 2025 | -.0283 | .02758 | 1 | .305 | -.0823 | .0258 |
|  | 2025 | 2020 | .0140 | .05417 | 1 | .796 | -.0922 | .1202 |
|  |  | 2021 | .0048 | .03877 | 1 | .901 | -.0712 | .0808 |
|  |  | 2022 | .0311 | .02964 | 1 | .294 | -.0270 | .0892 |
|  |  | 2023 | .0776 | .02699 | 1 | .004 | .0247 | .1305 |
|  |  | 2024 | .0283 | .02758 | 1 | .305 | -.0258 | .0823 |

| **Overall Test** | | | |
| --- | --- | --- | --- |
| Gender | Wald χ² | Degrees of Freedom | P |
| 1 | 17.722 | 5 | .003 |
| 2 | 12.290 | 5 | .031 |

**Estimated Marginal Means 6：Year* Gender**

| **Estimate** | | | | | |
| --- | --- | --- | --- | --- | --- |
| Year | Gender | Mean | Standard Error | 95% Wald Confidence Interval | |
|  |  |  |  | Lower Bound | Upper Bound |
| 2020 | 1 | .6573 | .04098 | .5817 | .7427 |
|  | 2 | .6712 | .04928 | .5813 | .7751 |
| 2021 | 1 | .7298 | .02593 | .6807 | .7824 |
|  | 2 | .6804 | .03180 | .6209 | .7457 |
| 2022 | 1 | .7383 | .01957 | .7009 | .7777 |
|  | 2 | .6542 | .01954 | .6170 | .6936 |
| 2023 | 1 | .6846 | .01410 | .6575 | .7128 |
|  | 2 | .6076 | .01450 | .5799 | .6367 |
| 2024 | 1 | .7101 | .01496 | .6813 | .7400 |
|  | 2 | .6570 | .01591 | .6265 | .6889 |
| 2025 | 1 | .7846 | .02348 | .7399 | .8320 |
|  | 2 | .6852 | .02318 | .6413 | .7322 |

| **Pairwise Comparisons** | | | | | | | | |
| --- | --- | --- | --- | --- | --- | --- | --- | --- |
| Year | (I) Gender | (J) Gender | Mean Difference (I-J) | Standard Error | Degrees of Freedom | P | 95% Wald Confidence Interval | |
|  |  |  |  |  |  |  | Lower Bound | Upper Bound |
| 2020 | 1 | 2 | -.0139 | .05575 | 1 | .803 | -.1232 | .0953 |
|  | 2 | 1 | .0139 | .05575 | 1 | .803 | -.0953 | .1232 |
| 2021 | 1 | 2 | .0493 | .03696 | 1 | .182 | -.0231 | .1218 |
|  | 2 | 1 | -.0493 | .03696 | 1 | .182 | -.1218 | .0231 |
| 2022 | 1 | 2 | .0841 | .02262 | 1 | .000 | .0398 | .1285 |
|  | 2 | 1 | -.0841 | .02262 | 1 | .000 | -.1285 | -.0398 |
| 2023 | 1 | 2 | .0770 | .01918 | 1 | .000 | .0394 | .1146 |
|  | 2 | 1 | -.0770 | .01918 | 1 | .000 | -.1146 | -.0394 |
| 2024 | 1 | 2 | .0531 | .01977 | 1 | .007 | .0144 | .0919 |
|  | 2 | 1 | -.0531 | .01977 | 1 | .007 | -.0919 | -.0144 |
| 2025 | 1 | 2 | .0994 | .03015 | 1 | .001 | .0403 | .1585 |
|  | 2 | 1 | -.0994 | .03015 | 1 | .001 | -.1585 | -.0403 |

| **Overall Test** | | | |
| --- | --- | --- | --- |
| Year | Wald χ² | Degrees of Freedom | P |
| 2020 | .062 | 1 | .803 |
| 2021 | 1.782 | 1 | .182 |
| 2022 | 13.841 | 1 | .000 |
| 2023 | 16.114 | 1 | .000 |
| 2024 | 7.216 | 1 | .007 |
| 2025 | 10.869 | 1 | .001 |

**Estimated Marginal Means 7：Year* Age**

| **Estimate** | | | | | |
| --- | --- | --- | --- | --- | --- |
| Year | Age | Mean | Standard Error | 95% Wald Confidence Interval | |
|  |  |  |  | Lower Bound | Upper Bound |
| 2020 | 1 | .1902 | .01309 | .1662 | .2177 |
|  | 2 | .5393 | .03441 | .4759 | .6111 |
|  | 3 | 1.1814 | .17534 | .8832 | 1.5802 |
|  | 4 | 1.6060 | .19562 | 1.2649 | 2.0390 |
| 2021 | 1 | .2023 | .01019 | .1833 | .2233 |
|  | 2 | .6402 | .02614 | .5910 | .6935 |
|  | 3 | 1.2140 | .07053 | 1.0833 | 1.3604 |
|  | 4 | 1.5684 | .13190 | 1.3301 | 1.8495 |
| 2022 | 1 | .2085 | .00651 | .1961 | .2216 |
|  | 2 | .5979 | .01442 | .5703 | .6269 |
|  | 3 | 1.1186 | .04067 | 1.0417 | 1.2013 |
|  | 4 | 1.6728 | .11669 | 1.4591 | 1.9179 |
| 2023 | 1 | .1786 | .00567 | .1678 | .1901 |
|  | 2 | .5337 | .01268 | .5094 | .5591 |
|  | 3 | 1.1379 | .03566 | 1.0701 | 1.2099 |
|  | 4 | 1.5956 | .05913 | 1.4838 | 1.7158 |
| 2024 | 1 | .1954 | .00559 | .1848 | .2067 |
|  | 2 | .5433 | .01317 | .5181 | .5697 |
|  | 3 | 1.1523 | .03737 | 1.0813 | 1.2279 |
|  | 4 | 1.7784 | .07048 | 1.6455 | 1.9221 |
| 2025 | 1 | .2361 | .00887 | .2193 | .2541 |
|  | 2 | .6040 | .01858 | .5687 | .6415 |
|  | 3 | 1.1677 | .05836 | 1.0588 | 1.2879 |
|  | 4 | 1.7363 | .11430 | 1.5261 | 1.9754 |

| **Pairwise Comparisons** | | | | | | | | |
| --- | --- | --- | --- | --- | --- | --- | --- | --- |
| Age | (I) Year | (J) Year | Mean Difference (I-J) | Standard Error | Degrees of Freedom | P | 95% Wald Confidence Interval | |
|  |  |  |  |  |  |  | Lower Bound | Upper Bound |
| 1 | 2020 | 2021 | -.0120 | .01637 | 1 | .462 | -.0441 | .0201 |
|  |  | 2022 | -.0182 | .01436 | 1 | .204 | -.0464 | .0099 |
|  |  | 2023 | .0116 | .01408 | 1 | .409 | -.0160 | .0392 |
|  |  | 2024 | -.0052 | .01401 | 1 | .710 | -.0327 | .0223 |
|  |  | 2025 | -.0458 | .01563 | 1 | .003 | -.0765 | -.0152 |
|  | 2021 | 2020 | .0120 | .01637 | 1 | .462 | -.0201 | .0441 |
|  |  | 2022 | -.0062 | .01156 | 1 | .591 | -.0289 | .0165 |
|  |  | 2023 | .0237 | .01131 | 1 | .036 | .0015 | .0458 |
|  |  | 2024 | .0068 | .01117 | 1 | .541 | -.0151 | .0287 |
|  |  | 2025 | -.0338 | .01317 | 1 | .010 | -.0596 | -.0080 |
|  | 2022 | 2020 | .0182 | .01436 | 1 | .204 | -.0099 | .0464 |
|  |  | 2021 | .0062 | .01156 | 1 | .591 | -.0165 | .0289 |
|  |  | 2023 | .0299 | .00810 | 1 | .000 | .0140 | .0458 |
|  |  | 2024 | .0130 | .00790 | 1 | .099 | -.0024 | .0285 |
|  |  | 2025 | -.0276 | .01054 | 1 | .009 | -.0482 | -.0069 |
|  | 2023 | 2020 | -.0116 | .01408 | 1 | .409 | -.0392 | .0160 |
|  |  | 2021 | -.0237 | .01131 | 1 | .036 | -.0458 | -.0015 |
|  |  | 2022 | -.0299 | .00810 | 1 | .000 | -.0458 | -.0140 |
|  |  | 2024 | -.0168 | .00748 | 1 | .024 | -.0315 | -.0022 |
|  |  | 2025 | -.0575 | .01020 | 1 | .000 | -.0774 | -.0375 |
|  | 2024 | 2020 | .0052 | .01401 | 1 | .710 | -.0223 | .0327 |
|  |  | 2021 | -.0068 | .01117 | 1 | .541 | -.0287 | .0151 |
|  |  | 2022 | -.0130 | .00790 | 1 | .099 | -.0285 | .0024 |
|  |  | 2023 | .0168 | .00748 | 1 | .024 | .0022 | .0315 |
|  |  | 2025 | -.0406 | .01008 | 1 | .000 | -.0604 | -.0209 |
|  | 2025 | 2020 | .0458 | .01563 | 1 | .003 | .0152 | .0765 |
|  |  | 2021 | .0338 | .01317 | 1 | .010 | .0080 | .0596 |
|  |  | 2022 | .0276 | .01054 | 1 | .009 | .0069 | .0482 |
|  |  | 2023 | .0575 | .01020 | 1 | .000 | .0375 | .0774 |
|  |  | 2024 | .0406 | .01008 | 1 | .000 | .0209 | .0604 |
| 2 | 2020 | 2021 | -.1009 | .04318 | 1 | .019 | -.1855 | -.0162 |
|  |  | 2022 | -.0586 | .03727 | 1 | .116 | -.1317 | .0144 |
|  |  | 2023 | .0057 | .03664 | 1 | .877 | -.0661 | .0775 |
|  |  | 2024 | -.0040 | .03680 | 1 | .914 | -.0761 | .0682 |
|  |  | 2025 | -.0647 | .03908 | 1 | .098 | -.1413 | .0119 |
|  | 2021 | 2020 | .1009 | .04318 | 1 | .019 | .0162 | .1855 |
|  |  | 2022 | .0423 | .02967 | 1 | .154 | -.0159 | .1004 |
|  |  | 2023 | .1066 | .02890 | 1 | .000 | .0499 | .1632 |
|  |  | 2024 | .0969 | .02907 | 1 | .001 | .0400 | .1539 |
|  |  | 2025 | .0362 | .03195 | 1 | .257 | -.0264 | .0988 |
|  | 2022 | 2020 | .0586 | .03727 | 1 | .116 | -.0144 | .1317 |
|  |  | 2021 | -.0423 | .02967 | 1 | .154 | -.1004 | .0159 |
|  |  | 2023 | .0643 | .01896 | 1 | .001 | .0271 | .1014 |
|  |  | 2024 | .0547 | .01920 | 1 | .004 | .0170 | .0923 |
|  |  | 2025 | -.0061 | .02335 | 1 | .795 | -.0518 | .0397 |
|  | 2023 | 2020 | -.0057 | .03664 | 1 | .877 | -.0775 | .0661 |
|  |  | 2021 | -.1066 | .02890 | 1 | .000 | -.1632 | -.0499 |
|  |  | 2022 | -.0643 | .01896 | 1 | .001 | -.1014 | -.0271 |
|  |  | 2024 | -.0096 | .01797 | 1 | .592 | -.0449 | .0256 |
|  |  | 2025 | -.0703 | .02234 | 1 | .002 | -.1141 | -.0266 |
|  | 2024 | 2020 | .0040 | .03680 | 1 | .914 | -.0682 | .0761 |
|  |  | 2021 | -.0969 | .02907 | 1 | .001 | -.1539 | -.0400 |
|  |  | 2022 | -.0547 | .01920 | 1 | .004 | -.0923 | -.0170 |
|  |  | 2023 | .0096 | .01797 | 1 | .592 | -.0256 | .0449 |
|  |  | 2025 | -.0607 | .02257 | 1 | .007 | -.1050 | -.0165 |
|  | 2025 | 2020 | .0647 | .03908 | 1 | .098 | -.0119 | .1413 |
|  |  | 2021 | -.0362 | .03195 | 1 | .257 | -.0988 | .0264 |
|  |  | 2022 | .0061 | .02335 | 1 | .795 | -.0397 | .0518 |
|  |  | 2023 | .0703 | .02234 | 1 | .002 | .0266 | .1141 |
|  |  | 2024 | .0607 | .02257 | 1 | .007 | .0165 | .1050 |
| 3 | 2020 | 2021 | -.0326 | .18871 | 1 | .863 | -.4025 | .3372 |
|  |  | 2022 | .0627 | .17964 | 1 | .727 | -.2894 | .4148 |
|  |  | 2023 | .0435 | .17860 | 1 | .808 | -.3065 | .3935 |
|  |  | 2024 | .0291 | .17877 | 1 | .871 | -.3213 | .3794 |
|  |  | 2025 | .0136 | .18461 | 1 | .941 | -.3482 | .3755 |
|  | 2021 | 2020 | .0326 | .18871 | 1 | .863 | -.3372 | .4025 |
|  |  | 2022 | .0953 | .08094 | 1 | .239 | -.0633 | .2540 |
|  |  | 2023 | .0761 | .07852 | 1 | .332 | -.0778 | .2300 |
|  |  | 2024 | .0617 | .07915 | 1 | .436 | -.0934 | .2168 |
|  |  | 2025 | .0463 | .09133 | 1 | .613 | -.1328 | .2253 |
|  | 2022 | 2020 | -.0627 | .17964 | 1 | .727 | -.4148 | .2894 |
|  |  | 2021 | -.0953 | .08094 | 1 | .239 | -.2540 | .0633 |
|  |  | 2023 | -.0192 | .05316 | 1 | .718 | -.1234 | .0850 |
|  |  | 2024 | -.0337 | .05384 | 1 | .532 | -.1392 | .0719 |
|  |  | 2025 | -.0491 | .07066 | 1 | .487 | -.1876 | .0894 |
|  | 2023 | 2020 | -.0435 | .17860 | 1 | .808 | -.3935 | .3065 |
|  |  | 2021 | -.0761 | .07852 | 1 | .332 | -.2300 | .0778 |
|  |  | 2022 | .0192 | .05316 | 1 | .718 | -.0850 | .1234 |
|  |  | 2024 | -.0144 | .05030 | 1 | .774 | -.1130 | .0841 |
|  |  | 2025 | -.0299 | .06796 | 1 | .660 | -.1631 | .1033 |
|  | 2024 | 2020 | -.0291 | .17877 | 1 | .871 | -.3794 | .3213 |
|  |  | 2021 | -.0617 | .07915 | 1 | .436 | -.2168 | .0934 |
|  |  | 2022 | .0337 | .05384 | 1 | .532 | -.0719 | .1392 |
|  |  | 2023 | .0144 | .05030 | 1 | .774 | -.0841 | .1130 |
|  |  | 2025 | -.0154 | .06864 | 1 | .822 | -.1500 | .1191 |
|  | 2025 | 2020 | -.0136 | .18461 | 1 | .941 | -.3755 | .3482 |
|  |  | 2021 | -.0463 | .09133 | 1 | .613 | -.2253 | .1328 |
|  |  | 2022 | .0491 | .07066 | 1 | .487 | -.0894 | .1876 |
|  |  | 2023 | .0299 | .06796 | 1 | .660 | -.1033 | .1631 |
|  |  | 2024 | .0154 | .06864 | 1 | .822 | -.1191 | .1500 |
| 4 | 2020 | 2021 | .0376 | .23593 | 1 | .873 | -.4248 | .5000 |
|  |  | 2022 | -.0668 | .22823 | 1 | .770 | -.5142 | .3805 |
|  |  | 2023 | .0104 | .20529 | 1 | .960 | -.3920 | .4127 |
|  |  | 2024 | -.1724 | .20917 | 1 | .410 | -.5824 | .2375 |
|  |  | 2025 | -.1303 | .22635 | 1 | .565 | -.5739 | .3133 |
|  | 2021 | 2020 | -.0376 | .23593 | 1 | .873 | -.5000 | .4248 |
|  |  | 2022 | -.1044 | .17548 | 1 | .552 | -.4484 | .2395 |
|  |  | 2023 | -.0272 | .14378 | 1 | .850 | -.3090 | .2546 |
|  |  | 2024 | -.2100 | .14839 | 1 | .157 | -.5009 | .0808 |
|  |  | 2025 | -.1679 | .17422 | 1 | .335 | -.5093 | .1736 |
|  | 2022 | 2020 | .0668 | .22823 | 1 | .770 | -.3805 | .5142 |
|  |  | 2021 | .1044 | .17548 | 1 | .552 | -.2395 | .4484 |
|  |  | 2023 | .0772 | .12947 | 1 | .551 | -.1765 | .3310 |
|  |  | 2024 | -.1056 | .13445 | 1 | .432 | -.3691 | .1579 |
|  |  | 2025 | -.0635 | .16322 | 1 | .697 | -.3834 | .2564 |
|  | 2023 | 2020 | -.0104 | .20529 | 1 | .960 | -.4127 | .3920 |
|  |  | 2021 | .0272 | .14378 | 1 | .850 | -.2546 | .3090 |
|  |  | 2022 | -.0772 | .12947 | 1 | .551 | -.3310 | .1765 |
|  |  | 2024 | -.1828 | .08799 | 1 | .038 | -.3553 | -.0104 |
|  |  | 2025 | -.1407 | .12867 | 1 | .274 | -.3929 | .1115 |
|  | 2024 | 2020 | .1724 | .20917 | 1 | .410 | -.2375 | .5824 |
|  |  | 2021 | .2100 | .14839 | 1 | .157 | -.0808 | .5009 |
|  |  | 2022 | .1056 | .13445 | 1 | .432 | -.1579 | .3691 |
|  |  | 2023 | .1828 | .08799 | 1 | .038 | .0104 | .3553 |
|  |  | 2025 | .0421 | .13419 | 1 | .754 | -.2209 | .3051 |
|  | 2025 | 2020 | .1303 | .22635 | 1 | .565 | -.3133 | .5739 |
|  |  | 2021 | .1679 | .17422 | 1 | .335 | -.1736 | .5093 |
|  |  | 2022 | .0635 | .16322 | 1 | .697 | -.2564 | .3834 |
|  |  | 2023 | .1407 | .12867 | 1 | .274 | -.1115 | .3929 |
|  |  | 2024 | -.0421 | .13419 | 1 | .754 | -.3051 | .2209 |

| **Overall Test** | | | |
| --- | --- | --- | --- |
| Age | Wald χ² | Degrees of Freedom | P |
| 1 | 36.008 | 5 | .000 |
| 2 | 27.730 | 5 | .000 |
| 3 | 1.666 | 5 | .893 |
| 4 | 5.359 | 5 | .374 |

**Estimated Marginal Means 8：Year* Age**

| **Estimate** | | | | | |
| --- | --- | --- | --- | --- | --- |
| Year | Age | Mean | Standard Error | 95% Wald Confidence Interval | |
|  |  |  |  | Lower Bound | Upper Bound |
| 2020 | 1 | .1902 | .01309 | .1662 | .2177 |
|  | 2 | .5393 | .03441 | .4759 | .6111 |
|  | 3 | 1.1814 | .17534 | .8832 | 1.5802 |
|  | 4 | 1.6060 | .19562 | 1.2649 | 2.0390 |
| 2021 | 1 | .2023 | .01019 | .1833 | .2233 |
|  | 2 | .6402 | .02614 | .5910 | .6935 |
|  | 3 | 1.2140 | .07053 | 1.0833 | 1.3604 |
|  | 4 | 1.5684 | .13190 | 1.3301 | 1.8495 |
| 2022 | 1 | .2085 | .00651 | .1961 | .2216 |
|  | 2 | .5979 | .01442 | .5703 | .6269 |
|  | 3 | 1.1186 | .04067 | 1.0417 | 1.2013 |
|  | 4 | 1.6728 | .11669 | 1.4591 | 1.9179 |
| 2023 | 1 | .1786 | .00567 | .1678 | .1901 |
|  | 2 | .5337 | .01268 | .5094 | .5591 |
|  | 3 | 1.1379 | .03566 | 1.0701 | 1.2099 |
|  | 4 | 1.5956 | .05913 | 1.4838 | 1.7158 |
| 2024 | 1 | .1954 | .00559 | .1848 | .2067 |
|  | 2 | .5433 | .01317 | .5181 | .5697 |
|  | 3 | 1.1523 | .03737 | 1.0813 | 1.2279 |
|  | 4 | 1.7784 | .07048 | 1.6455 | 1.9221 |
| 2025 | 1 | .2361 | .00887 | .2193 | .2541 |
|  | 2 | .6040 | .01858 | .5687 | .6415 |
|  | 3 | 1.1677 | .05836 | 1.0588 | 1.2879 |
|  | 4 | 1.7363 | .11430 | 1.5261 | 1.9754 |

| **Pairwise Comparisons** | | | | | | | | |
| --- | --- | --- | --- | --- | --- | --- | --- | --- |
| Year | (I) Age | (J) Age | Mean Difference (I-J) | Standard Error | Degrees of Freedom | P | 95% Wald Confidence Interval | |
|  |  |  |  |  |  |  | Lower Bound | Upper Bound |
| 2020 | 1 | 2 | -.3491 | .03651 | 1 | .000 | -.4206 | -.2775 |
|  |  | 3 | -.9911 | .17516 | 1 | .000 | -1.3344 | -.6478 |
|  |  | 4 | -1.4158 | .19641 | 1 | .000 | -1.8007 | -1.0308 |
|  | 2 | 1 | .3491 | .03651 | 1 | .000 | .2775 | .4206 |
|  |  | 3 | -.6420 | .17841 | 1 | .000 | -.9917 | -.2924 |
|  |  | 4 | -1.0667 | .19896 | 1 | .000 | -1.4566 | -.6767 |
|  | 3 | 1 | .9911 | .17516 | 1 | .000 | .6478 | 1.3344 |
|  |  | 2 | .6420 | .17841 | 1 | .000 | .2924 | .9917 |
|  |  | 4 | -.4246 | .26378 | 1 | .107 | -.9416 | .0924 |
|  | 4 | 1 | 1.4158 | .19641 | 1 | .000 | 1.0308 | 1.8007 |
|  |  | 2 | 1.0667 | .19896 | 1 | .000 | .6767 | 1.4566 |
|  |  | 3 | .4246 | .26378 | 1 | .107 | -.0924 | .9416 |
| 2021 | 1 | 2 | -.4379 | .02697 | 1 | .000 | -.4908 | -.3851 |
|  |  | 3 | -1.0117 | .07064 | 1 | .000 | -1.1502 | -.8732 |
|  |  | 4 | -1.3662 | .13193 | 1 | .000 | -1.6247 | -1.1076 |
|  | 2 | 1 | .4379 | .02697 | 1 | .000 | .3851 | .4908 |
|  |  | 3 | -.5738 | .07414 | 1 | .000 | -.7191 | -.4285 |
|  |  | 4 | -.9282 | .13380 | 1 | .000 | -1.1905 | -.6660 |
|  | 3 | 1 | 1.0117 | .07064 | 1 | .000 | .8732 | 1.1502 |
|  |  | 2 | .5738 | .07414 | 1 | .000 | .4285 | .7191 |
|  |  | 4 | -.3544 | .14840 | 1 | .017 | -.6453 | -.0636 |
|  | 4 | 1 | 1.3662 | .13193 | 1 | .000 | 1.1076 | 1.6247 |
|  |  | 2 | .9282 | .13380 | 1 | .000 | .6660 | 1.1905 |
|  |  | 3 | .3544 | .14840 | 1 | .017 | .0636 | .6453 |
| 2022 | 1 | 2 | -.3894 | .01504 | 1 | .000 | -.4189 | -.3600 |
|  |  | 3 | -.9101 | .04068 | 1 | .000 | -.9899 | -.8304 |
|  |  | 4 | -1.4644 | .11666 | 1 | .000 | -1.6930 | -1.2357 |
|  | 2 | 1 | .3894 | .01504 | 1 | .000 | .3600 | .4189 |
|  |  | 3 | -.5207 | .04241 | 1 | .000 | -.6038 | -.4376 |
|  |  | 4 | -1.0749 | .11726 | 1 | .000 | -1.3047 | -.8451 |
|  | 3 | 1 | .9101 | .04068 | 1 | .000 | .8304 | .9899 |
|  |  | 2 | .5207 | .04241 | 1 | .000 | .4376 | .6038 |
|  |  | 4 | -.5542 | .12307 | 1 | .000 | -.7954 | -.3130 |
|  | 4 | 1 | 1.4644 | .11666 | 1 | .000 | 1.2357 | 1.6930 |
|  |  | 2 | 1.0749 | .11726 | 1 | .000 | .8451 | 1.3047 |
|  |  | 3 | .5542 | .12307 | 1 | .000 | .3130 | .7954 |
| 2023 | 1 | 2 | -.3550 | .01354 | 1 | .000 | -.3816 | -.3285 |
|  |  | 3 | -.9592 | .03587 | 1 | .000 | -1.0295 | -.8889 |
|  |  | 4 | -1.4170 | .05918 | 1 | .000 | -1.5330 | -1.3010 |
|  | 2 | 1 | .3550 | .01354 | 1 | .000 | .3285 | .3816 |
|  |  | 3 | -.6042 | .03743 | 1 | .000 | -.6776 | -.5308 |
|  |  | 4 | -1.0620 | .06008 | 1 | .000 | -1.1797 | -.9442 |
|  | 3 | 1 | .9592 | .03587 | 1 | .000 | .8889 | 1.0295 |
|  |  | 2 | .6042 | .03743 | 1 | .000 | .5308 | .6776 |
|  |  | 4 | -.4578 | .06837 | 1 | .000 | -.5918 | -.3238 |
|  | 4 | 1 | 1.4170 | .05918 | 1 | .000 | 1.3010 | 1.5330 |
|  |  | 2 | 1.0620 | .06008 | 1 | .000 | .9442 | 1.1797 |
|  |  | 3 | .4578 | .06837 | 1 | .000 | .3238 | .5918 |
| 2024 | 1 | 2 | -.3478 | .01371 | 1 | .000 | -.3747 | -.3210 |
|  |  | 3 | -.9569 | .03730 | 1 | .000 | -1.0300 | -.8837 |
|  |  | 4 | -1.5830 | .07030 | 1 | .000 | -1.7208 | -1.4452 |
|  | 2 | 1 | .3478 | .01371 | 1 | .000 | .3210 | .3747 |
|  |  | 3 | -.6090 | .03871 | 1 | .000 | -.6849 | -.5332 |
|  |  | 4 | -1.2352 | .07093 | 1 | .000 | -1.3742 | -1.0961 |
|  | 3 | 1 | .9569 | .03730 | 1 | .000 | .8837 | 1.0300 |
|  |  | 2 | .6090 | .03871 | 1 | .000 | .5332 | .6849 |
|  |  | 4 | -.6261 | .07822 | 1 | .000 | -.7794 | -.4728 |
|  | 4 | 1 | 1.5830 | .07030 | 1 | .000 | 1.4452 | 1.7208 |
|  |  | 2 | 1.2352 | .07093 | 1 | .000 | 1.0961 | 1.3742 |
|  |  | 3 | .6261 | .07822 | 1 | .000 | .4728 | .7794 |
| 2025 | 1 | 2 | -.3679 | .02010 | 1 | .000 | -.4073 | -.3285 |
|  |  | 3 | -.9317 | .05881 | 1 | .000 | -1.0469 | -.8164 |
|  |  | 4 | -1.5002 | .11461 | 1 | .000 | -1.7249 | -1.2756 |
|  | 2 | 1 | .3679 | .02010 | 1 | .000 | .3285 | .4073 |
|  |  | 3 | -.5637 | .06097 | 1 | .000 | -.6832 | -.4442 |
|  |  | 4 | -1.1323 | .11574 | 1 | .000 | -1.3592 | -.9055 |
|  | 3 | 1 | .9317 | .05881 | 1 | .000 | .8164 | 1.0469 |
|  |  | 2 | .5637 | .06097 | 1 | .000 | .4442 | .6832 |
|  |  | 4 | -.5686 | .12838 | 1 | .000 | -.8202 | -.3170 |
|  | 4 | 1 | 1.5002 | .11461 | 1 | .000 | 1.2756 | 1.7249 |
|  |  | 2 | 1.1323 | .11574 | 1 | .000 | .9055 | 1.3592 |
|  |  | 3 | .5686 | .12838 | 1 | .000 | .3170 | .8202 |

| **Overall Test** | | | |
| --- | --- | --- | --- |
| Year | Wald χ² | Degrees of Freedom | P |
| 2020 | 171.172 | 3 | .000 |
| 2021 | 535.279 | 3 | .000 |
| 2022 | 1234.207 | 3 | .000 |
| 2023 | 1799.993 | 3 | .000 |
| 2024 | 1619.903 | 3 | .000 |
| 2025 | 707.091 | 3 | .000 |

**Estimated Marginal Means 9：Year* Diagnostic**

| **Estimate** | | | | | |
| --- | --- | --- | --- | --- | --- |
| Year | Diagnostic | Mean | Standard Error | 95% Wald Confidence Interval | |
|  |  |  |  | Lower Bound | Upper Bound |
| 2020 | 1 | .6873 | .05055 | .5950 | .7939 |
|  | 2 | .6419 | .04174 | .5651 | .7292 |
| 2021 | 1 | .7622 | .03769 | .6918 | .8398 |
|  | 2 | .6514 | .02287 | .6081 | .6978 |
| 2022 | 1 | .6815 | .02372 | .6366 | .7297 |
|  | 2 | .7087 | .01670 | .6767 | .7422 |
| 2023 | 1 | .6412 | .01761 | .6076 | .6767 |
|  | 2 | .6488 | .01129 | .6270 | .6713 |
| 2024 | 1 | .6669 | .02015 | .6285 | .7076 |
|  | 2 | .6995 | .01123 | .6778 | .7219 |
| 2025 | 1 | .7656 | .02746 | .7136 | .8213 |
|  | 2 | .7023 | .02030 | .6636 | .7432 |

| **Pairwise Comparisons** | | | | | | | | |
| --- | --- | --- | --- | --- | --- | --- | --- | --- |
| Diagnostic | (I) Year | (J) Year | Mean Difference (I-J) | Standard Error | Degrees of Freedom | P | 95% Wald Confidence Interval | |
|  |  |  |  |  |  |  | Lower Bound | Upper Bound |
| 1 | 2020 | 2021 | -.0750 | .06288 | 1 | .233 | -.1982 | .0483 |
|  |  | 2022 | .0057 | .05563 | 1 | .918 | -.1033 | .1148 |
|  |  | 2023 | .0461 | .05341 | 1 | .388 | -.0586 | .1508 |
|  |  | 2024 | .0204 | .05425 | 1 | .707 | -.0859 | .1267 |
|  |  | 2025 | -.0783 | .05733 | 1 | .172 | -.1906 | .0341 |
|  | 2021 | 2020 | .0750 | .06288 | 1 | .233 | -.0483 | .1982 |
|  |  | 2022 | .0807 | .04381 | 1 | .065 | -.0052 | .1666 |
|  |  | 2023 | .1210 | .04113 | 1 | .003 | .0404 | .2017 |
|  |  | 2024 | .0954 | .04222 | 1 | .024 | .0126 | .1781 |
|  |  | 2025 | -.0033 | .04620 | 1 | .943 | -.0939 | .0872 |
|  | 2022 | 2020 | -.0057 | .05563 | 1 | .918 | -.1148 | .1033 |
|  |  | 2021 | -.0807 | .04381 | 1 | .065 | -.1666 | .0052 |
|  |  | 2023 | .0403 | .02889 | 1 | .163 | -.0163 | .0970 |
|  |  | 2024 | .0147 | .03040 | 1 | .629 | -.0449 | .0742 |
|  |  | 2025 | -.0840 | .03571 | 1 | .019 | -.1540 | -.0140 |
|  | 2023 | 2020 | -.0461 | .05341 | 1 | .388 | -.1508 | .0586 |
|  |  | 2021 | -.1210 | .04113 | 1 | .003 | -.2017 | -.0404 |
|  |  | 2022 | -.0403 | .02889 | 1 | .163 | -.0970 | .0163 |
|  |  | 2024 | -.0257 | .02626 | 1 | .328 | -.0771 | .0258 |
|  |  | 2025 | -.1244 | .03225 | 1 | .000 | -.1876 | -.0611 |
|  | 2024 | 2020 | -.0204 | .05425 | 1 | .707 | -.1267 | .0859 |
|  |  | 2021 | -.0954 | .04222 | 1 | .024 | -.1781 | -.0126 |
|  |  | 2022 | -.0147 | .03040 | 1 | .629 | -.0742 | .0449 |
|  |  | 2023 | .0257 | .02626 | 1 | .328 | -.0258 | .0771 |
|  |  | 2025 | -.0987 | .03362 | 1 | .003 | -.1646 | -.0328 |
|  | 2025 | 2020 | .0783 | .05733 | 1 | .172 | -.0341 | .1906 |
|  |  | 2021 | .0033 | .04620 | 1 | .943 | -.0872 | .0939 |
|  |  | 2022 | .0840 | .03571 | 1 | .019 | .0140 | .1540 |
|  |  | 2023 | .1244 | .03225 | 1 | .000 | .0611 | .1876 |
|  |  | 2024 | .0987 | .03362 | 1 | .003 | .0328 | .1646 |
| 2 | 2020 | 2021 | -.0095 | .04733 | 1 | .841 | -.1023 | .0833 |
|  |  | 2022 | -.0667 | .04465 | 1 | .135 | -.1542 | .0208 |
|  |  | 2023 | -.0068 | .04315 | 1 | .874 | -.0914 | .0777 |
|  |  | 2024 | -.0576 | .04310 | 1 | .182 | -.1420 | .0269 |
|  |  | 2025 | -.0604 | .04605 | 1 | .190 | -.1506 | .0299 |
|  | 2021 | 2020 | .0095 | .04733 | 1 | .841 | -.0833 | .1023 |
|  |  | 2022 | -.0572 | .02804 | 1 | .041 | -.1122 | -.0023 |
|  |  | 2023 | .0027 | .02541 | 1 | .916 | -.0471 | .0525 |
|  |  | 2024 | -.0481 | .02537 | 1 | .058 | -.0978 | .0017 |
|  |  | 2025 | -.0509 | .03027 | 1 | .093 | -.1102 | .0085 |
|  | 2022 | 2020 | .0667 | .04465 | 1 | .135 | -.0208 | .1542 |
|  |  | 2021 | .0572 | .02804 | 1 | .041 | .0023 | .1122 |
|  |  | 2023 | .0599 | .02001 | 1 | .003 | .0207 | .0991 |
|  |  | 2024 | .0092 | .01997 | 1 | .646 | -.0300 | .0483 |
|  |  | 2025 | .0063 | .02587 | 1 | .806 | -.0444 | .0571 |
|  | 2023 | 2020 | .0068 | .04315 | 1 | .874 | -.0777 | .0914 |
|  |  | 2021 | -.0027 | .02541 | 1 | .916 | -.0525 | .0471 |
|  |  | 2022 | -.0599 | .02001 | 1 | .003 | -.0991 | -.0207 |
|  |  | 2024 | -.0507 | .01585 | 1 | .001 | -.0818 | -.0197 |
|  |  | 2025 | -.0535 | .02306 | 1 | .020 | -.0987 | -.0084 |
|  | 2024 | 2020 | .0576 | .04310 | 1 | .182 | -.0269 | .1420 |
|  |  | 2021 | .0481 | .02537 | 1 | .058 | -.0017 | .0978 |
|  |  | 2022 | -.0092 | .01997 | 1 | .646 | -.0483 | .0300 |
|  |  | 2023 | .0507 | .01585 | 1 | .001 | .0197 | .0818 |
|  |  | 2025 | -.0028 | .02303 | 1 | .902 | -.0480 | .0423 |
|  | 2025 | 2020 | .0604 | .04605 | 1 | .190 | -.0299 | .1506 |
|  |  | 2021 | .0509 | .03027 | 1 | .093 | -.0085 | .1102 |
|  |  | 2022 | -.0063 | .02587 | 1 | .806 | -.0571 | .0444 |
|  |  | 2023 | .0535 | .02306 | 1 | .020 | .0084 | .0987 |
|  |  | 2024 | .0028 | .02303 | 1 | .902 | -.0423 | .0480 |

| **Overall Test** | | | |
| --- | --- | --- | --- |
| Diagnostic | Wald χ² | Degrees of Freedom | P |
| 1 | 20.212 | 5 | .001 |
| 2 | 17.450 | 5 | .004 |

**Estimated Marginal Means 10：Year* Diagnostic**

| **Estimate** | | | | | |
| --- | --- | --- | --- | --- | --- |
| Year | Diagnostic | Mean | Standard Error | 95% Wald Confidence Interval | |
|  |  |  |  | Lower Bound | Upper Bound |
| 2020 | 1 | .6873 | .05055 | .5950 | .7939 |
|  | 2 | .6419 | .04174 | .5651 | .7292 |
| 2021 | 1 | .7622 | .03769 | .6918 | .8398 |
|  | 2 | .6514 | .02287 | .6081 | .6978 |
| 2022 | 1 | .6815 | .02372 | .6366 | .7297 |
|  | 2 | .7087 | .01670 | .6767 | .7422 |
| 2023 | 1 | .6412 | .01761 | .6076 | .6767 |
|  | 2 | .6488 | .01129 | .6270 | .6713 |
| 2024 | 1 | .6669 | .02015 | .6285 | .7076 |
|  | 2 | .6995 | .01123 | .6778 | .7219 |
| 2025 | 1 | .7656 | .02746 | .7136 | .8213 |
|  | 2 | .7023 | .02030 | .6636 | .7432 |

| **Pairwise Comparisons** | | | | | | | | |
| --- | --- | --- | --- | --- | --- | --- | --- | --- |
| Year | (I) Diagnostic | (J) Diagnostic | Mean Difference (I-J) | Standard Error | Degrees of Freedom | P | 95% Wald Confidence Interval | |
|  |  |  |  |  |  |  | Lower Bound | Upper Bound |
| 2020 | 1 | 2 | .0453 | .05879 | 1 | .440 | -.0699 | .1606 |
|  | 2 | 1 | -.0453 | .05879 | 1 | .440 | -.1606 | .0699 |
| 2021 | 1 | 2 | .1108 | .04172 | 1 | .008 | .0290 | .1926 |
|  | 2 | 1 | -.1108 | .04172 | 1 | .008 | -.1926 | -.0290 |
| 2022 | 1 | 2 | -.0271 | .02590 | 1 | .295 | -.0779 | .0236 |
|  | 2 | 1 | .0271 | .02590 | 1 | .295 | -.0236 | .0779 |
| 2023 | 1 | 2 | -.0076 | .02056 | 1 | .713 | -.0479 | .0327 |
|  | 2 | 1 | .0076 | .02056 | 1 | .713 | -.0327 | .0479 |
| 2024 | 1 | 2 | -.0326 | .02262 | 1 | .149 | -.0769 | .0117 |
|  | 2 | 1 | .0326 | .02262 | 1 | .149 | -.0117 | .0769 |
| 2025 | 1 | 2 | .0632 | .03210 | 1 | .049 | .0003 | .1261 |
|  | 2 | 1 | -.0632 | .03210 | 1 | .049 | -.1261 | -.0003 |

| **Overall Test** | | | |
| --- | --- | --- | --- |
| Year | Wald χ² | Degrees of Freedom | P |
| 2020 | .595 | 1 | .440 |
| 2021 | 7.053 | 1 | .008 |
| 2022 | 1.097 | 1 | .295 |
| 2023 | .135 | 1 | .713 |
| 2024 | 2.079 | 1 | .149 |
| 2025 | 3.882 | 1 | .049 |

**Estimated Marginal Means 11：Gender* Age**

| **Estimate** | | | | | |
| --- | --- | --- | --- | --- | --- |
| Gender | Age | Mean | Standard Error | 95% Wald Confidence Interval | |
|  |  |  |  | Lower Bound | Upper Bound |
| 1 | 1 | .2145 | .00484 | .2052 | .2242 |
|  | 2 | .5936 | .01145 | .5716 | .6165 |
|  | 3 | 1.2063 | .04017 | 1.1301 | 1.2876 |
|  | 4 | 1.7142 | .06148 | 1.5979 | 1.8390 |
| 2 | 1 | .1885 | .00523 | .1785 | .1991 |
|  | 2 | .5570 | .01207 | .5339 | .5812 |
|  | 3 | 1.1185 | .04122 | 1.0406 | 1.2023 |
|  | 4 | 1.6033 | .06583 | 1.4794 | 1.7377 |

| **Pairwise Comparisons** | | | | | | | | |
| --- | --- | --- | --- | --- | --- | --- | --- | --- |
| Age | (I) Gender | (J) Gender | Mean Difference (I-J) | Standard Error | Degrees of Freedom | P | 95% Wald Confidence Interval | |
|  |  |  |  |  |  |  | Lower Bound | Upper Bound |
| 1 | 1 | 2 | .0260 | .00609 | 1 | .000 | .0140 | .0379 |
|  | 2 | 1 | -.0260 | .00609 | 1 | .000 | -.0379 | -.0140 |
| 2 | 1 | 2 | .0366 | .01530 | 1 | .017 | .0066 | .0665 |
|  | 2 | 1 | -.0366 | .01530 | 1 | .017 | -.0665 | -.0066 |
| 3 | 1 | 2 | .0878 | .04225 | 1 | .038 | .0050 | .1706 |
|  | 2 | 1 | -.0878 | .04225 | 1 | .038 | -.1706 | -.0050 |
| 4 | 1 | 2 | .1109 | .07559 | 1 | .142 | -.0373 | .2591 |
|  | 2 | 1 | -.1109 | .07559 | 1 | .142 | -.2591 | .0373 |

| **Overall Test** | | | |
| --- | --- | --- | --- |
| Age | Wald χ² | Degrees of Freedom | P |
| 1 | 18.165 | 1 | .000 |
| 2 | 5.709 | 1 | .017 |
| 3 | 4.317 | 1 | .038 |
| 4 | 2.152 | 1 | .142 |

**Estimated Marginal Means 12：Gender* Age**

| **Estimate** | | | | | |
| --- | --- | --- | --- | --- | --- |
| Gender | Age | Mean | Standard Error | 95% Wald Confidence Interval | |
|  |  |  |  | Lower Bound | Upper Bound |
| 1 | 1 | .2145 | .00484 | .2052 | .2242 |
|  | 2 | .5936 | .01145 | .5716 | .6165 |
|  | 3 | 1.2063 | .04017 | 1.1301 | 1.2876 |
|  | 4 | 1.7142 | .06148 | 1.5979 | 1.8390 |
| 2 | 1 | .1885 | .00523 | .1785 | .1991 |
|  | 2 | .5570 | .01207 | .5339 | .5812 |
|  | 3 | 1.1185 | .04122 | 1.0406 | 1.2023 |
|  | 4 | 1.6033 | .06583 | 1.4794 | 1.7377 |

| **Pairwise Comparisons** | | | | | | | | |
| --- | --- | --- | --- | --- | --- | --- | --- | --- |
| Gender | (I) Age | (J) Age | Mean Difference (I-J) | Standard Error | Degrees of Freedom | P | 95% Wald Confidence Interval | |
|  |  |  |  |  |  |  | Lower Bound | Upper Bound |
| 1 | 1 | 2 | -.3791 | .01209 | 1 | .000 | -.4028 | -.3554 |
|  |  | 3 | -.9918 | .04024 | 1 | .000 | -1.0707 | -.9129 |
|  |  | 4 | -1.4997 | .06156 | 1 | .000 | -1.6204 | -1.3791 |
|  | 2 | 1 | .3791 | .01209 | 1 | .000 | .3554 | .4028 |
|  |  | 3 | -.6127 | .04110 | 1 | .000 | -.6932 | -.5322 |
|  |  | 4 | -1.1206 | .06198 | 1 | .000 | -1.2421 | -.9991 |
|  | 3 | 1 | .9918 | .04024 | 1 | .000 | .9129 | 1.0707 |
|  |  | 2 | .6127 | .04110 | 1 | .000 | .5322 | .6932 |
|  |  | 4 | -.5079 | .07269 | 1 | .000 | -.6504 | -.3655 |
|  | 4 | 1 | 1.4997 | .06156 | 1 | .000 | 1.3791 | 1.6204 |
|  |  | 2 | 1.1206 | .06198 | 1 | .000 | .9991 | 1.2421 |
|  |  | 3 | .5079 | .07269 | 1 | .000 | .3655 | .6504 |
| 2 | 1 | 2 | -.3685 | .01248 | 1 | .000 | -.3930 | -.3441 |
|  |  | 3 | -.9300 | .04098 | 1 | .000 | -1.0103 | -.8497 |
|  |  | 4 | -1.4148 | .06562 | 1 | .000 | -1.5434 | -1.2862 |
|  | 2 | 1 | .3685 | .01248 | 1 | .000 | .3441 | .3930 |
|  |  | 3 | -.5615 | .04183 | 1 | .000 | -.6434 | -.4795 |
|  |  | 4 | -1.0463 | .06596 | 1 | .000 | -1.1756 | -.9170 |
|  | 3 | 1 | .9300 | .04098 | 1 | .000 | .8497 | 1.0103 |
|  |  | 2 | .5615 | .04183 | 1 | .000 | .4795 | .6434 |
|  |  | 4 | -.4848 | .07551 | 1 | .000 | -.6328 | -.3368 |
|  | 4 | 1 | 1.4148 | .06562 | 1 | .000 | 1.2862 | 1.5434 |
|  |  | 2 | 1.0463 | .06596 | 1 | .000 | .9170 | 1.1756 |
|  |  | 3 | .4848 | .07551 | 1 | .000 | .3368 | .6328 |

| **Overall Test** | | | |
| --- | --- | --- | --- |
| Gender | Wald χ² | Degrees of Freedom | P |
| 1 | 1961.172 | 3 | .000 |
| 2 | 1632.321 | 3 | .000 |

**Estimated Marginal Means 13：Gender* Diagnostic**

| **Estimate** | | | | | |
| --- | --- | --- | --- | --- | --- |
| Gender | Diagnostic | Mean | Standard Error | 95% Wald Confidence Interval | |
|  |  |  |  | Lower Bound | Upper Bound |
| 1 | 1 | .7384 | .01640 | .7069 | .7712 |
|  | 2 | .6949 | .01215 | .6715 | .7191 |
| 2 | 1 | .6622 | .01785 | .6281 | .6981 |
|  | 2 | .6553 | .01307 | .6302 | .6815 |

| **Pairwise Comparisons** | | | | | | | | |
| --- | --- | --- | --- | --- | --- | --- | --- | --- |
| Diagnostic | (I) Gender | (J) Gender | Mean Difference (I-J) | Standard Error | Degrees of Freedom | P | 95% Wald Confidence Interval | |
|  |  |  |  |  |  |  | Lower Bound | Upper Bound |
| 1 | 1 | 2 | .0762 | .02184 | 1 | .000 | .0334 | .1190 |
|  | 2 | 1 | -.0762 | .02184 | 1 | .000 | -.1190 | -.0334 |
| 2 | 1 | 2 | .0396 | .01555 | 1 | .011 | .0091 | .0701 |
|  | 2 | 1 | -.0396 | .01555 | 1 | .011 | -.0701 | -.0091 |

| **Overall Test** | | | |
| --- | --- | --- | --- |
| Diagnostic | Wald χ² | Degrees of Freedom | P |
| 1 | 12.166 | 1 | .000 |
| 2 | 6.482 | 1 | .011 |

**Estimated Marginal Means 14：Gender* Diagnostic**

| **Estimate** | | | | | |
| --- | --- | --- | --- | --- | --- |
| Gender | Diagnostic | Mean | Standard Error | 95% Wald Confidence Interval | |
|  |  |  |  | Lower Bound | Upper Bound |
| 1 | 1 | .7384 | .01640 | .7069 | .7712 |
|  | 2 | .6949 | .01215 | .6715 | .7191 |
| 2 | 1 | .6622 | .01785 | .6281 | .6981 |
|  | 2 | .6553 | .01307 | .6302 | .6815 |

| **Pairwise Comparisons** | | | | | | | | |
| --- | --- | --- | --- | --- | --- | --- | --- | --- |
| Gender | (I) Diagnostic | (J) Diagnostic | Mean Difference (I-J) | Standard Error | Degrees of Freedom | P | 95% Wald Confidence Interval | |
|  |  |  |  |  |  |  | Lower Bound | Upper Bound |
| 1 | 1 | 2 | .0434 | .01895 | 1 | .022 | .0063 | .0806 |
|  | 2 | 1 | -.0434 | .01895 | 1 | .022 | -.0806 | -.0063 |
| 2 | 1 | 2 | .0069 | .01996 | 1 | .731 | -.0323 | .0460 |
|  | 2 | 1 | -.0069 | .01996 | 1 | .731 | -.0460 | .0323 |

| **Overall Test** | | | |
| --- | --- | --- | --- |
| Gender | Wald χ² | Degrees of Freedom | P |
| 1 | 5.252 | 1 | .022 |
| 2 | .118 | 1 | .731 |

**Estimated Marginal Means 15：Age* Diagnostic**

| **Estimate** | | | | | |
| --- | --- | --- | --- | --- | --- |
| Age | Diagnostic | Mean | Standard Error | 95% Wald Confidence Interval | |
|  |  |  |  | Lower Bound | Upper Bound |
| 1 | 1 | .2123 | .00727 | .1986 | .2271 |
|  | 2 | .1904 | .00337 | .1839 | .1972 |
| 2 | 1 | .5425 | .01266 | .5182 | .5678 |
|  | 2 | .6096 | .01103 | .5883 | .6316 |
| 3 | 1 | 1.1988 | .04711 | 1.1100 | 1.2948 |
|  | 2 | 1.1255 | .03580 | 1.0574 | 1.1979 |
| 4 | 1 | 1.7312 | .07227 | 1.5952 | 1.8788 |
|  | 2 | 1.5876 | .05973 | 1.4748 | 1.7091 |

| **Pairwise Comparisons** | | | | | | | | |
| --- | --- | --- | --- | --- | --- | --- | --- | --- |
| Diagnostic | (I) Age | (J) Age | Mean Difference (I-J) | Standard Error | Degrees of Freedom | P | 95% Wald Confidence Interval | |
|  |  |  |  |  |  |  | Lower Bound | Upper Bound |
| 1 | 1 | 2 | -.3301 | .01402 | 1 | .000 | -.3576 | -.3026 |
|  |  | 3 | -.9865 | .04714 | 1 | .000 | -1.0789 | -.8941 |
|  |  | 4 | -1.5188 | .07235 | 1 | .000 | -1.6606 | -1.3770 |
|  | 2 | 1 | .3301 | .01402 | 1 | .000 | .3026 | .3576 |
|  |  | 3 | -.6564 | .04776 | 1 | .000 | -.7500 | -.5628 |
|  |  | 4 | -1.1887 | .07289 | 1 | .000 | -1.3316 | -1.0459 |
|  | 3 | 1 | .9865 | .04714 | 1 | .000 | .8941 | 1.0789 |
|  |  | 2 | .6564 | .04776 | 1 | .000 | .5628 | .7500 |
|  |  | 4 | -.5323 | .08504 | 1 | .000 | -.6990 | -.3657 |
|  | 4 | 1 | 1.5188 | .07235 | 1 | .000 | 1.3770 | 1.6606 |
|  |  | 2 | 1.1887 | .07289 | 1 | .000 | 1.0459 | 1.3316 |
|  |  | 3 | .5323 | .08504 | 1 | .000 | .3657 | .6990 |
| 2 | 1 | 2 | -.4191 | .01136 | 1 | .000 | -.4414 | -.3969 |
|  |  | 3 | -.9350 | .03585 | 1 | .000 | -1.0053 | -.8648 |
|  |  | 4 | -1.3972 | .05968 | 1 | .000 | -1.5142 | -1.2802 |
|  | 2 | 1 | .4191 | .01136 | 1 | .000 | .3969 | .4414 |
|  |  | 3 | -.5159 | .03689 | 1 | .000 | -.5882 | -.4436 |
|  |  | 4 | -.9781 | .05982 | 1 | .000 | -1.0953 | -.8608 |
|  | 3 | 1 | .9350 | .03585 | 1 | .000 | .8648 | 1.0053 |
|  |  | 2 | .5159 | .03689 | 1 | .000 | .4436 | .5882 |
|  |  | 4 | -.4622 | .06819 | 1 | .000 | -.5958 | -.3285 |
|  | 4 | 1 | 1.3972 | .05968 | 1 | .000 | 1.2802 | 1.5142 |
|  |  | 2 | .9781 | .05982 | 1 | .000 | .8608 | 1.0953 |
|  |  | 3 | .4622 | .06819 | 1 | .000 | .3285 | .5958 |

| **Overall Test** | | | |
| --- | --- | --- | --- |
| Diagnostic | Wald χ² | Degrees of Freedom | P |
| 1 | 1267.273 | 3 | .000 |
| 2 | 2300.999 | 3 | .000 |

**Estimated Marginal Means 16：Age* Diagnostic**

| **Estimate** | | | | | |
| --- | --- | --- | --- | --- | --- |
| Age | Diagnostic | Mean | Standard Error | 95% Wald Confidence Interval | |
|  |  |  |  | Lower Bound | Upper Bound |
| 1 | 1 | .2123 | .00727 | .1986 | .2271 |
|  | 2 | .1904 | .00337 | .1839 | .1972 |
| 2 | 1 | .5425 | .01266 | .5182 | .5678 |
|  | 2 | .6096 | .01103 | .5883 | .6316 |
| 3 | 1 | 1.1988 | .04711 | 1.1100 | 1.2948 |
|  | 2 | 1.1255 | .03580 | 1.0574 | 1.1979 |
| 4 | 1 | 1.7312 | .07227 | 1.5952 | 1.8788 |
|  | 2 | 1.5876 | .05973 | 1.4748 | 1.7091 |

| **Pairwise Comparisons** | | | | | | | | |
| --- | --- | --- | --- | --- | --- | --- | --- | --- |
| Age | (I) Diagnostic | (J) Diagnostic | Mean Difference (I-J) | Standard Error | Degrees of Freedom | P | 95% Wald Confidence Interval | |
|  |  |  |  |  |  |  | Lower Bound | Upper Bound |
| 1 | 1 | 2 | .0219 | .00766 | 1 | .004 | .0069 | .0369 |
|  | 2 | 1 | -.0219 | .00766 | 1 | .004 | -.0369 | -.0069 |
| 2 | 1 | 2 | -.0671 | .01579 | 1 | .000 | -.0980 | -.0362 |
|  | 2 | 1 | .0671 | .01579 | 1 | .000 | .0362 | .0980 |
| 3 | 1 | 2 | .0734 | .04576 | 1 | .109 | -.0163 | .1631 |
|  | 2 | 1 | -.0734 | .04576 | 1 | .109 | -.1631 | .0163 |
| 4 | 1 | 2 | .1436 | .08310 | 1 | .084 | -.0193 | .3064 |
|  | 2 | 1 | -.1436 | .08310 | 1 | .084 | -.3064 | .0193 |

| **Overall Test** | | | |
| --- | --- | --- | --- |
| Age | Wald χ² | Degrees of Freedom | P |
| 1 | 8.180 | 1 | .004 |
| 2 | 18.059 | 1 | .000 |
| 3 | 2.571 | 1 | .109 |
| 4 | 2.984 | 1 | .084 |
