# Supplementary material for: The impact of COVID-19 social isolation and reduced microbial exposure on the immune system in children: a retrospective study
Source: PeerJ. 2026 Jul 7;14:e21469. doi: 10.7717/peerj.21469 (PMC13353229; doi:10.7717/peerj.21469)
Supplement: Supplemental Information 10 [file peerj-14-21469-s010.docx]

**Immunoglobulin M Generalized Linear Model**

For immunoglobulin analysis, patients were grouped as follows:
Group 1: 0–1 year,
Group 2: 1–4 years,
Group 3: 4–6 years,
Group 4: 6–13 years.

| **Parameter Estimate** | | | | | | | |
| --- | --- | --- | --- | --- | --- | --- | --- |
| Parameter | B | Standard Error | 95% Wald Confidence Interval | | Hypothesis Testing | | |
|  |  |  | Lower Bound | Upper Bound | Wald χ² | Degrees of Freedom | P |
| （Intercept） | .543 | .0554 | .434 | .651 | 95.875 | 1 | .000 |
| [Year=2020] | -.037 | .1103 | -.253 | .179 | .111 | 1 | .739 |
| [Year=2021] | -.133 | .0804 | -.291 | .025 | 2.736 | 1 | .098 |
| [Year=2022] | -.048 | .0704 | -.186 | .090 | .467 | 1 | .495 |
| [Year=2023] | -.135 | .0576 | -.248 | -.022 | 5.462 | 1 | .019 |
| [Year=2024] | -.109 | .0582 | -.223 | .005 | 3.534 | 1 | .060 |
| [Year=2025] | 0 | . | . | . | . | . | . |
| [Gender=1] | -.219 | .0402 | -.298 | -.140 | 29.675 | 1 | .000 |
| [Gender=2] | 0 | . | . | . | . | . | . |
| [Age=1] | -.940 | .0582 | -1.054 | -.826 | 260.881 | 1 | .000 |
| [Age=2] | -.333 | .0561 | -.443 | -.224 | 35.390 | 1 | .000 |
| [Age=3] | -.282 | .0627 | -.405 | -.159 | 20.173 | 1 | .000 |
| [Age=4] | 0 | . | . | . | . | . | . |
| [Diagnostic=1] | .028 | .0444 | -.059 | .115 | .397 | 1 | .528 |
| [Diagnostic=2] | 0 | . | . | . | . | . | . |
| [Year=2020] * [Gender=1] | -.124 | .0627 | -.246 | -.001 | 3.888 | 1 | .049 |
| [Year=2020] * [Gender=2] | 0 | . | . | . | . | . | . |
| [Year=2021] * [Gender=1] | -.011 | .0444 | -.098 | .076 | .066 | 1 | .797 |
| [Year=2021] * [Gender=2] | 0 | . | . | . | . | . | . |
| [Year=2022] * [Gender=1] | .001 | .0340 | -.066 | .067 | .000 | 1 | .984 |
| [Year=2022] * [Gender=2] | 0 | . | . | . | . | . | . |
| [Year=2023] * [Gender=1] | -.005 | .0335 | -.070 | .061 | .019 | 1 | .889 |
| [Year=2023] * [Gender=2] | 0 | . | . | . | . | . | . |
| [Year=2024] * [Gender=1] | .021 | .0329 | -.043 | .086 | .414 | 1 | .520 |
| [Year=2024] * [Gender=2] | 0 | . | . | . | . | . | . |
| [Year=2025] * [Gender=1] | 0 | . | . | . | . | . | . |
| [Year=2025] * [Gender=2] | 0 | . | . | . | . | . | . |
| [Year=2020] * [Age=1] | -.022 | .1099 | -.237 | .194 | .040 | 1 | .842 |
| [Year=2020] * [Age=2] | .037 | .1063 | -.171 | .246 | .122 | 1 | .727 |
| [Year=2020] * [Age=3] | .125 | .1420 | -.153 | .403 | .777 | 1 | .378 |
| [Year=2020] * [Age=4] | 0 | . | . | . | . | . | . |
| [Year=2021] * [Age=1] | .145 | .0826 | -.017 | .307 | 3.093 | 1 | .079 |
| [Year=2021] * [Age=2] | .217 | .0799 | .060 | .373 | 7.355 | 1 | .007 |
| [Year=2021] * [Age=3] | .236 | .0889 | .062 | .410 | 7.061 | 1 | .008 |
| [Year=2021] * [Age=4] | 0 | . | . | . | . | . | . |
| [Year=2022] * [Age=1] | .031 | .0722 | -.111 | .172 | .179 | 1 | .673 |
| [Year=2022] * [Age=2] | .080 | .0702 | -.058 | .217 | 1.282 | 1 | .258 |
| [Year=2022] * [Age=3] | .090 | .0774 | -.062 | .241 | 1.341 | 1 | .247 |
| [Year=2022] * [Age=4] | 0 | . | . | . | . | . | . |
| [Year=2023] * [Age=1] | .159 | .0605 | .040 | .278 | 6.893 | 1 | .009 |
| [Year=2023] * [Age=2] | .152 | .0576 | .040 | .265 | 6.999 | 1 | .008 |
| [Year=2023] * [Age=3] | .175 | .0650 | .048 | .303 | 7.282 | 1 | .007 |
| [Year=2023] * [Age=4] | 0 | . | . | . | . | . | . |
| [Year=2024] * [Age=1] | .181 | .0603 | .062 | .299 | 8.975 | 1 | .003 |
| [Year=2024] * [Age=2] | .174 | .0584 | .060 | .288 | 8.878 | 1 | .003 |
| [Year=2024] * [Age=3] | .210 | .0658 | .081 | .339 | 10.207 | 1 | .001 |
| [Year=2024] * [Age=4] | 0 | . | . | . | . | . | . |
| [Year=2025] * [Age=1] | 0 | . | . | . | . | . | . |
| [Year=2025] * [Age=2] | 0 | . | . | . | . | . | . |
| [Year=2025] * [Age=3] | 0 | . | . | . | . | . | . |
| [Year=2025] * [Age=4] | 0 | . | . | . | . | . | . |
| [Year=2020] * [Diagnostic=1] | .131 | .0664 | .001 | .262 | 3.916 | 1 | .048 |
| [Year=2020] * [Diagnostic=2] | 0 | . | . | . | . | . | . |
| [Year=2021] * [Diagnostic=1] | -.021 | .0475 | -.115 | .072 | .203 | 1 | .653 |
| [Year=2021] * [Diagnostic=2] | 0 | . | . | . | . | . | . |
| [Year=2022] * [Diagnostic=1] | -.022 | .0377 | -.096 | .052 | .339 | 1 | .561 |
| [Year=2022] * [Diagnostic=2] | 0 | . | . | . | . | . | . |
| [Year=2023] * [Diagnostic=1] | -.049 | .0359 | -.120 | .021 | 1.882 | 1 | .170 |
| [Year=2023] * [Diagnostic=2] | 0 | . | . | . | . | . | . |
| [Year=2024] * [Diagnostic=1] | -.103 | .0364 | -.174 | -.031 | 7.937 | 1 | .005 |
| [Year=2024] * [Diagnostic=2] | 0 | . | . | . | . | . | . |
| [Year=2025] * [Diagnostic=1] | 0 | . | . | . | . | . | . |
| [Year=2025] * [Diagnostic=2] | 0 | . | . | . | . | . | . |
| [Gender=1] * [Age=1] | .115 | .0346 | .048 | .183 | 11.120 | 1 | .001 |
| [Gender=1] * [Age=2] | .067 | .0332 | .002 | .132 | 4.060 | 1 | .044 |
| [Gender=1] * [Age=3] | .080 | .0368 | .008 | .152 | 4.749 | 1 | .029 |
| [Gender=1] * [Age=4] | 0 | . | . | . | . | . | . |
| [Gender=2] * [Age=1] | 0 | . | . | . | . | . | . |
| [Gender=2] * [Age=2] | 0 | . | . | . | . | . | . |
| [Gender=2] * [Age=3] | 0 | . | . | . | . | . | . |
| [Gender=2] * [Age=4] | 0 | . | . | . | . | . | . |
| [Gender=1] * [Diagnostic=1] | .010 | .0226 | -.034 | .054 | .188 | 1 | .665 |
| [Gender=1] * [Diagnostic=2] | 0 | . | . | . | . | . | . |
| [Gender=2] * [Diagnostic=1] | 0 | . | . | . | . | . | . |
| [Gender=2] * [Diagnostic=2] | 0 | . | . | . | . | . | . |
| [Age=1] * [Diagnostic=1] | -.002 | .0402 | -.081 | .077 | .003 | 1 | .955 |
| [Age=1] * [Diagnostic=2] | 0 | . | . | . | . | . | . |
| [Age=2] * [Diagnostic=1] | -.111 | .0367 | -.183 | -.040 | 9.242 | 1 | .002 |
| [Age=2] * [Diagnostic=2] | 0 | . | . | . | . | . | . |
| [Age=3] * [Diagnostic=1] | .000 | .0406 | -.080 | .079 | .000 | 1 | .996 |
| [Age=3] * [Diagnostic=2] | 0 | . | . | . | . | . | . |
| [Age=4] * [Diagnostic=1] | 0 | . | . | . | . | . | . |
| [Age=4] * [Diagnostic=2] | 0 | . | . | . | . | . | . |
| （标度） | .179 | .0028 | .174 | .184 |  |  |  |

**Estimated Marginal Means 1：Year**

| **Estimate** | | | | |
| --- | --- | --- | --- | --- |
| Year | Mean | Standard Error | 95% Wald Confidence Interval | |
|  |  |  | Lower Bound | Upper Bound |
| 2020 | 1.0850 | .03930 | 1.0107 | 1.1649 |
| 2021 | 1.0828 | .02362 | 1.0374 | 1.1301 |
| 2022 | 1.0731 | .01687 | 1.0405 | 1.1066 |
| 2023 | 1.0402 | .01167 | 1.0176 | 1.0634 |
| 2024 | 1.0730 | .01272 | 1.0484 | 1.0982 |
| 2025 | 1.0825 | .01787 | 1.0481 | 1.1181 |

| **Pairwise Comparisons** | | | | | | | |
| --- | --- | --- | --- | --- | --- | --- | --- |
| (I) Year | (J) Year | Mean Difference (I-J) | Standard Error | Degrees of Freedom | P | 95% Wald Confidence Interval | |
|  |  |  |  |  |  | Lower Bound | Upper Bound |
| 2020 | 2021 | .0023 | .04583 | 1 | .960 | -.0875 | .0921 |
|  | 2022 | .0120 | .04278 | 1 | .779 | -.0719 | .0958 |
|  | 2023 | .0448 | .04101 | 1 | .275 | -.0356 | .1252 |
|  | 2024 | .0120 | .04135 | 1 | .771 | -.0690 | .0931 |
|  | 2025 | .0025 | .04308 | 1 | .954 | -.0819 | .0869 |
| 2021 | 2020 | -.0023 | .04583 | 1 | .960 | -.0921 | .0875 |
|  | 2022 | .0097 | .02885 | 1 | .737 | -.0468 | .0662 |
|  | 2023 | .0425 | .02625 | 1 | .105 | -.0089 | .0940 |
|  | 2024 | .0098 | .02670 | 1 | .715 | -.0426 | .0621 |
|  | 2025 | .0002 | .02956 | 1 | .994 | -.0577 | .0581 |
| 2022 | 2020 | -.0120 | .04278 | 1 | .779 | -.0958 | .0719 |
|  | 2021 | -.0097 | .02885 | 1 | .737 | -.0662 | .0468 |
|  | 2023 | .0328 | .02037 | 1 | .107 | -.0071 | .0727 |
|  | 2024 | .0001 | .02094 | 1 | .998 | -.0410 | .0411 |
|  | 2025 | -.0095 | .02453 | 1 | .699 | -.0576 | .0386 |
| 2023 | 2020 | -.0448 | .04101 | 1 | .275 | -.1252 | .0356 |
|  | 2021 | -.0425 | .02625 | 1 | .105 | -.0940 | .0089 |
|  | 2022 | -.0328 | .02037 | 1 | .107 | -.0727 | .0071 |
|  | 2024 | -.0328 | .01713 | 1 | .056 | -.0663 | .0008 |
|  | 2025 | -.0423 | .02133 | 1 | .047 | -.0841 | -.0005 |
| 2024 | 2020 | -.0120 | .04135 | 1 | .771 | -.0931 | .0690 |
|  | 2021 | -.0098 | .02670 | 1 | .715 | -.0621 | .0426 |
|  | 2022 | -.0001 | .02094 | 1 | .998 | -.0411 | .0410 |
|  | 2023 | .0328 | .01713 | 1 | .056 | -.0008 | .0663 |
|  | 2025 | -.0095 | .02194 | 1 | .664 | -.0525 | .0335 |
| 2025 | 2020 | -.0025 | .04308 | 1 | .954 | -.0869 | .0819 |
|  | 2021 | -.0002 | .02956 | 1 | .994 | -.0581 | .0577 |
|  | 2022 | .0095 | .02453 | 1 | .699 | -.0386 | .0576 |
|  | 2023 | .0423 | .02133 | 1 | .047 | .0005 | .0841 |
|  | 2024 | .0095 | .02194 | 1 | .664 | -.0335 | .0525 |

| **Overall Test** | | |
| --- | --- | --- |
| Wald χ² | Degrees of Freedom | P |
| 6.978 | 5 | .222 |

**Estimated Marginal Means 2：Gender**

| **Estimate** | | | | |
| --- | --- | --- | --- | --- |
| Gender | Mean | Standard Error | 95% Wald Confidence Interval | |
|  |  |  | Lower Bound | Upper Bound |
| 1 | .9861 | .01013 | .9664 | 1.0061 |
| 2 | 1.1668 | .01440 | 1.1389 | 1.1954 |

| **Pairwise Comparisons** | | | | | | | |
| --- | --- | --- | --- | --- | --- | --- | --- |
| (I) Gender | (J) Gender | Mean Difference (I-J) | Standard Error | Degrees of Freedom | P | 95% Wald Confidence Interval | |
|  |  |  |  |  |  | Lower Bound | Upper Bound |
| 1 | 2 | -.1807 | .01648 | 1 | .000 | -.2130 | -.1484 |
| 2 | 1 | .1807 | .01648 | 1 | .000 | .1484 | .2130 |

| **Overall Test** | | |
| --- | --- | --- |
| Wald χ² | Degrees of Freedom | P |
| 120.247 | 1 | .000 |

**Estimated Marginal Means 3：Age**

| **Estimate** | | | | |
| --- | --- | --- | --- | --- |
| Age | Mean | Standard Error | 95% Wald Confidence Interval | |
|  |  |  | Lower Bound | Upper Bound |
| 1 | .6418 | .00876 | .6248 | .6592 |
| 2 | 1.1181 | .01182 | 1.0952 | 1.1415 |
| 3 | 1.2906 | .02616 | 1.2404 | 1.3429 |
| 4 | 1.4295 | .03015 | 1.3716 | 1.4899 |

| **Pairwise Comparisons** | | | | | | | |
| --- | --- | --- | --- | --- | --- | --- | --- |
| (I) Age | (J) Age | Mean Difference (I-J) | Standard Error | Degrees of Freedom | P | 95% Wald Confidence Interval | |
|  |  |  |  |  |  | Lower Bound | Upper Bound |
| 1 | 2 | -.4763 | .01462 | 1 | .000 | -.5050 | -.4477 |
|  | 3 | -.6489 | .02744 | 1 | .000 | -.7026 | -.5951 |
|  | 4 | -.7878 | .03146 | 1 | .000 | -.8494 | -.7261 |
| 2 | 1 | .4763 | .01462 | 1 | .000 | .4477 | .5050 |
|  | 3 | -.1725 | .02865 | 1 | .000 | -.2287 | -.1164 |
|  | 4 | -.3115 | .03238 | 1 | .000 | -.3749 | -.2480 |
| 3 | 1 | .6489 | .02744 | 1 | .000 | .5951 | .7026 |
|  | 2 | .1725 | .02865 | 1 | .000 | .1164 | .2287 |
|  | 4 | -.1389 | .03993 | 1 | .001 | -.2172 | -.0607 |
| 4 | 1 | .7878 | .03146 | 1 | .000 | .7261 | .8494 |
|  | 2 | .3115 | .03238 | 1 | .000 | .2480 | .3749 |
|  | 3 | .1389 | .03993 | 1 | .001 | .0607 | .2172 |

| **Overall Test** | | |
| --- | --- | --- |
| Wald χ² | Degrees of Freedom | P |
| 1740.792 | 3 | .000 |

**Estimated Marginal Means 4：Diagnostic**

| **Estimate** | | | | |
| --- | --- | --- | --- | --- |
| Diagnostic | Mean | Standard Error | 95% Wald Confidence Interval | |
|  |  |  | Lower Bound | Upper Bound |
| 1 | 1.0693 | .01372 | 1.0428 | 1.0966 |
| 2 | 1.0760 | .01093 | 1.0548 | 1.0976 |

| **Pairwise Comparisons** | | | | | | | |
| --- | --- | --- | --- | --- | --- | --- | --- |
| (I) Diagnostic | (J) Diagnostic | Mean Difference (I-J) | Standard Error | Degrees of Freedom | P | 95% Wald Confidence Interval | |
|  |  |  |  |  |  | Lower Bound | Upper Bound |
| 1 | 2 | -.0067 | .01677 | 1 | .691 | -.0395 | .0262 |
| 2 | 1 | .0067 | .01677 | 1 | .691 | -.0262 | .0395 |

| **Overall Test** | | |
| --- | --- | --- |
| Wald χ² | Degrees of Freedom | P |
| .158 | 1 | .691 |

**Estimated Marginal Means 5：Year* Gender**

| **Estimate** | | | | | |
| --- | --- | --- | --- | --- | --- |
| Year | Gender | Mean | Standard Error | 95% Wald Confidence Interval | |
|  |  |  |  | Lower Bound | Upper Bound |
| 2020 | 1 | .9469 | .04027 | .8712 | 1.0293 |
|  | 2 | 1.2433 | .06131 | 1.1287 | 1.3694 |
| 2021 | 1 | .9995 | .02417 | .9532 | 1.0480 |
|  | 2 | 1.1730 | .03732 | 1.1021 | 1.2484 |
| 2022 | 1 | .9965 | .01801 | .9618 | 1.0325 |
|  | 2 | 1.1554 | .02352 | 1.1103 | 1.2025 |
| 2023 | 1 | .9635 | .01337 | .9376 | .9900 |
|  | 2 | 1.1231 | .01829 | 1.0878 | 1.1595 |
| 2024 | 1 | 1.0067 | .01443 | .9789 | 1.0354 |
|  | 2 | 1.1436 | .01882 | 1.1073 | 1.1811 |
| 2025 | 1 | 1.0050 | .02038 | .9658 | 1.0457 |
|  | 2 | 1.1661 | .02675 | 1.1148 | 1.2197 |

| **Pairwise Comparisons** | | | | | | | | |
| --- | --- | --- | --- | --- | --- | --- | --- | --- |
| Gender | (I) Year | (J) Year | Mean Difference (I-J) | Standard Error | Degrees of Freedom | P | 95% Wald Confidence Interval | |
|  |  |  |  |  |  |  | Lower Bound | Upper Bound |
| 1 | 2020 | 2021 | -.0525 | .04684 | 1 | .262 | -.1443 | .0393 |
|  |  | 2022 | -.0496 | .04389 | 1 | .259 | -.1356 | .0364 |
|  |  | 2023 | -.0165 | .04230 | 1 | .696 | -.0994 | .0664 |
|  |  | 2024 | -.0598 | .04261 | 1 | .161 | -.1433 | .0237 |
|  |  | 2025 | -.0581 | .04487 | 1 | .196 | -.1460 | .0299 |
|  | 2021 | 2020 | .0525 | .04684 | 1 | .262 | -.0393 | .1443 |
|  |  | 2022 | .0029 | .02983 | 1 | .921 | -.0555 | .0614 |
|  |  | 2023 | .0360 | .02740 | 1 | .189 | -.0177 | .0897 |
|  |  | 2024 | -.0073 | .02791 | 1 | .795 | -.0620 | .0475 |
|  |  | 2025 | -.0055 | .03140 | 1 | .860 | -.0671 | .0560 |
|  | 2022 | 2020 | .0496 | .04389 | 1 | .259 | -.0364 | .1356 |
|  |  | 2021 | -.0029 | .02983 | 1 | .921 | -.0614 | .0555 |
|  |  | 2023 | .0331 | .02192 | 1 | .131 | -.0099 | .0760 |
|  |  | 2024 | -.0102 | .02249 | 1 | .650 | -.0543 | .0339 |
|  |  | 2025 | -.0085 | .02675 | 1 | .752 | -.0609 | .0440 |
|  | 2023 | 2020 | .0165 | .04230 | 1 | .696 | -.0664 | .0994 |
|  |  | 2021 | -.0360 | .02740 | 1 | .189 | -.0897 | .0177 |
|  |  | 2022 | -.0331 | .02192 | 1 | .131 | -.0760 | .0099 |
|  |  | 2024 | -.0433 | .01920 | 1 | .024 | -.0809 | -.0057 |
|  |  | 2025 | -.0415 | .02407 | 1 | .084 | -.0887 | .0056 |
|  | 2024 | 2020 | .0598 | .04261 | 1 | .161 | -.0237 | .1433 |
|  |  | 2021 | .0073 | .02791 | 1 | .795 | -.0475 | .0620 |
|  |  | 2022 | .0102 | .02249 | 1 | .650 | -.0339 | .0543 |
|  |  | 2023 | .0433 | .01920 | 1 | .024 | .0057 | .0809 |
|  |  | 2025 | .0017 | .02463 | 1 | .944 | -.0465 | .0500 |
|  | 2025 | 2020 | .0581 | .04487 | 1 | .196 | -.0299 | .1460 |
|  |  | 2021 | .0055 | .03140 | 1 | .860 | -.0560 | .0671 |
|  |  | 2022 | .0085 | .02675 | 1 | .752 | -.0440 | .0609 |
|  |  | 2023 | .0415 | .02407 | 1 | .084 | -.0056 | .0887 |
|  |  | 2024 | -.0017 | .02463 | 1 | .944 | -.0500 | .0465 |
| 2 | 2020 | 2021 | .0703 | .07140 | 1 | .325 | -.0696 | .2103 |
|  |  | 2022 | .0878 | .06531 | 1 | .179 | -.0402 | .2158 |
|  |  | 2023 | .1202 | .06382 | 1 | .060 | -.0049 | .2452 |
|  |  | 2024 | .0997 | .06393 | 1 | .119 | -.0256 | .2249 |
|  |  | 2025 | .0772 | .06654 | 1 | .246 | -.0532 | .2076 |
|  | 2021 | 2020 | -.0703 | .07140 | 1 | .325 | -.2103 | .0696 |
|  |  | 2022 | .0175 | .04301 | 1 | .684 | -.0668 | .1018 |
|  |  | 2023 | .0498 | .04092 | 1 | .223 | -.0304 | .1301 |
|  |  | 2024 | .0293 | .04089 | 1 | .473 | -.0508 | .1095 |
|  |  | 2025 | .0069 | .04524 | 1 | .879 | -.0818 | .0956 |
|  | 2022 | 2020 | -.0878 | .06531 | 1 | .179 | -.2158 | .0402 |
|  |  | 2021 | -.0175 | .04301 | 1 | .684 | -.1018 | .0668 |
|  |  | 2023 | .0323 | .02896 | 1 | .264 | -.0244 | .0891 |
|  |  | 2024 | .0118 | .02890 | 1 | .682 | -.0448 | .0685 |
|  |  | 2025 | -.0106 | .03482 | 1 | .760 | -.0789 | .0576 |
|  | 2023 | 2020 | -.1202 | .06382 | 1 | .060 | -.2452 | .0049 |
|  |  | 2021 | -.0498 | .04092 | 1 | .223 | -.1301 | .0304 |
|  |  | 2022 | -.0323 | .02896 | 1 | .264 | -.0891 | .0244 |
|  |  | 2024 | -.0205 | .02542 | 1 | .420 | -.0703 | .0293 |
|  |  | 2025 | -.0430 | .03196 | 1 | .179 | -.1056 | .0197 |
|  | 2024 | 2020 | -.0997 | .06393 | 1 | .119 | -.2249 | .0256 |
|  |  | 2021 | -.0293 | .04089 | 1 | .473 | -.1095 | .0508 |
|  |  | 2022 | -.0118 | .02890 | 1 | .682 | -.0685 | .0448 |
|  |  | 2023 | .0205 | .02542 | 1 | .420 | -.0293 | .0703 |
|  |  | 2025 | -.0224 | .03210 | 1 | .484 | -.0854 | .0405 |
|  | 2025 | 2020 | -.0772 | .06654 | 1 | .246 | -.2076 | .0532 |
|  |  | 2021 | -.0069 | .04524 | 1 | .879 | -.0956 | .0818 |
|  |  | 2022 | .0106 | .03482 | 1 | .760 | -.0576 | .0789 |
|  |  | 2023 | .0430 | .03196 | 1 | .179 | -.0197 | .1056 |
|  |  | 2024 | .0224 | .03210 | 1 | .484 | -.0405 | .0854 |

| **Overall Test** | | | |
| --- | --- | --- | --- |
| Gender | Wald χ² | Degrees of Freedom | P |
| 1 | 7.517 | 5 | .185 |
| 2 | 5.401 | 5 | .369 |

**Estimated Marginal Means 6：Year* Gender**

| **Estimate** | | | | | |
| --- | --- | --- | --- | --- | --- |
| Year | Gender | Mean | Standard Error | 95% Wald Confidence Interval | |
|  |  |  |  | Lower Bound | Upper Bound |
| 2020 | 1 | .9469 | .04027 | .8712 | 1.0293 |
|  | 2 | 1.2433 | .06131 | 1.1287 | 1.3694 |
| 2021 | 1 | .9995 | .02417 | .9532 | 1.0480 |
|  | 2 | 1.1730 | .03732 | 1.1021 | 1.2484 |
| 2022 | 1 | .9965 | .01801 | .9618 | 1.0325 |
|  | 2 | 1.1554 | .02352 | 1.1103 | 1.2025 |
| 2023 | 1 | .9635 | .01337 | .9376 | .9900 |
|  | 2 | 1.1231 | .01829 | 1.0878 | 1.1595 |
| 2024 | 1 | 1.0067 | .01443 | .9789 | 1.0354 |
|  | 2 | 1.1436 | .01882 | 1.1073 | 1.1811 |
| 2025 | 1 | 1.0050 | .02038 | .9658 | 1.0457 |
|  | 2 | 1.1661 | .02675 | 1.1148 | 1.2197 |

| **Pairwise Comparisons** | | | | | | | | |
| --- | --- | --- | --- | --- | --- | --- | --- | --- |
| Year | (I) Gender | (J) Gender | Mean Difference (I-J) | Standard Error | Degrees of Freedom | P | 95% Wald Confidence Interval | |
|  |  |  |  |  |  |  | Lower Bound | Upper Bound |
| 2020 | 1 | 2 | -.2963 | .06476 | 1 | .000 | -.4233 | -.1694 |
|  | 2 | 1 | .2963 | .06476 | 1 | .000 | .1694 | .4233 |
| 2021 | 1 | 2 | -.1735 | .04021 | 1 | .000 | -.2523 | -.0947 |
|  | 2 | 1 | .1735 | .04021 | 1 | .000 | .0947 | .2523 |
| 2022 | 1 | 2 | -.1589 | .02434 | 1 | .000 | -.2066 | -.1112 |
|  | 2 | 1 | .1589 | .02434 | 1 | .000 | .1112 | .2066 |
| 2023 | 1 | 2 | -.1597 | .02154 | 1 | .000 | -.2019 | -.1174 |
|  | 2 | 1 | .1597 | .02154 | 1 | .000 | .1174 | .2019 |
| 2024 | 1 | 2 | -.1369 | .02152 | 1 | .000 | -.1790 | -.0947 |
|  | 2 | 1 | .1369 | .02152 | 1 | .000 | .0947 | .1790 |
| 2025 | 1 | 2 | -.1611 | .03085 | 1 | .000 | -.2215 | -.1006 |
|  | 2 | 1 | .1611 | .03085 | 1 | .000 | .1006 | .2215 |

| **Overall Test** | | | |
| --- | --- | --- | --- |
| Year | Wald χ² | Degrees of Freedom | P |
| 2020 | 20.938 | 1 | .000 |
| 2021 | 18.613 | 1 | .000 |
| 2022 | 42.636 | 1 | .000 |
| 2023 | 54.953 | 1 | .000 |
| 2024 | 40.476 | 1 | .000 |
| 2025 | 27.254 | 1 | .000 |

**Estimated Marginal Means 7：Year* Age**

| **Estimate** | | | | | |
| --- | --- | --- | --- | --- | --- |
| Year | Age | Mean | Standard Error | 95% Wald Confidence Interval | |
|  |  |  |  | Lower Bound | Upper Bound |
| 2020 | 1 | .6136 | .02896 | .5594 | .6731 |
|  | 2 | 1.1031 | .04786 | 1.0132 | 1.2010 |
|  | 3 | 1.3500 | .13526 | 1.1093 | 1.6430 |
|  | 4 | 1.5169 | .12600 | 1.2890 | 1.7850 |
| 2021 | 1 | .6455 | .02176 | .6042 | .6896 |
|  | 2 | 1.1748 | .03241 | 1.1129 | 1.2400 |
|  | 3 | 1.3426 | .05294 | 1.2427 | 1.4505 |
|  | 4 | 1.3500 | .07759 | 1.2061 | 1.5109 |
| 2022 | 1 | .6301 | .01332 | .6045 | .6567 |
|  | 2 | 1.1213 | .01831 | 1.0860 | 1.1578 |
|  | 3 | 1.2696 | .03136 | 1.2096 | 1.3325 |
|  | 4 | 1.4780 | .07029 | 1.3465 | 1.6224 |
| 2023 | 1 | .6464 | .01398 | .6195 | .6744 |
|  | 2 | 1.0883 | .01759 | 1.0544 | 1.1233 |
|  | 3 | 1.2482 | .02647 | 1.1974 | 1.3011 |
|  | 4 | 1.3336 | .03350 | 1.2695 | 1.4009 |
| 2024 | 1 | .6683 | .01293 | .6434 | .6941 |
|  | 2 | 1.1249 | .01847 | 1.0892 | 1.1617 |
|  | 3 | 1.3071 | .02886 | 1.2518 | 1.3650 |
|  | 4 | 1.3491 | .03650 | 1.2794 | 1.4225 |
| 2025 | 1 | .6481 | .01655 | .6165 | .6814 |
|  | 2 | 1.0983 | .02292 | 1.0542 | 1.1441 |
|  | 3 | 1.2310 | .04186 | 1.1516 | 1.3158 |
|  | 4 | 1.5674 | .07018 | 1.4357 | 1.7112 |

| **Pairwise Comparisons** | | | | | | | | |
| --- | --- | --- | --- | --- | --- | --- | --- | --- |
| Age | (I) Year | (J) Year | Mean Difference (I-J) | Standard Error | Degrees of Freedom | P | 95% Wald Confidence Interval | |
|  |  |  |  |  |  |  | Lower Bound | Upper Bound |
| 1 | 2020 | 2021 | -.0319 | .03578 | 1 | .372 | -.1020 | .0382 |
|  |  | 2022 | -.0165 | .03133 | 1 | .599 | -.0779 | .0449 |
|  |  | 2023 | -.0328 | .03168 | 1 | .301 | -.0949 | .0293 |
|  |  | 2024 | -.0546 | .03119 | 1 | .080 | -.1158 | .0065 |
|  |  | 2025 | -.0345 | .03297 | 1 | .295 | -.0991 | .0301 |
|  | 2021 | 2020 | .0319 | .03578 | 1 | .372 | -.0382 | .1020 |
|  |  | 2022 | .0154 | .02450 | 1 | .529 | -.0326 | .0635 |
|  |  | 2023 | -.0009 | .02505 | 1 | .973 | -.0500 | .0482 |
|  |  | 2024 | -.0227 | .02436 | 1 | .351 | -.0705 | .0250 |
|  |  | 2025 | -.0026 | .02670 | 1 | .923 | -.0549 | .0497 |
|  | 2022 | 2020 | .0165 | .03133 | 1 | .599 | -.0449 | .0779 |
|  |  | 2021 | -.0154 | .02450 | 1 | .529 | -.0635 | .0326 |
|  |  | 2023 | -.0163 | .01809 | 1 | .368 | -.0517 | .0192 |
|  |  | 2024 | -.0382 | .01710 | 1 | .026 | -.0717 | -.0047 |
|  |  | 2025 | -.0180 | .02032 | 1 | .375 | -.0579 | .0218 |
|  | 2023 | 2020 | .0328 | .03168 | 1 | .301 | -.0293 | .0949 |
|  |  | 2021 | .0009 | .02505 | 1 | .973 | -.0482 | .0500 |
|  |  | 2022 | .0163 | .01809 | 1 | .368 | -.0192 | .0517 |
|  |  | 2024 | -.0219 | .01786 | 1 | .220 | -.0569 | .0131 |
|  |  | 2025 | -.0017 | .02090 | 1 | .934 | -.0427 | .0392 |
|  | 2024 | 2020 | .0546 | .03119 | 1 | .080 | -.0065 | .1158 |
|  |  | 2021 | .0227 | .02436 | 1 | .351 | -.0250 | .0705 |
|  |  | 2022 | .0382 | .01710 | 1 | .026 | .0047 | .0717 |
|  |  | 2023 | .0219 | .01786 | 1 | .220 | -.0131 | .0569 |
|  |  | 2025 | .0201 | .02013 | 1 | .317 | -.0193 | .0596 |
|  | 2025 | 2020 | .0345 | .03297 | 1 | .295 | -.0301 | .0991 |
|  |  | 2021 | .0026 | .02670 | 1 | .923 | -.0497 | .0549 |
|  |  | 2022 | .0180 | .02032 | 1 | .375 | -.0218 | .0579 |
|  |  | 2023 | .0017 | .02090 | 1 | .934 | -.0392 | .0427 |
|  |  | 2024 | -.0201 | .02013 | 1 | .317 | -.0596 | .0193 |
| 2 | 2020 | 2021 | -.0717 | .05777 | 1 | .215 | -.1849 | .0415 |
|  |  | 2022 | -.0183 | .05120 | 1 | .721 | -.1186 | .0821 |
|  |  | 2023 | .0148 | .05094 | 1 | .772 | -.0851 | .1146 |
|  |  | 2024 | -.0218 | .05124 | 1 | .671 | -.1222 | .0786 |
|  |  | 2025 | .0048 | .05304 | 1 | .928 | -.0991 | .1088 |
|  | 2021 | 2020 | .0717 | .05777 | 1 | .215 | -.0415 | .1849 |
|  |  | 2022 | .0534 | .03702 | 1 | .149 | -.0191 | .1260 |
|  |  | 2023 | .0865 | .03667 | 1 | .018 | .0146 | .1583 |
|  |  | 2024 | .0499 | .03705 | 1 | .178 | -.0227 | .1225 |
|  |  | 2025 | .0765 | .03957 | 1 | .053 | -.0010 | .1541 |
|  | 2022 | 2020 | .0183 | .05120 | 1 | .721 | -.0821 | .1186 |
|  |  | 2021 | -.0534 | .03702 | 1 | .149 | -.1260 | .0191 |
|  |  | 2023 | .0330 | .02506 | 1 | .187 | -.0161 | .0822 |
|  |  | 2024 | -.0035 | .02560 | 1 | .891 | -.0537 | .0466 |
|  |  | 2025 | .0231 | .02915 | 1 | .428 | -.0340 | .0802 |
|  | 2023 | 2020 | -.0148 | .05094 | 1 | .772 | -.1146 | .0851 |
|  |  | 2021 | -.0865 | .03667 | 1 | .018 | -.1583 | -.0146 |
|  |  | 2022 | -.0330 | .02506 | 1 | .187 | -.0822 | .0161 |
|  |  | 2024 | -.0366 | .02507 | 1 | .145 | -.0857 | .0126 |
|  |  | 2025 | -.0100 | .02869 | 1 | .728 | -.0662 | .0463 |
|  | 2024 | 2020 | .0218 | .05124 | 1 | .671 | -.0786 | .1222 |
|  |  | 2021 | -.0499 | .03705 | 1 | .178 | -.1225 | .0227 |
|  |  | 2022 | .0035 | .02560 | 1 | .891 | -.0466 | .0537 |
|  |  | 2023 | .0366 | .02507 | 1 | .145 | -.0126 | .0857 |
|  |  | 2025 | .0266 | .02919 | 1 | .362 | -.0306 | .0838 |
|  | 2025 | 2020 | -.0048 | .05304 | 1 | .928 | -.1088 | .0991 |
|  |  | 2021 | -.0765 | .03957 | 1 | .053 | -.1541 | .0010 |
|  |  | 2022 | -.0231 | .02915 | 1 | .428 | -.0802 | .0340 |
|  |  | 2023 | .0100 | .02869 | 1 | .728 | -.0463 | .0662 |
|  |  | 2024 | -.0266 | .02919 | 1 | .362 | -.0838 | .0306 |
| 3 | 2020 | 2021 | .0075 | .14509 | 1 | .959 | -.2769 | .2918 |
|  |  | 2022 | .0805 | .13870 | 1 | .562 | -.1914 | .3523 |
|  |  | 2023 | .1019 | .13768 | 1 | .459 | -.1680 | .3717 |
|  |  | 2024 | .0429 | .13806 | 1 | .756 | -.2277 | .3135 |
|  |  | 2025 | .1191 | .14149 | 1 | .400 | -.1582 | .3964 |
|  | 2021 | 2020 | -.0075 | .14509 | 1 | .959 | -.2918 | .2769 |
|  |  | 2022 | .0730 | .06107 | 1 | .232 | -.0467 | .1927 |
|  |  | 2023 | .0944 | .05873 | 1 | .108 | -.0207 | .2095 |
|  |  | 2024 | .0354 | .05965 | 1 | .552 | -.0815 | .1523 |
|  |  | 2025 | .1116 | .06722 | 1 | .097 | -.0201 | .2434 |
|  | 2022 | 2020 | -.0805 | .13870 | 1 | .562 | -.3523 | .1914 |
|  |  | 2021 | -.0730 | .06107 | 1 | .232 | -.1927 | .0467 |
|  |  | 2023 | .0214 | .04036 | 1 | .596 | -.0577 | .1005 |
|  |  | 2024 | -.0376 | .04151 | 1 | .365 | -.1189 | .0438 |
|  |  | 2025 | .0386 | .05187 | 1 | .457 | -.0630 | .1403 |
|  | 2023 | 2020 | -.1019 | .13768 | 1 | .459 | -.3717 | .1680 |
|  |  | 2021 | -.0944 | .05873 | 1 | .108 | -.2095 | .0207 |
|  |  | 2022 | -.0214 | .04036 | 1 | .596 | -.1005 | .0577 |
|  |  | 2024 | -.0590 | .03817 | 1 | .122 | -.1338 | .0158 |
|  |  | 2025 | .0172 | .04916 | 1 | .726 | -.0791 | .1136 |
|  | 2024 | 2020 | -.0429 | .13806 | 1 | .756 | -.3135 | .2277 |
|  |  | 2021 | -.0354 | .05965 | 1 | .552 | -.1523 | .0815 |
|  |  | 2022 | .0376 | .04151 | 1 | .365 | -.0438 | .1189 |
|  |  | 2023 | .0590 | .03817 | 1 | .122 | -.0158 | .1338 |
|  |  | 2025 | .0762 | .05024 | 1 | .129 | -.0223 | .1747 |
|  | 2025 | 2020 | -.1191 | .14149 | 1 | .400 | -.3964 | .1582 |
|  |  | 2021 | -.1116 | .06722 | 1 | .097 | -.2434 | .0201 |
|  |  | 2022 | -.0386 | .05187 | 1 | .457 | -.1403 | .0630 |
|  |  | 2023 | -.0172 | .04916 | 1 | .726 | -.1136 | .0791 |
|  |  | 2024 | -.0762 | .05024 | 1 | .129 | -.1747 | .0223 |
| 4 | 2020 | 2021 | .1669 | .14821 | 1 | .260 | -.1236 | .4574 |
|  |  | 2022 | .0388 | .14482 | 1 | .789 | -.2450 | .3227 |
|  |  | 2023 | .1833 | .13107 | 1 | .162 | -.0736 | .4402 |
|  |  | 2024 | .1678 | .13212 | 1 | .204 | -.0912 | .4267 |
|  |  | 2025 | -.0506 | .14391 | 1 | .725 | -.3326 | .2315 |
|  | 2021 | 2020 | -.1669 | .14821 | 1 | .260 | -.4574 | .1236 |
|  |  | 2022 | -.1281 | .10406 | 1 | .218 | -.3320 | .0759 |
|  |  | 2023 | .0164 | .08382 | 1 | .845 | -.1479 | .1807 |
|  |  | 2024 | .0009 | .08481 | 1 | .992 | -.1653 | .1671 |
|  |  | 2025 | -.2175 | .10455 | 1 | .038 | -.4224 | -.0125 |
|  | 2022 | 2020 | -.0388 | .14482 | 1 | .789 | -.3227 | .2450 |
|  |  | 2021 | .1281 | .10406 | 1 | .218 | -.0759 | .3320 |
|  |  | 2023 | .1445 | .07688 | 1 | .060 | -.0062 | .2951 |
|  |  | 2024 | .1290 | .07788 | 1 | .098 | -.0237 | .2816 |
|  |  | 2025 | -.0894 | .09948 | 1 | .369 | -.2844 | .1056 |
|  | 2023 | 2020 | -.1833 | .13107 | 1 | .162 | -.4402 | .0736 |
|  |  | 2021 | -.0164 | .08382 | 1 | .845 | -.1807 | .1479 |
|  |  | 2022 | -.1445 | .07688 | 1 | .060 | -.2951 | .0062 |
|  |  | 2024 | -.0155 | .04730 | 1 | .743 | -.1082 | .0772 |
|  |  | 2025 | -.2339 | .07806 | 1 | .003 | -.3869 | -.0809 |
|  | 2024 | 2020 | -.1678 | .13212 | 1 | .204 | -.4267 | .0912 |
|  |  | 2021 | -.0009 | .08481 | 1 | .992 | -.1671 | .1653 |
|  |  | 2022 | -.1290 | .07788 | 1 | .098 | -.2816 | .0237 |
|  |  | 2023 | .0155 | .04730 | 1 | .743 | -.0772 | .1082 |
|  |  | 2025 | -.2183 | .07950 | 1 | .006 | -.3742 | -.0625 |
|  | 2025 | 2020 | .0506 | .14391 | 1 | .725 | -.2315 | .3326 |
|  |  | 2021 | .2175 | .10455 | 1 | .038 | .0125 | .4224 |
|  |  | 2022 | .0894 | .09948 | 1 | .369 | -.1056 | .2844 |
|  |  | 2023 | .2339 | .07806 | 1 | .003 | .0809 | .3869 |
|  |  | 2024 | .2183 | .07950 | 1 | .006 | .0625 | .3742 |

| **Overall Test** | | | |
| --- | --- | --- | --- |
| Age | Wald χ² | Degrees of Freedom | P |
| 1 | 6.439 | 5 | .266 |
| 2 | 6.783 | 5 | .237 |
| 3 | 5.487 | 5 | .359 |
| 4 | 12.838 | 5 | .025 |

**Estimated Marginal Means 8：Year* Age**

| **Estimate** | | | | | |
| --- | --- | --- | --- | --- | --- |
| Year | Age | Mean | Standard Error | 95% Wald Confidence Interval | |
|  |  |  |  | Lower Bound | Upper Bound |
| 2020 | 1 | .6136 | .02896 | .5594 | .6731 |
|  | 2 | 1.1031 | .04786 | 1.0132 | 1.2010 |
|  | 3 | 1.3500 | .13526 | 1.1093 | 1.6430 |
|  | 4 | 1.5169 | .12600 | 1.2890 | 1.7850 |
| 2021 | 1 | .6455 | .02176 | .6042 | .6896 |
|  | 2 | 1.1748 | .03241 | 1.1129 | 1.2400 |
|  | 3 | 1.3426 | .05294 | 1.2427 | 1.4505 |
|  | 4 | 1.3500 | .07759 | 1.2061 | 1.5109 |
| 2022 | 1 | .6301 | .01332 | .6045 | .6567 |
|  | 2 | 1.1213 | .01831 | 1.0860 | 1.1578 |
|  | 3 | 1.2696 | .03136 | 1.2096 | 1.3325 |
|  | 4 | 1.4780 | .07029 | 1.3465 | 1.6224 |
| 2023 | 1 | .6464 | .01398 | .6195 | .6744 |
|  | 2 | 1.0883 | .01759 | 1.0544 | 1.1233 |
|  | 3 | 1.2482 | .02647 | 1.1974 | 1.3011 |
|  | 4 | 1.3336 | .03350 | 1.2695 | 1.4009 |
| 2024 | 1 | .6683 | .01293 | .6434 | .6941 |
|  | 2 | 1.1249 | .01847 | 1.0892 | 1.1617 |
|  | 3 | 1.3071 | .02886 | 1.2518 | 1.3650 |
|  | 4 | 1.3491 | .03650 | 1.2794 | 1.4225 |
| 2025 | 1 | .6481 | .01655 | .6165 | .6814 |
|  | 2 | 1.0983 | .02292 | 1.0542 | 1.1441 |
|  | 3 | 1.2310 | .04186 | 1.1516 | 1.3158 |
|  | 4 | 1.5674 | .07018 | 1.4357 | 1.7112 |

| **Pairwise Comparisons** | | | | | | | | |
| --- | --- | --- | --- | --- | --- | --- | --- | --- |
| Year | (I) Age | (J) Age | Mean Difference (I-J) | Standard Error | Degrees of Freedom | P | 95% Wald Confidence Interval | |
|  |  |  |  |  |  |  | Lower Bound | Upper Bound |
| 2020 | 1 | 2 | -.4895 | .05507 | 1 | .000 | -.5974 | -.3815 |
|  |  | 3 | -.7364 | .13732 | 1 | .000 | -1.0056 | -.4673 |
|  |  | 4 | -.9032 | .13059 | 1 | .000 | -1.1592 | -.6473 |
|  | 2 | 1 | .4895 | .05507 | 1 | .000 | .3815 | .5974 |
|  |  | 3 | -.2470 | .14320 | 1 | .085 | -.5276 | .0337 |
|  |  | 4 | -.4138 | .13549 | 1 | .002 | -.6793 | -.1482 |
|  | 3 | 1 | .7364 | .13732 | 1 | .000 | .4673 | 1.0056 |
|  |  | 2 | .2470 | .14320 | 1 | .085 | -.0337 | .5276 |
|  |  | 4 | -.1668 | .18545 | 1 | .368 | -.5303 | .1967 |
|  | 4 | 1 | .9032 | .13059 | 1 | .000 | .6473 | 1.1592 |
|  |  | 2 | .4138 | .13549 | 1 | .002 | .1482 | .6793 |
|  |  | 3 | .1668 | .18545 | 1 | .368 | -.1967 | .5303 |
| 2021 | 1 | 2 | -.5293 | .03716 | 1 | .000 | -.6021 | -.4564 |
|  |  | 3 | -.6971 | .05590 | 1 | .000 | -.8066 | -.5875 |
|  |  | 4 | -.7044 | .07955 | 1 | .000 | -.8604 | -.5485 |
|  | 2 | 1 | .5293 | .03716 | 1 | .000 | .4564 | .6021 |
|  |  | 3 | -.1678 | .06087 | 1 | .006 | -.2871 | -.0485 |
|  |  | 4 | -.1752 | .08306 | 1 | .035 | -.3380 | -.0124 |
|  | 3 | 1 | .6971 | .05590 | 1 | .000 | .5875 | .8066 |
|  |  | 2 | .1678 | .06087 | 1 | .006 | .0485 | .2871 |
|  |  | 4 | -.0074 | .09290 | 1 | .937 | -.1895 | .1747 |
|  | 4 | 1 | .7044 | .07955 | 1 | .000 | .5485 | .8604 |
|  |  | 2 | .1752 | .08306 | 1 | .035 | .0124 | .3380 |
|  |  | 3 | .0074 | .09290 | 1 | .937 | -.1747 | .1895 |
| 2022 | 1 | 2 | -.4913 | .02126 | 1 | .000 | -.5329 | -.4496 |
|  |  | 3 | -.6395 | .03310 | 1 | .000 | -.7044 | -.5746 |
|  |  | 4 | -.8480 | .07103 | 1 | .000 | -.9872 | -.7087 |
|  | 2 | 1 | .4913 | .02126 | 1 | .000 | .4496 | .5329 |
|  |  | 3 | -.1482 | .03546 | 1 | .000 | -.2177 | -.0787 |
|  |  | 4 | -.3567 | .07216 | 1 | .000 | -.4981 | -.2153 |
|  | 3 | 1 | .6395 | .03310 | 1 | .000 | .5746 | .7044 |
|  |  | 2 | .1482 | .03546 | 1 | .000 | .0787 | .2177 |
|  |  | 4 | -.2085 | .07649 | 1 | .006 | -.3584 | -.0585 |
|  | 4 | 1 | .8480 | .07103 | 1 | .000 | .7087 | .9872 |
|  |  | 2 | .3567 | .07216 | 1 | .000 | .2153 | .4981 |
|  |  | 3 | .2085 | .07649 | 1 | .006 | .0585 | .3584 |
| 2023 | 1 | 2 | -.4419 | .02167 | 1 | .000 | -.4844 | -.3994 |
|  |  | 3 | -.6018 | .02941 | 1 | .000 | -.6595 | -.5442 |
|  |  | 4 | -.6872 | .03577 | 1 | .000 | -.7573 | -.6171 |
|  | 2 | 1 | .4419 | .02167 | 1 | .000 | .3994 | .4844 |
|  |  | 3 | -.1599 | .03127 | 1 | .000 | -.2212 | -.0986 |
|  |  | 4 | -.2453 | .03733 | 1 | .000 | -.3184 | -.1721 |
|  | 3 | 1 | .6018 | .02941 | 1 | .000 | .5442 | .6595 |
|  |  | 2 | .1599 | .03127 | 1 | .000 | .0986 | .2212 |
|  |  | 4 | -.0854 | .04227 | 1 | .043 | -.1682 | -.0025 |
|  | 4 | 1 | .6872 | .03577 | 1 | .000 | .6171 | .7573 |
|  |  | 2 | .2453 | .03733 | 1 | .000 | .1721 | .3184 |
|  |  | 3 | .0854 | .04227 | 1 | .043 | .0025 | .1682 |
| 2024 | 1 | 2 | -.4566 | .02134 | 1 | .000 | -.4984 | -.4148 |
|  |  | 3 | -.6389 | .03058 | 1 | .000 | -.6988 | -.5790 |
|  |  | 4 | -.6808 | .03782 | 1 | .000 | -.7549 | -.6067 |
|  | 2 | 1 | .4566 | .02134 | 1 | .000 | .4148 | .4984 |
|  |  | 3 | -.1823 | .03312 | 1 | .000 | -.2472 | -.1174 |
|  |  | 4 | -.2242 | .03990 | 1 | .000 | -.3024 | -.1460 |
|  | 3 | 1 | .6389 | .03058 | 1 | .000 | .5790 | .6988 |
|  |  | 2 | .1823 | .03312 | 1 | .000 | .1174 | .2472 |
|  |  | 4 | -.0419 | .04541 | 1 | .356 | -.1309 | .0471 |
|  | 4 | 1 | .6808 | .03782 | 1 | .000 | .6067 | .7549 |
|  |  | 2 | .2242 | .03990 | 1 | .000 | .1460 | .3024 |
|  |  | 3 | .0419 | .04541 | 1 | .356 | -.0471 | .1309 |
| 2025 | 1 | 2 | -.4502 | .02745 | 1 | .000 | -.5039 | -.3964 |
|  |  | 3 | -.5829 | .04454 | 1 | .000 | -.6701 | -.4956 |
|  |  | 4 | -.9193 | .07221 | 1 | .000 | -1.0608 | -.7778 |
|  | 2 | 1 | .4502 | .02745 | 1 | .000 | .3964 | .5039 |
|  |  | 3 | -.1327 | .04734 | 1 | .005 | -.2255 | -.0399 |
|  |  | 4 | -.4692 | .07391 | 1 | .000 | -.6140 | -.3243 |
|  | 3 | 1 | .5829 | .04454 | 1 | .000 | .4956 | .6701 |
|  |  | 2 | .1327 | .04734 | 1 | .005 | .0399 | .2255 |
|  |  | 4 | -.3365 | .08186 | 1 | .000 | -.4969 | -.1760 |
|  | 4 | 1 | .9193 | .07221 | 1 | .000 | .7778 | 1.0608 |
|  |  | 2 | .4692 | .07391 | 1 | .000 | .3243 | .6140 |
|  |  | 3 | .3365 | .08186 | 1 | .000 | .1760 | .4969 |

| **Overall Test** | | | |
| --- | --- | --- | --- |
| Year | Wald χ² | Degrees of Freedom | P |
| 2020 | 132.919 | 3 | .000 |
| 2021 | 340.978 | 3 | .000 |
| 2022 | 838.788 | 3 | .000 |
| 2023 | 828.035 | 3 | .000 |
| 2024 | 894.977 | 3 | .000 |
| 2025 | 472.748 | 3 | .000 |

**Estimated Marginal Means 9：Year* Diagnostic**

| **Estimate** | | | | | |
| --- | --- | --- | --- | --- | --- |
| Year | Diagnostic | Mean | Standard Error | 95% Wald Confidence Interval | |
|  |  |  |  | Lower Bound | Upper Bound |
| 2020 | 1 | 1.1613 | .05691 | 1.0549 | 1.2783 |
|  | 2 | 1.0138 | .04577 | .9280 | 1.1076 |
| 2021 | 1 | 1.0736 | .03618 | 1.0050 | 1.1469 |
|  | 2 | 1.0920 | .02571 | 1.0427 | 1.1436 |
| 2022 | 1 | 1.0637 | .02536 | 1.0151 | 1.1146 |
|  | 2 | 1.0825 | .01718 | 1.0493 | 1.1167 |
| 2023 | 1 | 1.0172 | .01898 | .9806 | 1.0551 |
|  | 2 | 1.0638 | .01257 | 1.0395 | 1.0887 |
| 2024 | 1 | 1.0216 | .02102 | .9812 | 1.0637 |
|  | 2 | 1.1270 | .01226 | 1.1032 | 1.1512 |
| 2025 | 1 | 1.0849 | .02622 | 1.0347 | 1.1376 |
|  | 2 | 1.0802 | .02150 | 1.0388 | 1.1231 |

| **Pairwise Comparisons** | | | | | | | | |
| --- | --- | --- | --- | --- | --- | --- | --- | --- |
| Diagnostic | (I) Year | (J) Year | Mean Difference (I-J) | Standard Error | Degrees of Freedom | P | 95% Wald Confidence Interval | |
|  |  |  |  |  |  |  | Lower Bound | Upper Bound |
| 1 | 2020 | 2021 | .0877 | .06725 | 1 | .192 | -.0441 | .2195 |
|  |  | 2022 | .0976 | .06209 | 1 | .116 | -.0241 | .2193 |
|  |  | 2023 | .1441 | .05988 | 1 | .016 | .0267 | .2614 |
|  |  | 2024 | .1396 | .06051 | 1 | .021 | .0211 | .2582 |
|  |  | 2025 | .0763 | .06248 | 1 | .222 | -.0461 | .1988 |
|  | 2021 | 2020 | -.0877 | .06725 | 1 | .192 | -.2195 | .0441 |
|  |  | 2022 | .0099 | .04338 | 1 | .819 | -.0751 | .0949 |
|  |  | 2023 | .0564 | .04035 | 1 | .162 | -.0227 | .1355 |
|  |  | 2024 | .0520 | .04125 | 1 | .208 | -.0289 | .1328 |
|  |  | 2025 | -.0113 | .04426 | 1 | .798 | -.0981 | .0754 |
|  | 2022 | 2020 | -.0976 | .06209 | 1 | .116 | -.2193 | .0241 |
|  |  | 2021 | -.0099 | .04338 | 1 | .819 | -.0949 | .0751 |
|  |  | 2023 | .0465 | .03095 | 1 | .133 | -.0141 | .1072 |
|  |  | 2024 | .0421 | .03210 | 1 | .190 | -.0208 | .1050 |
|  |  | 2025 | -.0212 | .03592 | 1 | .554 | -.0916 | .0492 |
|  | 2023 | 2020 | -.1441 | .05988 | 1 | .016 | -.2614 | -.0267 |
|  |  | 2021 | -.0564 | .04035 | 1 | .162 | -.1355 | .0227 |
|  |  | 2022 | -.0465 | .03095 | 1 | .133 | -.1072 | .0141 |
|  |  | 2024 | -.0044 | .02779 | 1 | .873 | -.0589 | .0500 |
|  |  | 2025 | -.0677 | .03203 | 1 | .034 | -.1305 | -.0050 |
|  | 2024 | 2020 | -.1396 | .06051 | 1 | .021 | -.2582 | -.0211 |
|  |  | 2021 | -.0520 | .04125 | 1 | .208 | -.1328 | .0289 |
|  |  | 2022 | -.0421 | .03210 | 1 | .190 | -.1050 | .0208 |
|  |  | 2023 | .0044 | .02779 | 1 | .873 | -.0500 | .0589 |
|  |  | 2025 | -.0633 | .03318 | 1 | .056 | -.1283 | .0017 |
|  | 2025 | 2020 | -.0763 | .06248 | 1 | .222 | -.1988 | .0461 |
|  |  | 2021 | .0113 | .04426 | 1 | .798 | -.0754 | .0981 |
|  |  | 2022 | .0212 | .03592 | 1 | .554 | -.0492 | .0916 |
|  |  | 2023 | .0677 | .03203 | 1 | .034 | .0050 | .1305 |
|  |  | 2024 | .0633 | .03318 | 1 | .056 | -.0017 | .1283 |
| 2 | 2020 | 2021 | -.0782 | .05220 | 1 | .134 | -.1805 | .0241 |
|  |  | 2022 | -.0687 | .04857 | 1 | .157 | -.1639 | .0265 |
|  |  | 2023 | -.0500 | .04733 | 1 | .291 | -.1427 | .0428 |
|  |  | 2024 | -.1131 | .04724 | 1 | .017 | -.2057 | -.0205 |
|  |  | 2025 | -.0663 | .05010 | 1 | .185 | -.1645 | .0319 |
|  | 2021 | 2020 | .0782 | .05220 | 1 | .134 | -.0241 | .1805 |
|  |  | 2022 | .0095 | .03067 | 1 | .757 | -.0506 | .0696 |
|  |  | 2023 | .0282 | .02851 | 1 | .323 | -.0277 | .0841 |
|  |  | 2024 | -.0350 | .02838 | 1 | .218 | -.0906 | .0207 |
|  |  | 2025 | .0118 | .03318 | 1 | .721 | -.0532 | .0769 |
|  | 2022 | 2020 | .0687 | .04857 | 1 | .157 | -.0265 | .1639 |
|  |  | 2021 | -.0095 | .03067 | 1 | .757 | -.0696 | .0506 |
|  |  | 2023 | .0187 | .02113 | 1 | .376 | -.0227 | .0601 |
|  |  | 2024 | -.0445 | .02096 | 1 | .034 | -.0856 | -.0034 |
|  |  | 2025 | .0023 | .02708 | 1 | .931 | -.0508 | .0554 |
|  | 2023 | 2020 | .0500 | .04733 | 1 | .291 | -.0428 | .1427 |
|  |  | 2021 | -.0282 | .02851 | 1 | .323 | -.0841 | .0277 |
|  |  | 2022 | -.0187 | .02113 | 1 | .376 | -.0601 | .0227 |
|  |  | 2024 | -.0632 | .01747 | 1 | .000 | -.0974 | -.0289 |
|  |  | 2025 | -.0164 | .02470 | 1 | .508 | -.0648 | .0320 |
|  | 2024 | 2020 | .1131 | .04724 | 1 | .017 | .0205 | .2057 |
|  |  | 2021 | .0350 | .02838 | 1 | .218 | -.0207 | .0906 |
|  |  | 2022 | .0445 | .02096 | 1 | .034 | .0034 | .0856 |
|  |  | 2023 | .0632 | .01747 | 1 | .000 | .0289 | .0974 |
|  |  | 2025 | .0468 | .02456 | 1 | .057 | -.0013 | .0949 |
|  | 2025 | 2020 | .0663 | .05010 | 1 | .185 | -.0319 | .1645 |
|  |  | 2021 | -.0118 | .03318 | 1 | .721 | -.0769 | .0532 |
|  |  | 2022 | -.0023 | .02708 | 1 | .931 | -.0554 | .0508 |
|  |  | 2023 | .0164 | .02470 | 1 | .508 | -.0320 | .0648 |
|  |  | 2024 | -.0468 | .02456 | 1 | .057 | -.0949 | .0013 |

| **Overall Test** | | | |
| --- | --- | --- | --- |
| Diagnostic | Wald χ² | Degrees of Freedom | P |
| 1 | 11.240 | 5 | .047 |
| 2 | 16.732 | 5 | .005 |

**Estimated Marginal Means 10：Year* Diagnostic**

| **Estimate** | | | | | |
| --- | --- | --- | --- | --- | --- |
| Year | Diagnostic | Mean | Standard Error | 95% Wald Confidence Interval | |
|  |  |  |  | Lower Bound | Upper Bound |
| 2020 | 1 | 1.1613 | .05691 | 1.0549 | 1.2783 |
|  | 2 | 1.0138 | .04577 | .9280 | 1.1076 |
| 2021 | 1 | 1.0736 | .03618 | 1.0050 | 1.1469 |
|  | 2 | 1.0920 | .02571 | 1.0427 | 1.1436 |
| 2022 | 1 | 1.0637 | .02536 | 1.0151 | 1.1146 |
|  | 2 | 1.0825 | .01718 | 1.0493 | 1.1167 |
| 2023 | 1 | 1.0172 | .01898 | .9806 | 1.0551 |
|  | 2 | 1.0638 | .01257 | 1.0395 | 1.0887 |
| 2024 | 1 | 1.0216 | .02102 | .9812 | 1.0637 |
|  | 2 | 1.1270 | .01226 | 1.1032 | 1.1512 |
| 2025 | 1 | 1.0849 | .02622 | 1.0347 | 1.1376 |
|  | 2 | 1.0802 | .02150 | 1.0388 | 1.1231 |

| **Pairwise Comparisons** | | | | | | | | |
| --- | --- | --- | --- | --- | --- | --- | --- | --- |
| Year | (I) Diagnostic | (J) Diagnostic | Mean Difference (I-J) | Standard Error | Degrees of Freedom | P | 95% Wald Confidence Interval | |
|  |  |  |  |  |  |  | Lower Bound | Upper Bound |
| 2020 | 1 | 2 | .1474 | .06621 | 1 | .026 | .0177 | .2772 |
|  | 2 | 1 | -.1474 | .06621 | 1 | .026 | -.2772 | -.0177 |
| 2021 | 1 | 2 | -.0184 | .04146 | 1 | .657 | -.0996 | .0629 |
|  | 2 | 1 | .0184 | .04146 | 1 | .657 | -.0629 | .0996 |
| 2022 | 1 | 2 | -.0188 | .02729 | 1 | .491 | -.0723 | .0347 |
|  | 2 | 1 | .0188 | .02729 | 1 | .491 | -.0347 | .0723 |
| 2023 | 1 | 2 | -.0466 | .02239 | 1 | .037 | -.0905 | -.0027 |
|  | 2 | 1 | .0466 | .02239 | 1 | .037 | .0027 | .0905 |
| 2024 | 1 | 2 | -.1053 | .02384 | 1 | .000 | -.1521 | -.0586 |
|  | 2 | 1 | .1053 | .02384 | 1 | .000 | .0586 | .1521 |
| 2025 | 1 | 2 | .0048 | .03195 | 1 | .881 | -.0579 | .0674 |
|  | 2 | 1 | -.0048 | .03195 | 1 | .881 | -.0674 | .0579 |

| **Overall Test** | | | |
| --- | --- | --- | --- |
| Year | Wald χ² | Degrees of Freedom | P |
| 2020 | 4.960 | 1 | .026 |
| 2021 | .197 | 1 | .657 |
| 2022 | .474 | 1 | .491 |
| 2023 | 4.336 | 1 | .037 |
| 2024 | 19.525 | 1 | .000 |
| 2025 | .022 | 1 | .881 |

**Estimated Marginal Means 11：Gender* Age**

| **Estimate** | | | | | |
| --- | --- | --- | --- | --- | --- |
| Gender | Age | Mean | Standard Error | 95% Wald Confidence Interval | |
|  |  |  |  | Lower Bound | Upper Bound |
| 1 | 1 | .6048 | .00928 | .5869 | .6233 |
|  | 2 | 1.0285 | .01348 | 1.0025 | 1.0553 |
|  | 3 | 1.1952 | .02693 | 1.1435 | 1.2491 |
|  | 4 | 1.2717 | .03109 | 1.2122 | 1.3342 |
| 2 | 1 | .6810 | .01281 | .6563 | .7065 |
|  | 2 | 1.2154 | .01785 | 1.1809 | 1.2509 |
|  | 3 | 1.3937 | .03474 | 1.3272 | 1.4635 |
|  | 4 | 1.6069 | .04477 | 1.5215 | 1.6971 |

| **Pairwise Comparisons** | | | | | | | | |
| --- | --- | --- | --- | --- | --- | --- | --- | --- |
| Age | (I) Gender | (J) Gender | Mean Difference (I-J) | Standard Error | Degrees of Freedom | P | 95% Wald Confidence Interval | |
|  |  |  |  |  |  |  | Lower Bound | Upper Bound |
| 1 | 1 | 2 | -.0762 | .01364 | 1 | .000 | -.1029 | -.0494 |
|  | 2 | 1 | .0762 | .01364 | 1 | .000 | .0494 | .1029 |
| 2 | 1 | 2 | -.1869 | .02063 | 1 | .000 | -.2273 | -.1465 |
|  | 2 | 1 | .1869 | .02063 | 1 | .000 | .1465 | .2273 |
| 3 | 1 | 2 | -.1985 | .03282 | 1 | .000 | -.2629 | -.1342 |
|  | 2 | 1 | .1985 | .03282 | 1 | .000 | .1342 | .2629 |
| 4 | 1 | 2 | -.3352 | .04628 | 1 | .000 | -.4259 | -.2445 |
|  | 2 | 1 | .3352 | .04628 | 1 | .000 | .2445 | .4259 |

| **Overall Test** | | | |
| --- | --- | --- | --- |
| Age | Wald χ² | Degrees of Freedom | P |
| 1 | 31.165 | 1 | .000 |
| 2 | 82.105 | 1 | .000 |
| 3 | 36.590 | 1 | .000 |
| 4 | 52.467 | 1 | .000 |

**Estimated Marginal Means 12：Gender* Age**

| **Estimate** | | | | | |
| --- | --- | --- | --- | --- | --- |
| Gender | Age | Mean | Standard Error | 95% Wald Confidence Interval | |
|  |  |  |  | Lower Bound | Upper Bound |
| 1 | 1 | .6048 | .00928 | .5869 | .6233 |
|  | 2 | 1.0285 | .01348 | 1.0025 | 1.0553 |
|  | 3 | 1.1952 | .02693 | 1.1435 | 1.2491 |
|  | 4 | 1.2717 | .03109 | 1.2122 | 1.3342 |
| 2 | 1 | .6810 | .01281 | .6563 | .7065 |
|  | 2 | 1.2154 | .01785 | 1.1809 | 1.2509 |
|  | 3 | 1.3937 | .03474 | 1.3272 | 1.4635 |
|  | 4 | 1.6069 | .04477 | 1.5215 | 1.6971 |

| **Pairwise Comparisons** | | | | | | | | |
| --- | --- | --- | --- | --- | --- | --- | --- | --- |
| Gender | (I) Age | (J) Age | Mean Difference (I-J) | Standard Error | Degrees of Freedom | P | 95% Wald Confidence Interval | |
|  |  |  |  |  |  |  | Lower Bound | Upper Bound |
| 1 | 1 | 2 | -.4237 | .01577 | 1 | .000 | -.4546 | -.3928 |
|  |  | 3 | -.5904 | .02811 | 1 | .000 | -.6454 | -.5353 |
|  |  | 4 | -.6669 | .03225 | 1 | .000 | -.7301 | -.6037 |
|  | 2 | 1 | .4237 | .01577 | 1 | .000 | .3928 | .4546 |
|  |  | 3 | -.1666 | .02936 | 1 | .000 | -.2242 | -.1091 |
|  |  | 4 | -.2432 | .03324 | 1 | .000 | -.3083 | -.1780 |
|  | 3 | 1 | .5904 | .02811 | 1 | .000 | .5353 | .6454 |
|  |  | 2 | .1666 | .02936 | 1 | .000 | .1091 | .2242 |
|  |  | 4 | -.0766 | .04059 | 1 | .059 | -.1561 | .0030 |
|  | 4 | 1 | .6669 | .03225 | 1 | .000 | .6037 | .7301 |
|  |  | 2 | .2432 | .03324 | 1 | .000 | .1780 | .3083 |
|  |  | 3 | .0766 | .04059 | 1 | .059 | -.0030 | .1561 |
| 2 | 1 | 2 | -.5345 | .02049 | 1 | .000 | -.5746 | -.4943 |
|  |  | 3 | -.7127 | .03572 | 1 | .000 | -.7827 | -.6427 |
|  |  | 4 | -.9260 | .04564 | 1 | .000 | -1.0154 | -.8365 |
|  | 2 | 1 | .5345 | .02049 | 1 | .000 | .4943 | .5746 |
|  |  | 3 | -.1783 | .03752 | 1 | .000 | -.2518 | -.1047 |
|  |  | 4 | -.3915 | .04691 | 1 | .000 | -.4834 | -.2996 |
|  | 3 | 1 | .7127 | .03572 | 1 | .000 | .6427 | .7827 |
|  |  | 2 | .1783 | .03752 | 1 | .000 | .1047 | .2518 |
|  |  | 4 | -.2132 | .05501 | 1 | .000 | -.3210 | -.1054 |
|  | 4 | 1 | .9260 | .04564 | 1 | .000 | .8365 | 1.0154 |
|  |  | 2 | .3915 | .04691 | 1 | .000 | .2996 | .4834 |
|  |  | 3 | .2132 | .05501 | 1 | .000 | .1054 | .3210 |

| **Overall Test** | | | |
| --- | --- | --- | --- |
| Gender | Wald χ² | Degrees of Freedom | P |
| 1 | 1203.573 | 3 | .000 |
| 2 | 1140.872 | 3 | .000 |

**Estimated Marginal Means 13：Gender* Diagnostic**

| **Estimate** | | | | | |
| --- | --- | --- | --- | --- | --- |
| Gender | Diagnostic | Mean | Standard Error | 95% Wald Confidence Interval | |
|  |  |  |  | Lower Bound | Upper Bound |
| 1 | 1 | .9854 | .01482 | .9568 | 1.0149 |
|  | 2 | .9867 | .01186 | .9638 | 1.0103 |
| 2 | 1 | 1.1604 | .02113 | 1.1197 | 1.2025 |
|  | 2 | 1.1733 | .01596 | 1.1425 | 1.2050 |

| **Pairwise Comparisons** | | | | | | | | |
| --- | --- | --- | --- | --- | --- | --- | --- | --- |
| Diagnostic | (I) Gender | (J) Gender | Mean Difference (I-J) | Standard Error | Degrees of Freedom | P | 95% Wald Confidence Interval | |
|  |  |  |  |  |  |  | Lower Bound | Upper Bound |
| 1 | 1 | 2 | -.1749 | .02347 | 1 | .000 | -.2209 | -.1289 |
|  | 2 | 1 | .1749 | .02347 | 1 | .000 | .1289 | .2209 |
| 2 | 1 | 2 | -.1866 | .01731 | 1 | .000 | -.2205 | -.1527 |
|  | 2 | 1 | .1866 | .01731 | 1 | .000 | .1527 | .2205 |

| **Overall Test** | | | |
| --- | --- | --- | --- |
| Diagnostic | Wald χ² | Degrees of Freedom | P |
| 1 | 55.562 | 1 | .000 |
| 2 | 116.206 | 1 | .000 |

**Estimated Marginal Means 14：Gender* Diagnostic**

| **Estimate** | | | | | |
| --- | --- | --- | --- | --- | --- |
| Gender | Diagnostic | Mean | Standard Error | 95% Wald Confidence Interval | |
|  |  |  |  | Lower Bound | Upper Bound |
| 1 | 1 | .9854 | .01482 | .9568 | 1.0149 |
|  | 2 | .9867 | .01186 | .9638 | 1.0103 |
| 2 | 1 | 1.1604 | .02113 | 1.1197 | 1.2025 |
|  | 2 | 1.1733 | .01596 | 1.1425 | 1.2050 |

| **Pairwise Comparisons** | | | | | | | | |
| --- | --- | --- | --- | --- | --- | --- | --- | --- |
| Gender | (I) Diagnostic | (J) Diagnostic | Mean Difference (I-J) | Standard Error | Degrees of Freedom | P | 95% Wald Confidence Interval | |
|  |  |  |  |  |  |  | Lower Bound | Upper Bound |
| 1 | 1 | 2 | -.0013 | .01762 | 1 | .941 | -.0358 | .0332 |
|  | 2 | 1 | .0013 | .01762 | 1 | .941 | -.0332 | .0358 |
| 2 | 1 | 2 | -.0130 | .02399 | 1 | .589 | -.0600 | .0341 |
|  | 2 | 1 | .0130 | .02399 | 1 | .589 | -.0341 | .0600 |

| **Overall Test** | | | |
| --- | --- | --- | --- |
| Gender | Wald χ² | Degrees of Freedom | P |
| 1 | .005 | 1 | .941 |
| 2 | .292 | 1 | .589 |

**Estimated Marginal Means 15：Age* Diagnostic**

| **Estimate** | | | | | |
| --- | --- | --- | --- | --- | --- |
| Age | Diagnostic | Mean | Standard Error | 95% Wald Confidence Interval | |
|  |  |  |  | Lower Bound | Upper Bound |
| 1 | 1 | .6482 | .01502 | .6194 | .6783 |
|  | 2 | .6354 | .00765 | .6206 | .6506 |
| 2 | 1 | 1.0693 | .01697 | 1.0366 | 1.1031 |
|  | 2 | 1.1691 | .01431 | 1.1413 | 1.1975 |
| 3 | 1 | 1.3049 | .03451 | 1.2390 | 1.3744 |
|  | 2 | 1.2765 | .02776 | 1.2232 | 1.3320 |
| 4 | 1 | 1.4455 | .04106 | 1.3673 | 1.5283 |
|  | 2 | 1.4137 | .03646 | 1.3440 | 1.4870 |

| **Pairwise Comparisons** | | | | | | | | |
| --- | --- | --- | --- | --- | --- | --- | --- | --- |
| Diagnostic | (I) Age | (J) Age | Mean Difference (I-J) | Standard Error | Degrees of Freedom | P | 95% Wald Confidence Interval | |
|  |  |  |  |  |  |  | Lower Bound | Upper Bound |
| 1 | 1 | 2 | -.4211 | .02162 | 1 | .000 | -.4635 | -.3787 |
|  |  | 3 | -.6567 | .03667 | 1 | .000 | -.7286 | -.5848 |
|  |  | 4 | -.7973 | .04322 | 1 | .000 | -.8820 | -.7126 |
|  | 2 | 1 | .4211 | .02162 | 1 | .000 | .3787 | .4635 |
|  |  | 3 | -.2356 | .03723 | 1 | .000 | -.3086 | -.1626 |
|  |  | 4 | -.3762 | .04387 | 1 | .000 | -.4622 | -.2902 |
|  | 3 | 1 | .6567 | .03667 | 1 | .000 | .5848 | .7286 |
|  |  | 2 | .2356 | .03723 | 1 | .000 | .1626 | .3086 |
|  |  | 4 | -.1406 | .05289 | 1 | .008 | -.2443 | -.0369 |
|  | 4 | 1 | .7973 | .04322 | 1 | .000 | .7126 | .8820 |
|  |  | 2 | .3762 | .04387 | 1 | .000 | .2902 | .4622 |
|  |  | 3 | .1406 | .05289 | 1 | .008 | .0369 | .2443 |
| 2 | 1 | 2 | -.5337 | .01588 | 1 | .000 | -.5648 | -.5026 |
|  |  | 3 | -.6411 | .02852 | 1 | .000 | -.6970 | -.5852 |
|  |  | 4 | -.7783 | .03691 | 1 | .000 | -.8507 | -.7060 |
|  | 2 | 1 | .5337 | .01588 | 1 | .000 | .5026 | .5648 |
|  |  | 3 | -.1074 | .03041 | 1 | .000 | -.1670 | -.0478 |
|  |  | 4 | -.2447 | .03795 | 1 | .000 | -.3190 | -.1703 |
|  | 3 | 1 | .6411 | .02852 | 1 | .000 | .5852 | .6970 |
|  |  | 2 | .1074 | .03041 | 1 | .000 | .0478 | .1670 |
|  |  | 4 | -.1373 | .04454 | 1 | .002 | -.2246 | -.0500 |
|  | 4 | 1 | .7783 | .03691 | 1 | .000 | .7060 | .8507 |
|  |  | 2 | .2447 | .03795 | 1 | .000 | .1703 | .3190 |
|  |  | 3 | .1373 | .04454 | 1 | .002 | .0500 | .2246 |

| **Overall Test** | | | |
| --- | --- | --- | --- |
| Diagnostic | Wald χ² | Degrees of Freedom | P |
| 1 | 736.466 | 3 | .000 |
| 2 | 1661.867 | 3 | .000 |

**Estimated Marginal Means 16：Age* Diagnostic**

| **Estimate** | | | | | |
| --- | --- | --- | --- | --- | --- |
| Age | Diagnostic | Mean | Standard Error | 95% Wald Confidence Interval | |
|  |  |  |  | Lower Bound | Upper Bound |
| 1 | 1 | .6482 | .01502 | .6194 | .6783 |
|  | 2 | .6354 | .00765 | .6206 | .6506 |
| 2 | 1 | 1.0693 | .01697 | 1.0366 | 1.1031 |
|  | 2 | 1.1691 | .01431 | 1.1413 | 1.1975 |
| 3 | 1 | 1.3049 | .03451 | 1.2390 | 1.3744 |
|  | 2 | 1.2765 | .02776 | 1.2232 | 1.3320 |
| 4 | 1 | 1.4455 | .04106 | 1.3673 | 1.5283 |
|  | 2 | 1.4137 | .03646 | 1.3440 | 1.4870 |

| **Pairwise Comparisons** | | | | | | | | |
| --- | --- | --- | --- | --- | --- | --- | --- | --- |
| Age | (I) Diagnostic | (J) Diagnostic | Mean Difference (I-J) | Standard Error | Degrees of Freedom | P | 95% Wald Confidence Interval | |
|  |  |  |  |  |  |  | Lower Bound | Upper Bound |
| 1 | 1 | 2 | .0128 | .01608 | 1 | .425 | -.0187 | .0444 |
|  | 2 | 1 | -.0128 | .01608 | 1 | .425 | -.0444 | .0187 |
| 2 | 1 | 2 | -.0997 | .02088 | 1 | .000 | -.1407 | -.0588 |
|  | 2 | 1 | .0997 | .02088 | 1 | .000 | .0588 | .1407 |
| 3 | 1 | 2 | .0285 | .03432 | 1 | .407 | -.0388 | .0957 |
|  | 2 | 1 | -.0285 | .03432 | 1 | .407 | -.0957 | .0388 |
| 4 | 1 | 2 | .0318 | .04885 | 1 | .515 | -.0639 | .1276 |
|  | 2 | 1 | -.0318 | .04885 | 1 | .515 | -.1276 | .0639 |

| **Overall Test** | | | |
| --- | --- | --- | --- |
| Age | Wald χ² | Degrees of Freedom | P |
| 1 | .638 | 1 | .425 |
| 2 | 22.808 | 1 | .000 |
| 3 | .688 | 1 | .407 |
| 4 | .424 | 1 | .515 |
