# Supplementary material for: The impact of COVID-19 social isolation and reduced microbial exposure on the immune system in children: a retrospective study
Source: PeerJ. 2026 Jul 7;14:e21469. doi: 10.7717/peerj.21469 (PMC13353229; doi:10.7717/peerj.21469)
Supplement: Supplemental Information 11 [file peerj-14-21469-s011.docx]

**Immunoglobulin G Generalized Linear Model**

For immunoglobulin analysis, patients were grouped as follows:
Group 1: 0–1 year,
Group 2: 1–4 years,
Group 3: 4–6 years,
Group 4: 6–13 years.

| **Parameter Estimate** | | | | | | | |
| --- | --- | --- | --- | --- | --- | --- | --- |
| Parameter | B | Standard Error | 95% Wald Confidence Interval | | Hypothesis Testing | | |
|  |  |  | Lower Bound | Upper Bound | Wald χ² | Degrees of Freedom | P |
| （Intercept） | 2.266 | .0386 | 2.190 | 2.342 | 3450.239 | 1 | .000 |
| [Year=2020] | .079 | .0775 | -.073 | .230 | 1.026 | 1 | .311 |
| [Year=2021] | -.027 | .0568 | -.139 | .084 | .230 | 1 | .631 |
| [Year=2022] | -.019 | .0496 | -.116 | .078 | .144 | 1 | .705 |
| [Year=2023] | .044 | .0404 | -.035 | .123 | 1.196 | 1 | .274 |
| [Year=2024] | .083 | .0407 | .003 | .162 | 4.113 | 1 | .043 |
| [Year=2025] | 0 | . | . | . | . | . | . |
| [Gender=1] | -.010 | .0286 | -.066 | .046 | .134 | 1 | .715 |
| [Gender=2] | 0 | . | . | . | . | . | . |
| [Age=1] | -.751 | .0408 | -.831 | -.671 | 339.557 | 1 | .000 |
| [Age=2] | -.293 | .0393 | -.370 | -.216 | 55.429 | 1 | .000 |
| [Age=3] | -.161 | .0441 | -.247 | -.074 | 13.289 | 1 | .000 |
| [Age=4] | 0 | . | . | . | . | . | . |
| [Diagnostic=1] | .020 | .0314 | -.042 | .082 | .406 | 1 | .524 |
| [Diagnostic=2] | 0 | . | . | . | . | . | . |
| [Year=2020] * [Gender=1] | -.037 | .0448 | -.125 | .051 | .685 | 1 | .408 |
| [Year=2020] * [Gender=2] | 0 | . | . | . | . | . | . |
| [Year=2021] * [Gender=1] | -.009 | .0315 | -.071 | .052 | .089 | 1 | .766 |
| [Year=2021] * [Gender=2] | 0 | . | . | . | . | . | . |
| [Year=2022] * [Gender=1] | -.010 | .0241 | -.057 | .038 | .164 | 1 | .686 |
| [Year=2022] * [Gender=2] | 0 | . | . | . | . | . | . |
| [Year=2023] * [Gender=1] | -.017 | .0238 | -.063 | .030 | .493 | 1 | .482 |
| [Year=2023] * [Gender=2] | 0 | . | . | . | . | . | . |
| [Year=2024] * [Gender=1] | -.030 | .0233 | -.076 | .016 | 1.680 | 1 | .195 |
| [Year=2024] * [Gender=2] | 0 | . | . | . | . | . | . |
| [Year=2025] * [Gender=1] | 0 | . | . | . | . | . | . |
| [Year=2025] * [Gender=2] | 0 | . | . | . | . | . | . |
| [Year=2020] * [Age=1] | -.032 | .0772 | -.183 | .120 | .169 | 1 | .681 |
| [Year=2020] * [Age=2] | -.033 | .0750 | -.180 | .114 | .189 | 1 | .664 |
| [Year=2020] * [Age=3] | -.011 | .1006 | -.208 | .186 | .011 | 1 | .916 |
| [Year=2020] * [Age=4] | 0 | . | . | . | . | . | . |
| [Year=2021] * [Age=1] | -.009 | .0586 | -.123 | .106 | .021 | 1 | .884 |
| [Year=2021] * [Age=2] | .069 | .0566 | -.041 | .180 | 1.506 | 1 | .220 |
| [Year=2021] * [Age=3] | .073 | .0630 | -.050 | .197 | 1.355 | 1 | .244 |
| [Year=2021] * [Age=4] | 0 | . | . | . | . | . | . |
| [Year=2022] * [Age=1] | -.015 | .0511 | -.116 | .085 | .092 | 1 | .762 |
| [Year=2022] * [Age=2] | .023 | .0497 | -.075 | .120 | .211 | 1 | .646 |
| [Year=2022] * [Age=3] | .051 | .0548 | -.056 | .159 | .873 | 1 | .350 |
| [Year=2022] * [Age=4] | 0 | . | . | . | . | . | . |
| [Year=2023] * [Age=1] | -.004 | .0427 | -.088 | .079 | .011 | 1 | .917 |
| [Year=2023] * [Age=2] | .019 | .0406 | -.061 | .098 | .211 | 1 | .646 |
| [Year=2023] * [Age=3] | .031 | .0459 | -.059 | .121 | .451 | 1 | .502 |
| [Year=2023] * [Age=4] | 0 | . | . | . | . | . | . |
| [Year=2024] * [Age=1] | -.038 | .0424 | -.121 | .045 | .799 | 1 | .371 |
| [Year=2024] * [Age=2] | -.004 | .0411 | -.085 | .077 | .010 | 1 | .921 |
| [Year=2024] * [Age=3] | .028 | .0464 | -.063 | .119 | .361 | 1 | .548 |
| [Year=2024] * [Age=4] | 0 | . | . | . | . | . | . |
| [Year=2025] * [Age=1] | 0 | . | . | . | . | . | . |
| [Year=2025] * [Age=2] | 0 | . | . | . | . | . | . |
| [Year=2025] * [Age=3] | 0 | . | . | . | . | . | . |
| [Year=2025] * [Age=4] | 0 | . | . | . | . | . | . |
| [Year=2020] * [Diagnostic=1] | -.077 | .0468 | -.169 | .015 | 2.687 | 1 | .101 |
| [Year=2020] * [Diagnostic=2] | 0 | . | . | . | . | . | . |
| [Year=2021] * [Diagnostic=1] | .034 | .0338 | -.032 | .100 | 1.022 | 1 | .312 |
| [Year=2021] * [Diagnostic=2] | 0 | . | . | . | . | . | . |
| [Year=2022] * [Diagnostic=1] | -.008 | .0267 | -.060 | .045 | .082 | 1 | .774 |
| [Year=2022] * [Diagnostic=2] | 0 | . | . | . | . | . | . |
| [Year=2023] * [Diagnostic=1] | -.043 | .0254 | -.092 | .007 | 2.796 | 1 | .094 |
| [Year=2023] * [Diagnostic=2] | 0 | . | . | . | . | . | . |
| [Year=2024] * [Diagnostic=1] | -.058 | .0258 | -.108 | -.007 | 4.992 | 1 | .025 |
| [Year=2024] * [Diagnostic=2] | 0 | . | . | . | . | . | . |
| [Year=2025] * [Diagnostic=1] | 0 | . | . | . | . | . | . |
| [Year=2025] * [Diagnostic=2] | 0 | . | . | . | . | . | . |
| [Gender=1] * [Age=1] | -.011 | .0246 | -.059 | .037 | .192 | 1 | .662 |
| [Gender=1] * [Age=2] | -.039 | .0236 | -.085 | .008 | 2.697 | 1 | .101 |
| [Gender=1] * [Age=3] | -.012 | .0261 | -.064 | .039 | .228 | 1 | .633 |
| [Gender=1] * [Age=4] | 0 | . | . | . | . | . | . |
| [Gender=2] * [Age=1] | 0 | . | . | . | . | . | . |
| [Gender=2] * [Age=2] | 0 | . | . | . | . | . | . |
| [Gender=2] * [Age=3] | 0 | . | . | . | . | . | . |
| [Gender=2] * [Age=4] | 0 | . | . | . | . | . | . |
| [Gender=1] * [Diagnostic=1] | .035 | .0160 | .004 | .066 | 4.756 | 1 | .029 |
| [Gender=1] * [Diagnostic=2] | 0 | . | . | . | . | . | . |
| [Gender=2] * [Diagnostic=1] | 0 | . | . | . | . | . | . |
| [Gender=2] * [Diagnostic=2] | 0 | . | . | . | . | . | . |
| [Age=1] * [Diagnostic=1] | -.002 | .0284 | -.057 | .054 | .004 | 1 | .949 |
| [Age=1] * [Diagnostic=2] | 0 | . | . | . | . | . | . |
| [Age=2] * [Diagnostic=1] | -.072 | .0259 | -.123 | -.021 | 7.788 | 1 | .005 |
| [Age=2] * [Diagnostic=2] | 0 | . | . | . | . | . | . |
| [Age=3] * [Diagnostic=1] | .014 | .0287 | -.042 | .070 | .233 | 1 | .629 |
| [Age=3] * [Diagnostic=2] | 0 | . | . | . | . | . | . |
| [Age=4] * [Diagnostic=1] | 0 | . | . | . | . | . | . |
| [Age=4] * [Diagnostic=2] | 0 | . | . | . | . | . | . |
| （标度） | .090 | .0014 | .088 | .093 |  |  |  |

**Estimated Marginal Means 1：Year**

| **Estimate** | | | | |
| --- | --- | --- | --- | --- |
| Year | Mean | Standard Error | 95% Wald Confidence Interval | |
|  |  |  | Lower Bound | Upper Bound |
| 2020 | 7.1418 | .18468 | 6.7888 | 7.5131 |
| 2021 | 7.2555 | .11183 | 7.0396 | 7.4781 |
| 2022 | 7.0301 | .07838 | 6.8782 | 7.1854 |
| 2023 | 7.3076 | .05818 | 7.1945 | 7.4225 |
| 2024 | 7.3755 | .06183 | 7.2553 | 7.4977 |
| 2025 | 7.1213 | .08379 | 6.9590 | 7.2874 |

| **Pairwise Comparisons** | | | | | | | |
| --- | --- | --- | --- | --- | --- | --- | --- |
| (I) Year | (J) Year | Mean Difference (I-J) | Standard Error | Degrees of Freedom | P | 95% Wald Confidence Interval | |
|  |  |  |  |  |  | Lower Bound | Upper Bound |
| 2020 | 2021 | -.1138 | .21571 | 1 | .598 | -.5365 | .3090 |
|  | 2022 | .1117 | .20060 | 1 | .578 | -.2815 | .5048 |
|  | 2023 | -.1658 | .19363 | 1 | .392 | -.5454 | .2137 |
|  | 2024 | -.2338 | .19489 | 1 | .230 | -.6157 | .1482 |
|  | 2025 | .0205 | .20250 | 1 | .920 | -.3764 | .4174 |
| 2021 | 2020 | .1138 | .21571 | 1 | .598 | -.3090 | .5365 |
|  | 2022 | .2254 | .13573 | 1 | .097 | -.0406 | .4914 |
|  | 2023 | -.0521 | .12555 | 1 | .678 | -.2982 | .1940 |
|  | 2024 | -.1200 | .12725 | 1 | .346 | -.3694 | .1294 |
|  | 2025 | .1342 | .13932 | 1 | .335 | -.1388 | .4073 |
| 2022 | 2020 | -.1117 | .20060 | 1 | .578 | -.5048 | .2815 |
|  | 2021 | -.2254 | .13573 | 1 | .097 | -.4914 | .0406 |
|  | 2023 | -.2775 | .09692 | 1 | .004 | -.4675 | -.0875 |
|  | 2024 | -.3454 | .09905 | 1 | .000 | -.5395 | -.1513 |
|  | 2025 | -.0912 | .11439 | 1 | .425 | -.3154 | .1330 |
| 2023 | 2020 | .1658 | .19363 | 1 | .392 | -.2137 | .5454 |
|  | 2021 | .0521 | .12555 | 1 | .678 | -.1940 | .2982 |
|  | 2022 | .2775 | .09692 | 1 | .004 | .0875 | .4675 |
|  | 2024 | -.0679 | .08431 | 1 | .421 | -.2332 | .0973 |
|  | 2025 | .1863 | .10181 | 1 | .067 | -.0132 | .3859 |
| 2024 | 2020 | .2338 | .19489 | 1 | .230 | -.1482 | .6157 |
|  | 2021 | .1200 | .12725 | 1 | .346 | -.1294 | .3694 |
|  | 2022 | .3454 | .09905 | 1 | .000 | .1513 | .5395 |
|  | 2023 | .0679 | .08431 | 1 | .421 | -.0973 | .2332 |
|  | 2025 | .2542 | .10400 | 1 | .015 | .0504 | .4581 |
| 2025 | 2020 | -.0205 | .20250 | 1 | .920 | -.4174 | .3764 |
|  | 2021 | -.1342 | .13932 | 1 | .335 | -.4073 | .1388 |
|  | 2022 | .0912 | .11439 | 1 | .425 | -.1330 | .3154 |
|  | 2023 | -.1863 | .10181 | 1 | .067 | -.3859 | .0132 |
|  | 2024 | -.2542 | .10400 | 1 | .015 | -.4581 | -.0504 |

| **Overall Test** | | |
| --- | --- | --- |
| Wald χ² | Degrees of Freedom | P |
| 15.812 | 5 | .007 |

**Estimated Marginal Means 2：Gender**

| **Estimate** | | | | |
| --- | --- | --- | --- | --- |
| Gender | Mean | Standard Error | 95% Wald Confidence Interval | |
|  |  |  | Lower Bound | Upper Bound |
| 1 | 7.1124 | .05183 | 7.0115 | 7.2147 |
| 2 | 7.2975 | .06424 | 7.1727 | 7.4245 |

| **Pairwise Comparisons** | | | | | | | |
| --- | --- | --- | --- | --- | --- | --- | --- |
| (I) Gender | (J) Gender | Mean Difference (I-J) | Standard Error | Degrees of Freedom | P | 95% Wald Confidence Interval | |
|  |  |  |  |  |  | Lower Bound | Upper Bound |
| 1 | 2 | -.1852 | .07704 | 1 | .016 | -.3362 | -.0342 |
| 2 | 1 | .1852 | .07704 | 1 | .016 | .0342 | .3362 |

| **Overall Test** | | |
| --- | --- | --- |
| Wald χ² | Degrees of Freedom | P |
| 5.777 | 1 | .016 |

**Estimated Marginal Means 3：Age**

| **Estimate** | | | | |
| --- | --- | --- | --- | --- |
| Age | Mean | Standard Error | 95% Wald Confidence Interval | |
|  |  |  | Lower Bound | Upper Bound |
| 1 | 4.5326 | .04413 | 4.4469 | 4.6200 |
| 2 | 7.0227 | .05261 | 6.9204 | 7.1266 |
| 3 | 8.6144 | .12428 | 8.3742 | 8.8614 |
| 4 | 9.8243 | .14711 | 9.5401 | 10.1169 |

| **Pairwise Comparisons** | | | | | | | |
| --- | --- | --- | --- | --- | --- | --- | --- |
| (I) Age | (J) Age | Mean Difference (I-J) | Standard Error | Degrees of Freedom | P | 95% Wald Confidence Interval | |
|  |  |  |  |  |  | Lower Bound | Upper Bound |
| 1 | 2 | -2.4901 | .06847 | 1 | .000 | -2.6243 | -2.3559 |
|  | 3 | -4.0817 | .13105 | 1 | .000 | -4.3386 | -3.8249 |
|  | 4 | -5.2916 | .15367 | 1 | .000 | -5.5928 | -4.9904 |
| 2 | 1 | 2.4901 | .06847 | 1 | .000 | 2.3559 | 2.6243 |
|  | 3 | -1.5916 | .13487 | 1 | .000 | -1.8560 | -1.3273 |
|  | 4 | -2.8015 | .15610 | 1 | .000 | -3.1075 | -2.4956 |
| 3 | 1 | 4.0817 | .13105 | 1 | .000 | 3.8249 | 4.3386 |
|  | 2 | 1.5916 | .13487 | 1 | .000 | 1.3273 | 1.8560 |
|  | 4 | -1.2099 | .19262 | 1 | .000 | -1.5874 | -.8324 |
| 4 | 1 | 5.2916 | .15367 | 1 | .000 | 4.9904 | 5.5928 |
|  | 2 | 2.8015 | .15610 | 1 | .000 | 2.4956 | 3.1075 |
|  | 3 | 1.2099 | .19262 | 1 | .000 | .8324 | 1.5874 |

| **Overall Test** | | |
| --- | --- | --- |
| Wald χ² | Degrees of Freedom | P |
| 2628.865 | 3 | .000 |

**Estimated Marginal Means 4：Diagnostic**

| **Estimate** | | | | |
| --- | --- | --- | --- | --- |
| Diagnostic | Mean | Standard Error | 95% Wald Confidence Interval | |
|  |  |  | Lower Bound | Upper Bound |
| 1 | 7.1948 | .06588 | 7.0668 | 7.3251 |
| 2 | 7.2139 | .05143 | 7.1138 | 7.3154 |

| **Pairwise Comparisons** | | | | | | | |
| --- | --- | --- | --- | --- | --- | --- | --- |
| (I) Diagnostic | (J) Diagnostic | Mean Difference (I-J) | Standard Error | Degrees of Freedom | P | 95% Wald Confidence Interval | |
|  |  |  |  |  |  | Lower Bound | Upper Bound |
| 1 | 2 | -.0191 | .07949 | 1 | .810 | -.1749 | .1367 |
| 2 | 1 | .0191 | .07949 | 1 | .810 | -.1367 | .1749 |

| **Overall Test** | | |
| --- | --- | --- |
| Wald χ² | Degrees of Freedom | P |
| .058 | 1 | .810 |

**Estimated Marginal Means 5：Year* Gender**

| **Estimate** | | | | | |
| --- | --- | --- | --- | --- | --- |
| Year | Gender | Mean | Standard Error | 95% Wald Confidence Interval | |
|  |  |  |  | Lower Bound | Upper Bound |
| 2020 | 1 | 6.9809 | .21026 | 6.5807 | 7.4054 |
|  | 2 | 7.3064 | .25911 | 6.8158 | 7.8323 |
| 2021 | 1 | 7.1910 | .12388 | 6.9522 | 7.4379 |
|  | 2 | 7.3207 | .16401 | 7.0062 | 7.6493 |
| 2022 | 1 | 6.9662 | .08906 | 6.7938 | 7.1430 |
|  | 2 | 7.0946 | .10272 | 6.8961 | 7.2988 |
| 2023 | 1 | 7.2160 | .07100 | 7.0782 | 7.3565 |
|  | 2 | 7.4004 | .08557 | 7.2345 | 7.5700 |
| 2024 | 1 | 7.2339 | .07376 | 7.0908 | 7.3800 |
|  | 2 | 7.5199 | .08733 | 7.3506 | 7.6930 |
| 2025 | 1 | 7.0911 | .10200 | 6.8940 | 7.2939 |
|  | 2 | 7.1517 | .11699 | 6.9260 | 7.3847 |

| **Pairwise Comparisons** | | | | | | | | |
| --- | --- | --- | --- | --- | --- | --- | --- | --- |
| Gender | (I) Year | (J) Year | Mean Difference (I-J) | Standard Error | Degrees of Freedom | P | 95% Wald Confidence Interval | |
|  |  |  |  |  |  |  | Lower Bound | Upper Bound |
| 1 | 2020 | 2021 | -.2101 | .24333 | 1 | .388 | -.6870 | .2668 |
|  |  | 2022 | .0146 | .22718 | 1 | .949 | -.4306 | .4599 |
|  |  | 2023 | -.2352 | .22122 | 1 | .288 | -.6688 | .1984 |
|  |  | 2024 | -.2531 | .22190 | 1 | .254 | -.6880 | .1818 |
|  |  | 2025 | -.1102 | .23253 | 1 | .635 | -.5660 | .3455 |
|  | 2021 | 2020 | .2101 | .24333 | 1 | .388 | -.2668 | .6870 |
|  |  | 2022 | .2247 | .15099 | 1 | .137 | -.0712 | .5207 |
|  |  | 2023 | -.0251 | .14159 | 1 | .859 | -.3026 | .2524 |
|  |  | 2024 | -.0430 | .14292 | 1 | .764 | -.3231 | .2371 |
|  |  | 2025 | .0999 | .15923 | 1 | .531 | -.2122 | .4120 |
|  | 2022 | 2020 | -.0146 | .22718 | 1 | .949 | -.4599 | .4306 |
|  |  | 2021 | -.2247 | .15099 | 1 | .137 | -.5207 | .0712 |
|  |  | 2023 | -.2498 | .11139 | 1 | .025 | -.4681 | -.0315 |
|  |  | 2024 | -.2677 | .11268 | 1 | .018 | -.4886 | -.0469 |
|  |  | 2025 | -.1249 | .13317 | 1 | .348 | -.3859 | .1361 |
|  | 2023 | 2020 | .2352 | .22122 | 1 | .288 | -.1984 | .6688 |
|  |  | 2021 | .0251 | .14159 | 1 | .859 | -.2524 | .3026 |
|  |  | 2022 | .2498 | .11139 | 1 | .025 | .0315 | .4681 |
|  |  | 2024 | -.0179 | .09991 | 1 | .858 | -.2137 | .1779 |
|  |  | 2025 | .1249 | .12269 | 1 | .309 | -.1155 | .3654 |
|  | 2024 | 2020 | .2531 | .22190 | 1 | .254 | -.1818 | .6880 |
|  |  | 2021 | .0430 | .14292 | 1 | .764 | -.2371 | .3231 |
|  |  | 2022 | .2677 | .11268 | 1 | .018 | .0469 | .4886 |
|  |  | 2023 | .0179 | .09991 | 1 | .858 | -.1779 | .2137 |
|  |  | 2025 | .1429 | .12407 | 1 | .250 | -.1003 | .3860 |
|  | 2025 | 2020 | .1102 | .23253 | 1 | .635 | -.3455 | .5660 |
|  |  | 2021 | -.0999 | .15923 | 1 | .531 | -.4120 | .2122 |
|  |  | 2022 | .1249 | .13317 | 1 | .348 | -.1361 | .3859 |
|  |  | 2023 | -.1249 | .12269 | 1 | .309 | -.3654 | .1155 |
|  |  | 2024 | -.1429 | .12407 | 1 | .250 | -.3860 | .1003 |
| 2 | 2020 | 2021 | -.0143 | .30501 | 1 | .963 | -.6121 | .5835 |
|  |  | 2022 | .2118 | .27713 | 1 | .445 | -.3314 | .7550 |
|  |  | 2023 | -.0940 | .27212 | 1 | .730 | -.6273 | .4394 |
|  |  | 2024 | -.2135 | .27250 | 1 | .433 | -.7476 | .3206 |
|  |  | 2025 | .1547 | .28284 | 1 | .584 | -.3996 | .7091 |
|  | 2021 | 2020 | .0143 | .30501 | 1 | .963 | -.5835 | .6121 |
|  |  | 2022 | .2261 | .18877 | 1 | .231 | -.1439 | .5961 |
|  |  | 2023 | -.0797 | .18211 | 1 | .662 | -.4366 | .2772 |
|  |  | 2024 | -.1992 | .18192 | 1 | .274 | -.5558 | .1573 |
|  |  | 2025 | .1690 | .19848 | 1 | .394 | -.2200 | .5580 |
|  | 2022 | 2020 | -.2118 | .27713 | 1 | .445 | -.7550 | .3314 |
|  |  | 2021 | -.2261 | .18877 | 1 | .231 | -.5961 | .1439 |
|  |  | 2023 | -.3058 | .12980 | 1 | .018 | -.5602 | -.0514 |
|  |  | 2024 | -.4253 | .12948 | 1 | .001 | -.6791 | -.1715 |
|  |  | 2025 | -.0571 | .15200 | 1 | .707 | -.3550 | .2409 |
|  | 2023 | 2020 | .0940 | .27212 | 1 | .730 | -.4394 | .6273 |
|  |  | 2021 | .0797 | .18211 | 1 | .662 | -.2772 | .4366 |
|  |  | 2022 | .3058 | .12980 | 1 | .018 | .0514 | .5602 |
|  |  | 2024 | -.1195 | .11855 | 1 | .313 | -.3519 | .1128 |
|  |  | 2025 | .2487 | .14275 | 1 | .081 | -.0311 | .5285 |
|  | 2024 | 2020 | .2135 | .27250 | 1 | .433 | -.3206 | .7476 |
|  |  | 2021 | .1992 | .18192 | 1 | .274 | -.1573 | .5558 |
|  |  | 2022 | .4253 | .12948 | 1 | .001 | .1715 | .6791 |
|  |  | 2023 | .1195 | .11855 | 1 | .313 | -.1128 | .3519 |
|  |  | 2025 | .3682 | .14309 | 1 | .010 | .0878 | .6487 |
|  | 2025 | 2020 | -.1547 | .28284 | 1 | .584 | -.7091 | .3996 |
|  |  | 2021 | -.1690 | .19848 | 1 | .394 | -.5580 | .2200 |
|  |  | 2022 | .0571 | .15200 | 1 | .707 | -.2409 | .3550 |
|  |  | 2023 | -.2487 | .14275 | 1 | .081 | -.5285 | .0311 |
|  |  | 2024 | -.3682 | .14309 | 1 | .010 | -.6487 | -.0878 |

| **Overall Test** | | | |
| --- | --- | --- | --- |
| Gender | Wald χ² | Degrees of Freedom | P |
| 1 | 7.820 | 5 | .166 |
| 2 | 13.863 | 5 | .017 |

**Estimated Marginal Means 6：Year* Gender**

| **Estimate** | | | | | |
| --- | --- | --- | --- | --- | --- |
| Year | Gender | Mean | Standard Error | 95% Wald Confidence Interval | |
|  |  |  |  | Lower Bound | Upper Bound |
| 2020 | 1 | 6.9809 | .21026 | 6.5807 | 7.4054 |
|  | 2 | 7.3064 | .25911 | 6.8158 | 7.8323 |
| 2021 | 1 | 7.1910 | .12388 | 6.9522 | 7.4379 |
|  | 2 | 7.3207 | .16401 | 7.0062 | 7.6493 |
| 2022 | 1 | 6.9662 | .08906 | 6.7938 | 7.1430 |
|  | 2 | 7.0946 | .10272 | 6.8961 | 7.2988 |
| 2023 | 1 | 7.2160 | .07100 | 7.0782 | 7.3565 |
|  | 2 | 7.4004 | .08557 | 7.2345 | 7.5700 |
| 2024 | 1 | 7.2339 | .07376 | 7.0908 | 7.3800 |
|  | 2 | 7.5199 | .08733 | 7.3506 | 7.6930 |
| 2025 | 1 | 7.0911 | .10200 | 6.8940 | 7.2939 |
|  | 2 | 7.1517 | .11699 | 6.9260 | 7.3847 |

| **Pairwise Comparisons** | | | | | | | | |
| --- | --- | --- | --- | --- | --- | --- | --- | --- |
| Year | (I) Gender | (J) Gender | Mean Difference (I-J) | Standard Error | Degrees of Freedom | P | 95% Wald Confidence Interval | |
|  |  |  |  |  |  |  | Lower Bound | Upper Bound |
| 2020 | 1 | 2 | -.3255 | .29214 | 1 | .265 | -.8981 | .2471 |
|  | 2 | 1 | .3255 | .29214 | 1 | .265 | -.2471 | .8981 |
| 2021 | 1 | 2 | -.1297 | .18512 | 1 | .483 | -.4925 | .2331 |
|  | 2 | 1 | .1297 | .18512 | 1 | .483 | -.2331 | .4925 |
| 2022 | 1 | 2 | -.1284 | .11113 | 1 | .248 | -.3462 | .0894 |
|  | 2 | 1 | .1284 | .11113 | 1 | .248 | -.0894 | .3462 |
| 2023 | 1 | 2 | -.1843 | .10552 | 1 | .081 | -.3912 | .0225 |
|  | 2 | 1 | .1843 | .10552 | 1 | .081 | -.0225 | .3912 |
| 2024 | 1 | 2 | -.2859 | .10375 | 1 | .006 | -.4893 | -.0826 |
|  | 2 | 1 | .2859 | .10375 | 1 | .006 | .0826 | .4893 |
| 2025 | 1 | 2 | -.0606 | .14169 | 1 | .669 | -.3383 | .2171 |
|  | 2 | 1 | .0606 | .14169 | 1 | .669 | -.2171 | .3383 |

| **Overall Test** | | | |
| --- | --- | --- | --- |
| Year | Wald χ² | Degrees of Freedom | P |
| 2020 | 1.242 | 1 | .265 |
| 2021 | .491 | 1 | .483 |
| 2022 | 1.334 | 1 | .248 |
| 2023 | 3.052 | 1 | .081 |
| 2024 | 7.595 | 1 | .006 |
| 2025 | .183 | 1 | .669 |

**Estimated Marginal Means 7：Year* Age**

| **Estimate** | | | | | |
| --- | --- | --- | --- | --- | --- |
| Year | Age | Mean | Standard Error | 95% Wald Confidence Interval | |
|  |  |  |  | Lower Bound | Upper Bound |
| 2020 | 1 | 4.5365 | .15255 | 4.2471 | 4.8455 |
|  | 2 | 6.8237 | .20971 | 6.4248 | 7.2474 |
|  | 3 | 8.4167 | .60058 | 7.3182 | 9.6801 |
|  | 4 | 9.9848 | .58470 | 8.9022 | 11.1992 |
| 2021 | 1 | 4.4765 | .10836 | 4.2691 | 4.6940 |
|  | 2 | 7.2858 | .14249 | 7.0118 | 7.5705 |
|  | 3 | 8.8260 | .24685 | 8.3552 | 9.3233 |
|  | 4 | 9.6272 | .39141 | 8.8898 | 10.4258 |
| 2022 | 1 | 4.3897 | .06580 | 4.2626 | 4.5206 |
|  | 2 | 6.8668 | .07977 | 6.7122 | 7.0249 |
|  | 3 | 8.5244 | .14933 | 8.2367 | 8.8222 |
|  | 4 | 9.5061 | .32081 | 8.8977 | 10.1561 |
| 2023 | 1 | 4.6291 | .07095 | 4.4921 | 4.7703 |
|  | 2 | 7.1321 | .08200 | 6.9732 | 7.2947 |
|  | 3 | 8.7118 | .13133 | 8.4582 | 8.9731 |
|  | 4 | 9.9146 | .17633 | 9.5749 | 10.2663 |
| 2024 | 1 | 4.5856 | .06267 | 4.4644 | 4.7101 |
|  | 2 | 7.1414 | .08385 | 6.9789 | 7.3076 |
|  | 3 | 8.8975 | .13889 | 8.6294 | 9.1739 |
|  | 4 | 10.1561 | .19421 | 9.7825 | 10.5439 |
| 2025 | 1 | 4.5826 | .08320 | 4.4224 | 4.7486 |
|  | 2 | 6.8990 | .10192 | 6.7021 | 7.1017 |
|  | 3 | 8.3251 | .20117 | 7.9401 | 8.7289 |
|  | 4 | 9.7714 | .31014 | 9.1820 | 10.3985 |

| **Pairwise Comparisons** | | | | | | | | |
| --- | --- | --- | --- | --- | --- | --- | --- | --- |
| Age | (I) Year | (J) Year | Mean Difference (I-J) | Standard Error | Degrees of Freedom | P | 95% Wald Confidence Interval | |
|  |  |  |  |  |  |  | Lower Bound | Upper Bound |
| 1 | 2020 | 2021 | .0600 | .18470 | 1 | .745 | -.3020 | .4220 |
|  |  | 2022 | .1468 | .16337 | 1 | .369 | -.1734 | .4670 |
|  |  | 2023 | -.0927 | .16577 | 1 | .576 | -.4176 | .2322 |
|  |  | 2024 | -.0492 | .16235 | 1 | .762 | -.3674 | .2691 |
|  |  | 2025 | -.0461 | .17179 | 1 | .789 | -.3828 | .2906 |
|  | 2021 | 2020 | -.0600 | .18470 | 1 | .745 | -.4220 | .3020 |
|  |  | 2022 | .0868 | .12150 | 1 | .475 | -.1513 | .3250 |
|  |  | 2023 | -.1526 | .12518 | 1 | .223 | -.3980 | .0927 |
|  |  | 2024 | -.1091 | .12038 | 1 | .365 | -.3451 | .1268 |
|  |  | 2025 | -.1060 | .13320 | 1 | .426 | -.3671 | .1550 |
|  | 2022 | 2020 | -.1468 | .16337 | 1 | .369 | -.4670 | .1734 |
|  |  | 2021 | -.0868 | .12150 | 1 | .475 | -.3250 | .1513 |
|  |  | 2023 | -.2395 | .09069 | 1 | .008 | -.4172 | -.0617 |
|  |  | 2024 | -.1959 | .08388 | 1 | .019 | -.3603 | -.0315 |
|  |  | 2025 | -.1929 | .10147 | 1 | .057 | -.3917 | .0060 |
|  | 2023 | 2020 | .0927 | .16577 | 1 | .576 | -.2322 | .4176 |
|  |  | 2021 | .1526 | .12518 | 1 | .223 | -.0927 | .3980 |
|  |  | 2022 | .2395 | .09069 | 1 | .008 | .0617 | .4172 |
|  |  | 2024 | .0435 | .08891 | 1 | .625 | -.1307 | .2178 |
|  |  | 2025 | .0466 | .10546 | 1 | .659 | -.1601 | .2533 |
|  | 2024 | 2020 | .0492 | .16235 | 1 | .762 | -.2691 | .3674 |
|  |  | 2021 | .1091 | .12038 | 1 | .365 | -.1268 | .3451 |
|  |  | 2022 | .1959 | .08388 | 1 | .019 | .0315 | .3603 |
|  |  | 2023 | -.0435 | .08891 | 1 | .625 | -.2178 | .1307 |
|  |  | 2025 | .0031 | .09989 | 1 | .975 | -.1927 | .1989 |
|  | 2025 | 2020 | .0461 | .17179 | 1 | .789 | -.2906 | .3828 |
|  |  | 2021 | .1060 | .13320 | 1 | .426 | -.1550 | .3671 |
|  |  | 2022 | .1929 | .10147 | 1 | .057 | -.0060 | .3917 |
|  |  | 2023 | -.0466 | .10546 | 1 | .659 | -.2533 | .1601 |
|  |  | 2024 | -.0031 | .09989 | 1 | .975 | -.1989 | .1927 |
| 2 | 2020 | 2021 | -.4621 | .25348 | 1 | .068 | -.9589 | .0347 |
|  |  | 2022 | -.0431 | .22425 | 1 | .848 | -.4826 | .3965 |
|  |  | 2023 | -.3084 | .22506 | 1 | .171 | -.7495 | .1327 |
|  |  | 2024 | -.3177 | .22569 | 1 | .159 | -.7600 | .1247 |
|  |  | 2025 | -.0753 | .23311 | 1 | .747 | -.5322 | .3816 |
|  | 2021 | 2020 | .4621 | .25348 | 1 | .068 | -.0347 | .9589 |
|  |  | 2022 | .4190 | .16241 | 1 | .010 | .1007 | .7373 |
|  |  | 2023 | .1536 | .16345 | 1 | .347 | -.1667 | .4740 |
|  |  | 2024 | .1444 | .16417 | 1 | .379 | -.1774 | .4662 |
|  |  | 2025 | .3868 | .17464 | 1 | .027 | .0445 | .7291 |
|  | 2022 | 2020 | .0431 | .22425 | 1 | .848 | -.3965 | .4826 |
|  |  | 2021 | -.4190 | .16241 | 1 | .010 | -.7373 | -.1007 |
|  |  | 2023 | -.2654 | .11284 | 1 | .019 | -.4865 | -.0442 |
|  |  | 2024 | -.2746 | .11374 | 1 | .016 | -.4975 | -.0517 |
|  |  | 2025 | -.0322 | .12859 | 1 | .802 | -.2843 | .2198 |
|  | 2023 | 2020 | .3084 | .22506 | 1 | .171 | -.1327 | .7495 |
|  |  | 2021 | -.1536 | .16345 | 1 | .347 | -.4740 | .1667 |
|  |  | 2022 | .2654 | .11284 | 1 | .019 | .0442 | .4865 |
|  |  | 2024 | -.0092 | .11510 | 1 | .936 | -.2348 | .2164 |
|  |  | 2025 | .2331 | .12989 | 1 | .073 | -.0214 | .4877 |
|  | 2024 | 2020 | .3177 | .22569 | 1 | .159 | -.1247 | .7600 |
|  |  | 2021 | -.1444 | .16417 | 1 | .379 | -.4662 | .1774 |
|  |  | 2022 | .2746 | .11374 | 1 | .016 | .0517 | .4975 |
|  |  | 2023 | .0092 | .11510 | 1 | .936 | -.2164 | .2348 |
|  |  | 2025 | .2424 | .13084 | 1 | .064 | -.0141 | .4988 |
|  | 2025 | 2020 | .0753 | .23311 | 1 | .747 | -.3816 | .5322 |
|  |  | 2021 | -.3868 | .17464 | 1 | .027 | -.7291 | -.0445 |
|  |  | 2022 | .0322 | .12859 | 1 | .802 | -.2198 | .2843 |
|  |  | 2023 | -.2331 | .12989 | 1 | .073 | -.4877 | .0214 |
|  |  | 2024 | -.2424 | .13084 | 1 | .064 | -.4988 | .0141 |
| 3 | 2020 | 2021 | -.4092 | .64843 | 1 | .528 | -1.6801 | .8617 |
|  |  | 2022 | -.1076 | .61791 | 1 | .862 | -1.3187 | 1.1034 |
|  |  | 2023 | -.2951 | .61384 | 1 | .631 | -1.4982 | .9080 |
|  |  | 2024 | -.4807 | .61496 | 1 | .434 | -1.6861 | .7246 |
|  |  | 2025 | .0916 | .63272 | 1 | .885 | -1.1485 | 1.3317 |
|  | 2021 | 2020 | .4092 | .64843 | 1 | .528 | -.8617 | 1.6801 |
|  |  | 2022 | .3016 | .28649 | 1 | .293 | -.2599 | .8631 |
|  |  | 2023 | .1141 | .27757 | 1 | .681 | -.4299 | .6582 |
|  |  | 2024 | -.0715 | .28043 | 1 | .799 | -.6212 | .4781 |
|  |  | 2025 | .5008 | .31721 | 1 | .114 | -.1209 | 1.1225 |
|  | 2022 | 2020 | .1076 | .61791 | 1 | .862 | -1.1034 | 1.3187 |
|  |  | 2021 | -.3016 | .28649 | 1 | .293 | -.8631 | .2599 |
|  |  | 2023 | -.1875 | .19557 | 1 | .338 | -.5708 | .1959 |
|  |  | 2024 | -.3731 | .19885 | 1 | .061 | -.7628 | .0166 |
|  |  | 2025 | .1993 | .24848 | 1 | .423 | -.2878 | .6863 |
|  | 2023 | 2020 | .2951 | .61384 | 1 | .631 | -.9080 | 1.4982 |
|  |  | 2021 | -.1141 | .27757 | 1 | .681 | -.6582 | .4299 |
|  |  | 2022 | .1875 | .19557 | 1 | .338 | -.1959 | .5708 |
|  |  | 2024 | -.1856 | .18638 | 1 | .319 | -.5509 | .1796 |
|  |  | 2025 | .3867 | .23839 | 1 | .105 | -.0805 | .8539 |
|  | 2024 | 2020 | .4807 | .61496 | 1 | .434 | -.7246 | 1.6861 |
|  |  | 2021 | .0715 | .28043 | 1 | .799 | -.4781 | .6212 |
|  |  | 2022 | .3731 | .19885 | 1 | .061 | -.0166 | .7628 |
|  |  | 2023 | .1856 | .18638 | 1 | .319 | -.1796 | .5509 |
|  |  | 2025 | .5724 | .24156 | 1 | .018 | .0989 | 1.0458 |
|  | 2025 | 2020 | -.0916 | .63272 | 1 | .885 | -1.3317 | 1.1485 |
|  |  | 2021 | -.5008 | .31721 | 1 | .114 | -1.1225 | .1209 |
|  |  | 2022 | -.1993 | .24848 | 1 | .423 | -.6863 | .2878 |
|  |  | 2023 | -.3867 | .23839 | 1 | .105 | -.8539 | .0805 |
|  |  | 2024 | -.5724 | .24156 | 1 | .018 | -1.0458 | -.0989 |
| 4 | 2020 | 2021 | .3576 | .70374 | 1 | .611 | -1.0217 | 1.7369 |
|  |  | 2022 | .4788 | .66832 | 1 | .474 | -.8311 | 1.7886 |
|  |  | 2023 | .0703 | .61298 | 1 | .909 | -1.1312 | 1.2717 |
|  |  | 2024 | -.1712 | .61933 | 1 | .782 | -1.3851 | 1.0426 |
|  |  | 2025 | .2135 | .66125 | 1 | .747 | -1.0826 | 1.5095 |
|  | 2021 | 2020 | -.3576 | .70374 | 1 | .611 | -1.7369 | 1.0217 |
|  |  | 2022 | .1211 | .50353 | 1 | .810 | -.8658 | 1.1080 |
|  |  | 2023 | -.2874 | .42635 | 1 | .500 | -1.1230 | .5483 |
|  |  | 2024 | -.5289 | .43308 | 1 | .222 | -1.3777 | .3200 |
|  |  | 2025 | -.1441 | .49846 | 1 | .772 | -1.1211 | .8328 |
|  | 2022 | 2020 | -.4788 | .66832 | 1 | .474 | -1.7886 | .8311 |
|  |  | 2021 | -.1211 | .50353 | 1 | .810 | -1.1080 | .8658 |
|  |  | 2023 | -.4085 | .36135 | 1 | .258 | -1.1167 | .2997 |
|  |  | 2024 | -.6500 | .36872 | 1 | .078 | -1.3727 | .0727 |
|  |  | 2025 | -.2653 | .44594 | 1 | .552 | -1.1393 | .6088 |
|  | 2023 | 2020 | -.0703 | .61298 | 1 | .909 | -1.2717 | 1.1312 |
|  |  | 2021 | .2874 | .42635 | 1 | .500 | -.5483 | 1.1230 |
|  |  | 2022 | .4085 | .36135 | 1 | .258 | -.2997 | 1.1167 |
|  |  | 2024 | -.2415 | .25109 | 1 | .336 | -.7336 | .2506 |
|  |  | 2025 | .1432 | .35681 | 1 | .688 | -.5561 | .8426 |
|  | 2024 | 2020 | .1712 | .61933 | 1 | .782 | -1.0426 | 1.3851 |
|  |  | 2021 | .5289 | .43308 | 1 | .222 | -.3200 | 1.3777 |
|  |  | 2022 | .6500 | .36872 | 1 | .078 | -.0727 | 1.3727 |
|  |  | 2023 | .2415 | .25109 | 1 | .336 | -.2506 | .7336 |
|  |  | 2025 | .3847 | .36606 | 1 | .293 | -.3327 | 1.1022 |
|  | 2025 | 2020 | -.2135 | .66125 | 1 | .747 | -1.5095 | 1.0826 |
|  |  | 2021 | .1441 | .49846 | 1 | .772 | -.8328 | 1.1211 |
|  |  | 2022 | .2653 | .44594 | 1 | .552 | -.6088 | 1.1393 |
|  |  | 2023 | -.1432 | .35681 | 1 | .688 | -.8426 | .5561 |
|  |  | 2024 | -.3847 | .36606 | 1 | .293 | -1.1022 | .3327 |

| **Overall Test** | | | |
| --- | --- | --- | --- |
| Age | Wald χ² | Degrees of Freedom | P |
| 1 | 9.084 | 5 | .106 |
| 2 | 13.583 | 5 | .018 |
| 3 | 7.480 | 5 | .187 |
| 4 | 4.075 | 5 | .539 |

**Estimated Marginal Means 8：Year* Age**

| **Estimate** | | | | | |
| --- | --- | --- | --- | --- | --- |
| Year | Age | Mean | Standard Error | 95% Wald Confidence Interval | |
|  |  |  |  | Lower Bound | Upper Bound |
| 2020 | 1 | 4.5365 | .15255 | 4.2471 | 4.8455 |
|  | 2 | 6.8237 | .20971 | 6.4248 | 7.2474 |
|  | 3 | 8.4167 | .60058 | 7.3182 | 9.6801 |
|  | 4 | 9.9848 | .58470 | 8.9022 | 11.1992 |
| 2021 | 1 | 4.4765 | .10836 | 4.2691 | 4.6940 |
|  | 2 | 7.2858 | .14249 | 7.0118 | 7.5705 |
|  | 3 | 8.8260 | .24685 | 8.3552 | 9.3233 |
|  | 4 | 9.6272 | .39141 | 8.8898 | 10.4258 |
| 2022 | 1 | 4.3897 | .06580 | 4.2626 | 4.5206 |
|  | 2 | 6.8668 | .07977 | 6.7122 | 7.0249 |
|  | 3 | 8.5244 | .14933 | 8.2367 | 8.8222 |
|  | 4 | 9.5061 | .32081 | 8.8977 | 10.1561 |
| 2023 | 1 | 4.6291 | .07095 | 4.4921 | 4.7703 |
|  | 2 | 7.1321 | .08200 | 6.9732 | 7.2947 |
|  | 3 | 8.7118 | .13133 | 8.4582 | 8.9731 |
|  | 4 | 9.9146 | .17633 | 9.5749 | 10.2663 |
| 2024 | 1 | 4.5856 | .06267 | 4.4644 | 4.7101 |
|  | 2 | 7.1414 | .08385 | 6.9789 | 7.3076 |
|  | 3 | 8.8975 | .13889 | 8.6294 | 9.1739 |
|  | 4 | 10.1561 | .19421 | 9.7825 | 10.5439 |
| 2025 | 1 | 4.5826 | .08320 | 4.4224 | 4.7486 |
|  | 2 | 6.8990 | .10192 | 6.7021 | 7.1017 |
|  | 3 | 8.3251 | .20117 | 7.9401 | 8.7289 |
|  | 4 | 9.7714 | .31014 | 9.1820 | 10.3985 |

| **Pairwise Comparisons** | | | | | | | | |
| --- | --- | --- | --- | --- | --- | --- | --- | --- |
| Year | (I) Age | (J) Age | Mean Difference (I-J) | Standard Error | Degrees of Freedom | P | 95% Wald Confidence Interval | |
|  |  |  |  |  |  |  | Lower Bound | Upper Bound |
| 2020 | 1 | 2 | -2.2872 | .25765 | 1 | .000 | -2.7922 | -1.7822 |
|  |  | 3 | -3.8803 | .61286 | 1 | .000 | -5.0814 | -2.6791 |
|  |  | 4 | -5.4484 | .60776 | 1 | .000 | -6.6395 | -4.2572 |
|  | 2 | 1 | 2.2872 | .25765 | 1 | .000 | 1.7822 | 2.7922 |
|  |  | 3 | -1.5931 | .63569 | 1 | .012 | -2.8390 | -.3471 |
|  |  | 4 | -3.1611 | .62300 | 1 | .000 | -4.3822 | -1.9401 |
|  | 3 | 1 | 3.8803 | .61286 | 1 | .000 | 2.6791 | 5.0814 |
|  |  | 2 | 1.5931 | .63569 | 1 | .012 | .3471 | 2.8390 |
|  |  | 4 | -1.5681 | .83958 | 1 | .062 | -3.2136 | .0775 |
|  | 4 | 1 | 5.4484 | .60776 | 1 | .000 | 4.2572 | 6.6395 |
|  |  | 2 | 3.1611 | .62300 | 1 | .000 | 1.9401 | 4.3822 |
|  |  | 3 | 1.5681 | .83958 | 1 | .062 | -.0775 | 3.2136 |
| 2021 | 1 | 2 | -2.8093 | .17005 | 1 | .000 | -3.1426 | -2.4760 |
|  |  | 3 | -4.3495 | .26306 | 1 | .000 | -4.8651 | -3.8339 |
|  |  | 4 | -5.1507 | .40188 | 1 | .000 | -5.9384 | -4.3631 |
|  | 2 | 1 | 2.8093 | .17005 | 1 | .000 | 2.4760 | 3.1426 |
|  |  | 3 | -1.5402 | .27993 | 1 | .000 | -2.0889 | -.9915 |
|  |  | 4 | -2.3415 | .41277 | 1 | .000 | -3.1505 | -1.5324 |
|  | 3 | 1 | 4.3495 | .26306 | 1 | .000 | 3.8339 | 4.8651 |
|  |  | 2 | 1.5402 | .27993 | 1 | .000 | .9915 | 2.0889 |
|  |  | 4 | -.8013 | .45867 | 1 | .081 | -1.7002 | .0977 |
|  | 4 | 1 | 5.1507 | .40188 | 1 | .000 | 4.3631 | 5.9384 |
|  |  | 2 | 2.3415 | .41277 | 1 | .000 | 1.5324 | 3.1505 |
|  |  | 3 | .8013 | .45867 | 1 | .081 | -.0977 | 1.7002 |
| 2022 | 1 | 2 | -2.4771 | .09679 | 1 | .000 | -2.6668 | -2.2873 |
|  |  | 3 | -4.1347 | .15849 | 1 | .000 | -4.4453 | -3.8241 |
|  |  | 4 | -5.1164 | .32506 | 1 | .000 | -5.7535 | -4.4793 |
|  | 2 | 1 | 2.4771 | .09679 | 1 | .000 | 2.2873 | 2.6668 |
|  |  | 3 | -1.6576 | .16550 | 1 | .000 | -1.9820 | -1.3333 |
|  |  | 4 | -2.6393 | .32852 | 1 | .000 | -3.2832 | -1.9954 |
|  | 3 | 1 | 4.1347 | .15849 | 1 | .000 | 3.8241 | 4.4453 |
|  |  | 2 | 1.6576 | .16550 | 1 | .000 | 1.3333 | 1.9820 |
|  |  | 4 | -.9817 | .35170 | 1 | .005 | -1.6710 | -.2924 |
|  | 4 | 1 | 5.1164 | .32506 | 1 | .000 | 4.4793 | 5.7535 |
|  |  | 2 | 2.6393 | .32852 | 1 | .000 | 1.9954 | 3.2832 |
|  |  | 3 | .9817 | .35170 | 1 | .005 | .2924 | 1.6710 |
| 2023 | 1 | 2 | -2.5030 | .10451 | 1 | .000 | -2.7078 | -2.2981 |
|  |  | 3 | -4.0827 | .14655 | 1 | .000 | -4.3699 | -3.7955 |
|  |  | 4 | -5.2854 | .18744 | 1 | .000 | -5.6528 | -4.9180 |
|  | 2 | 1 | 2.5030 | .10451 | 1 | .000 | 2.2981 | 2.7078 |
|  |  | 3 | -1.5797 | .15230 | 1 | .000 | -1.8782 | -1.2812 |
|  |  | 4 | -2.7825 | .19207 | 1 | .000 | -3.1589 | -2.4060 |
|  | 3 | 1 | 4.0827 | .14655 | 1 | .000 | 3.7955 | 4.3699 |
|  |  | 2 | 1.5797 | .15230 | 1 | .000 | 1.2812 | 1.8782 |
|  |  | 4 | -1.2027 | .21767 | 1 | .000 | -1.6294 | -.7761 |
|  | 4 | 1 | 5.2854 | .18744 | 1 | .000 | 4.9180 | 5.6528 |
|  |  | 2 | 2.7825 | .19207 | 1 | .000 | 2.4060 | 3.1589 |
|  |  | 3 | 1.2027 | .21767 | 1 | .000 | .7761 | 1.6294 |
| 2024 | 1 | 2 | -2.5557 | .09889 | 1 | .000 | -2.7496 | -2.3619 |
|  |  | 3 | -4.3119 | .14751 | 1 | .000 | -4.6010 | -4.0227 |
|  |  | 4 | -5.5704 | .19986 | 1 | .000 | -5.9622 | -5.1787 |
|  | 2 | 1 | 2.5557 | .09889 | 1 | .000 | 2.3619 | 2.7496 |
|  |  | 3 | -1.7561 | .15685 | 1 | .000 | -2.0636 | -1.4487 |
|  |  | 4 | -3.0147 | .20679 | 1 | .000 | -3.4200 | -2.6094 |
|  | 3 | 1 | 4.3119 | .14751 | 1 | .000 | 4.0227 | 4.6010 |
|  |  | 2 | 1.7561 | .15685 | 1 | .000 | 1.4487 | 2.0636 |
|  |  | 4 | -1.2586 | .23340 | 1 | .000 | -1.7160 | -.8011 |
|  | 4 | 1 | 5.5704 | .19986 | 1 | .000 | 5.1787 | 5.9622 |
|  |  | 2 | 3.0147 | .20679 | 1 | .000 | 2.6094 | 3.4200 |
|  |  | 3 | 1.2586 | .23340 | 1 | .000 | .8011 | 1.7160 |
| 2025 | 1 | 2 | -2.3164 | .12785 | 1 | .000 | -2.5670 | -2.0658 |
|  |  | 3 | -3.7426 | .21516 | 1 | .000 | -4.1643 | -3.3209 |
|  |  | 4 | -5.1888 | .32097 | 1 | .000 | -5.8179 | -4.5597 |
|  | 2 | 1 | 2.3164 | .12785 | 1 | .000 | 2.0658 | 2.5670 |
|  |  | 3 | -1.4262 | .22382 | 1 | .000 | -1.8648 | -.9875 |
|  |  | 4 | -2.8724 | .32626 | 1 | .000 | -3.5118 | -2.2329 |
|  | 3 | 1 | 3.7426 | .21516 | 1 | .000 | 3.3209 | 4.1643 |
|  |  | 2 | 1.4262 | .22382 | 1 | .000 | .9875 | 1.8648 |
|  |  | 4 | -1.4462 | .36976 | 1 | .000 | -2.1709 | -.7215 |
|  | 4 | 1 | 5.1888 | .32097 | 1 | .000 | 4.5597 | 5.8179 |
|  |  | 2 | 2.8724 | .32626 | 1 | .000 | 2.2329 | 3.5118 |
|  |  | 3 | 1.4462 | .36976 | 1 | .000 | .7215 | 2.1709 |

| **Overall Test** | | | |
| --- | --- | --- | --- |
| Year | Wald χ² | Degrees of Freedom | P |
| 2020 | 163.453 | 3 | .000 |
| 2021 | 540.706 | 3 | .000 |
| 2022 | 1229.292 | 3 | .000 |
| 2023 | 1487.025 | 3 | .000 |
| 2024 | 1706.232 | 3 | .000 |
| 2025 | 675.594 | 3 | .000 |

**Estimated Marginal Means 9：Year* Diagnostic**

| **Estimate** | | | | | |
| --- | --- | --- | --- | --- | --- |
| Year | Diagnostic | Mean | Standard Error | 95% Wald Confidence Interval | |
|  |  |  |  | Lower Bound | Upper Bound |
| 2020 | 1 | 6.9503 | .24506 | 6.4862 | 7.4475 |
|  | 2 | 7.3386 | .23155 | 6.8985 | 7.8067 |
| 2021 | 1 | 7.4638 | .17768 | 7.1235 | 7.8203 |
|  | 2 | 7.0531 | .11881 | 6.8240 | 7.2898 |
| 2022 | 1 | 7.0822 | .11955 | 6.8517 | 7.3204 |
|  | 2 | 6.9785 | .07882 | 6.8257 | 7.1347 |
| 2023 | 1 | 7.2345 | .09575 | 7.0493 | 7.4247 |
|  | 2 | 7.3814 | .06190 | 7.2611 | 7.5037 |
| 2024 | 1 | 7.2468 | .10533 | 7.0433 | 7.4562 |
|  | 2 | 7.5066 | .05807 | 7.3936 | 7.6212 |
| 2025 | 1 | 7.2015 | .12491 | 6.9608 | 7.4506 |
|  | 2 | 7.0420 | .09817 | 6.8522 | 7.2370 |

| **Pairwise Comparisons** | | | | | | | | |
| --- | --- | --- | --- | --- | --- | --- | --- | --- |
| Diagnostic | (I) Year | (J) Year | Mean Difference (I-J) | Standard Error | Degrees of Freedom | P | 95% Wald Confidence Interval | |
|  |  |  |  |  |  |  | Lower Bound | Upper Bound |
| 1 | 2020 | 2021 | -.5135 | .30180 | 1 | .089 | -1.1050 | .0780 |
|  |  | 2022 | -.1319 | .27159 | 1 | .627 | -.6642 | .4004 |
|  |  | 2023 | -.2843 | .26245 | 1 | .279 | -.7987 | .2301 |
|  |  | 2024 | -.2965 | .26591 | 1 | .265 | -.8177 | .2246 |
|  |  | 2025 | -.2513 | .27411 | 1 | .359 | -.7885 | .2859 |
|  | 2021 | 2020 | .5135 | .30180 | 1 | .089 | -.0780 | 1.1050 |
|  |  | 2022 | .3816 | .21041 | 1 | .070 | -.0308 | .7940 |
|  |  | 2023 | .2292 | .19932 | 1 | .250 | -.1614 | .6199 |
|  |  | 2024 | .2170 | .20393 | 1 | .287 | -.1827 | .6167 |
|  |  | 2025 | .2622 | .21501 | 1 | .223 | -.1592 | .6837 |
|  | 2022 | 2020 | .1319 | .27159 | 1 | .627 | -.4004 | .6642 |
|  |  | 2021 | -.3816 | .21041 | 1 | .070 | -.7940 | .0308 |
|  |  | 2023 | -.1524 | .14969 | 1 | .309 | -.4458 | .1410 |
|  |  | 2024 | -.1646 | .15553 | 1 | .290 | -.4695 | .1402 |
|  |  | 2025 | -.1194 | .16997 | 1 | .482 | -.4525 | .2137 |
|  | 2023 | 2020 | .2843 | .26245 | 1 | .279 | -.2301 | .7987 |
|  |  | 2021 | -.2292 | .19932 | 1 | .250 | -.6199 | .1614 |
|  |  | 2022 | .1524 | .14969 | 1 | .309 | -.1410 | .4458 |
|  |  | 2024 | -.0122 | .13985 | 1 | .930 | -.2863 | .2619 |
|  |  | 2025 | .0330 | .15548 | 1 | .832 | -.2717 | .3377 |
|  | 2024 | 2020 | .2965 | .26591 | 1 | .265 | -.2246 | .8177 |
|  |  | 2021 | -.2170 | .20393 | 1 | .287 | -.6167 | .1827 |
|  |  | 2022 | .1646 | .15553 | 1 | .290 | -.1402 | .4695 |
|  |  | 2023 | .0122 | .13985 | 1 | .930 | -.2619 | .2863 |
|  |  | 2025 | .0452 | .16119 | 1 | .779 | -.2707 | .3612 |
|  | 2025 | 2020 | .2513 | .27411 | 1 | .359 | -.2859 | .7885 |
|  |  | 2021 | -.2622 | .21501 | 1 | .223 | -.6837 | .1592 |
|  |  | 2022 | .1194 | .16997 | 1 | .482 | -.2137 | .4525 |
|  |  | 2023 | -.0330 | .15548 | 1 | .832 | -.3377 | .2717 |
|  |  | 2024 | -.0452 | .16119 | 1 | .779 | -.3612 | .2707 |
| 2 | 2020 | 2021 | .2855 | .25895 | 1 | .270 | -.2221 | .7930 |
|  |  | 2022 | .3601 | .24325 | 1 | .139 | -.1166 | .8369 |
|  |  | 2023 | -.0429 | .23910 | 1 | .858 | -.5115 | .4258 |
|  |  | 2024 | -.1680 | .23812 | 1 | .481 | -.6347 | .2987 |
|  |  | 2025 | .2966 | .24975 | 1 | .235 | -.1929 | .7861 |
|  | 2021 | 2020 | -.2855 | .25895 | 1 | .270 | -.7930 | .2221 |
|  |  | 2022 | .0746 | .14136 | 1 | .598 | -.2024 | .3517 |
|  |  | 2023 | -.3283 | .13344 | 1 | .014 | -.5899 | -.0668 |
|  |  | 2024 | -.4535 | .13173 | 1 | .001 | -.7117 | -.1953 |
|  |  | 2025 | .0111 | .15266 | 1 | .942 | -.2881 | .3103 |
|  | 2022 | 2020 | -.3601 | .24325 | 1 | .139 | -.8369 | .1166 |
|  |  | 2021 | -.0746 | .14136 | 1 | .598 | -.3517 | .2024 |
|  |  | 2023 | -.4030 | .09946 | 1 | .000 | -.5979 | -.2080 |
|  |  | 2024 | -.5281 | .09719 | 1 | .000 | -.7186 | -.3376 |
|  |  | 2025 | -.0635 | .12406 | 1 | .609 | -.3067 | .1796 |
|  | 2023 | 2020 | .0429 | .23910 | 1 | .858 | -.4258 | .5115 |
|  |  | 2021 | .3283 | .13344 | 1 | .014 | .0668 | .5899 |
|  |  | 2022 | .4030 | .09946 | 1 | .000 | .2080 | .5979 |
|  |  | 2024 | -.1251 | .08444 | 1 | .138 | -.2906 | .0404 |
|  |  | 2025 | .3395 | .11515 | 1 | .003 | .1138 | .5652 |
|  | 2024 | 2020 | .1680 | .23812 | 1 | .481 | -.2987 | .6347 |
|  |  | 2021 | .4535 | .13173 | 1 | .001 | .1953 | .7117 |
|  |  | 2022 | .5281 | .09719 | 1 | .000 | .3376 | .7186 |
|  |  | 2023 | .1251 | .08444 | 1 | .138 | -.0404 | .2906 |
|  |  | 2025 | .4646 | .11322 | 1 | .000 | .2427 | .6865 |
|  | 2025 | 2020 | -.2966 | .24975 | 1 | .235 | -.7861 | .1929 |
|  |  | 2021 | -.0111 | .15266 | 1 | .942 | -.3103 | .2881 |
|  |  | 2022 | .0635 | .12406 | 1 | .609 | -.1796 | .3067 |
|  |  | 2023 | -.3395 | .11515 | 1 | .003 | -.5652 | -.1138 |
|  |  | 2024 | -.4646 | .11322 | 1 | .000 | -.6865 | -.2427 |

| **Overall Test** | | | |
| --- | --- | --- | --- |
| Diagnostic | Wald χ² | Degrees of Freedom | P |
| 1 | 4.631 | 5 | .463 |
| 2 | 42.272 | 5 | .000 |

**Estimated Marginal Means 10：Year* Diagnostic**

| **Estimate** | | | | | |
| --- | --- | --- | --- | --- | --- |
| Year | Diagnostic | Mean | Standard Error | 95% Wald Confidence Interval | |
|  |  |  |  | Lower Bound | Upper Bound |
| 2020 | 1 | 6.9503 | .24506 | 6.4862 | 7.4475 |
|  | 2 | 7.3386 | .23155 | 6.8985 | 7.8067 |
| 2021 | 1 | 7.4638 | .17768 | 7.1235 | 7.8203 |
|  | 2 | 7.0531 | .11881 | 6.8240 | 7.2898 |
| 2022 | 1 | 7.0822 | .11955 | 6.8517 | 7.3204 |
|  | 2 | 6.9785 | .07882 | 6.8257 | 7.1347 |
| 2023 | 1 | 7.2345 | .09575 | 7.0493 | 7.4247 |
|  | 2 | 7.3814 | .06190 | 7.2611 | 7.5037 |
| 2024 | 1 | 7.2468 | .10533 | 7.0433 | 7.4562 |
|  | 2 | 7.5066 | .05807 | 7.3936 | 7.6212 |
| 2025 | 1 | 7.2015 | .12491 | 6.9608 | 7.4506 |
|  | 2 | 7.0420 | .09817 | 6.8522 | 7.2370 |

| **Pairwise Comparisons** | | | | | | | | |
| --- | --- | --- | --- | --- | --- | --- | --- | --- |
| Year | (I) Diagnostic | (J) Diagnostic | Mean Difference (I-J) | Standard Error | Degrees of Freedom | P | 95% Wald Confidence Interval | |
|  |  |  |  |  |  |  | Lower Bound | Upper Bound |
| 2020 | 1 | 2 | -.3883 | .30237 | 1 | .199 | -.9810 | .2043 |
|  | 2 | 1 | .3883 | .30237 | 1 | .199 | -.2043 | .9810 |
| 2021 | 1 | 2 | .4107 | .20107 | 1 | .041 | .0166 | .8048 |
|  | 2 | 1 | -.4107 | .20107 | 1 | .041 | -.8048 | -.0166 |
| 2022 | 1 | 2 | .1037 | .12776 | 1 | .417 | -.1467 | .3541 |
|  | 2 | 1 | -.1037 | .12776 | 1 | .417 | -.3541 | .1467 |
| 2023 | 1 | 2 | -.1469 | .11211 | 1 | .190 | -.3666 | .0728 |
|  | 2 | 1 | .1469 | .11211 | 1 | .190 | -.0728 | .3666 |
| 2024 | 1 | 2 | -.2598 | .11797 | 1 | .028 | -.4910 | -.0285 |
|  | 2 | 1 | .2598 | .11797 | 1 | .028 | .0285 | .4910 |
| 2025 | 1 | 2 | .1596 | .14924 | 1 | .285 | -.1329 | .4521 |
|  | 2 | 1 | -.1596 | .14924 | 1 | .285 | -.4521 | .1329 |

| **Overall Test** | | | |
| --- | --- | --- | --- |
| Year | Wald χ² | Degrees of Freedom | P |
| 2020 | 1.649 | 1 | .199 |
| 2021 | 4.172 | 1 | .041 |
| 2022 | .659 | 1 | .417 |
| 2023 | 1.717 | 1 | .190 |
| 2024 | 4.848 | 1 | .028 |
| 2025 | 1.143 | 1 | .285 |

**Estimated Marginal Means 11：Gender* Age**

| **Estimate** | | | | | |
| --- | --- | --- | --- | --- | --- |
| Gender | Age | Mean | Standard Error | 95% Wald Confidence Interval | |
|  |  |  |  | Lower Bound | Upper Bound |
| 1 | 1 | 4.4854 | .04904 | 4.3903 | 4.5826 |
|  | 2 | 6.8528 | .06417 | 6.7281 | 6.9797 |
|  | 3 | 8.5173 | .13630 | 8.2543 | 8.7886 |
|  | 4 | 9.7743 | .16872 | 9.4492 | 10.1107 |
| 2 | 1 | 4.5803 | .06138 | 4.4616 | 4.7022 |
|  | 2 | 7.1969 | .07459 | 7.0522 | 7.3446 |
|  | 3 | 8.7126 | .15464 | 8.4147 | 9.0210 |
|  | 4 | 9.8744 | .19633 | 9.4970 | 10.2668 |

| **Pairwise Comparisons** | | | | | | | | |
| --- | --- | --- | --- | --- | --- | --- | --- | --- |
| Age | (I) Gender | (J) Gender | Mean Difference (I-J) | Standard Error | Degrees of Freedom | P | 95% Wald Confidence Interval | |
|  |  |  |  |  |  |  | Lower Bound | Upper Bound |
| 1 | 1 | 2 | -.0949 | .06728 | 1 | .158 | -.2268 | .0369 |
|  | 2 | 1 | .0949 | .06728 | 1 | .158 | -.0369 | .2268 |
| 2 | 1 | 2 | -.3441 | .09073 | 1 | .000 | -.5220 | -.1663 |
|  | 2 | 1 | .3441 | .09073 | 1 | .000 | .1663 | .5220 |
| 3 | 1 | 2 | -.1953 | .15196 | 1 | .199 | -.4932 | .1025 |
|  | 2 | 1 | .1953 | .15196 | 1 | .199 | -.1025 | .4932 |
| 4 | 1 | 2 | -.1001 | .21761 | 1 | .646 | -.5266 | .3264 |
|  | 2 | 1 | .1001 | .21761 | 1 | .646 | -.3264 | .5266 |

| **Overall Test** | | | |
| --- | --- | --- | --- |
| Age | Wald χ² | Degrees of Freedom | P |
| 1 | 1.991 | 1 | .158 |
| 2 | 14.385 | 1 | .000 |
| 3 | 1.652 | 1 | .199 |
| 4 | .212 | 1 | .646 |

**Estimated Marginal Means 12：Gender* Age**

| **Estimate** | | | | | |
| --- | --- | --- | --- | --- | --- |
| Gender | Age | Mean | Standard Error | 95% Wald Confidence Interval | |
|  |  |  |  | Lower Bound | Upper Bound |
| 1 | 1 | 4.4854 | .04904 | 4.3903 | 4.5826 |
|  | 2 | 6.8528 | .06417 | 6.7281 | 6.9797 |
|  | 3 | 8.5173 | .13630 | 8.2543 | 8.7886 |
|  | 4 | 9.7743 | .16872 | 9.4492 | 10.1107 |
| 2 | 1 | 4.5803 | .06138 | 4.4616 | 4.7022 |
|  | 2 | 7.1969 | .07459 | 7.0522 | 7.3446 |
|  | 3 | 8.7126 | .15464 | 8.4147 | 9.0210 |
|  | 4 | 9.8744 | .19633 | 9.4970 | 10.2668 |

| **Pairwise Comparisons** | | | | | | | | |
| --- | --- | --- | --- | --- | --- | --- | --- | --- |
| Gender | (I) Age | (J) Age | Mean Difference (I-J) | Standard Error | Degrees of Freedom | P | 95% Wald Confidence Interval | |
|  |  |  |  |  |  |  | Lower Bound | Upper Bound |
| 1 | 1 | 2 | -2.3674 | .07776 | 1 | .000 | -2.5198 | -2.2149 |
|  |  | 3 | -4.0319 | .14279 | 1 | .000 | -4.3117 | -3.7520 |
|  |  | 4 | -5.2889 | .17467 | 1 | .000 | -5.6313 | -4.9466 |
|  | 2 | 1 | 2.3674 | .07776 | 1 | .000 | 2.2149 | 2.5198 |
|  |  | 3 | -1.6645 | .14699 | 1 | .000 | -1.9526 | -1.3764 |
|  |  | 4 | -2.9216 | .17731 | 1 | .000 | -3.2691 | -2.5741 |
|  | 3 | 1 | 4.0319 | .14279 | 1 | .000 | 3.7520 | 4.3117 |
|  |  | 2 | 1.6645 | .14699 | 1 | .000 | 1.3764 | 1.9526 |
|  |  | 4 | -1.2571 | .21431 | 1 | .000 | -1.6771 | -.8370 |
|  | 4 | 1 | 5.2889 | .17467 | 1 | .000 | 4.9466 | 5.6313 |
|  |  | 2 | 2.9216 | .17731 | 1 | .000 | 2.5741 | 3.2691 |
|  |  | 3 | 1.2571 | .21431 | 1 | .000 | .8370 | 1.6771 |
| 2 | 1 | 2 | -2.6165 | .09010 | 1 | .000 | -2.7931 | -2.4400 |
|  |  | 3 | -4.1322 | .16001 | 1 | .000 | -4.4458 | -3.8186 |
|  |  | 4 | -5.2941 | .20091 | 1 | .000 | -5.6879 | -4.9003 |
|  | 2 | 1 | 2.6165 | .09010 | 1 | .000 | 2.4400 | 2.7931 |
|  |  | 3 | -1.5157 | .16540 | 1 | .000 | -1.8399 | -1.1915 |
|  |  | 4 | -2.6775 | .20443 | 1 | .000 | -3.0782 | -2.2769 |
|  | 3 | 1 | 4.1322 | .16001 | 1 | .000 | 3.8186 | 4.4458 |
|  |  | 2 | 1.5157 | .16540 | 1 | .000 | 1.1915 | 1.8399 |
|  |  | 4 | -1.1619 | .24221 | 1 | .000 | -1.6366 | -.6871 |
|  | 4 | 1 | 5.2941 | .20091 | 1 | .000 | 4.9003 | 5.6879 |
|  |  | 2 | 2.6775 | .20443 | 1 | .000 | 2.2769 | 3.0782 |
|  |  | 3 | 1.1619 | .24221 | 1 | .000 | .6871 | 1.6366 |

| **Overall Test** | | | |
| --- | --- | --- | --- |
| Gender | Wald χ² | Degrees of Freedom | P |
| 1 | 1977.845 | 3 | .000 |
| 2 | 1635.533 | 3 | .000 |

**Estimated Marginal Means 13：Gender* Diagnostic**

| **Estimate** | | | | | |
| --- | --- | --- | --- | --- | --- |
| Gender | Diagnostic | Mean | Standard Error | 95% Wald Confidence Interval | |
|  |  |  |  | Lower Bound | Upper Bound |
| 1 | 1 | 7.1652 | .07698 | 7.0159 | 7.3177 |
|  | 2 | 7.0599 | .05945 | 6.9443 | 7.1774 |
| 2 | 1 | 7.2245 | .09348 | 7.0436 | 7.4101 |
|  | 2 | 7.3713 | .07120 | 7.2330 | 7.5122 |

| **Pairwise Comparisons** | | | | | | | | |
| --- | --- | --- | --- | --- | --- | --- | --- | --- |
| Diagnostic | (I) Gender | (J) Gender | Mean Difference (I-J) | Standard Error | Degrees of Freedom | P | 95% Wald Confidence Interval | |
|  |  |  |  |  |  |  | Lower Bound | Upper Bound |
| 1 | 1 | 2 | -.0593 | .10929 | 1 | .587 | -.2735 | .1549 |
|  | 2 | 1 | .0593 | .10929 | 1 | .587 | -.1549 | .2735 |
| 2 | 1 | 2 | -.3113 | .08107 | 1 | .000 | -.4702 | -.1524 |
|  | 2 | 1 | .3113 | .08107 | 1 | .000 | .1524 | .4702 |

| **Overall Test** | | | |
| --- | --- | --- | --- |
| Diagnostic | Wald χ² | Degrees of Freedom | P |
| 1 | .295 | 1 | .587 |
| 2 | 14.748 | 1 | .000 |

**Estimated Marginal Means 14：Gender* Diagnostic**

| **Estimate** | | | | | |
| --- | --- | --- | --- | --- | --- |
| Gender | Diagnostic | Mean | Standard Error | 95% Wald Confidence Interval | |
|  |  |  |  | Lower Bound | Upper Bound |
| 1 | 1 | 7.1652 | .07698 | 7.0159 | 7.3177 |
|  | 2 | 7.0599 | .05945 | 6.9443 | 7.1774 |
| 2 | 1 | 7.2245 | .09348 | 7.0436 | 7.4101 |
|  | 2 | 7.3713 | .07120 | 7.2330 | 7.5122 |

| **Pairwise Comparisons** | | | | | | | | |
| --- | --- | --- | --- | --- | --- | --- | --- | --- |
| Gender | (I) Diagnostic | (J) Diagnostic | Mean Difference (I-J) | Standard Error | Degrees of Freedom | P | 95% Wald Confidence Interval | |
|  |  |  |  |  |  |  | Lower Bound | Upper Bound |
| 1 | 1 | 2 | .1053 | .09023 | 1 | .243 | -.0716 | .2821 |
|  | 2 | 1 | -.1053 | .09023 | 1 | .243 | -.2821 | .0716 |
| 2 | 1 | 2 | -.1467 | .10577 | 1 | .165 | -.3540 | .0606 |
|  | 2 | 1 | .1467 | .10577 | 1 | .165 | -.0606 | .3540 |

| **Overall Test** | | | |
| --- | --- | --- | --- |
| Gender | Wald χ² | Degrees of Freedom | P |
| 1 | 1.361 | 1 | .243 |
| 2 | 1.925 | 1 | .165 |

**Estimated Marginal Means 15：Age* Diagnostic**

| **Estimate** | | | | | |
| --- | --- | --- | --- | --- | --- |
| Age | Diagnostic | Mean | Standard Error | 95% Wald Confidence Interval | |
|  |  |  |  | Lower Bound | Upper Bound |
| 1 | 1 | 4.5566 | .07529 | 4.4114 | 4.7066 |
|  | 2 | 4.5088 | .03857 | 4.4338 | 4.5850 |
| 2 | 1 | 6.8159 | .07616 | 6.6683 | 6.9669 |
|  | 2 | 7.2358 | .06351 | 7.1123 | 7.3613 |
| 3 | 1 | 8.7280 | .16444 | 8.4116 | 9.0563 |
|  | 2 | 8.5022 | .13088 | 8.2495 | 8.7626 |
| 4 | 1 | 9.8853 | .20068 | 9.4997 | 10.2865 |
|  | 2 | 9.7636 | .17571 | 9.4252 | 10.1141 |

| **Pairwise Comparisons** | | | | | | | | |
| --- | --- | --- | --- | --- | --- | --- | --- | --- |
| Diagnostic | (I) Age | (J) Age | Mean Difference (I-J) | Standard Error | Degrees of Freedom | P | 95% Wald Confidence Interval | |
|  |  |  |  |  |  |  | Lower Bound | Upper Bound |
| 1 | 1 | 2 | -2.2593 | .10238 | 1 | .000 | -2.4600 | -2.0587 |
|  |  | 3 | -4.1714 | .17586 | 1 | .000 | -4.5160 | -3.8267 |
|  |  | 4 | -5.3287 | .21150 | 1 | .000 | -5.7432 | -4.9141 |
|  | 2 | 1 | 2.2593 | .10238 | 1 | .000 | 2.0587 | 2.4600 |
|  |  | 3 | -1.9121 | .17593 | 1 | .000 | -2.2569 | -1.5672 |
|  |  | 4 | -3.0693 | .21190 | 1 | .000 | -3.4847 | -2.6540 |
|  | 3 | 1 | 4.1714 | .17586 | 1 | .000 | 3.8267 | 4.5160 |
|  |  | 2 | 1.9121 | .17593 | 1 | .000 | 1.5672 | 2.2569 |
|  |  | 4 | -1.1573 | .25540 | 1 | .000 | -1.6579 | -.6567 |
|  | 4 | 1 | 5.3287 | .21150 | 1 | .000 | 4.9141 | 5.7432 |
|  |  | 2 | 3.0693 | .21190 | 1 | .000 | 2.6540 | 3.4847 |
|  |  | 3 | 1.1573 | .25540 | 1 | .000 | .6567 | 1.6579 |
| 2 | 1 | 2 | -2.7270 | .07263 | 1 | .000 | -2.8694 | -2.5847 |
|  |  | 3 | -3.9935 | .13509 | 1 | .000 | -4.2582 | -3.7287 |
|  |  | 4 | -5.2549 | .17824 | 1 | .000 | -5.6042 | -4.9055 |
|  | 2 | 1 | 2.7270 | .07263 | 1 | .000 | 2.5847 | 2.8694 |
|  |  | 3 | -1.2665 | .14197 | 1 | .000 | -1.5447 | -.9882 |
|  |  | 4 | -2.5278 | .18169 | 1 | .000 | -2.8840 | -2.1717 |
|  | 3 | 1 | 3.9935 | .13509 | 1 | .000 | 3.7287 | 4.2582 |
|  |  | 2 | 1.2665 | .14197 | 1 | .000 | .9882 | 1.5447 |
|  |  | 4 | -1.2614 | .21371 | 1 | .000 | -1.6803 | -.8425 |
|  | 4 | 1 | 5.2549 | .17824 | 1 | .000 | 4.9055 | 5.6042 |
|  |  | 2 | 2.5278 | .18169 | 1 | .000 | 2.1717 | 2.8840 |
|  |  | 3 | 1.2614 | .21371 | 1 | .000 | .8425 | 1.6803 |

| **Overall Test** | | | |
| --- | --- | --- | --- |
| Diagnostic | Wald χ² | Degrees of Freedom | P |
| 1 | 1183.154 | 3 | .000 |
| 2 | 2464.067 | 3 | .000 |

**Estimated Marginal Means 16：Age* Diagnostic**

| **Estimate** | | | | | |
| --- | --- | --- | --- | --- | --- |
| Age | Diagnostic | Mean | Standard Error | 95% Wald Confidence Interval | |
|  |  |  |  | Lower Bound | Upper Bound |
| 1 | 1 | 4.5566 | .07529 | 4.4114 | 4.7066 |
|  | 2 | 4.5088 | .03857 | 4.4338 | 4.5850 |
| 2 | 1 | 6.8159 | .07616 | 6.6683 | 6.9669 |
|  | 2 | 7.2358 | .06351 | 7.1123 | 7.3613 |
| 3 | 1 | 8.7280 | .16444 | 8.4116 | 9.0563 |
|  | 2 | 8.5022 | .13088 | 8.2495 | 8.7626 |
| 4 | 1 | 9.8853 | .20068 | 9.4997 | 10.2865 |
|  | 2 | 9.7636 | .17571 | 9.4252 | 10.1141 |

| **Pairwise Comparisons** | | | | | | | | |
| --- | --- | --- | --- | --- | --- | --- | --- | --- |
| Age | (I) Diagnostic | (J) Diagnostic | Mean Difference (I-J) | Standard Error | Degrees of Freedom | P | 95% Wald Confidence Interval | |
|  |  |  |  |  |  |  | Lower Bound | Upper Bound |
| 1 | 1 | 2 | .0479 | .08049 | 1 | .552 | -.1099 | .2056 |
|  | 2 | 1 | -.0479 | .08049 | 1 | .552 | -.2056 | .1099 |
| 2 | 1 | 2 | -.4198 | .09339 | 1 | .000 | -.6029 | -.2368 |
|  | 2 | 1 | .4198 | .09339 | 1 | .000 | .2368 | .6029 |
| 3 | 1 | 2 | .2258 | .16221 | 1 | .164 | -.0921 | .5437 |
|  | 2 | 1 | -.2258 | .16221 | 1 | .164 | -.5437 | .0921 |
| 4 | 1 | 2 | .1217 | .23583 | 1 | .606 | -.3405 | .5839 |
|  | 2 | 1 | -.1217 | .23583 | 1 | .606 | -.5839 | .3405 |

| **Overall Test** | | | |
| --- | --- | --- | --- |
| Age | Wald χ² | Degrees of Freedom | P |
| 1 | .354 | 1 | .552 |
| 2 | 20.207 | 1 | .000 |
| 3 | 1.938 | 1 | .164 |
| 4 | .266 | 1 | .606 |
