# Supplementary material for: The impact of COVID-19 social isolation and reduced microbial exposure on the immune system in children: a retrospective study
Source: PeerJ. 2026 Jul 7;14:e21469. doi: 10.7717/peerj.21469 (PMC13353229; doi:10.7717/peerj.21469)
Supplement: Supplemental Information 12 [file peerj-14-21469-s012.doc]

STROBE Statement—Checklist of items that should be included in reports of ***case-control studies***

|  | Item No | Recommendation |
| --- | --- | --- |
| **Title and abstract** | 1 | 1. Indicate the study’s design with a commonly used term in the title or the abstract   Line 1-38 |
|  |  | 1. Provide in the abstract an informative and balanced summary of what was done and what was found   Line 1-38 |
| Introduction | | |
| Background/rationale | 2 | Explain the scientific background and rationale for the investigation being reported  Line 41-59 |
| Objectives | 3 | State specific objectives, including any prespecified hypotheses  Line 41-59 |
| Methods | | |
| Study design | 4 | Present key elements of study design early in the paper  Line 53-55 |
| Setting | 5 | Describe the setting, locations, and relevant dates, including periods of recruitment, exposure, follow-up, and data collection  Line 62-81 |
| Participants | 6 | 1. Give the eligibility criteria, and the sources and methods of case ascertainment and control selection. Give the rationale for the choice of cases and controls   Line 82-103 |
|  |  | 1. For matched studies, give matching criteria and the number of controls per case   Line 82-103 |
| Variables | 7 | Clearly define all outcomes, exposures, predictors, potential confounders, and effect modifiers. Give diagnostic criteria, if applicable  Line 82-91 |
| Data sources/ measurement | 8* | For each variable of interest, give sources of data and details of methods of assessment (measurement). Describe comparability of assessment methods if there is more than one group  Line 106-114 |
| Bias | 9 | Describe any efforts to address potential sources of bias  Line 162-170 |
| Study size | 10 | Explain how the study size was arrived at  Line 41-53 |
| Quantitative variables | 11 | Explain how quantitative variables were handled in the analyses. If applicable, describe which groupings were chosen and why  Line 106-114 |
| Statistical methods | 12 | 1. Describe all statistical methods, including those used to control for confounding   Line 106-114 |
|  |  | 1. Describe any methods used to examine subgroups and interactions   Line 106-114 |
|  |  | 1. Explain how missing data were addressed   Line 106-114 |
|  |  | 1. If applicable, explain how matching of cases and controls was addressed   Line 106-114 |
|  |  | 1. Describe any sensitivity analyses   Line 106-114 |
| Results | | |
| Participants | 13* | 1. Report numbers of individuals at each stage of study—eg numbers potentially eligible, examined for eligibility, confirmed eligible, included in the study, completing follow-up, and analysed   Line 63-68 |
|  |  | 1. Give reasons for non-participation at each stage   Line 63-68 |
|  |  | 1. Consider use of a flow diagram   Line 63-68 |
| Descriptive data | 14* | 1. Give characteristics of study participants (eg demographic, clinical, social) and information on exposures and potential confounders   Line 82-103 |
|  |  | 1. Indicate number of participants with missing data for each variable of interest   Line 82-103 |
| Outcome data | 15* | Report numbers in each exposure category, or summary measures of exposure  Line 63-68 |
| Main results | 16 | 1. Give unadjusted estimates and, if applicable, confounder-adjusted estimates and their precision (eg, 95% confidence interval). Make clear which confounders were adjusted for and why they were included   Line 171-380 |
|  |  | 1. Report category boundaries when continuous variables were categorized   Line 171-380 |
|  |  | 1. If relevant, consider translating estimates of relative risk into absolute risk for a meaningful time period   Line 171-380 |

| Other analyses | 17 | Report other analyses done—eg analyses of subgroups and interactions, and sensitivity analyses |
| --- | --- | --- |
| Discussion | | |
| Key results | 18 | Summarise key results with reference to study objectives |
| Limitations | 19 | Discuss limitations of the study, taking into account sources of potential bias or imprecision. Discuss both direction and magnitude of any potential bias |
| Interpretation | 20 | Give a cautious overall interpretation of results considering objectives, limitations, multiplicity of analyses, results from similar studies, and other relevant evidence |
| Generalisability | 21 | Discuss the generalisability (external validity) of the study results |
| Other information | | |
| Funding | 22 | Give the source of funding and the role of the funders for the present study and, if applicable, for the original study on which the present article is based |

*Give information separately for cases and controls.

**Note:** An Explanation and Elaboration article discusses each checklist item and gives methodological background and published examples of transparent reporting. The STROBE checklist is best used in conjunction with this article (freely available on the Web sites of PLoS Medicine at http://www.plosmedicine.org/, Annals of Internal Medicine at http://www.annals.org/, and Epidemiology at http://www.epidem.com/). Information on the STROBE Initiative is available at http://www.strobe-statement.org.
